# Ligand flexibility as a concept to unlock catalytic activity: acyclic carbenes for base-free transfer ruthenium hydrogenation catalysis

**DOI:** 10.1039/d5sc08206d

**Published:** 2025-12-31

**Authors:** Gianluca Righetti, Georgyi Koidan, Sergiy L. Filimonchuk, Svitlana Shishkina, Aleksandr Kostyuk, Martin Albrecht

**Affiliations:** a Department of Chemistry, Biochemistry, and Pharmaceutical Sciences, University of Bern Freiestrasse 3 3012 Bern Switzerland martin.albrecht@unibe.ch; b Department of Organophosphorus Chemistry, Institute of Organic Chemistry Academician Kukhar Str. 5 Kyiv-94 02094 Ukraine a.kostyuk@yahoo.com; c SSI Institute of Single Crystals, NAS of Ukraine 60 Nauky Ave. 61001 Kharkiv Ukraine

## Abstract

Many ligands are structurally rigid and well-defined, *e.g. N*-heterocyclic carbenes display a fan-like structure with a defined buried volume. Here, we break this dogma by introducing more flexibility around the catalytically active center by using acyclic (diamino)carbene (ADC) ligands. The ADC ligand was constructed in a straightforward protocol on the ruthenium center *via* methyl isocyanide coordination and subsequent reaction with amines such as pyrrolidine. Ligand flexibility in the formed (pyrrolidine)(methylamine)carbene ruthenium complex Ru-2 was demonstrated both in solution (variable temperature NMR) and in the solid state through crystallographic identification with the protic NH site oriented either distal or proximal to the ruthenium center. In contrast to their cyclic analogues, the Ru-ADC complexes are highly active in base-free transfer hydrogenation, with turnover numbers >1000. The base-free conditions allowed for the transformation of substrates with base-sensitive groups such as esters, amides, acids, and amines, substrates that typically fail to undergo transfer hydrogenation under classical conditions. The absence of base also enabled late-stage hydrogenation of more complex substrates, and it avoids complications such as corrosion attributed to KOH and related strong bases.

## Abstract 2

Acyclic (diamino)carbene ligands impart flexibility and allow for protic units to be inserted proximal to a catalytic metal center, which enables base-free transfer hydrogenation of substrates that are incompatible with classic conditions.

## Graphical abstract

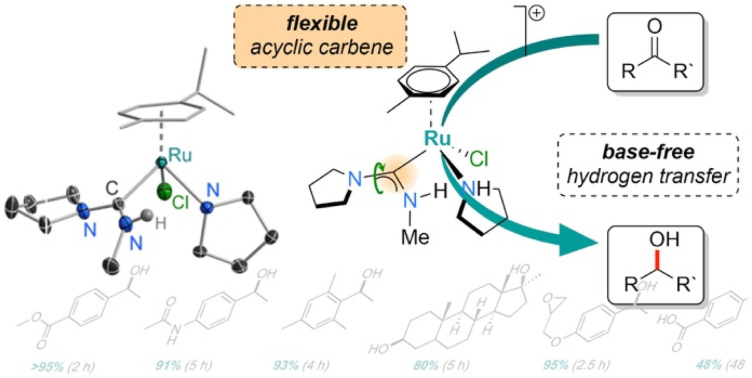

## Introduction

**Fig. 1:**
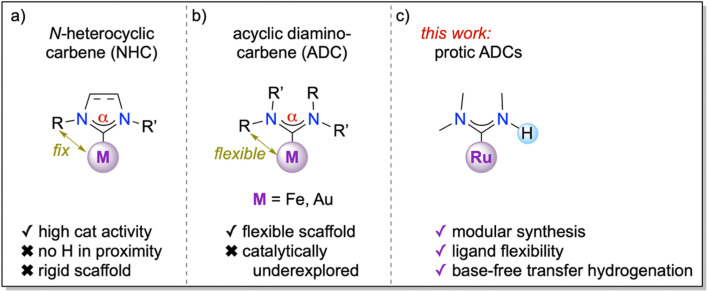
Schematic comparison of structural parameters of diamino carbene ligands: (a) cyclic diaminocarbenes (*N*-heterocyclic carbenes, NHCs) feature a rigid distance between the metal and the NHC wingtip groups, no flexibility in the N–C–N angle α, and no possibility for rotation about the carbene–nitrogen bond; (b) acyclic diaminocarbenes (ADCs) are flexible with respect to the N–C–N angle a and may rotate about the carbene–nitrogen bond, thus resulting in a dynamic positioning of the *N*-substituents with respect to the metal center, though complexes are only known for few metal centers and limited catalytic application; (c) this work introduces ruthenium complexes with ADC ligands that contain a proton in close proximity to the metal center and *cis*-coordinated ligands/substrates and demonstrates high catalytic activity of the complexes in base-free transfer hydrogenation.

A key feature of enzyme catalysis is the conformational flexibility in proximity to the catalytic pocket, which is both imparted and also restricted through weak interactions in the protein scaffold.^1^ In contrast, synthetic homogeneous catalysts based on metal complexes or organocatalysts are typically characterized by high rigidity. For example, classical phosphine ligands possess a fixed steric profile that is defined and classified by their cone angles,^2^ with perhaps the most notable exception of Buchwald phosphines, which contain a bulky biaryl substituent that may rotate in and out of the metal coordination sphere.^3^ Similarly, Arduengo-type *N*-heterocyclic carbenes (NHCs)^4,5^ are sterically rather rigid, especially with aryl wing-tip substituents that introduce fan-like sterics around the metal center (Fig. 1a)^6–8^ and result in well-defined buried volumes % *V*_bur_.^9^

The conformational flexibility of carbene ligands is, however, considerably enhanced in acyclic versions of NHCs, so-called acyclic (diamino)carbenes (ADCs, Fig. 1b).^10,11^ Specifically, ADCs offer a more flexible N–C–N angle α that can modulate the singlet–triplet gap and thus the donor properties of the carbene to the metal centre during catalytic transformations.^12^ Moreover, the two *N*-substituents R and R′ are, in principle, rotationally flexible about the C–N bond and may point towards the metal center or away, particularly when the C–N bond exhibits sufficient single bond character.^10–14^ Even though the very first diamino carbene complex was based on an ADC ligand,^15^ work in the last decades has focused almost exclusively on cyclic and hence rigid systems, with only few exceptions.^10–12,16–20^ For example, gold ADC complexes displayed promising catalytic activity in phenol synthesis and alkyne hydration,^21–26^ while iron ADC complexes were used to prepare mixed-metal assemblies.^27,28^

Attractive opportunities may emerge when one of the *N*-substituents is a hydrogen, as this feature introduces very low steric constraints, high lability of the substituent orientation, and a potential proton source in proximity to the metal center (Fig. 1c). Such ligand design features may be particularly advantageous when combined with a metal center that is active in redox-catalysis and that assumes a coordination geometry that allows a substrate to coordinate in *cis* position to the protic ligand. Here, we demonstrate that ADC ligands coordinated to an arene ruthenium(ii) synthon impart excellent activity in transfer hydrogenation, even under highly sought base-free conditions. The Ru–ADC complexes are readily accessible *via* a short, straightforward synthesis, which offers opportunities for facile ligand modulation.

## Results and discussion

**Scheme 1:**
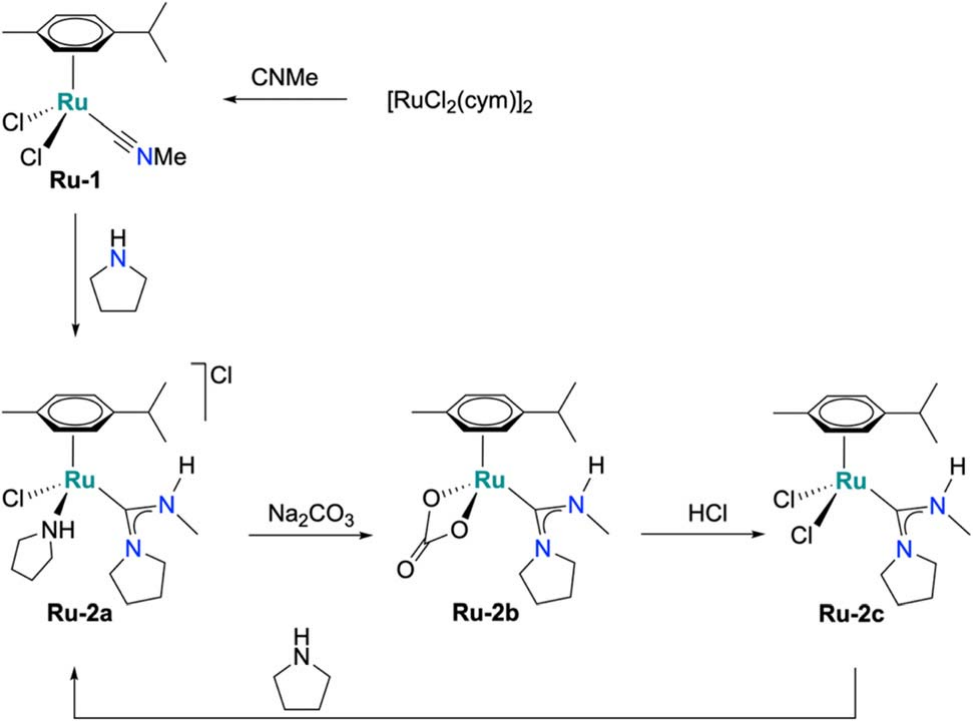
Synthetic protocol for the formation of Ru-ADC complexes (Ru-2a, Ru-2b, and Ru-2c by carbene ligand formation at the metal center.

A new family of ruthenium acyclic diamino carbene (Ru-ADC) complexes were synthesized from the isonitrile complex Ru-1, prepared from [RuCl_2_(cym)]_2_ with an excess of isocyanomethane at room temperature for 1 h (cym = *p*-cymene; Scheme 1). Reaction of Ru-1 with pyrrolidine afforded the ADC complex Ru-2a in 55% crystalline yield after acid extraction and recrystallization (Scheme 1).^29,30^ A short reaction time (30 min) was crucial to suppress *p*-cymene dissociation, while excess pyrrolidine (50 eqv.) promoted swift carbene formation. Treatment of complex Ru-2a with Na_2_CO_3_ yielded the carbonate complex Ru-2b (40%), and subsequent exposure to HCl gave the dichloride analogue Ru-2c (69%). Complexes Ru-2a and Ru-2c are air- and moisture-stable both in solution and in the solid state for several months. In contrast, the carbonate complex Ru-2b gradually decomposed in the solid state to an oil at room temperature, but remains stable at −20 °C. The reduced stability may be attributed to residual impurities, as indicated by deviations in elemental analysis of recrystallized samples (C: −1.34%, N: −0.42%).

Formation of the new acyclic carbene Ru complexes was evidenced by the characteristic high-frequency ^13^C NMR signals of the carbenic carbon at 209 ppm (Ru-2a), 203 ppm (Ru-2b), and 209 ppm (Ru-2c). Moreover, the N–CH_3_ resonance appears as a doublet around 3 ppm in the ^1^H NMR spectrum with a distinct coupling constant ^3^*J*_HH_ ∼5 Hz due to the adjacent NH unit, commensurate with similar systems developed by Ruiz and Johnson.^27,29^ A 2D NMR spectrum further supported the formation of the acyclic carbene ligand, specifically through the HMBC correlation between the N–CH_3_ resonance around 3 ppm and the carbenic carbon, as well as ^1^H–^1^H COSY correlations between the N–CH_3_ group and the N–H signal in all three complexes. In Ru-2a, only one of the two N–H resonances correlates with the N–CH_3_ group, indicating bonding of the second pyrrolidine to Ru rather than to the carbene scaffold. The carbonate complex Ru-2b features a diagnostic ^13^C resonance at 167 ppm for the *κ*^2^ -bound CO_3_^2−^ ligand. (Fig. S10).

**Fig. 2:**
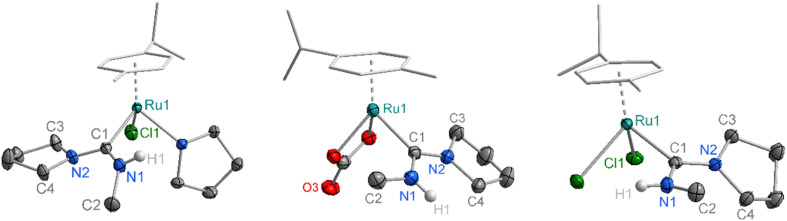
Crystallographically determined molecular structure of Ru-2a (left), Ru-2b (middle) and Ru-2c (right; all thermal ellipsoids at 50% probability for Ru-2a and Ru-2c, and at 30% for Ru-2c; all hydrogen atoms except H1 omitted for clarity; CCDC 2487351–2487353).

**Fig. 3:**
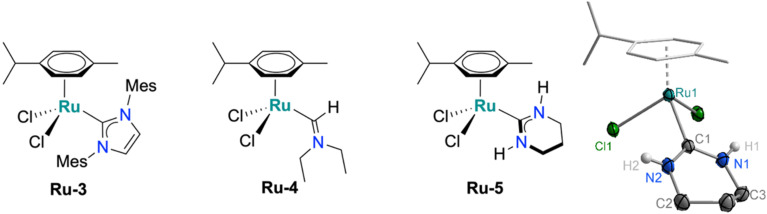
Schematic representation of reference complexes Ru-3, Ru-4 and the newly synthesized Ru-5 with its molecular structure from X-ray diffraction analysis (50% thermal ellipsoids).

Single crystals of complexes Ru-2a–c suitable for X-ray diffraction were grown by slow diffusion of Et_2_O into CH_2_Cl_2_ solutions of the corresponding complex. The molecular structures confirm the expected three-legged piano-stool geometry at the Ru centre (Fig. 2). The Ru–C bond lengths of 2.072(11) Å (Ru-2a), 2.08(2) Å (Ru-2b) and 2.074(17) Å (Ru-2c) are slightly elongated compared to previously reported metal–carbene bonds in acyclic aminocarbene ruthenium complexes (*e.g.*Ru-4, Fig. 3 and Table 1),^16,17^ but still shorter than analogous Ru complexes bearing NHC ligands such as IMes in Ru-3.^31^ Moreover, the shortened C–N bond indicates increased double-bond character between the carbenic carbon and the pyrrolidine nitrogen, resulting in ylide-rather than carbene-type bonding to the metal center. Additionally, the sum of the bond angles around N2 in all three complexes is 357–360°, consistent with a planar geometry and indicative of sp^2^ hybridization at the pyrrolidine nitrogen. In both Ru-2a and Ru-2c, the NMeH hydrogens are oriented toward the Ru centre, likely stabilized by hydrogen bonding interactions with the chloride ligands. In contrast, the hydrogen in the carbonate complex Ru-2b is oriented away from the metal, consistent with the absence of H-bond interactions with the carbonate ligand. While these different orientations indicate rotational flexibility about the C–N–Me angle, the lower field NH resonances of Ru-2a and Ru-2c at *δ*_H_ = 8.43 and 7.24 ppm, respectively, compared to *δ*_H_ = 6.69 ppm for Ru-2b suggests retention of this hydrogen bonding arrangement also in solution (RT, CD_2_Cl_2_). Furthermore, the N–H chemical shift is solvent dependent which is in agreement with the rotational flexibility of the ADC ligand.^32^ Structural flexibility is further supported by the coalescence of the pyrrolidine resonances in Ru-2c observed through variable-temperature ^1^H NMR experiments (Fig. S19). The coalescence temperature of 40 °C translates to a rotational barrier Δ*G*^‡^ = 14.9 kcal mol^−1^, in good agreement with previously reported literature values.^11^ The steric properties of the Ru-coordinated ADC ligand were assessed by calculating the buried volume (% *V*_bur_) using the SambVca 2.1 software.^33^ The steric map (Fig. S103) indicates a low % *V*_bur_ of 23.3% for the ADC ligand, reflecting minimal steric space requirements for this ligand. While the previously reported mono-amino carbene (MAC) ligand exhibits an even lower % *V*_bur_ of 21%),^17^ the ADC system is considerably less bulky than typical NHC ligands such as IMes with a % *V*_bur_ of 28%.^31^

**Table 1:** Selected crystallographic parameters for complexes Ru-2a–c and reference complexes Ru-3–5

Complex	**Ru-2a**	**Ru-2b**	**Ru-2c**	**Ru-3** a	**Ru-4** b	**Ru-5**
Ru1–C1/Å	2.0724(11)	2.080(2)	2.0735(17)	2.142(4)	2.0084(19)	2.052(2)
C1–N1/Å	1.3451(15)	1.337(3)	1.347(2)	1.375(5)	1.286(3)	1.330(4)
C1–N2/Å	1.3374(14)	1.332(3)	1.328(2)	1.370(5)	—	1.333(3)
Ru1⋯H1/Å	2.861	3.796	2.846	—	—	2.967
N1–C1–N2/°	117.03(10)	115.3(2)	117.44(15)	102.0(3)	—	117.4(2)
Σ∠N1/° c	355	360	357	360	360	360
Σ∠N2/° c	360	360	357	360	—	359
% *V*_bur_^d^	23.2	26.8	23.3	28.0	21.0	20.0

a Data from ref. 31.

b Data from ref. 17.

c Sum of angles with vertex N1 and N2, respectively.

d Buried volume calculated with SambVca 2.1 online software (ref. 33).

Another useful comparison is the cyclic 6-membered diamino carbene Ru-5 (Fig. 3), since the basicity of this carbene is similar to that of acyclic diaminocarbenes.^34^ Complex Ru-5 was prepared by a similar route starting from an isocyanide containing a silyl-protected aminopropyl isocyanide and [RuCl_2_(cym)]_2_. After amine deprotection, cyclization takes place and affords complex Ru-5, which was characterized by NMR, MS, and elemental analysis as well as X-ray diffraction.

**Scheme 2:**
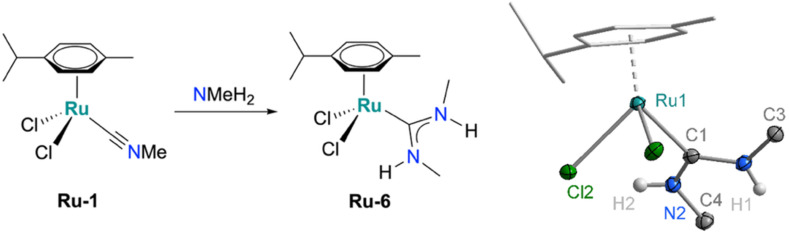
Synthesis of Ru-6 from Ru-1 and methylamine and crystallographically determined molecular structure (30% probability ellipsoids, carbon-bound hydrogens omitted for clarity, CCDC 2487350).

This synthetic strategy towards ADC complexes is not limited to pyrrolidine and was successfully extended to other amines. For example, addition of MeNH_2_ to the isonitrile ruthenium precursor Ru-1 followed by carbonate and HCl treatment afforded Ru-6 in high crystalline yield (58%, Scheme 2). The formation of the complex was indicated by the characteristic doublets at 3.28 ppm (*J* = 4.9 Hz) and 2.89 ppm (*J* = 5.0 Hz) in the ^1^H NMR spectrum, suggesting diastereotopic NHMe groups at the carbene. Further support for ADC formation was provided by the diagnostic resonance at 205 ppm for the carbenic carbon in ^13^C{^1^H} NMR spectroscopy. X-ray diffraction analysis confirmed the structure, revealing a piano-stool geometry analogous to the pyrrolidine-derived complexes and two distinct N–Me groups in a proximal and distal orientation, respectively (Scheme 2), presumably for minimizing steric congestion of the two methyl groups. Notably variable temperature NMR experiments indicate considerable line broadening of the NHMe resonances upon heating to 80 °C, suggesting rotational flexibility about the carbene–nitrogen bonds (Fig. S30). Such dynamics are impossible in NHCs such as Ru-3 or Ru-5. In polar solvents, the N–H resonances appear in closer proximity, which was attributed to less pronounced H⋯Cl hydrogen bond interactions (Fig. S31). In protic solvents such as EtOD or MeOD, complete N–H/D exchange was observed both for Ru-6 and Ru-2c (Fig. S32 and S18) indicating reactivity of this site.

Transfer hydrogenation (TH) of ketones is a well-established methodology for C=O bond reduction, and Ru(ii)–arene complexes are excellent catalysts for this reaction.^35,36^ However, most of the reported protocols rely on basic additives to achieve catalytic activity.^37^ This dependence on strong bases introduces significant limitations, including reactor corrosion,^37^ and, most important for a broad scope, incompatibility with base-sensitive substrates. Recent studies have demonstrated base-free TH of ketones in 2-propanol.^37–39^ However, such systems typically rely on synthetically challenging metal–hydride complexes or require additives to generate the active hydride species *in situ*.^39^

**Fig. 4:**
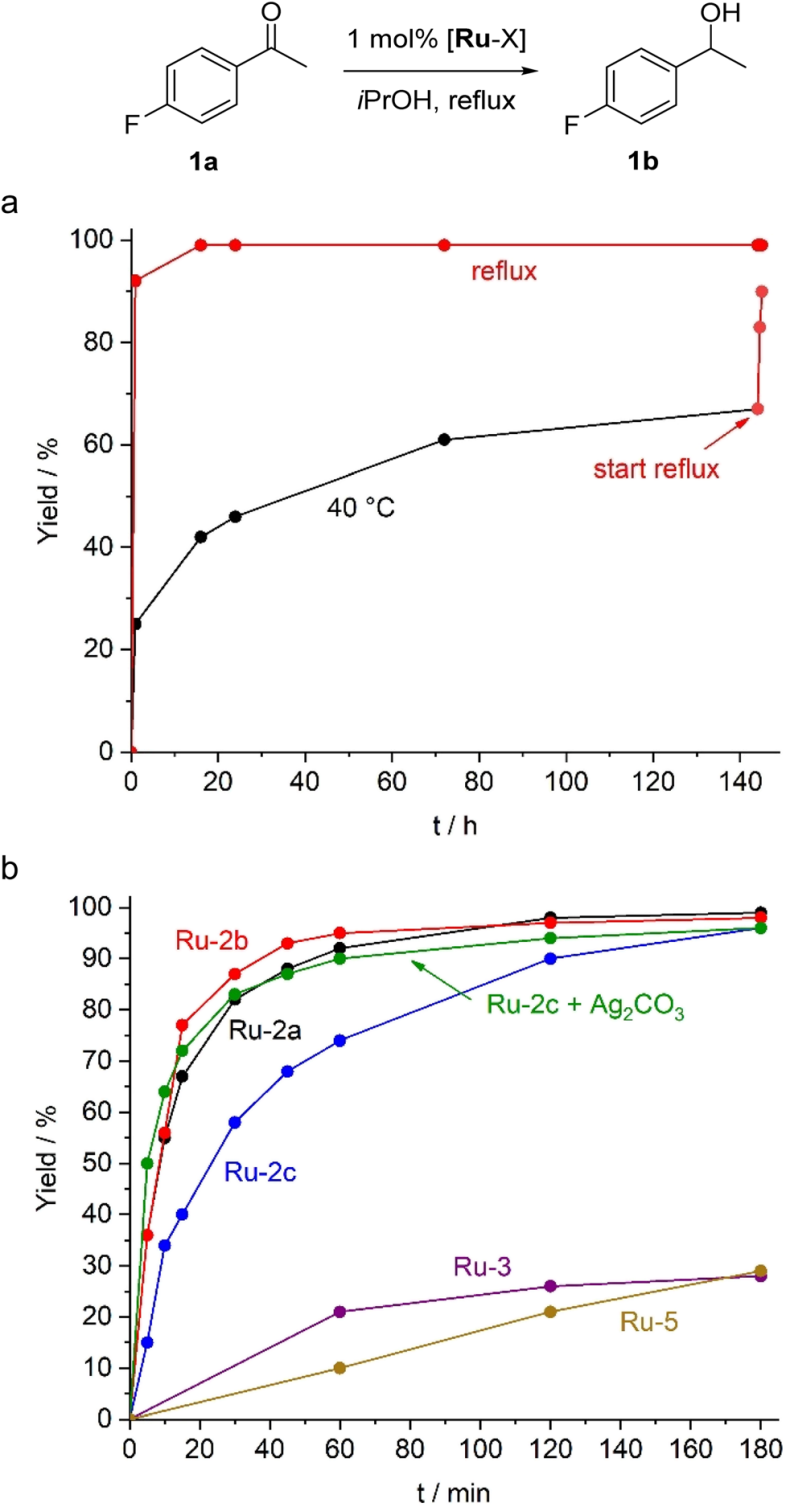
(a) Time-conversion profile of the TH of 4-fluoracetophenone catalyzed by Ru-2a at reflux (red dots) and 40 °C (black dots), with subsequent heating to reflux after 140 h (lines for guiding the eye only); Reaction conditions: substrate (0.29 mmol), catalyst (2.9 µmol, 1.0 mol%), degassed *i*PrOH (2.0 mL); (b) Time-conversion profile of the transfer hydrogenation (TH) of 4-fluoracetophenone (1a) catalyzed by Ru-2a, Ru-2b, Ru-2c, Ru-2c + Ag_2_CO_3_, Ru-3 and Ru-5 under conditions described in (a), reflux temperature.

The new Ru–ADC complexes feature a potential hydrogen source proximal to the metal centre, useful for mimicking base-assisted proton transfer. Indeed, complexes Ru-2 rapidly undergo H/D exchange at the NH group when dissolved in deuterated protic solvents such as CD_3_OD (Fig. S18).^40^ We therefore explored their activity in the base-free transfer hydrogenation using 4-fluoroacetophenone as a model substrate. With 1 mol% Ru-2a in 2-propanol, both as solvent and hydrogen source, full hydrogenation to 4-fluorophenyl-1-ethanol was achieved within 1 hour under reflux (Fig. 4a). Maximum turnover numbers of 750 and 1200 were obtained with 0.1 and 0.05 mol% catalyst loading, respectively, with maximum turnover frequencies, TOF_max_ = 820 h^−1^. These activites are some two orders of magnitude lower than the most active base-assisted Ru TH catalysts.^41,42^ The ADC system is catalytically active even at 40 °C, though turnover is slow, and conversions reached 65% only after 140 h. Subsequent heating to 85 °C led to essentially full conversion (Fig. 4a), indicating a high robustness of the catalytic system over several days and the potential to switch transfer hydrogenation on and off by temperature control.

The ancillary ligand on the ADC-Ru fragment played a significant role. Initial rates measured after 10 min revealed comparable TOFs for Ru-2a (330 h^−1^) and Ru-2b (340 h^−1^), whereas dichloride complex Ru-2c displayed a lower TOF of 200 h^−1^. This difference highlights the importance of the basicity of the ancillary ligand in facilitating catalyst activation, presumably as a stoichiometric internal base. Such a model is further supported by catalytic runs with Ru-2c in the presence of 1.5 mol% Ag_2_CO_3_. This protocol doubled the TOF to 390 h^−1^ and gave activities that are essentially identical to those of the carbonate complex Ru-2b. Furthermore, when the reaction was performed with the previously reported IMes complex Ru-3 or the protic cyclic analogue of Ru-2, namely Ru-5 (*cf*. Fig. 3), conversions to the corresponding alcohol were low and did not exceed 30% under base-free conditions, even after prolonged reaction times. This lower performance underscores the relevance of the flexible acyclic structure of the carbene ligand in combination with the proximal hydrogen source.

**Scheme 3:**
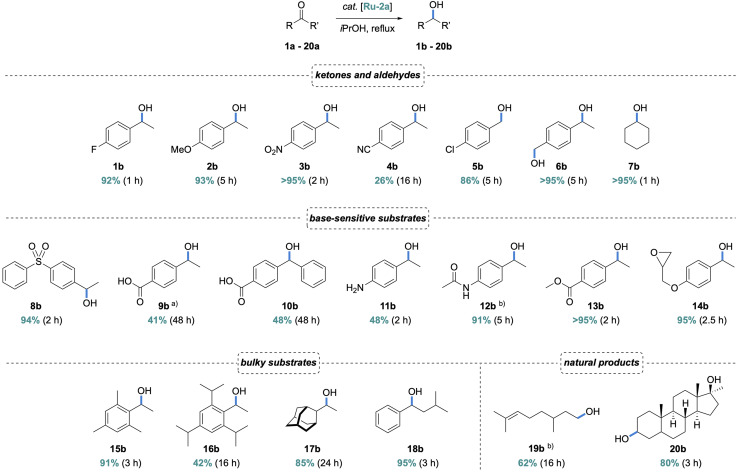
Scope of the transfer hydrogenation reaction with Ru-2a, general reaction conditions: substrate (0.29 mmol), Ru-2a (2.9 µmol, 1.0 mol%), 1,3,5-trimethoxybenzene (internal standard), degassed *i*PrOH (2.0 mL), reflux; yields determined by ^1^H NMR spectroscopy (see also SI); (a) yield increased to 56% conversion (*t* = 48 h) with Ru-2b; (b) conversion instead of yield; (c) using Ru-4 instead of Ru-2a.

To evaluate the scope of the ADC-Ru system, a range of aryl and aliphatic ketones were transfer hydrogenated under base-free conditions using 1 mol% Ru-2a. Electron-donating and -withdrawing aryl substituents in ketones 1a–3a were well tolerated, affording the corresponding alcohols 1b–3b in high yields and short reaction times (Scheme 3). In contrast, the nitrile-substituted ketone 4a showed significantly reduced reactivity (26%, 16 h), likely due to imine formation and subsequent coordination to the Ru center, thus deactivating the catalytic site. Aliphatic ketones and aldehydes 5a–7a were also reduced efficiently which resulted in high yields of alcohols 5b–7b. Notably, in the bifunctional substrate 6a, the aldehyde is reduced faster than the ketone, though not selectively (94% aldehyde reduction *vs.* 84% ketone reduction after 1 h, Fig. S52), consistent with the higher reactivity of aldehydes in transfer hydrogenation.^36^ In contrast to base-assisted TH protocols, the Ru–ADC system tolerates a broad range of base-sensitive functional groups, including sulfones (8a, 94%), acids (9a, 41%; 10a, 48%), amines (11a, 48%), amides (12a, 91%), and esters (13a, >95%), without any detectable (trans)esterification or hydrolysis, and also epoxides (14a, 95%). Of note, acidic substrates such as 9a and 10a are known to neutralize base additives and suppresses catalyst activation completely, while the Ru–ADC complexes reach appreciable conversion.

Due to the very low steric demand of the ADC ligand, we probed the competence of Ru-2a in the TH of sterically demanding ketones.^17^ Remarkably, 15a was reduced very efficiently, affording nearly quantitative yield (91%) after 3 h. This performance is even better than that of the mono-aminocarbene complex Ru-4 (*cf.* 67% after 24 h). Substrate 16a was converted less effectively (42% in 16 h, *cf.* 88% with Ru-4), though the MAC system required basic conditions and a higher 5 mol% catalyst loading. In contrast, the adamantyl ketone 17a was transfer hydrogenated with the ADC system with the same efficiency as the MAC analogue (85% conversion in 24 h), though with the advantage of base-free conditions. Notably, the tolerance of bulky ketones is not restricted to one side of the carbonyl group, as demonstrated by the efficient transfer hydrogenation of (*iso*-butyl)(phenyl)ketone 18a (95% in 3 h). The mild reaction conditions also allow for the selective reduction of more complex substrates such as citronellal 19a, and for late-stage derivatization as exemplified using mestanolone 20a. After 5 h, the corresponding mestanediol 20b was obtained in 80% isolated yield and was structurally characterized by ^1^H, ^13^C NMR, and EI-MS analysis.

In addition to the beneficial flexibility of ADCs, also the presence of a NH functionality proximal to the substrate bonding site is conceived to be relevant for imparting high catalytic activity to complexes Ru-2. In support of this hypothesis, transfer hydrogenation of bulky ketone substrates 15a, 16a, and 18a were run with Ru-4, which features similar flexibility but lacks the proximal NH functionality. Complex Ru-4 was considerably less effective and reached only about 20% conversion under base-free conditions, compared to >90% yield of 15b and 18b when the ADC complex Ru-2a was used as catalyst precursor (Scheme 3), strongly suggesting cooperative metal/NH effects.^43–45^

## Conclusions

This work showcases structural flexibility paired with a proximal protic functionality as a beneficial concept of ligand design in homogeneous catalysis. Specifically, acyclic diamino carbenes (ADCs) as a versatile family of ligands and new for ruthenium impart attractive catalytic activity in base-free transfer hydrogenation, which allows for the hydrogenation of substrates with base-sensitive groups. The much superior activity induced by acyclic ligands compared to cyclic analogues is attributed to a flexible catalytic pocket that synergistically assists turnover of substrates on the catalytic cycle. Moreover, the mild conditions enable late-stage functionalization of industrially and pharmaceutically relevant substrates. The synthesis of the complexes involves carbene formation at the ruthenium center. This approach is straightforward and provides direct access towards ligand diversification with obvious opportunities for tailoring activity and selectivity of the catalytically active species also for different applications. A key benefit of these ligands is their structural flexibility, a direct consequence of the acyclic scaffold of these diamino carbenes. Moreover, the proximal location of a cleavable NH hydrogen is presumed to promote cooperative effects with the metal center, which adds another unique feature to ADC ligands.

## Conflicts of interest

The authors declare no competing financial interest.

## Supplementary Material

SC-017-D5SC08206D-s001

SC-017-D5SC08206D-s002

## Data Availability

The supplementary information (SI) accompanying this submission contains all relevant data. Supplementary information: synthetic and catalytic procedures, analytical data for all new compounds, time-conversion profiles, buried volume data, and crystallographic details. See DOI: https://doi.org/10.1039/d5sc08206d. CCDC 2487350–2487355 contain the supplementary crystallographic data for this paper.^46*a*–*f*^
