# Supplementary material for: Ligand flexibility as a concept to unlock catalytic activity: acyclic carbenes for base-free transfer ruthenium hydrogenation catalysis
Source: Chem Sci. 2025 Dec 31;17(8):4325–31. doi: 10.1039/d5sc08206d (PMC12794291; doi:10.1039/d5sc08206d)
Supplement: SC-017-D5SC08206D-s001 [file SC-017-D5SC08206D-s001.pdf]

Supplementary Information (SI) for Chemical Science.
This journal is © The Royal Society of Chemistry 2025

Supporting Information

belonging to

Ligand flexibility as a Concept to Unlock Catalytic Activity: Acyclic Carbenes for Base-Free Transfer Ruthenium Hydrogenation Catalysis

Gianluca Righetti,[a] Georgyi Koidan,[b] Sergiy L. Filimonchuk,[b] Svitlana Shishkina,[c] Aleksandr Kostyuk[b,*] and Martin Albrecht[a,*]

[a] *Department of Chemistry, Biochemistry, and Pharmaceutical Sciences, University of Bern, Freiestrasse 3, 3012 Bern, Switzerland. Email: martin.albrecht@unibe.ch*

[b] *Department of Organophosphorus Chemistry, Institute of Organic Chemistry, Academician Kukhar str. 5, 02094, Kyiv-94, Ukraine. Email: a.kostyuk@yahoo.com*

[c] *SSI Institute of Single Crystals, NAS of Ukraine, 60 Nauky ave., 61001 Kharkiv, Ukraine.*

Email martin.albrecht@unibe.ch

Table of Contents

1. Synthesis of new Complexes S2
2. Catalytic Experiments – General Procedure S42
3. Time Conversion Profile – Different Catalysts S92
4. Temperature-dependent Experiments S99
5. Variation of Catalyst Loading S100
6. Calculation of Relative Buried Volume (%V_{bur}) S102
7. Thermal Elemental Analysis S104
8. Crystallographic Details S107
9. References S111

Synthesis of new Complexes

General Considerations

All reagents and solvents were commercially available and used as received unless specified otherwise. THF and CH_2Cl_2 were dried by diffusion through solvent purification columns (SPS) under argon atmosphere. 2-propanol (Sigma-Aldrich, HPLC grade 99.9% purity) was degassed under nitrogen atmosphere before use. **Ru-4** was synthesized according to literature procedure.[S1] Catalytic runs were carried out under an inert nitrogen atmosphere using standard Schlenk techniques. NMR spectra were measured at 25 °C on a Bruker spectrometer operating at 300, 400, or 500 MHz (^{1}H NMR) and 75, 101, or 126 MHz ($^{13}C\{^{1}H\}$ NMR), respectively. Chemical shifts (δ in ppm, coupling constants *J* in Hz) were referenced to residual solvent resonances downfield to $SiMe_4$. Elemental analyses were performed by the Microanalytic Laboratory of the Department of Chemistry, Biochemistry and Pharmacy (DCBP) of University of Bern, using a ThermoScientific Flash 2000 CHNS-O elemental analyzer. High-resolution mass spectrometry measurements were carried out using a ThermoScientific LTQ Orbitrap XL (ESI-TOF).

Synthesis of Ru-1

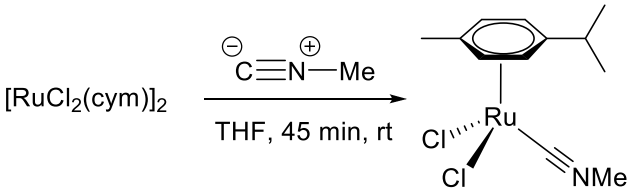

Ru-1

$[RuCl_2(cym)]_2$ (500 mg, 0.8 mmol) was added to a Schlenk flask, followed by the addition of THF (17 mL). Subsequently, methylisocyanide (0.22 mL, 4.05 mmol, 5.1 eqv.) was added and the reaction mixture was stirred for 45 minutes at room temperature. The crude product was filtered, washed with Et_2O (3 x 15 mL) and dried *in vacuo* to obtain the final product **Ru-1** as a red powder in a yield of 568 mg (98%).

^{1}H NMR (400 MHz, $CDCl_3$) δ 5.57 (d, J = 6.2 Hz, 2H, C_{cym}H), 5.39 (d, J = 6.2 Hz, 2H, C_{cym}H), 3.63 (s, 3H, N-CH_3), 2.82 (septet, J = 6.9 Hz, 1H, Cym-$CHMe_2$), 2.23 (s, 3H, Cym-CH_3), 1.28 (d, J = 6.9 Hz, 6H, Cym-CH(CH_3)$_2$).

^{13}C{^{1}H} NMR (101 MHz, CD_2Cl_2) δ 139.7 (Ru-C), 107.7 (C_{cym}), 106.9 (C_{cym}), 88.1 (C_{cym}H), 87.9 (C_{cym}H), 31.6 (Cym-$CHMe_2$), 22.5 (Cym-CH(CH$_3$)$_2$), 18.9 (Cym-CH_3).

Elemental analysis calcd. (%) for $C_{12}H_{17}Cl_2NRu$: C, 41.51; H, 4.93; N, 4.03. Found: C, 41.60; H, 4.92; N, 3.91.

HR-MS calcd. for $C_{12}H_{17}NClRu$ $[M–Cl]^+$ 312.0088. Found: 312.0085.

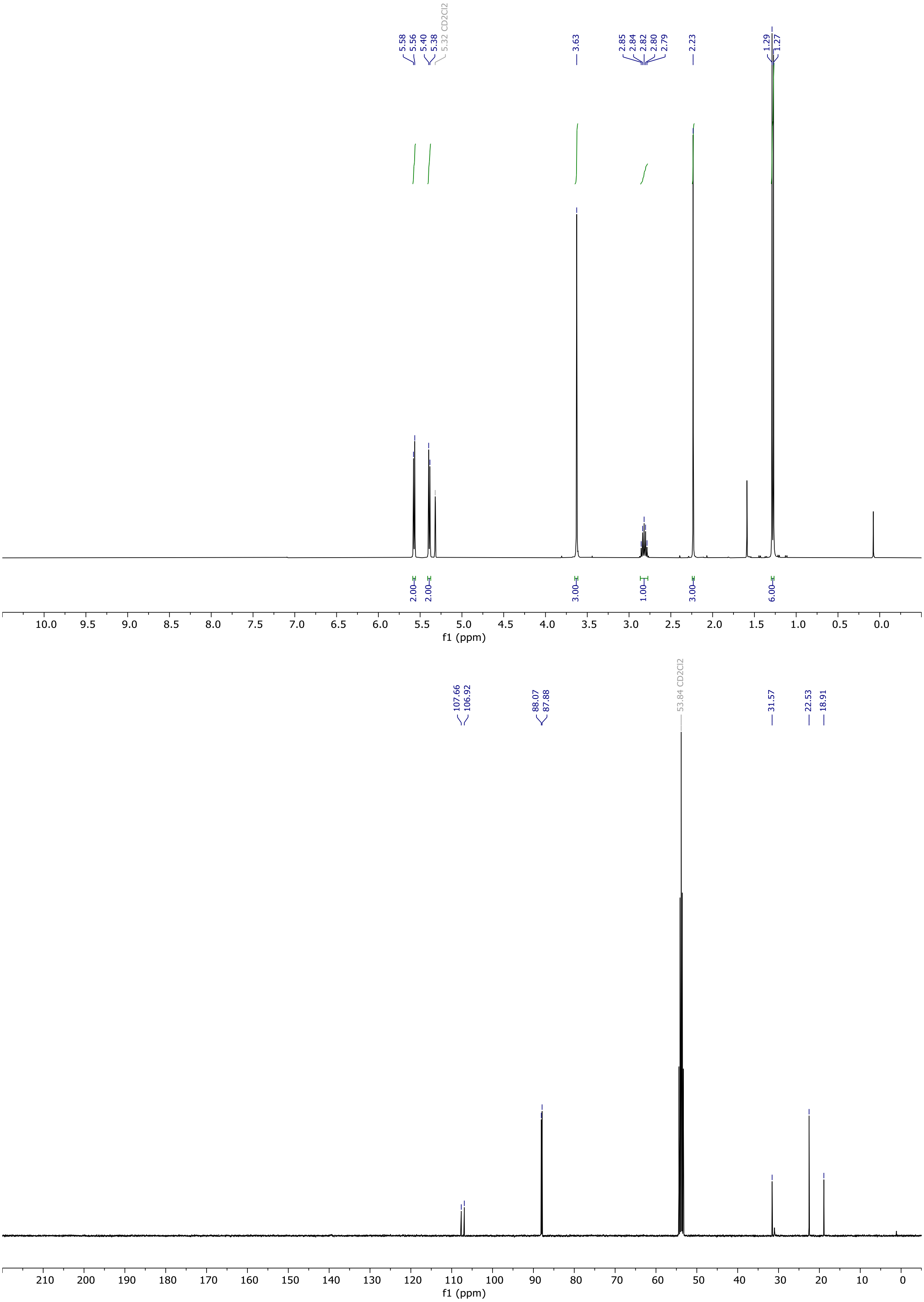

Figure S1:^{1}H (top) and ^{13}C{^{1}H} NMR (bottom) spectrum (CD_2Cl_2, 298 K, 400 MHz) of **Ru-1**.

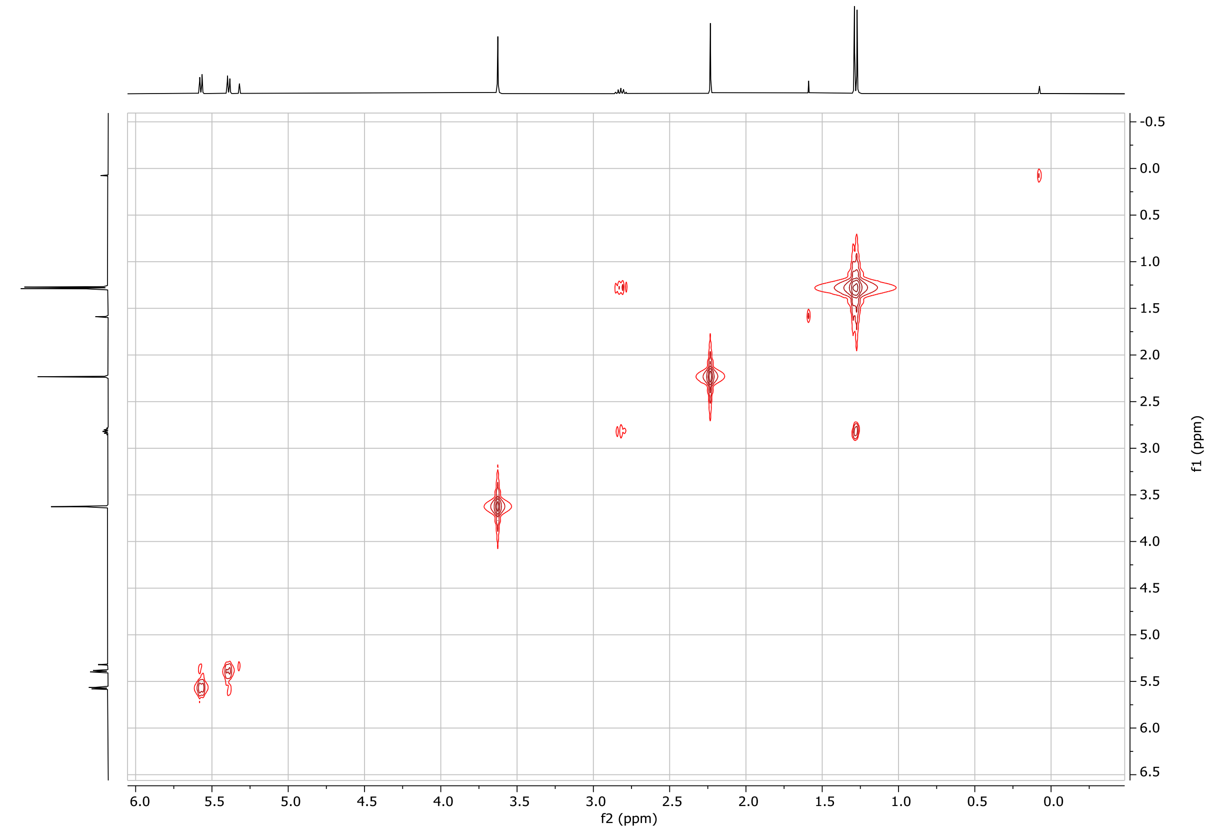

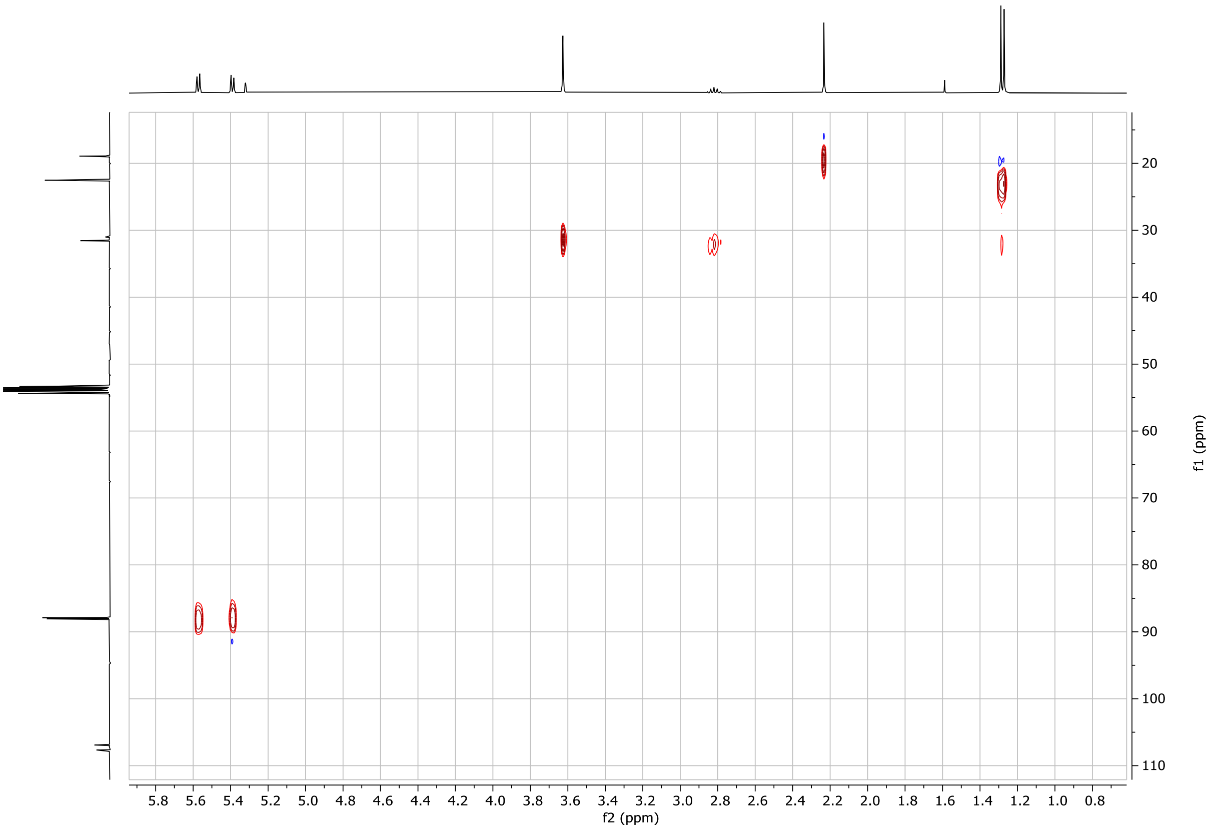

Figure S2:^{1}H-^{1}H-COSY (top) and ^{1}H-^{13}C-HSQC (bottom) spectrum (CD_2Cl_2, 298 K, 400 MHz) of **Ru-1**.

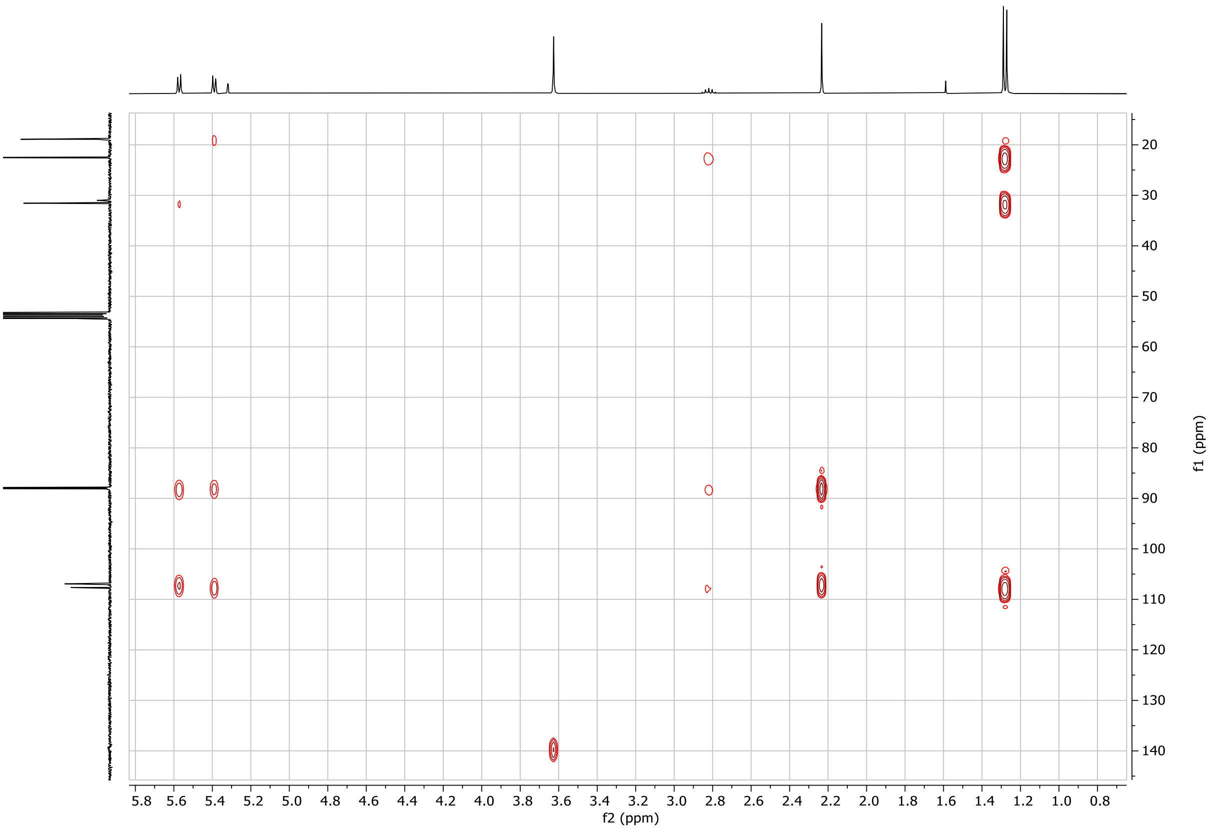

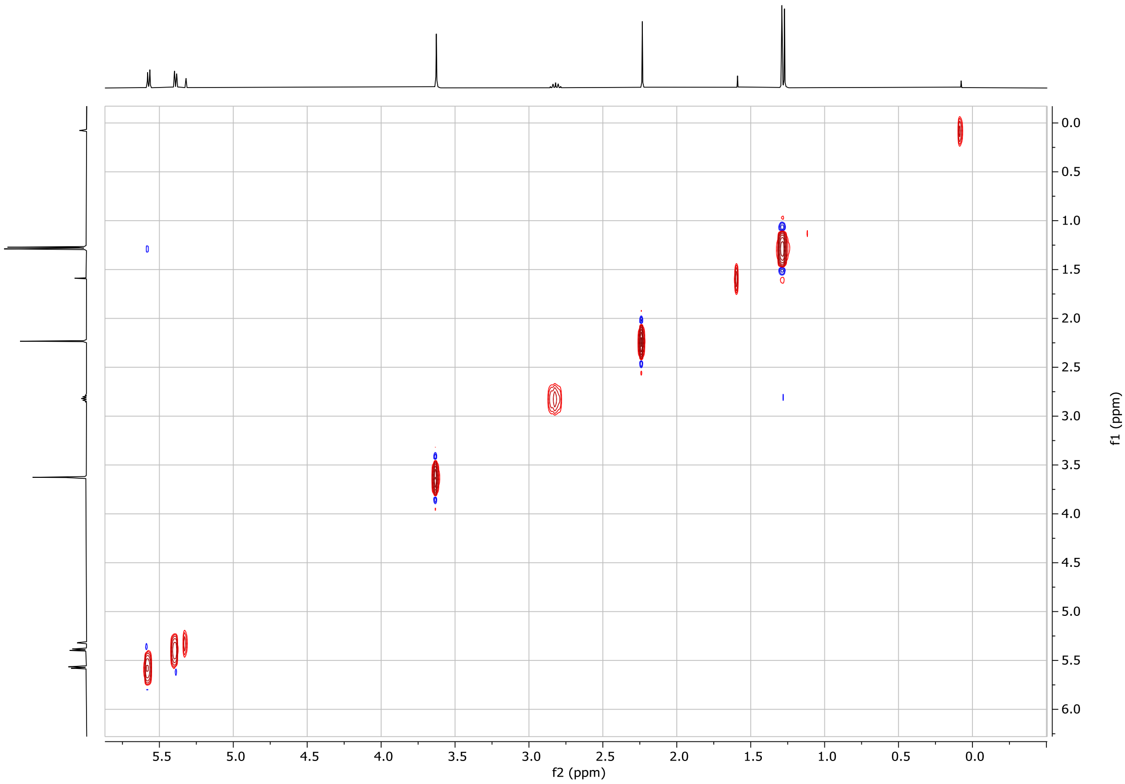

Figure S3: ^{1}H-^{13}C-HMBC (top) and ^{1}H-^{1}H-NOESY (bottom) spectrum (CD_2Cl_2, 298 K, 400 MHz) of **Ru-1**.

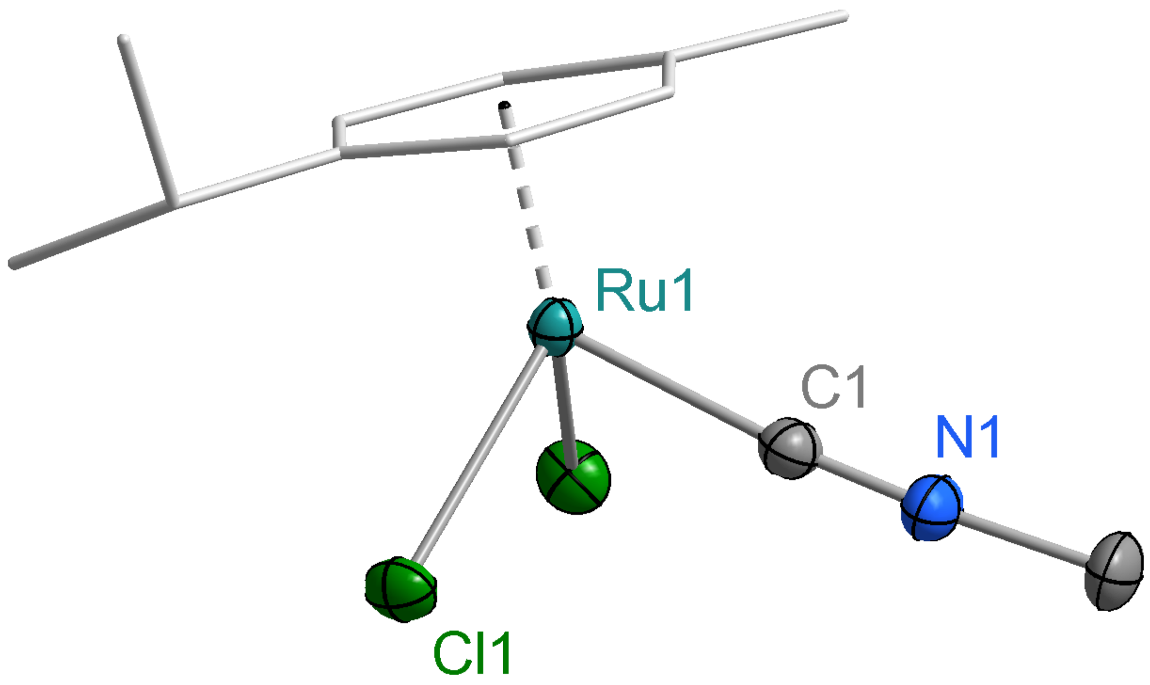

Figure S4: Molecular structure of **Ru-1** with 30% probability displacement ellipsoids. All H atoms have been omitted for clarity.

Synthesis of Ru-2a

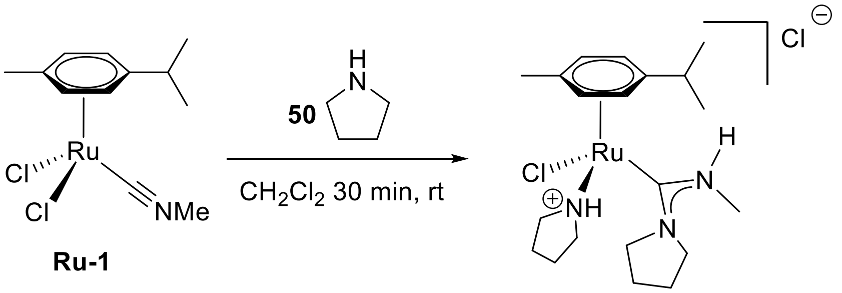

Ru-1 (200 mg, 0.55 mmol) was dissolved in CH_2Cl_2 (7 mL) in a microwave vial, affording a red solution. Pyrrolidine (2.3 mL, 27.5 mmol, 50 eqv) was added, resulting in an immediate colour change to yellow. The reaction mixture was stirred at room temperature for 30 min and subsequently quenched with HCl (1 M, 10 mL). The crude mixture was extracted with CH_2Cl_2 (2 × 15 mL), and the combined organic phases were further washed with HCl (1 M, 3 × 10 mL). The organic layer was dried over Na_2SO_4, filtered, and concentrated under reduced pressure. The resulting red solid was washed with pentane (3 × 10 mL) and purified by slow diffusion of Et_2O into a CH_2Cl_2 solution at room temperature, affording red crystals of **Ru-2a** (148 mg, 55%).

^{1}H NMR (400 MHz, CD_2Cl_2) δ 8.43 (br s, 1H, MeNH), 7.31 (br s, 1H, H_{pyr}), 5.57, 5.52, 5.15, 5.06 (4 × d, J = 6.0 Hz, 1H, H_{cym}), 4.51 (br s, 1H, H_{pyr}), 3.72 (br s, 1H, H_{pyr}), 3.63–3.42 (m, 3H, H_{pyr}), 3.27–3.14 (m, 1H, H_{pyr}), 3.07 (d, J = 5.1 Hz, 3H, HNCH_3), 2.92 (septet, J = 7.0 Hz, 1H, cym–HMe_2), 2.68–2.57 (m, 1H, H_{pyr}), 2.50–2.40 (m, 1H, H_{pyr}), 2.19 (s, 3H, cym-CH_3), 1.89 (s, 4H, H_{pyr}), 1.79–1.65 (m, 3H, H_{pyr}), 1.56–1.46 (m, 1H, H_{pyr}), 1.25, 1.21 (2 × d, 7.0 Hz, 3H, CH-CH_3).

^{13}C{^{1}H} NMR (101 MHz, CD_2Cl_2) δ 209.2 (NCN), 116.1, 97.3 (2 × C_{cym}), 88.1 (C_{cym}-H), 86.8 (C_{cym}-H), 81.1 (2 × C_{cym}-H), 58.2, 53.8, 50.8 (3 × C_{pyr}), 36.2 (N-CH_3), 30.7 (cym-$CHMe_2$), 27.8, 25.7 (2 × C_{pyr}), 24.1, 20.1 (2 × cym-CHCH_3), 18.5 (cym-CH_3).

Elemental analysis calcd. (%) for $C_{20}H_{35}Cl_2N_3Ru$: C, 49.08; H, 7.21; N, 8.58. Found: C, 49.21; H, 7.27; N, 8.50.

HR-MS calcd. for $C_{20}H_{36}N_3Cl_2Ru$ $[M+H]^+$ 490.1324. Found: 490.1319.

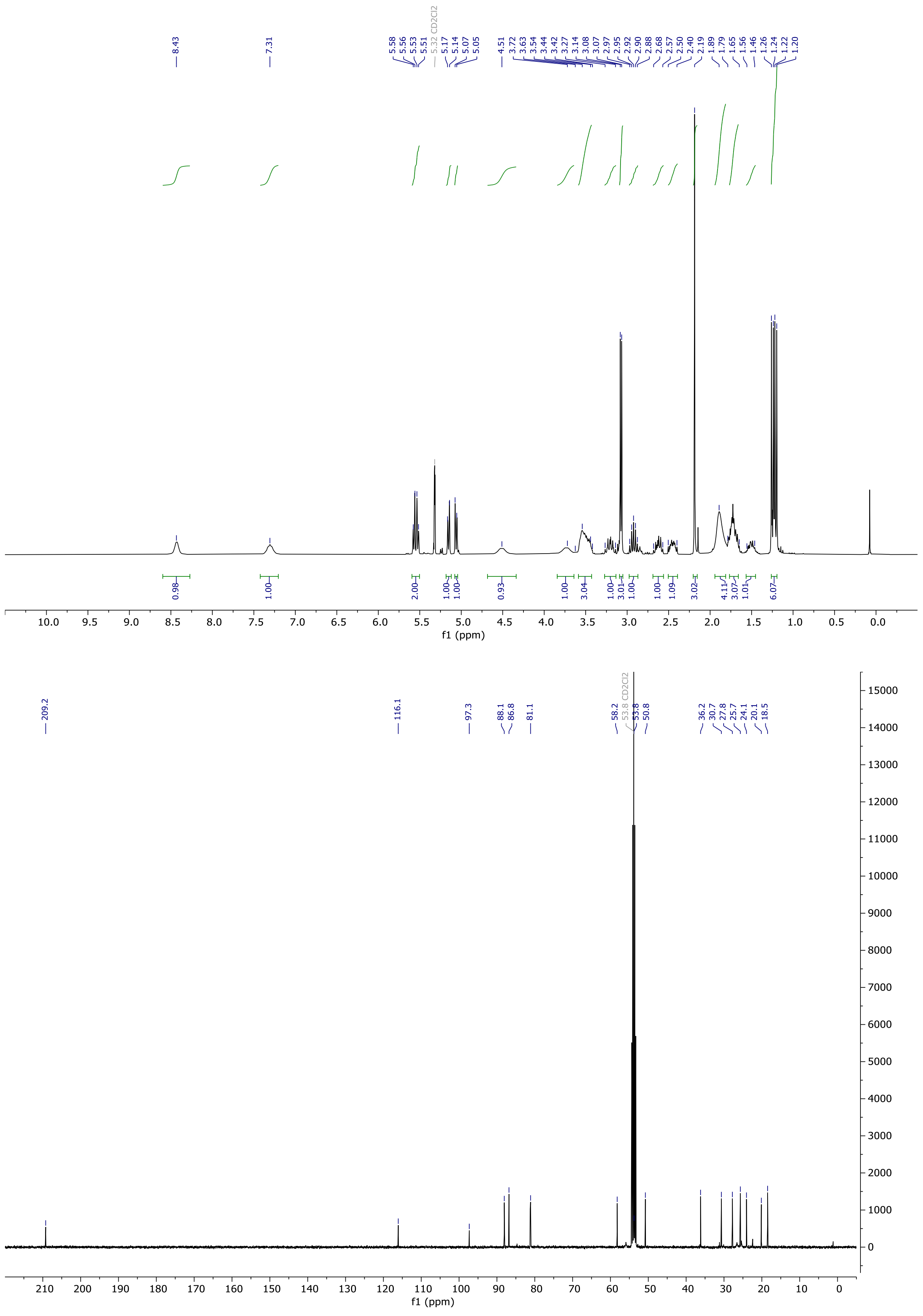

Figure S5:^{1}H (top) and ^{13}C{^{1}H} NMR (bottom) spectrum (CD_2Cl_2, 298 K, 400 MHz) of **Ru-2a**.

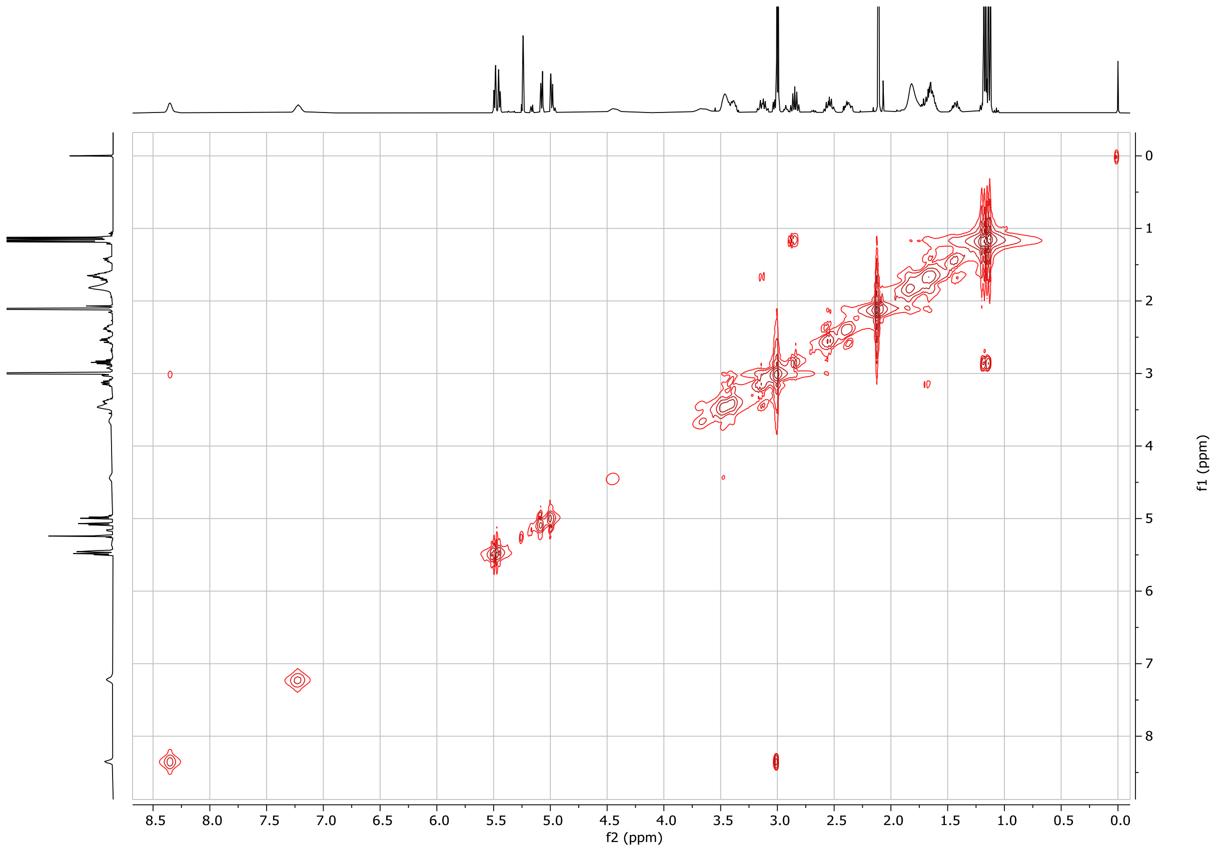

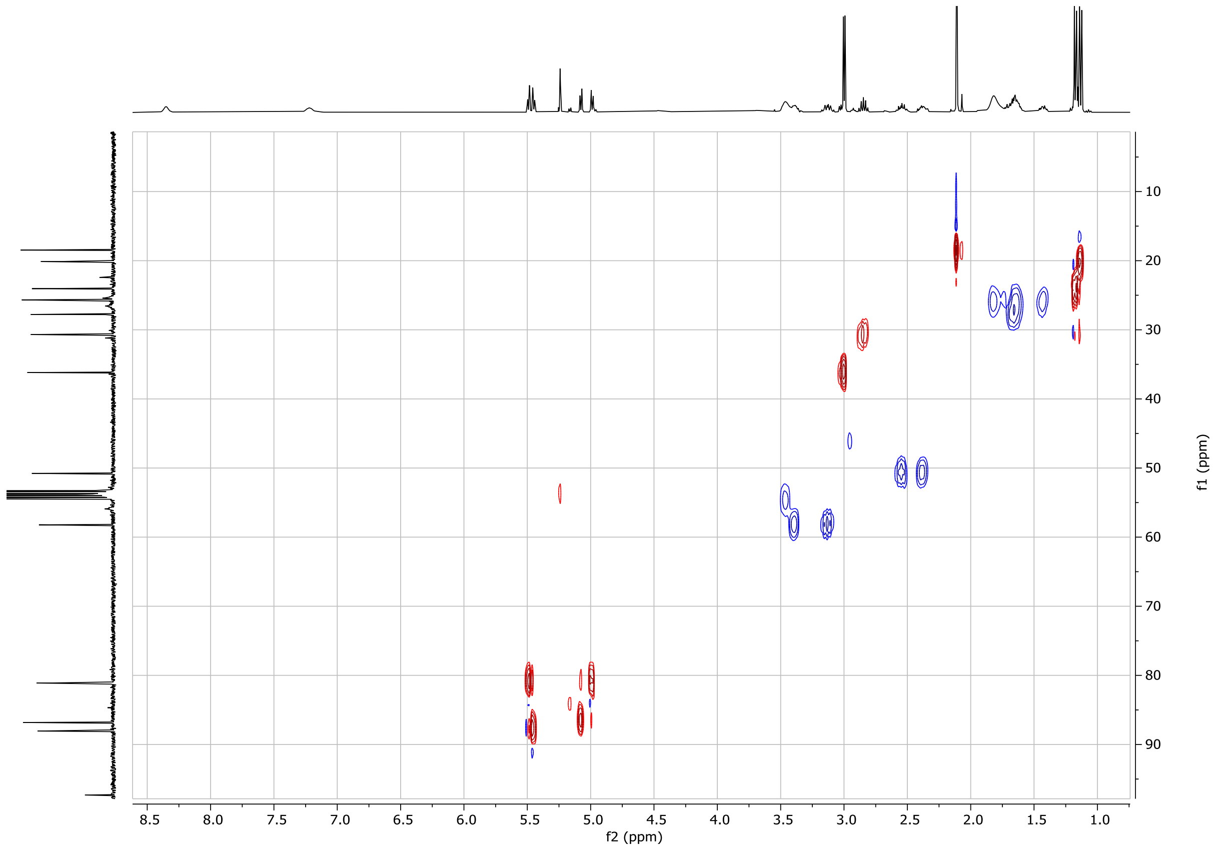

Figure S6:^{1}H-^{1}H-COSY (top) and ^{1}H-^{13}C-HSQC (bottom) spectrum (CD_2Cl_2, 298 K, 400 MHz) of **Ru-2a**.

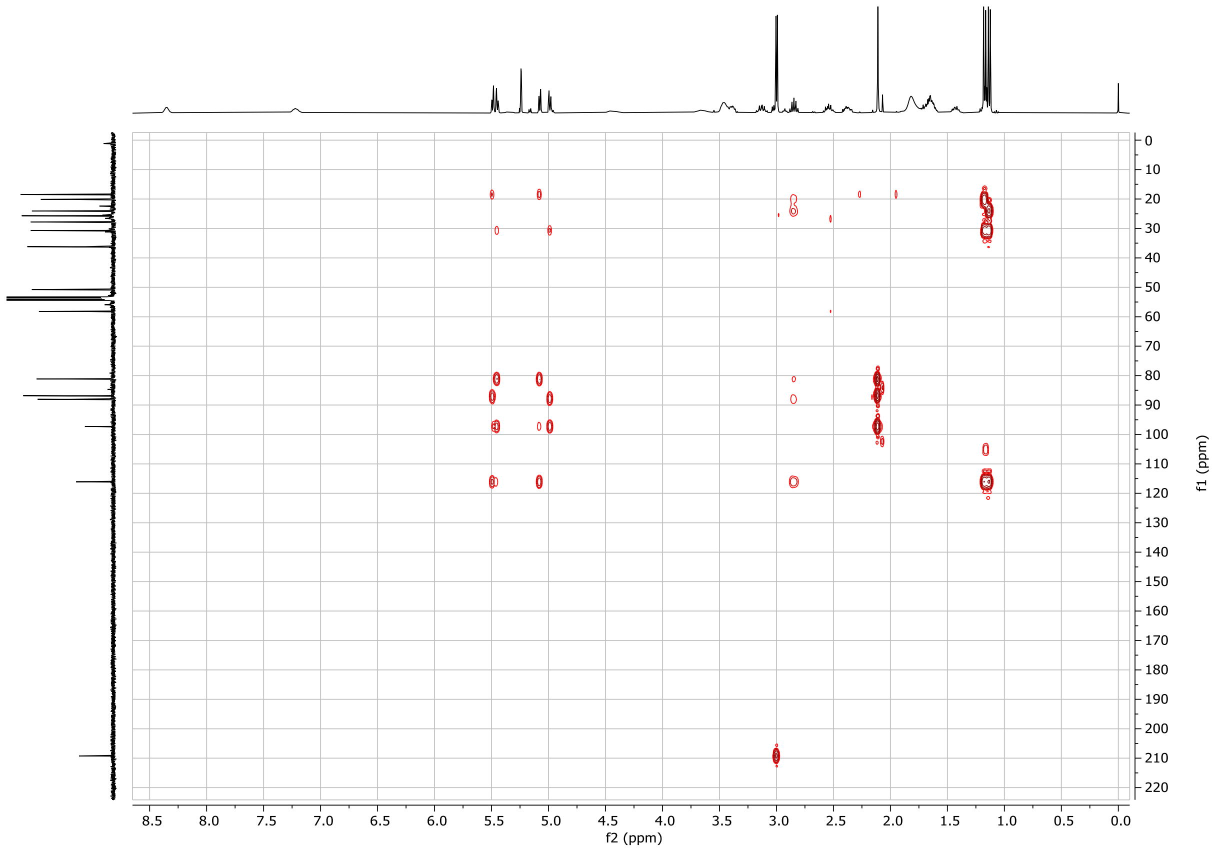

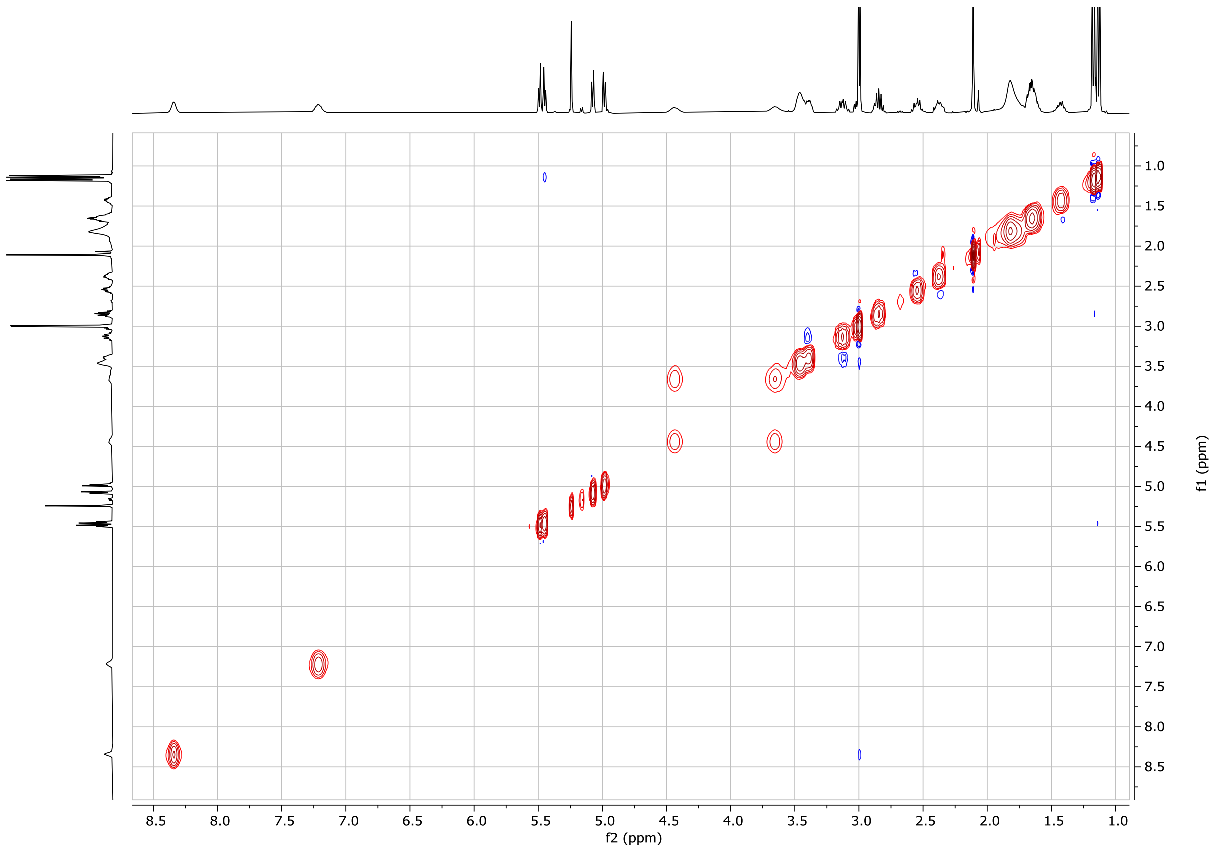

Figure S7: ^{1}H-^{13}C-HMBC (top) and ^{1}H-^{1}H-NOESY (bottom) spectrum (CD_2Cl_2, 298 K, 400 MHz) of **Ru-2a**.

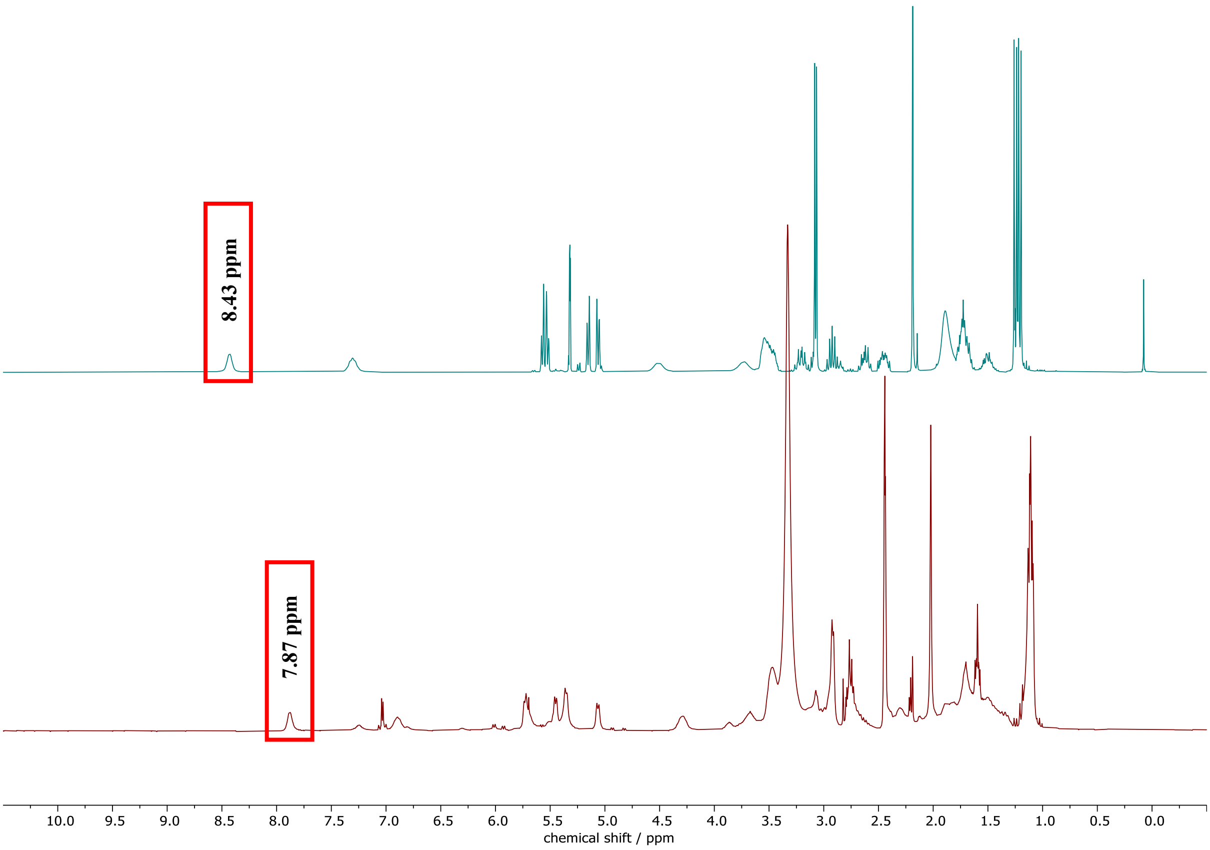

Figure S8: ^{1}H NMR spectra of **Ru-2a** in CD_2Cl_2 (top) and in DMSO-d_6 (bottom, both 298 K, 400 MHz), showing the solvent-dependent chemical shift of the N-H resonance (highlighted in red box).

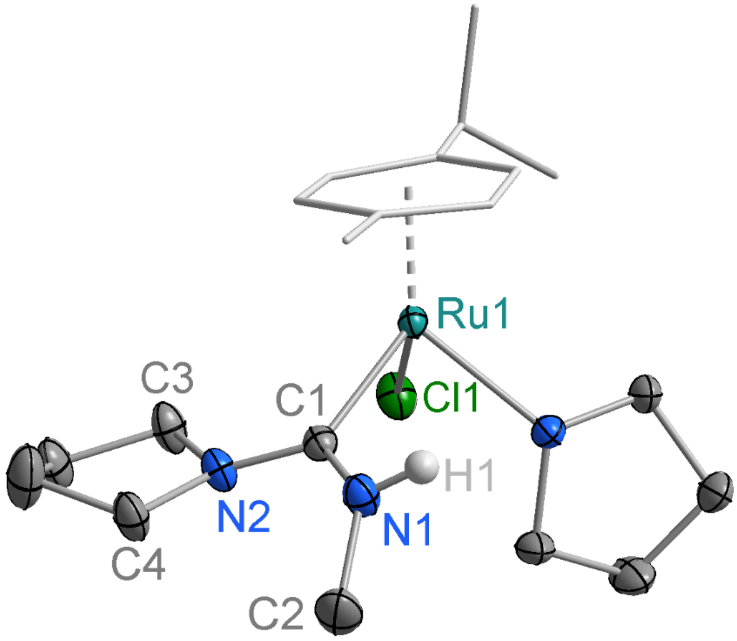

Figure S9: Molecular structure of **Ru-2a** with 30% probability displacement ellipsoids. All H (except H1) atoms have been omitted for clarity.

Synthesis of Ru-2b

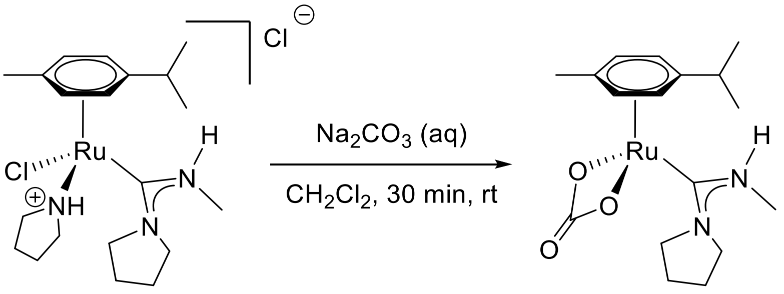

Ru-2a (190 mg, 0.39 mmol) was added to a one-neck round-bottom flask and dissolved in CH_2Cl_2 (20 mL), followed by the addition of saturated aqueous Na_2CO_3 solution (40 mL). The reaction mixture was stirred vigorously at room temperature for 30 minutes. The crude product was extracted with CH_2Cl_2 (3 × 15 mL), dried over Na_2SO_4, and the solvent was removed under vacuum. Recrystallization by slow diffusion of Et_2O into a solution of crude **Ru-2b** in CH_2Cl_2 afforded red crystals (70 mg, 40%).

^{1}H NMR (400 MHz, CD_2Cl_2) δ 6.69 (s, 1H, NH), 5.36, 5.32, 5.01, 4.98 (4 × d, J = 6.0 Hz, 1H, C_{cym}H), 3.90–3.84 (m, 1H, H_{pyr}), 3.48–3.32 (m, 3H, H_{pyr}), 2.93 (d, J = 4.6 Hz, 3H, N-CH_3), 2.72 (septet, J = 6.9 Hz, 1H, Cym-$CHMe_2$), 2.12 (s, 3H, Cym-CH_3), 1.95 (quintet, 2H, H_{pyr}), 1.83 – 1.76, 1.72 – 1.64 (2 × m, 1H, H_{pyr}), 1.27 (d, J = 6.9 Hz, 6H, Cym-CH(CH_3)$_2$).

^{13}C{^{1}H} NMR (101 MHz, CD_2Cl_2) δ 203.6 (NCN), 167.2 (CO_3), 107.9 (C_{cym}), 96.1 (C_{cym}), 83.3, 82.5, 80.8, 80.4 (4 × C_{cym}H), 53.8 (C_{pyr}), 47.4 (C_{pyr}), 35.4 (N-CH_3), 32.2 (Cym-$CHMe_2$), 25.9 (C_{pyr}), 25.6 (C_{pyr}), 22.8 (Cym-CH(CH$_3$)$_2$), 19.0 (Cym-CH$_3$).

Elemental analysis calcd. (%) for $C_{17}H_{26}N_2O_2Ru$: C, 50.11; H, 6.43; N, 6.88. Found: C, 48.77; H, 6.32; N, 6.46. Despite multiple attempts, no better CHN analysis of this compound were obtained.

HR-MS calcd. for $C_{17}H_{27}N_2O_3Ru$ $[M+H]^+$ 409.1060. Found: 409.1056.

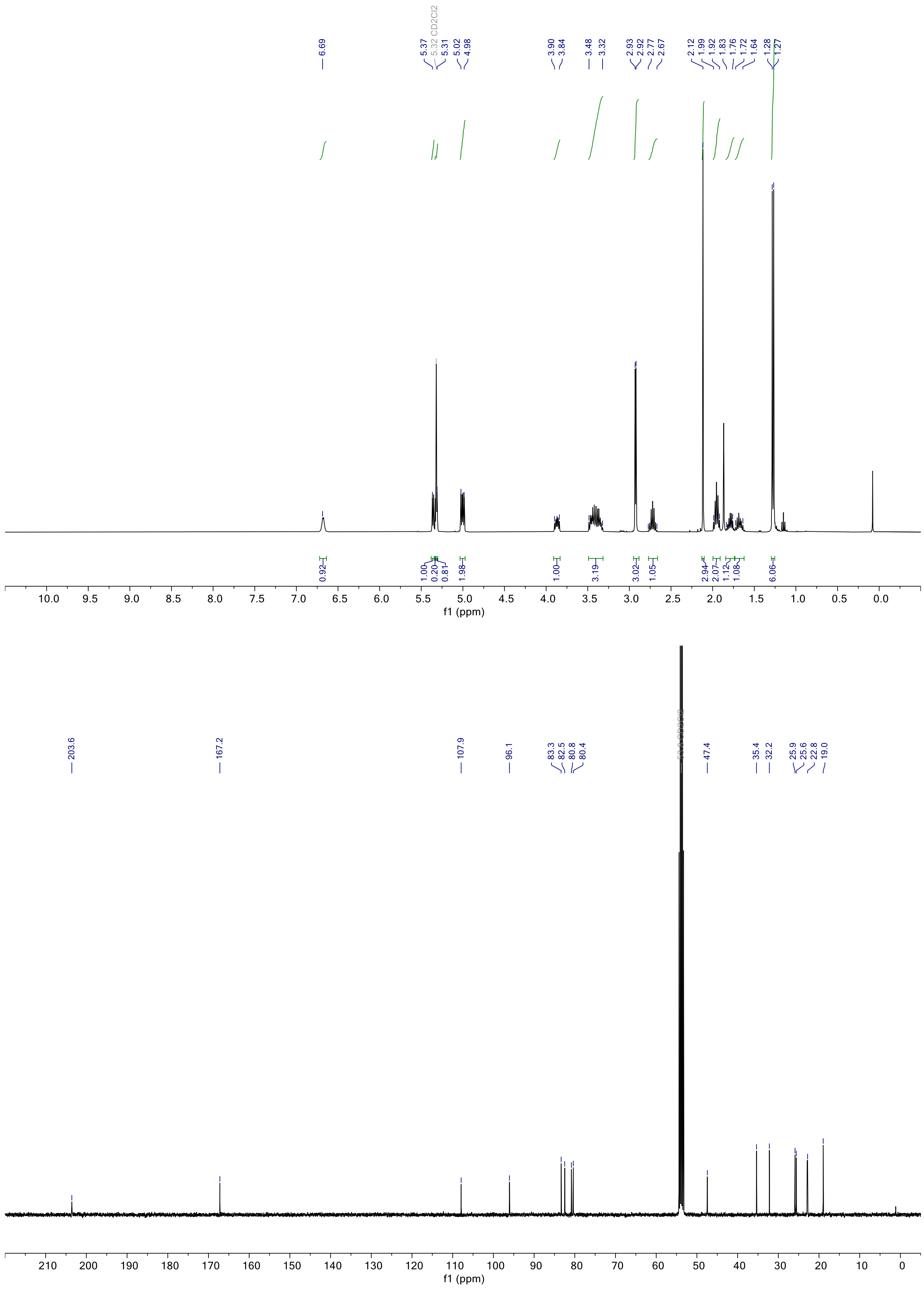

Figure S10:^{1}H (top) and $^{13}C\{^{1}H\}$ NMR (bottom) spectrum (CD_2Cl_2, 298 K, 400 MHz) of **Ru-2b**.

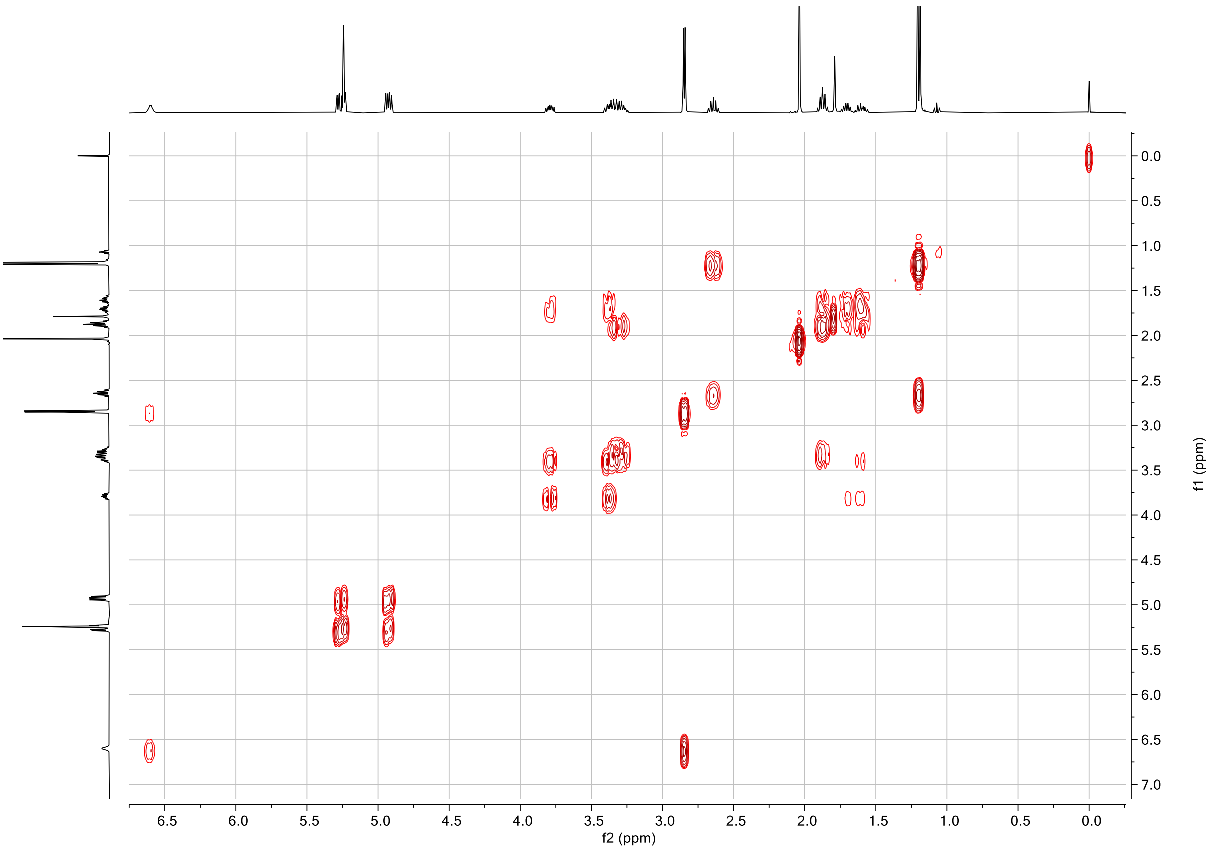

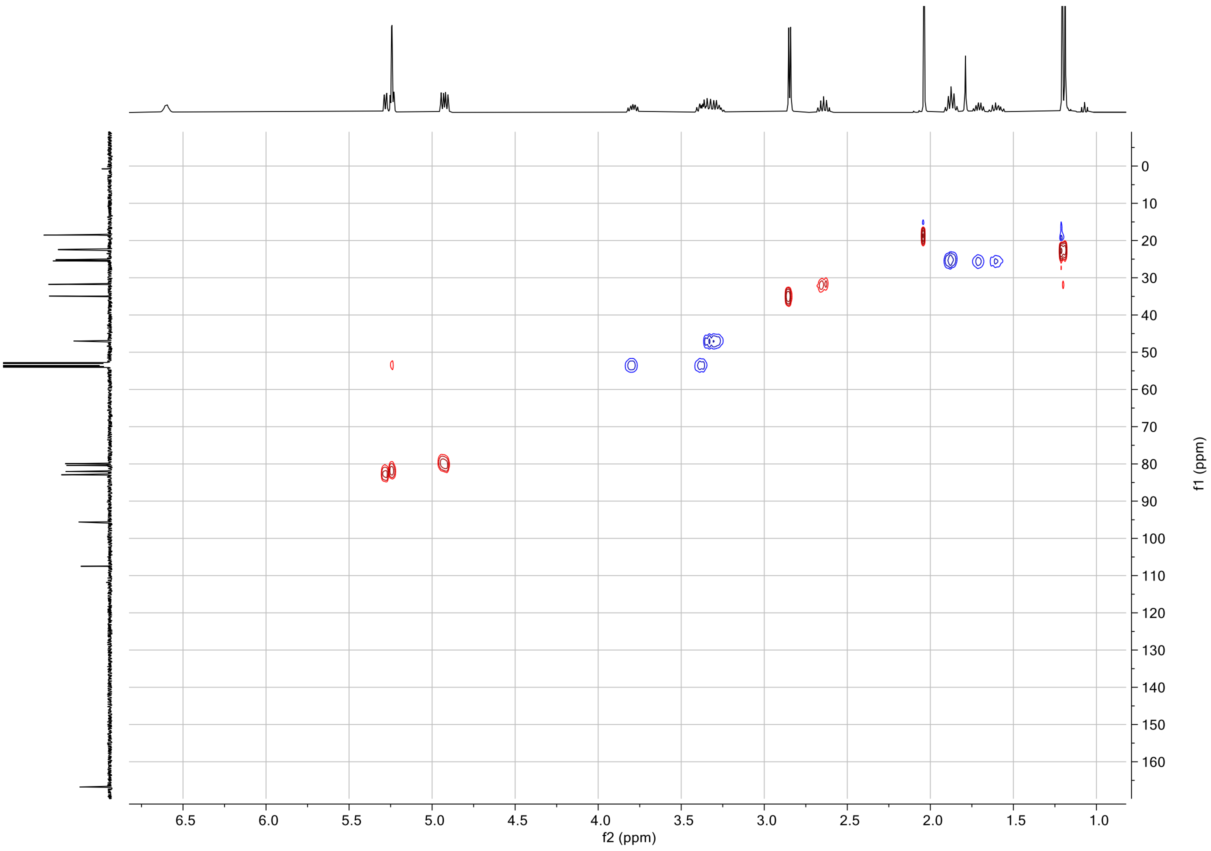

Figure S11: ^{1}H-^{1}H-COSY (top) and ^{1}H-^{13}C-HSQC (bottom) spectrum (CD_2Cl_2, 298 K, 400 MHz) of **Ru-2b**.

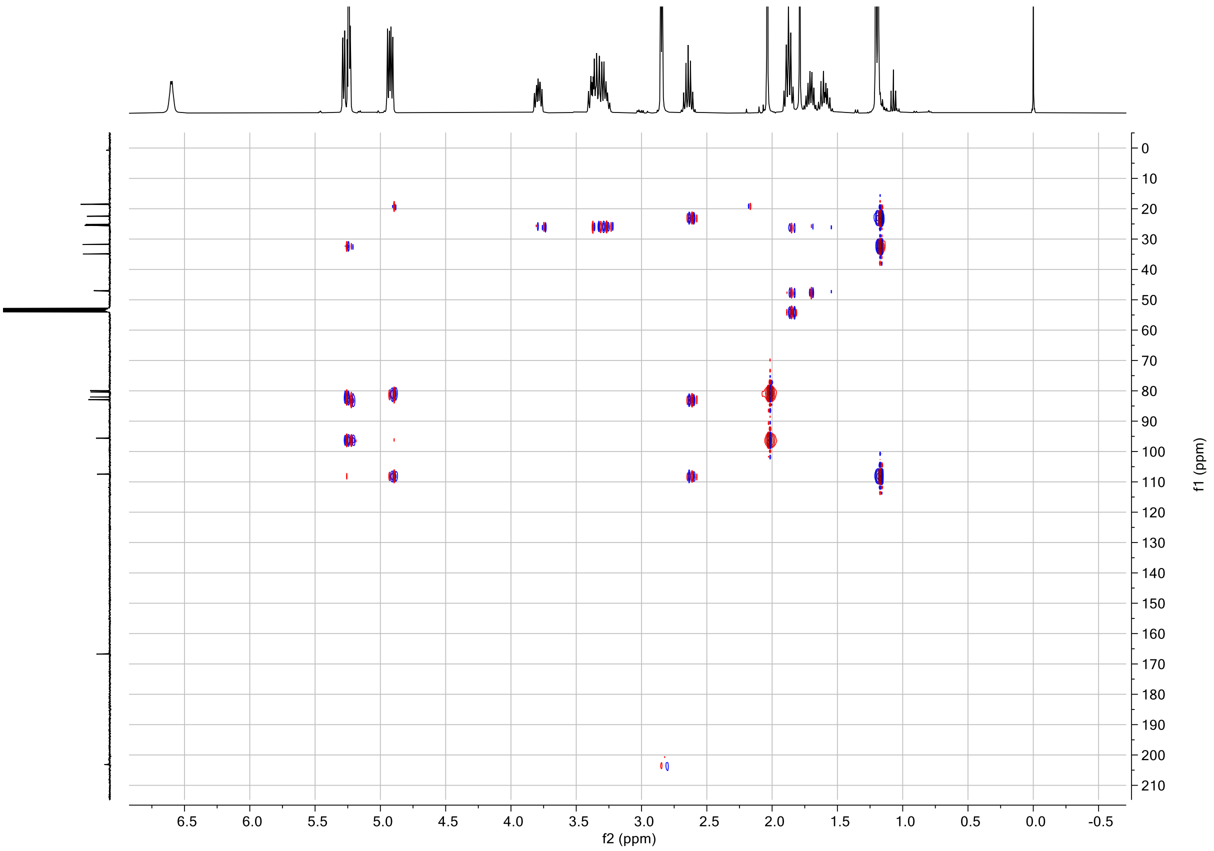

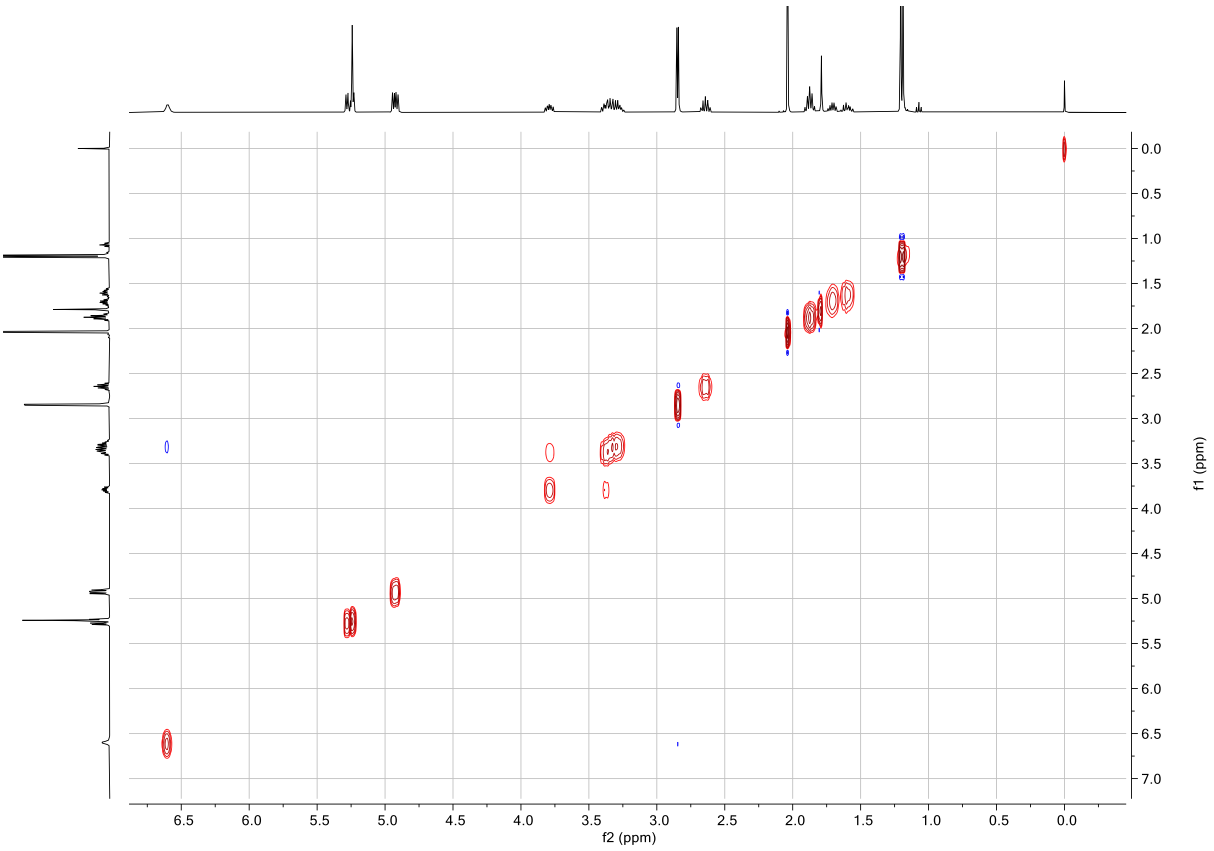

Figure S12: ^{1}H-^{13}C-HMBC (top) and ^{1}H-^{1}H-NOESY (bottom) spectrum (CD_2Cl_2, 298 K, 400 MHz) of **Ru-2b**.

Solvent dependent NH resonance in Ru-2b

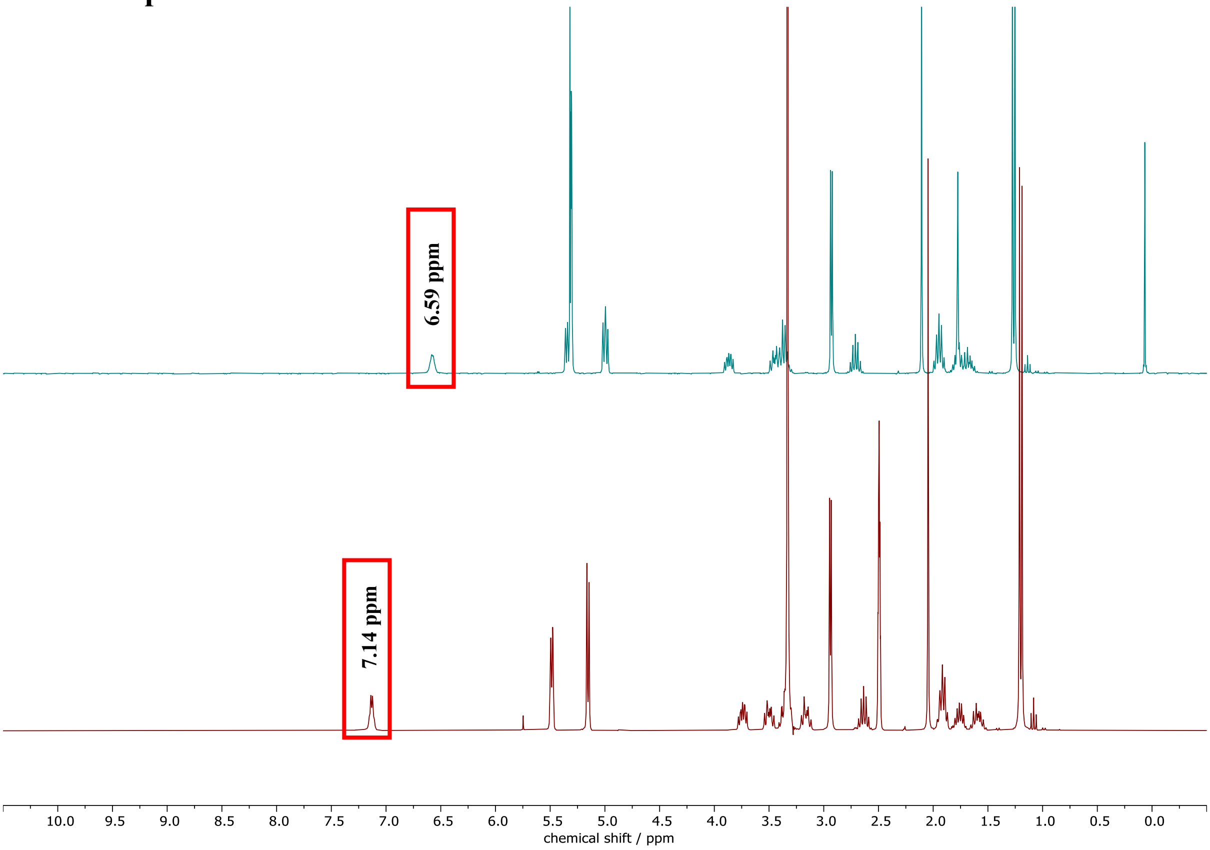

Figure S13: ^{1}H NMR spectra of **Ru-2b** in CD_2Cl_2 (top) and in DMSO-d_6 (bottom, both 298 K, 400 MHz), showing the solvent dependent chemical shift of the N-H resonance (highlighted in red boxes).

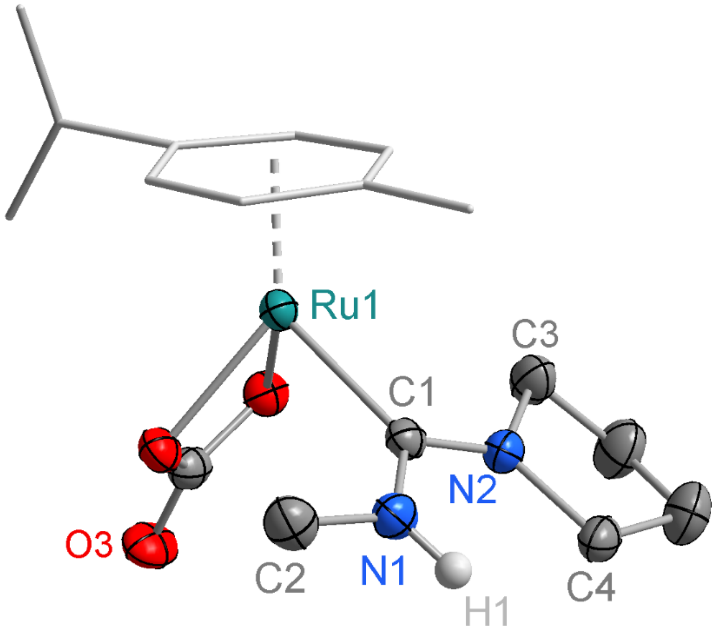

Figure S14: Molecular structure of **Ru-2b** with 30% probability displacement ellipsoids. All H atoms (except H1) have been omitted for clarity. Positional disorder has been omitted for clarity.

Synthesis of Ru-2c

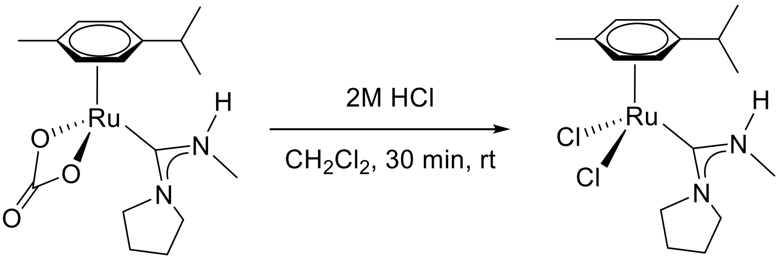

Ru-2b **Ru-2c**

Ru-2a (190 mg, 0.39 mmol) was added to a round-bottom flask and dissolved in CH_2Cl_2 (20 mL), followed by the addition of saturated aqueous Na_2CO_3 solution (40 mL) The mixture was stirred vigorously at room temperature for 30 minutes (formation of **Ru-2b** *in situ*). The yellow solution was extracted with CH_2Cl_2 (1 × 15 mL), and HCl (2 M, 30 mL) was subsequently added to the organic phase. The resulting orange-red solution was stirred for 30 minutes at room temperature and extracted with CH_2Cl_2 (3 × 15 mL). The combined organic phases were dried over Na_2SO_4, and the solvent was removed under vacuum. Recrystallization by diffusion of Et_2O into a solution of the crude product in CH_2Cl_2 afforded **Ru-2c** as red crystals (110 mg, 69%).

^{1}H NMR (400 MHz, CD_2Cl_2) δ 7.24 (s, 1H, N*H*), 5.24, 5.04 (2 × d, J = 5.9 Hz, 2H, C_{cym}H), 3.93, 3.66 (2 × br s, 2H, H_{pyr}), 3.10 (d, J = 5.4 Hz, 3H, N-CH_3), 2.76 (septet, J = 6.9 Hz, 1H, Cym-$CHMe_2$), 2.15 (s, 3H, Cym-CH_3), 1.88 (br s, 4H, H_{pyr}), 1.24 (d, J = 6.9 Hz, 6H, Cym-CH(C*H*$_3$)$_2$).

^{13}C{^{1}H} NMR (101 MHz, CD_2Cl_2) δ 209.6 (NCN), 105.1 (C_{cym}), 102.3 (C_{cym}), 84.6 (C_{cym}H), 84.1 (C_{cym}H), 56.4 (C_{pyr}), 53.3 (C_{pyr}), 36.4 (N-CH_3), 31.2 (Cym-$CHMe_2$), 26.4 (C_{pyr}), 25.3 (C_{pyr}), 23.5 (Cym-CH(*C*H$_3$)$_2$), 18.5 (Cym-*C*H$_3$).

Elemental analysis calcd. (%) for $C_{16}H_{26}Cl_2N_2Ru$: C, 45.93; H, 6.26; N, 6.70. Found: C, 45.64; H, 6.03; N, 6.39.

HR-MS calcd. for $C_{16}H_{26}N_2ClRu$ $[M–Cl]^+$ 383.0823. Found: 383.0820.

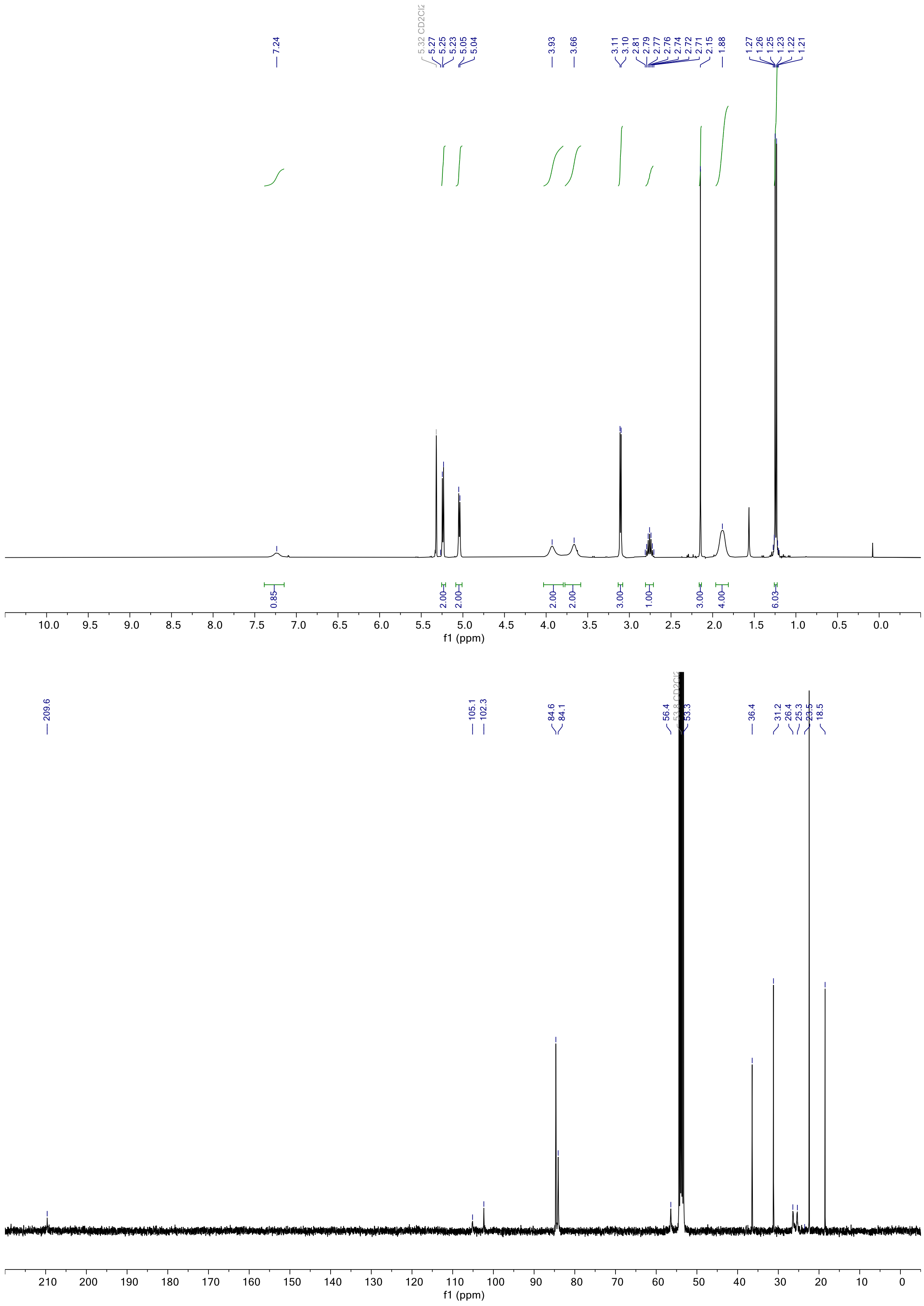

Figure S15:^{1}H (top) and ^{13}C{^{1}H} NMR (bottom) spectrum (CD_2Cl_2, 298 K, 400 MHz) of **Ru-2c**.

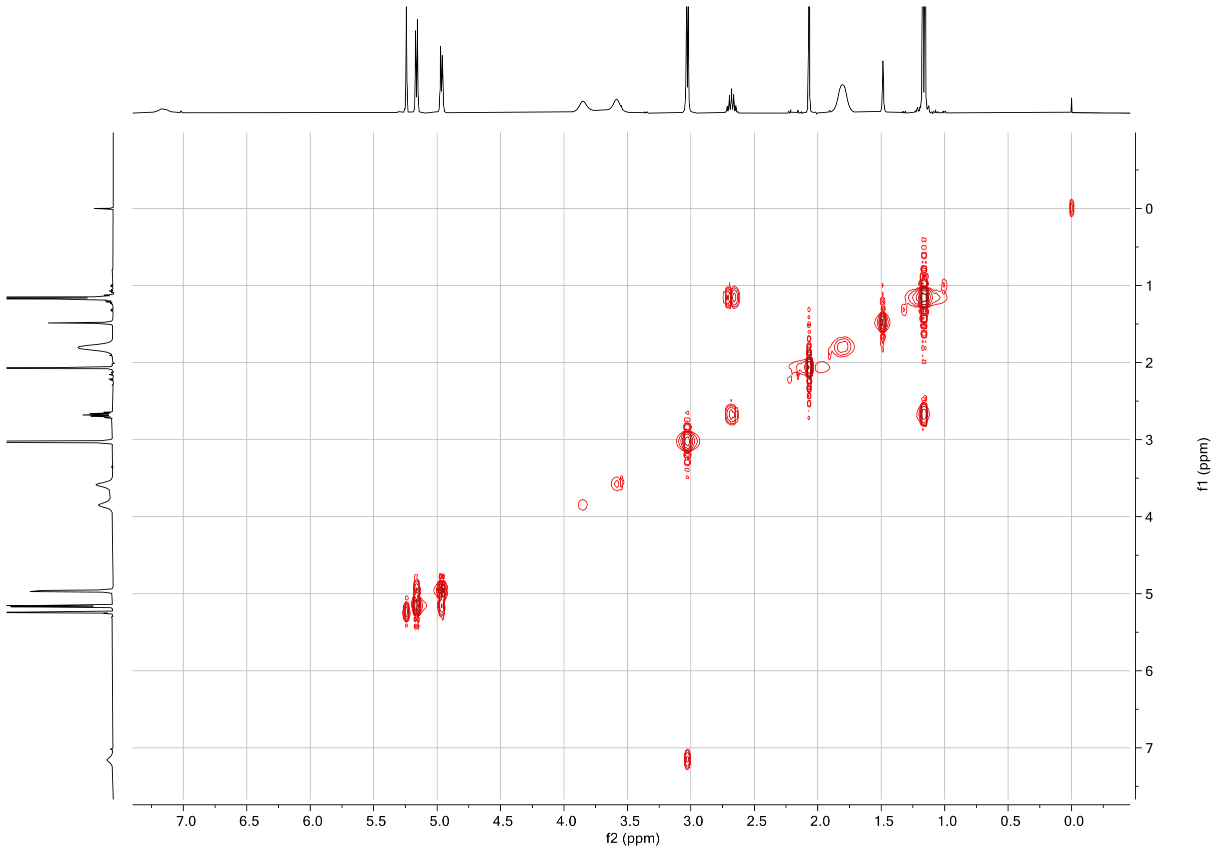

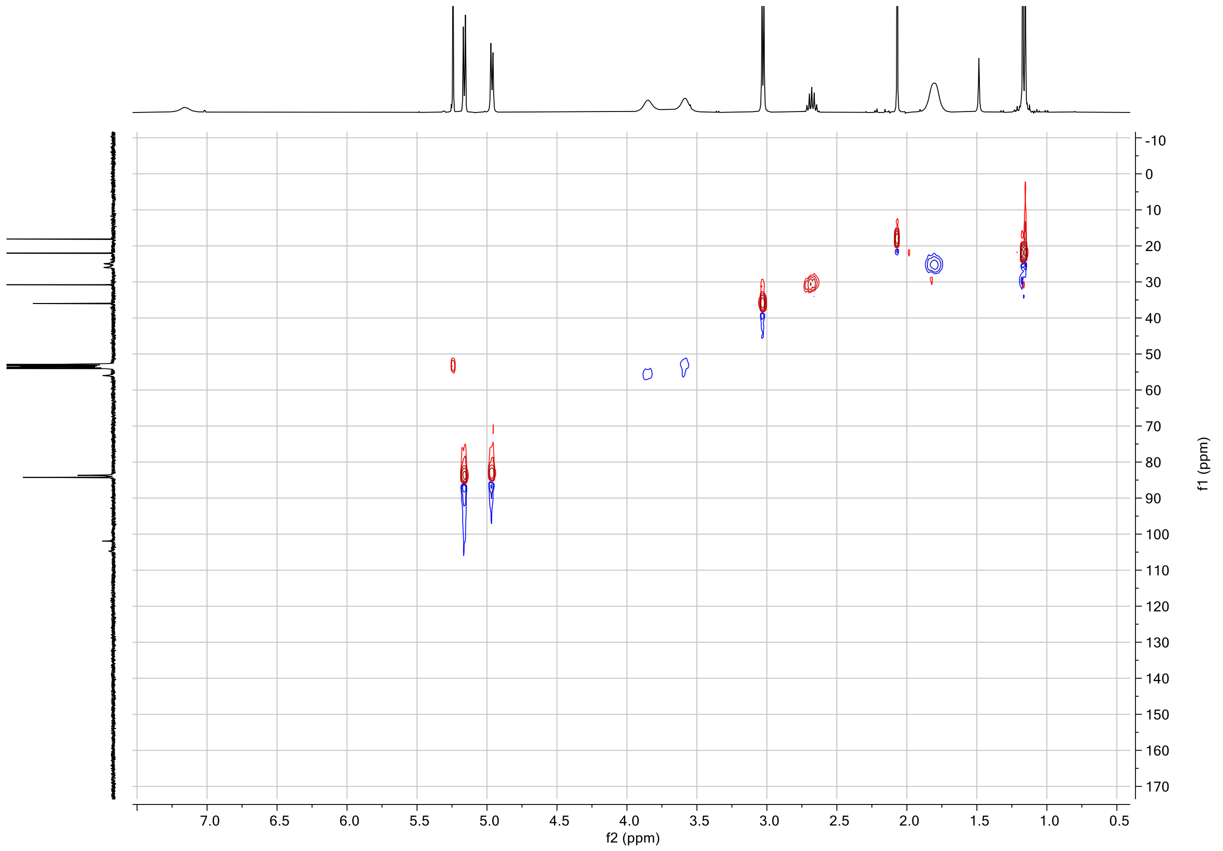

Figure S16:^{1}H-^{1}H-COSY (top) and ^{1}H-^{13}C-HSQC (bottom) spectrum (CD_2Cl_2, 298 K, 400 MHz) of **Ru-2c**.

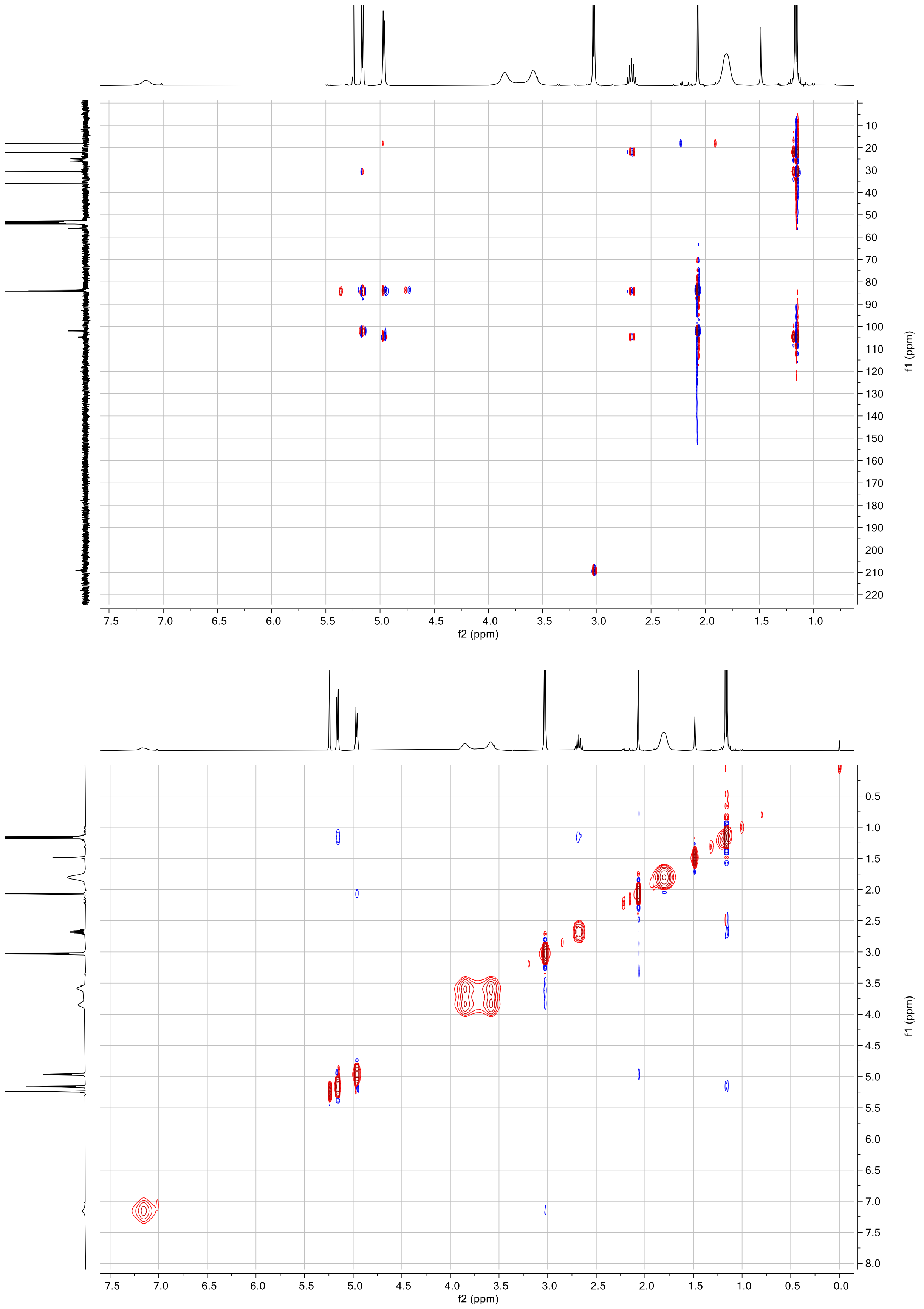

Figure S17: ^{1}H-^{13}C-HMBC (top) and ^{1}H-^{1}H-NOESY (bottom) spectrum (CD_2Cl_2, 298 K, 400 MHz) of **Ru-2c**.

H/D exchange in Ru-2c

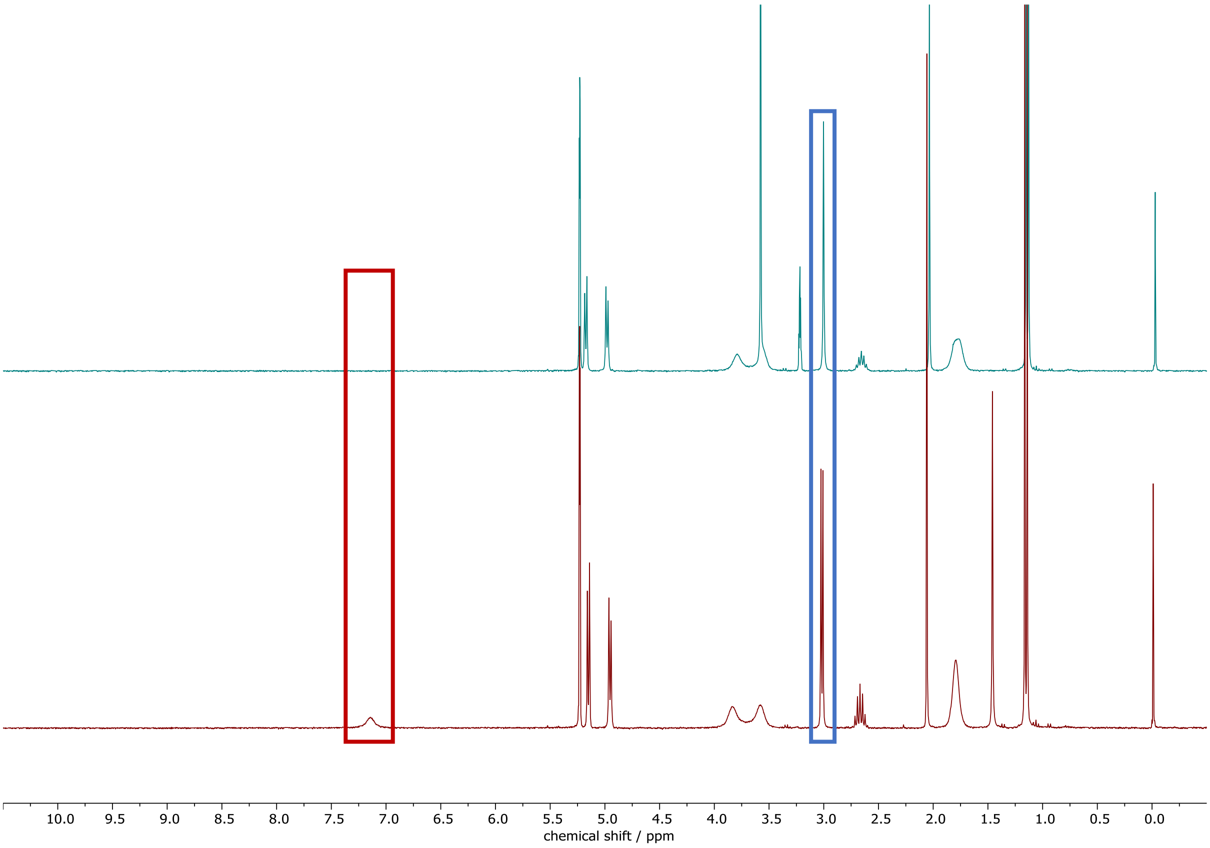

Figure S18: ^{1}H NMR spectrum (298 K, 400 MHz) of **Ru-2c** in CD_2Cl_2 (bottom) and in CD_3OD (top). H/D exchange is highlighted by disappearance of the N-H signal at $\delta = 7.23$ ppm (highlighted in red box) and transition of N-CH_3 doublet ($\delta = 3.12$ ppm) to a singlet (highlighted in blue box).

Variable Temperature NMR spectroscopy of Ru-2c

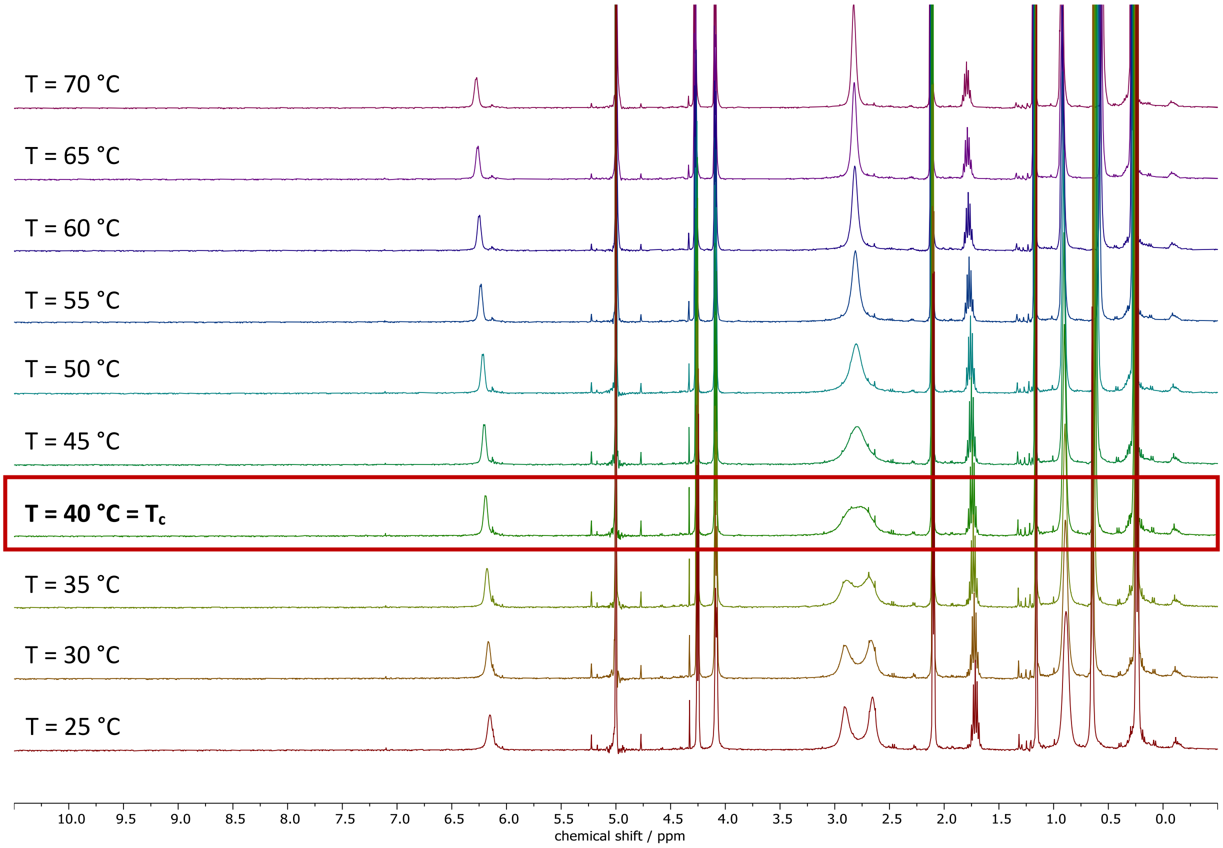

Figure S19: Stacked ^{1}H NMR spectra (298–343 K, 400 MHz) of **Ru-2c** in $C_2D_2Cl_4$. T_c is identified to be at 40 °C (highlighted in red).

Calculation of ΔG‡

ΔG‡ of the rotational barrier can be calculated using eqs. **(1)** and **(2)** as follows:

$$k = \frac{\pi \Delta \nu}{2\sqrt{2}} \quad \textbf{(1)}$$

$$\Delta G^{\ddagger} = RT_c ln\left(\frac{k_b T_c}{hk}\right) \quad \textbf{(2)}$$

With R=8.314 J $mol^{-1}K^{-1}$, k_B=1.380649×10^{-23} J K^{-1}, h = 6.62607015×10^{-34} Js , T_c = 313 K, Δν = 120.27 Hz, **ΔG‡ ≈ 14.9 kcal/mol**

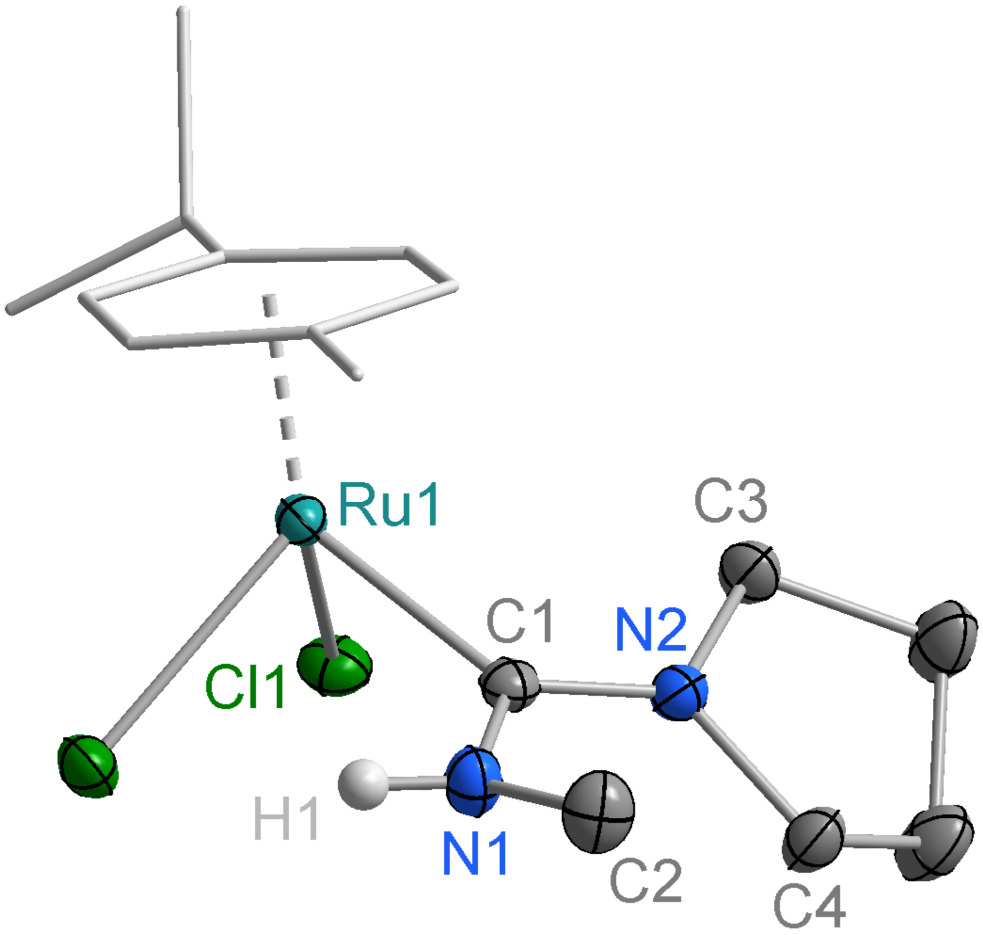

Figure S20: Molecular structure of **Ru-2c** with 30% probability displacement ellipsoids. All H atoms (except H1) have been omitted for clarity.

Synthesis of Ru-5

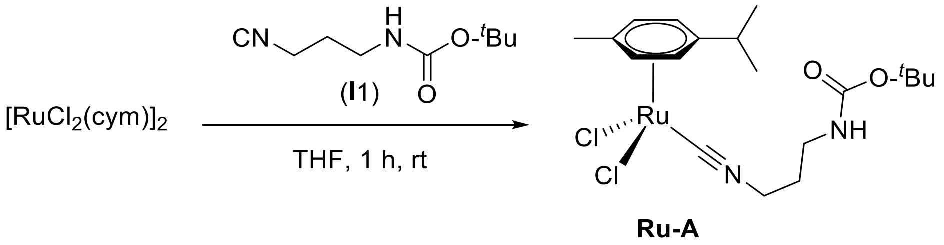

Isocyanide **I**1 (0.75 g, 4.1 mmol) was added to a suspension of $[RuCl_2(cym)]_2$ (0.7 g, 1.1 mmol) in THF (30 mL). The reaction mixture was stirred for 15 minutes at room temperature until a clear solution was obtained. Evaporation of THF resulted in a dark orange oil which was subsequently washed with Et_2O to remove any impurities. The resulting orange oil was dried *in vacuo* to give **Ru-A** as needle-shaped crystals (980 mg, 87%).

^{1}H NMR (400 MHz, CD_2Cl_2) δ 5.62 (d, J = 6.0 Hz, 2H, Ar-*H*), 5.42 (d, J = 6.1 Hz, 2H, Ar-*H*), 5.14 (t, J = 6.2 Hz, 1H, N-*H*), 3.82 (t, J = 6.4 Hz, 2H, CH_2), 3.25 (d, J = 6.4 Hz, 2H, CH_2), 2.80 (p, J = 7.0 Hz, 1H, Ar-C*H*), 2.20 (s, 3H, Ar-CH_3), 1.95 – 1.84 (m, 2H, CH_2), 1.35 (s, 9H, O-(CH_3)$_2$), 1.21 (d, J = 6.9 Hz, 6H, Ar-(CH_3)$_2$).

^{13}C{^{1}H} NMR (101 MHz, CD_2Cl_2) δ 156.2 (*C*=O), 107.2 (Ar-*C*), 106.3 (Ar-*C*), 87.8 (Ar-*C*), 87.4 (Ar-*C*), 77.5 (*C*≡N), 42.5 (*C*H_2), 36.7 (*C*H_2), 31.0 (Ar-*C*H), 29.2 (*C*H_2), 28.3 (OC(*C*H_3)$_2$), 22.4 (Ar-(*C*H_3)$_2$), 18.7 (Ar-*C*H_3).

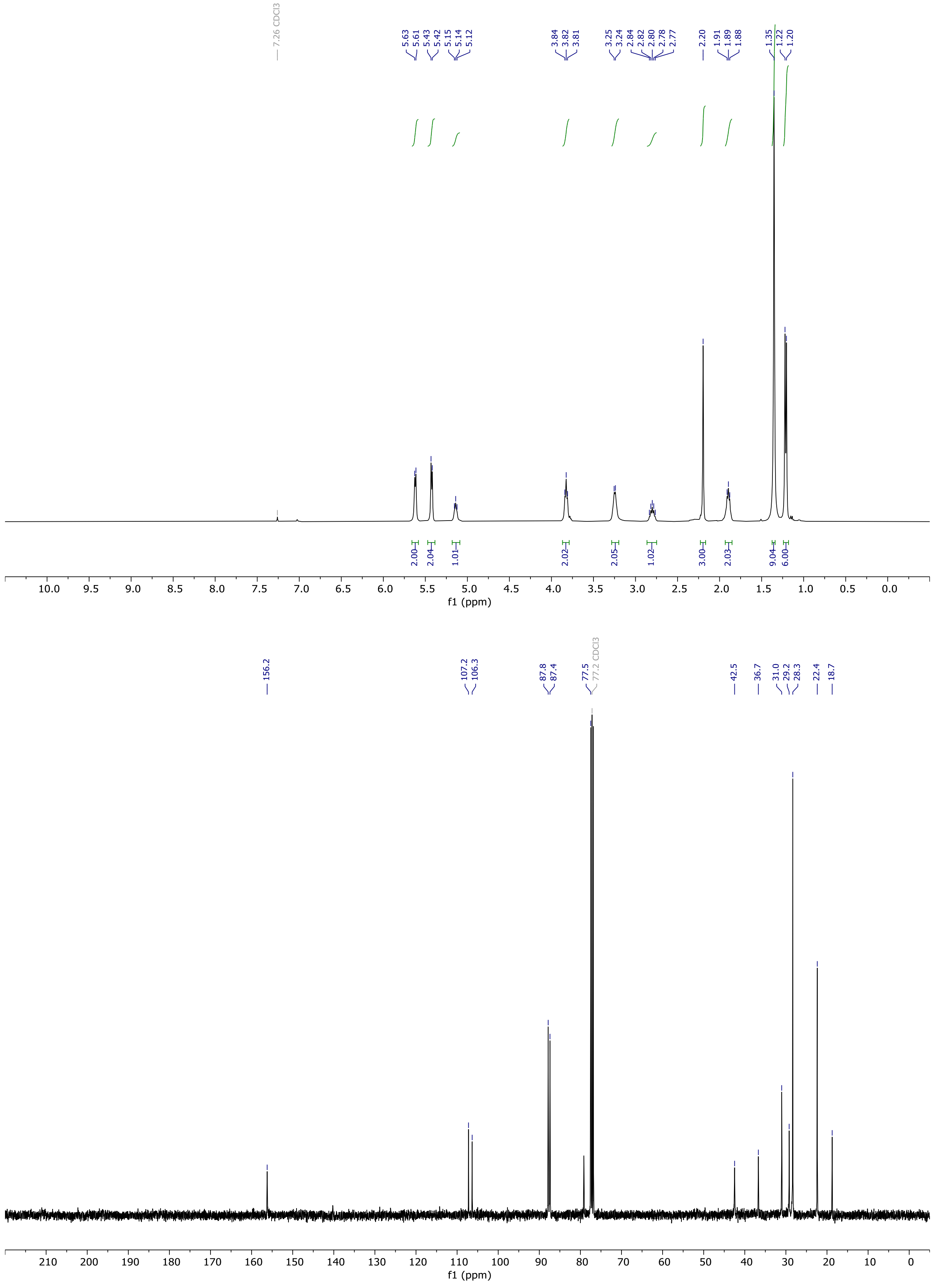

Figure S21:^{1}H (top) and ^{13}C{^{1}H} NMR (bottom) spectrum ($CDCl_3$, 298 K, 400 MHz) of **Ru-A**.

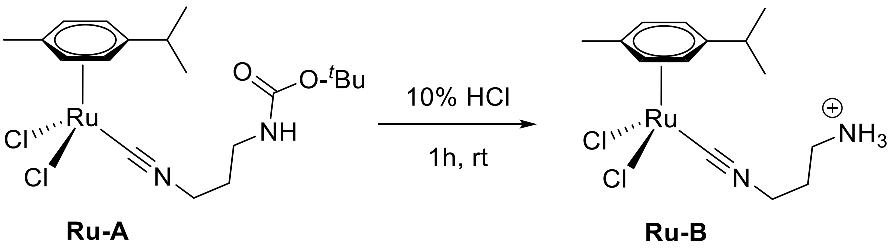

Ru-A (0.86 g, 1.8 mmol) was dissolved in HCl (10% dioxane solution, 10 g). The reaction mixture was stirred for 1 hour at room temperature resulting in precipitation of a brown oil. Then Et_2O (30 mL) was added, followed by decanting off the supernatants. The crude product was washed with Et_2O and dried *in vacuo* to obtain **Ru-B** (700 mg, 94%),

^{1}H NMR (400 MHz, CD_2Cl_2) δ 8.06 (s, 3H, NH_3), 5.81 (br s, 2H, H_{cym}), 5.66 (br s, 2H, H_{cym}), 4.30 (br s, 2H, CH_2), 3.24 (br s, 2H, CH_2), 2.87 (br s, 1H, Cym-$CHMe_2$), 2.35 (br s, 2H, CH_2), 2.26 (s, 3H, Cym-CH_3), 1.29 (d, J = 6.9 Hz, 6H, Cym-CH(CH_3)$_2$).

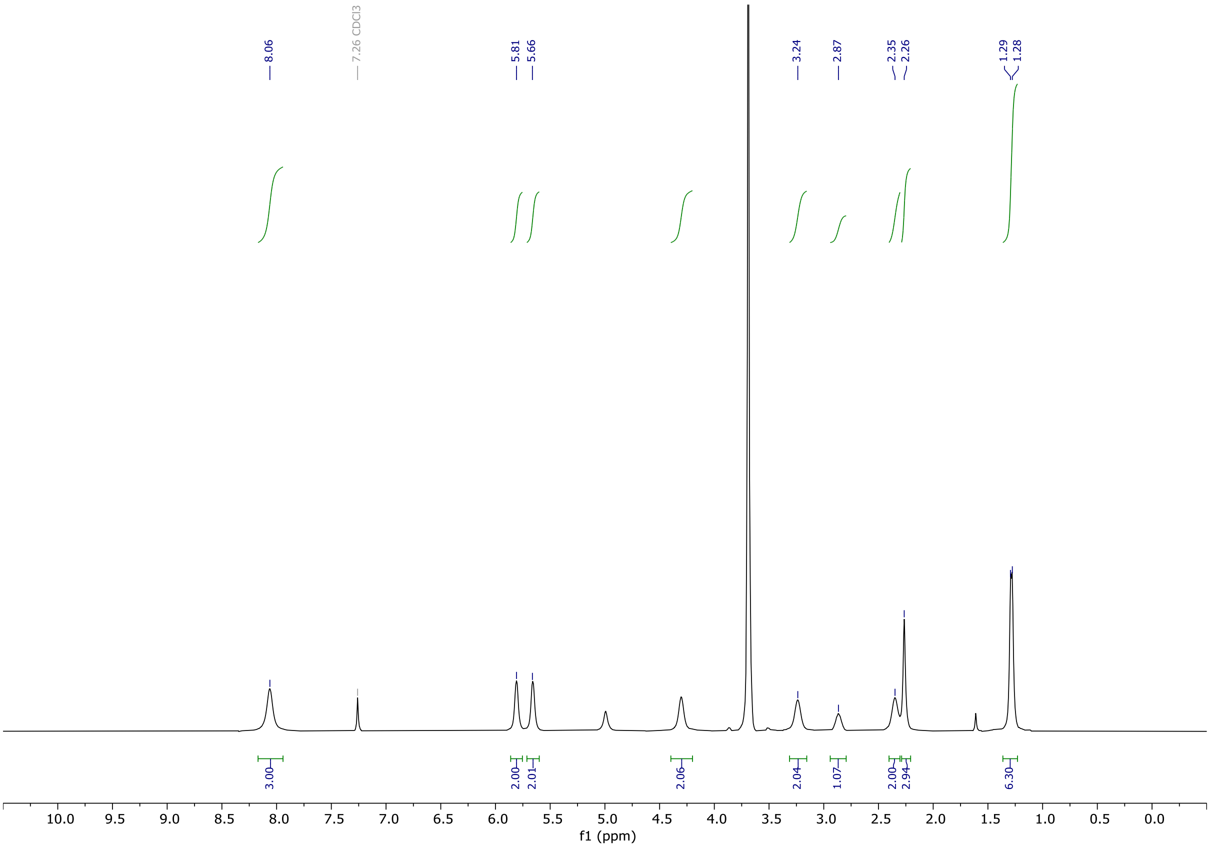

Figure S22:^{1}H spectrum ($CDCl_3$, 298 K, 400 MHz) of **Ru-B**.

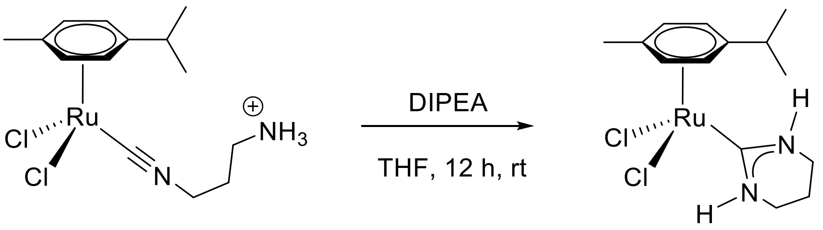

Ru-B **Ru-5**

Ru-B (1.0 g, 2.3 mmol) was dissolved in THF (20 mL), followed by the addition of NEt*i*Pr_2 (1.5 g, 15 mmol). The reaction mixture was stirred for 12 hours at room temperature. The formed precipitate was filtered, washed with Et_2O and dried *in vacuo*. The residue was extracted into acetone (3 × 30 mL), and the combined organic phases were collected and dried at reduced pressure. The residue was re-dissolved in MeCN (5 mL) and stirred for 1 hour at –20 °C. The precipitated solid ([HNEt*i*Pr_2]Cl) was filtered off. After three days at –20 °C, orange crystals were obtained, which were washed with cold MeCN (2 mL) and dried *in vacuo* to obtain **Ru-5** (640 mg, 70%).

^{1}H NMR (400 MHz, CD_2Cl_2) δ 6.82 (s, 2H, NH), 5.25, 5.05 (2 × d, J = 5.9 Hz, 2H, H_{cym}), 3.40–3.32 (m, 4H, CH_2), 2.79 (septet, J = 7.0 Hz, 1H, Cym-$CHMe_2$), 2.13 (s, 3H, Cym-CH_3), 1.99 (quintet, J = 5.9 Hz, 2H, CH_2), 1.24 (d, J = 7.0 Hz, 6H, Cym-CH(CH_3)$_2$).

^{13}C{^{1}H} NMR (101 MHz, CD_2Cl_2) δ 200.5 (NCN), 104.9 (C_{cym}), 100.3 (C_{cym}), 85.8 (C_{cym}H), 84.6 (C_{cym}H), 41.6 (*C*H_2), 31.1 (Cym-$CHMe_2$), 22.6 (Cym-CH(*C*H_3)$_2$), 20.7 (CH_2), 18.6 (Cym-CH_3).

Elemental analysis calcd. (%) for $C_{14}H_{22}Cl_2N_2Ru$: C, 43.08; H, 5.68; N, 7.18. Found: C, 43.26; H, 5.68, N, 7.08.

HR-MS calcd. for $C_{14}H_{22}N_2ClRu$ $[M–Cl]^+$ 355.0510. Found: 355.0506.

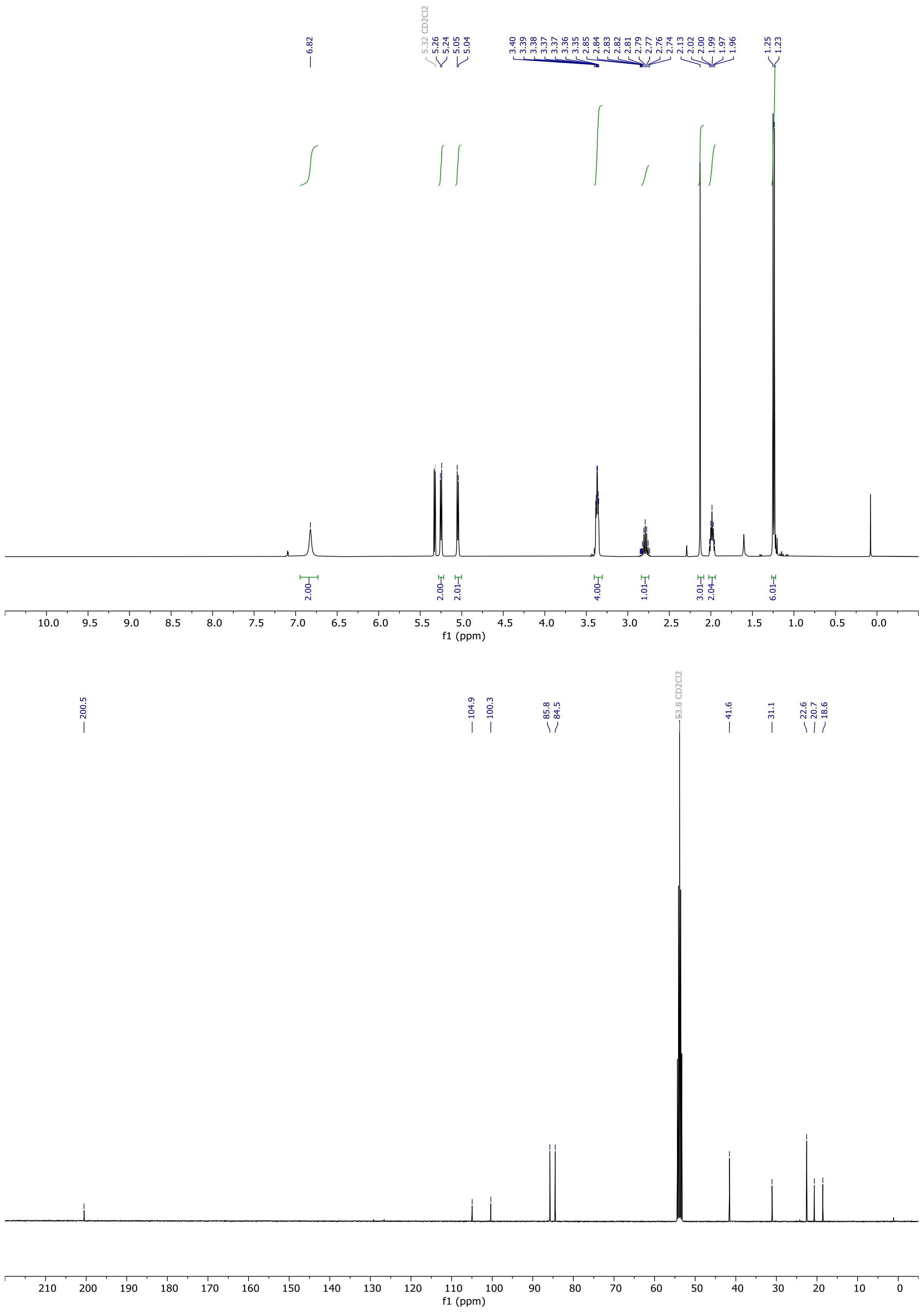

Figure S23: ^{1}H (top) and $^{13}C\{^{1}H\}$ NMR (bottom) spectrum (CD_2Cl_2, 298 K, 400 MHz) of **Ru-5**.

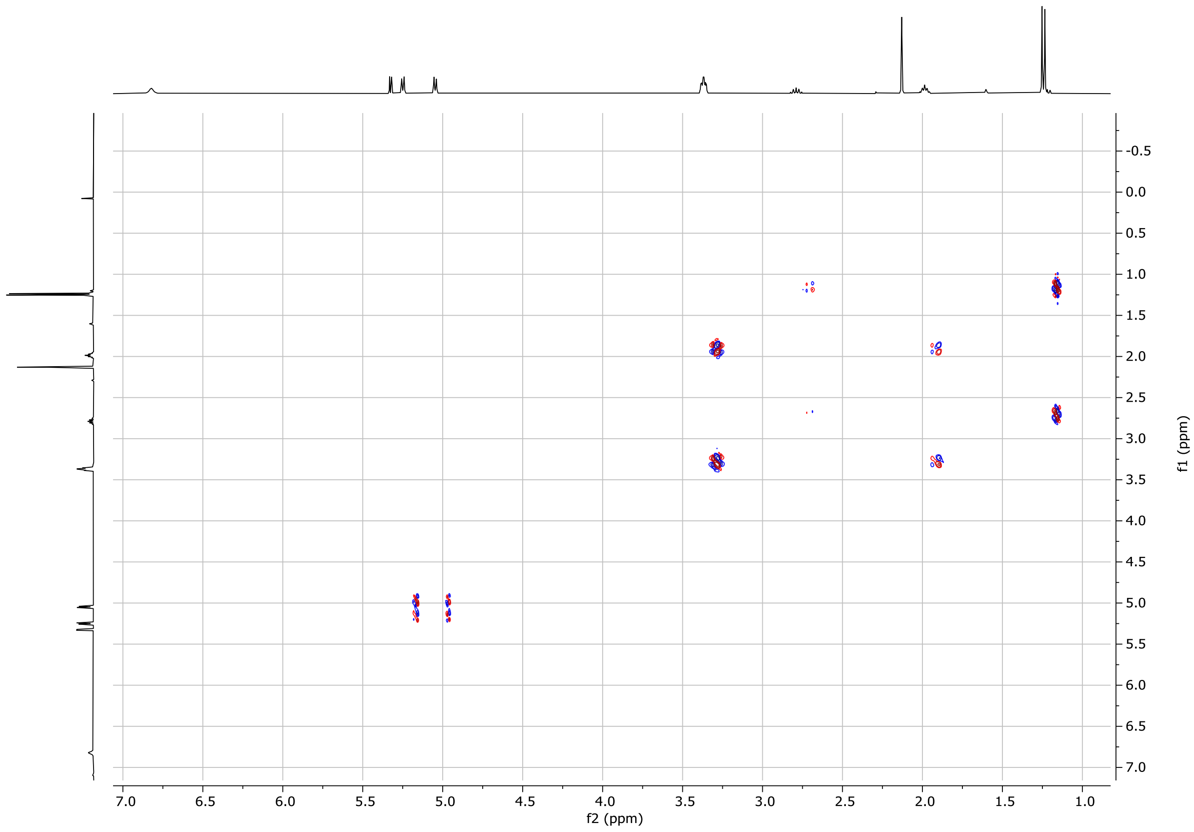

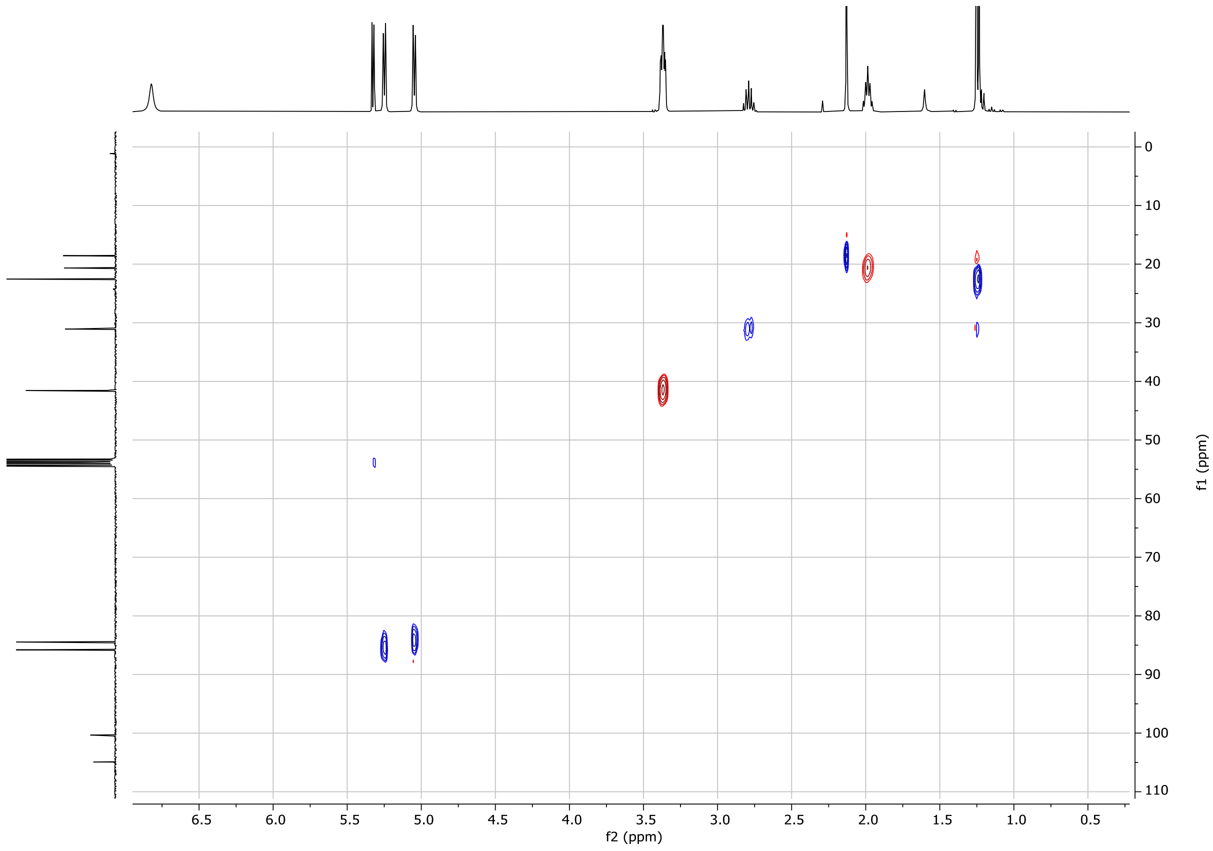

Figure S24:^{1}H-^{1}H-COSY (top) and ^{1}H-^{13}C-HSQC (bottom) spectrum (CD_2Cl_2, 298 K, 400 MHz) of **Ru-5**.

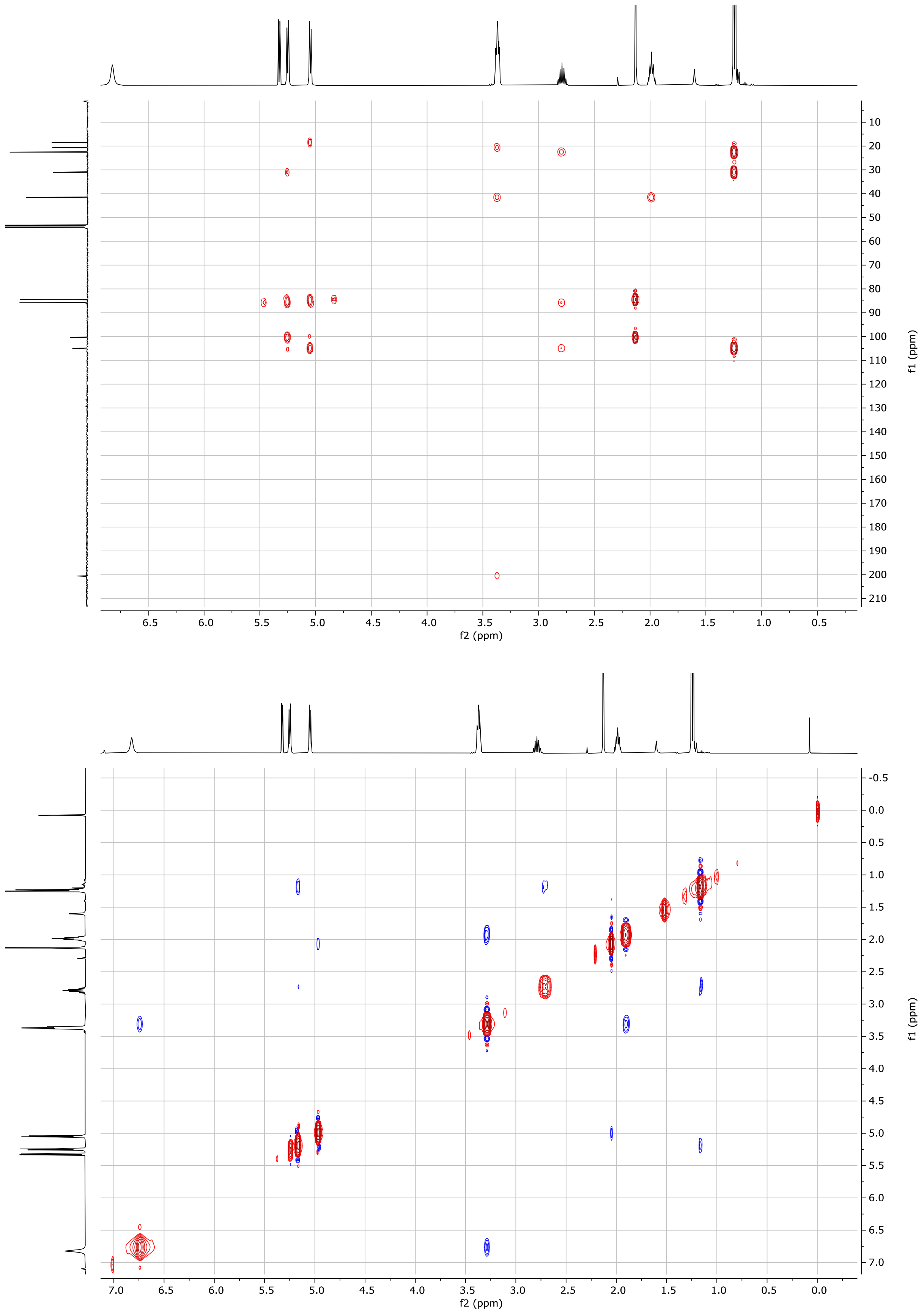

Figure S25: ^{1}H-^{13}C-HMBC (top) and ^{1}H-^{1}H-NOESY (bottom) spectrum (CD_2Cl_2, 298 K, 400 MHz) of **Ru-5**.

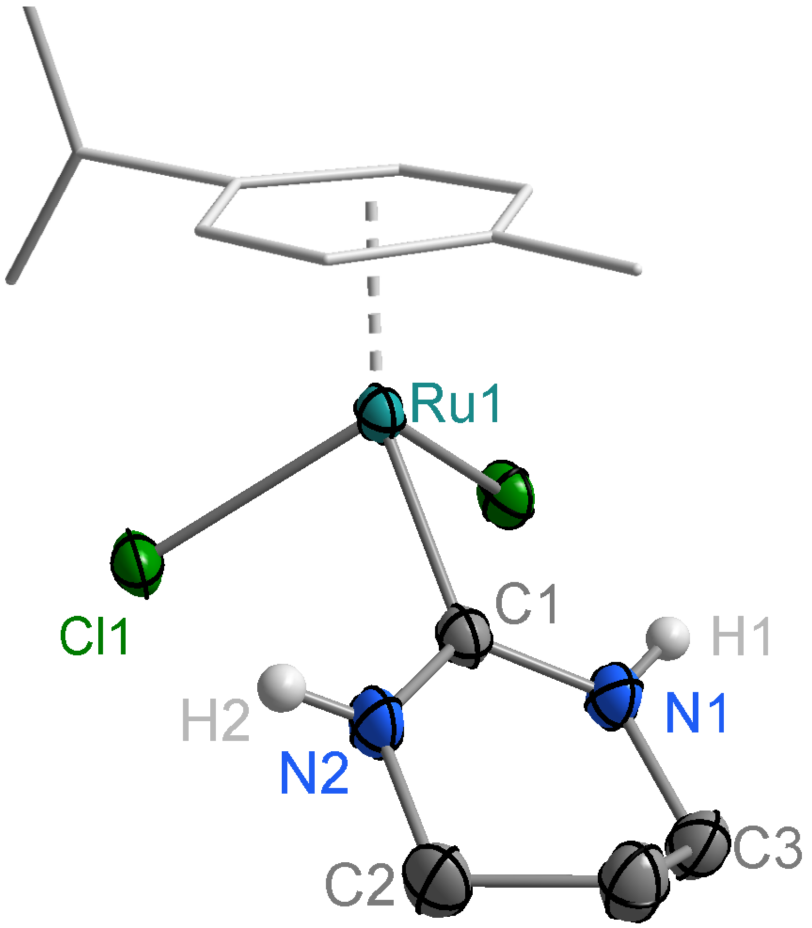

Figure S26: Molecular structure of **Ru-5** with 50% probability displacement ellipsoids. All H atoms (except H1 and H2) have been omitted for clarity.

Synthesis of Ru-6

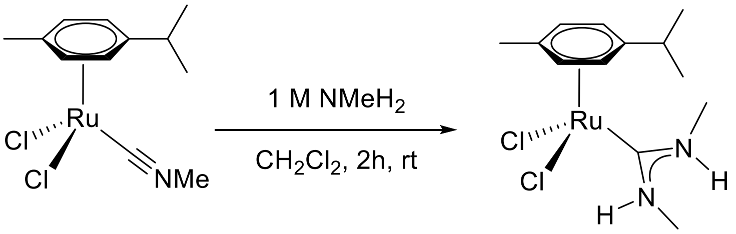

Ru-1 **Ru-6**

Ru-1 (200 mg, 0.58 mmol) was added to a microwave vial and dissolved in CH_2Cl_2 (3 mL), followed by the addition of $NMeH_2$ (1 M solution in THF, 6 mL). The mixture was stirred vigorously at room temperature for 2 h. Then HCl (1 M, 10 mL) was added and subsequently CH_2Cl_2 (15 mL). The aqueous phase was extracted and evaporated to dryness. The residue was suspended in CH_2Cl_2 (10 mL) dried over Na_2SO_4, filtered and the solvent was removed under vacuum. The product was washed with pentane and re-crystallized by Et_2O diffusion into a CH_2Cl_2 solution of the product to afford **Ru-6** as red crystals (104 mg, 58%).

^{1}H NMR (400 MHz, CD_2Cl_2) δ 7.39 (s, 1H, NH), 6.09 (s, 1H, NH), 5.28 (d, J = 5.8 Hz, 2H, H_{cym}), 5.11 (d, J = 5.8 Hz, 2H, H_{cym}), 3.28 (d, J = 4.9 Hz, 3H, N-CH_3), 2.89 (d, J = 5.0 Hz, 3H, N-CH_3), 2.75 (septet, J = 6.9 Hz, 1H, Cym-$CHMe_2$), 2.15 (s, 3H, Cym-CH_3), 1.22 (d, J = 6.9 Hz, 6H, Cym-CH(CH_3)$_2$).

^{13}C{^{1}H} NMR (101 MHz, CD_2Cl_2) δ 205.3 (NCN), 105.2 (C_{cym}), 102.1 (C_{cym}), 85.1 (C_{cym}H), 84.3 (C_{cym}H), 36.0 (N-CH_3), 31.1 (Cym-$CHMe_2$), 29.8 (N-CH_3), 22.4 (Cym-CH(CH$_3$)$_2$), 18.4 (Cym-CH_3).

Elemental analysis calcd. (%) for $C_{13}H_{22}Cl_2N_2Ru$: C, 41.27; H, 5.86; N, 7.41. Found: C, 41.34; H, 5.88; N, 7.42.

HR-MS calcd. for $C_{13}H_{22}N_2ClRu$ $[M–Cl]^+$ 343.0510. Found: 343.0507.

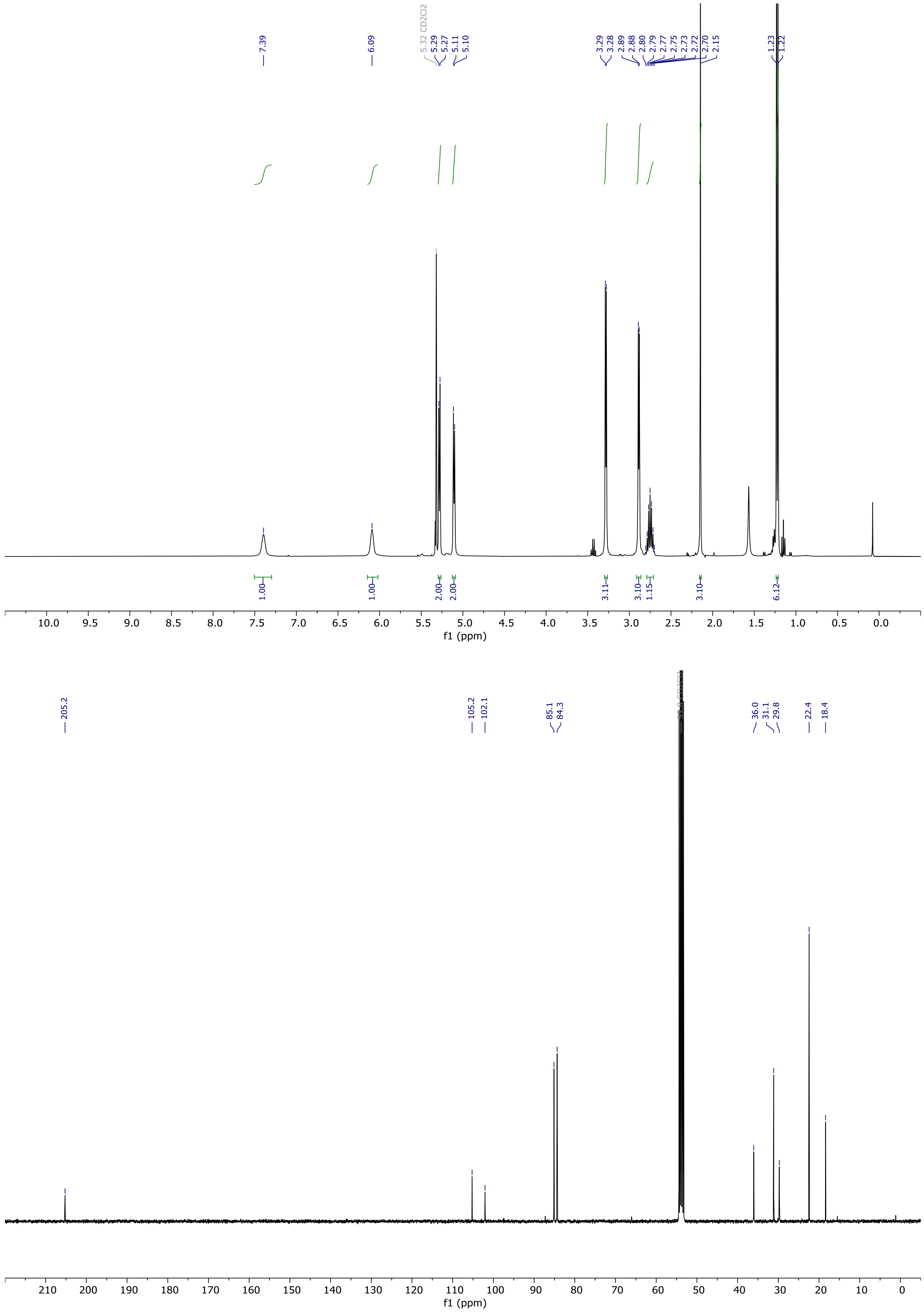

Figure S27:^{1}H (top) and ^{13}C{^{1}H} NMR (bottom) spectrum (CD_2Cl_2, 298 K, 400 MHz) of **Ru-6**.

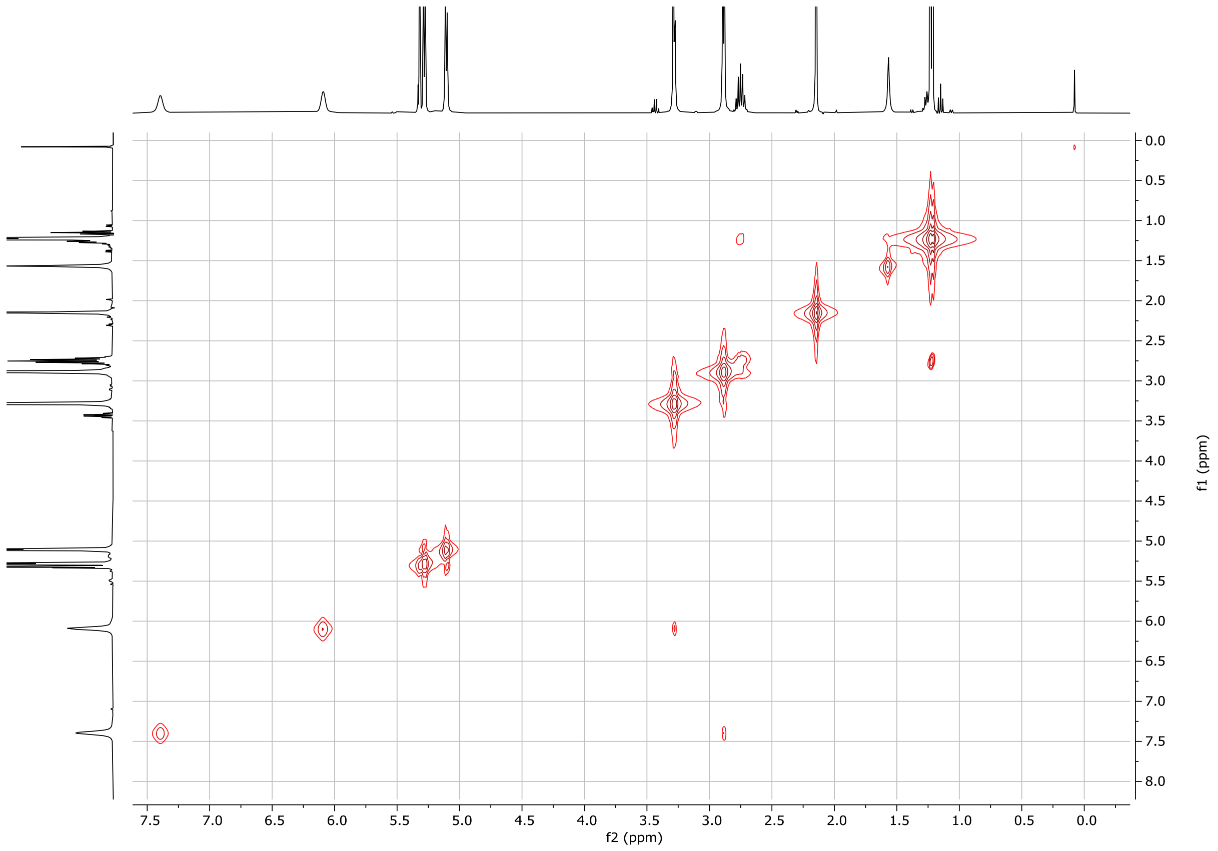

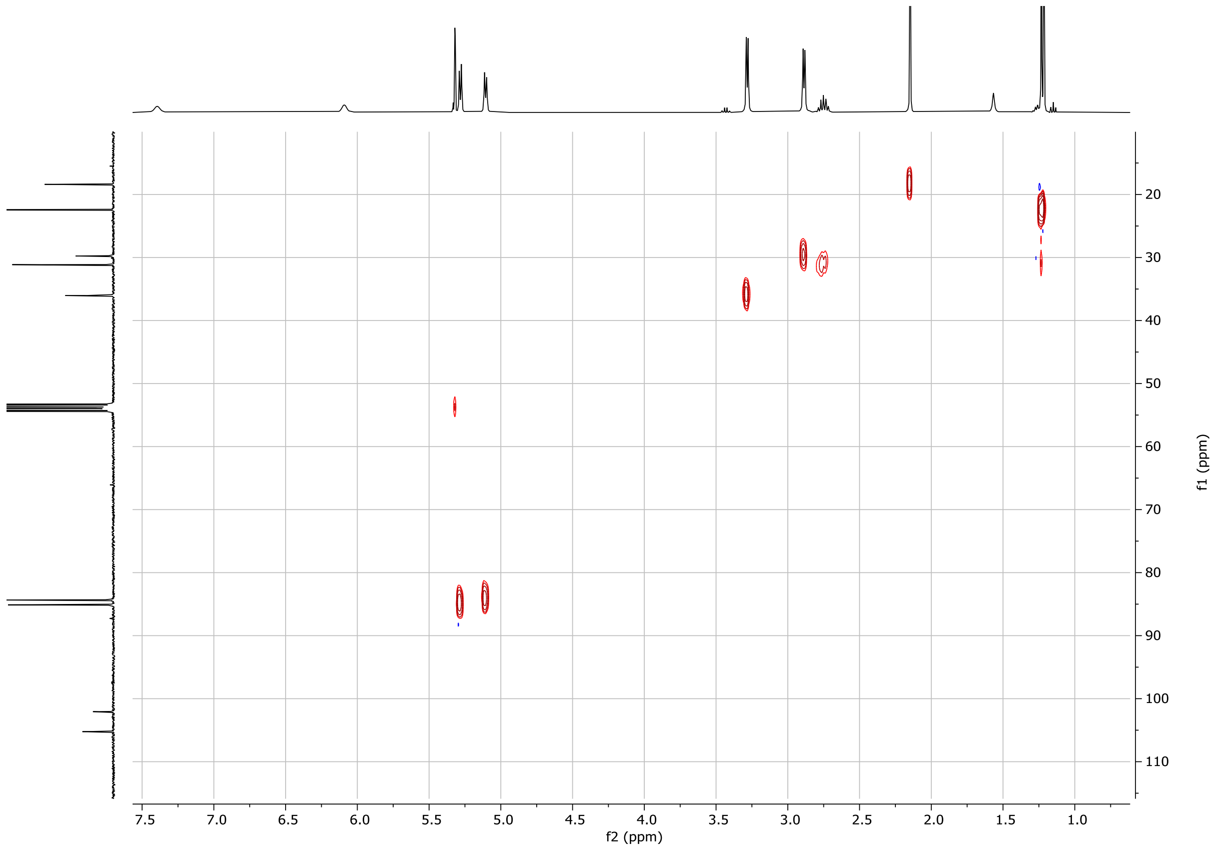

Figure S28:^{1}H-^{1}H-COSY (top) and ^{1}H-^{13}C-HSQC (bottom) spectrum (CD_2Cl_2, 298 K, 400 MHz) of **Ru-6**.

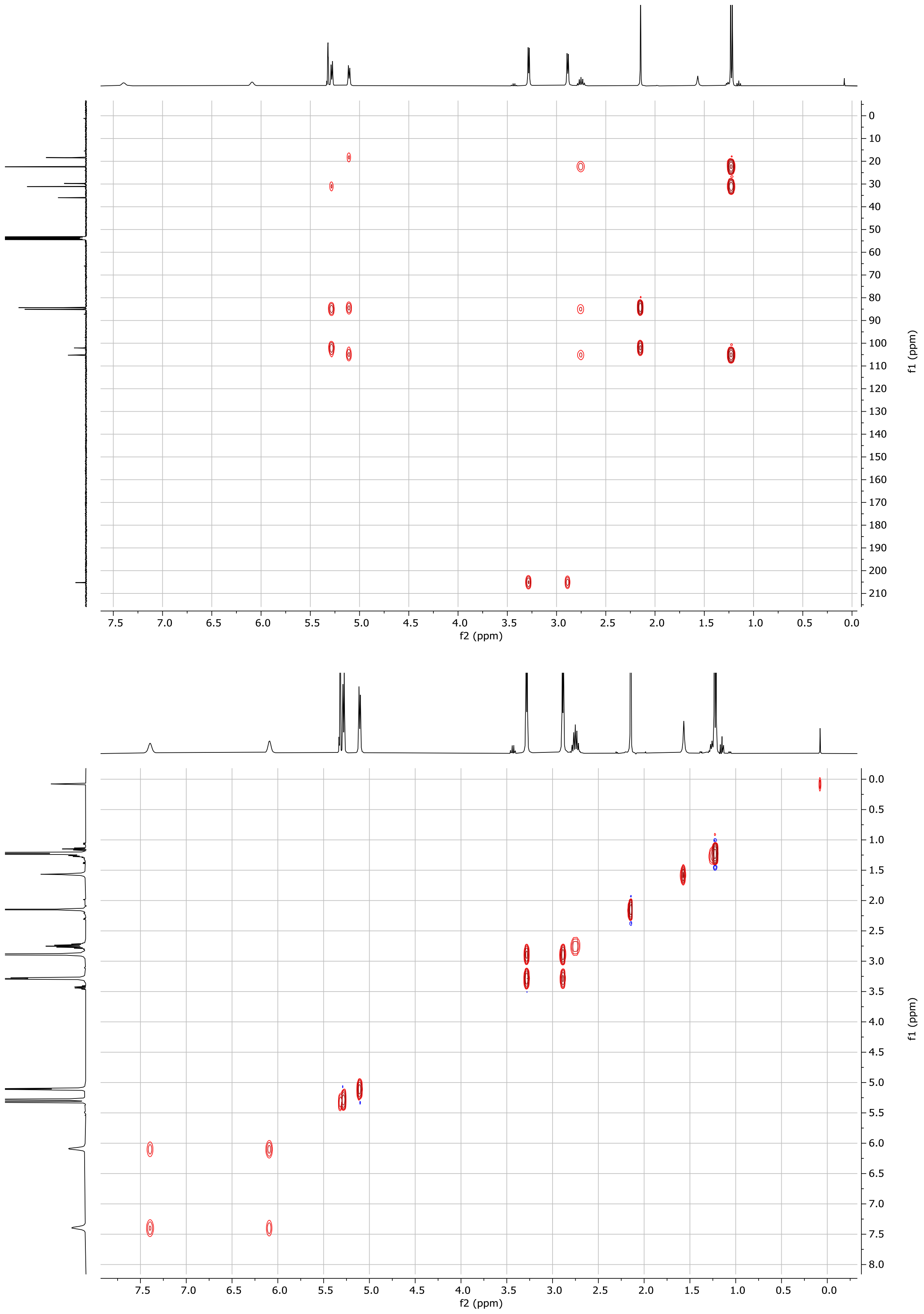

Figure S29: ^{1}H-^{13}C-HMBC (top) and ^{1}H-^{1}H-NOESY (bottom) spectrum (CD_2Cl_2, 298 K, 400 MHz) of **Ru-6**.

Variable-Temperature NMR spectra of Ru-6

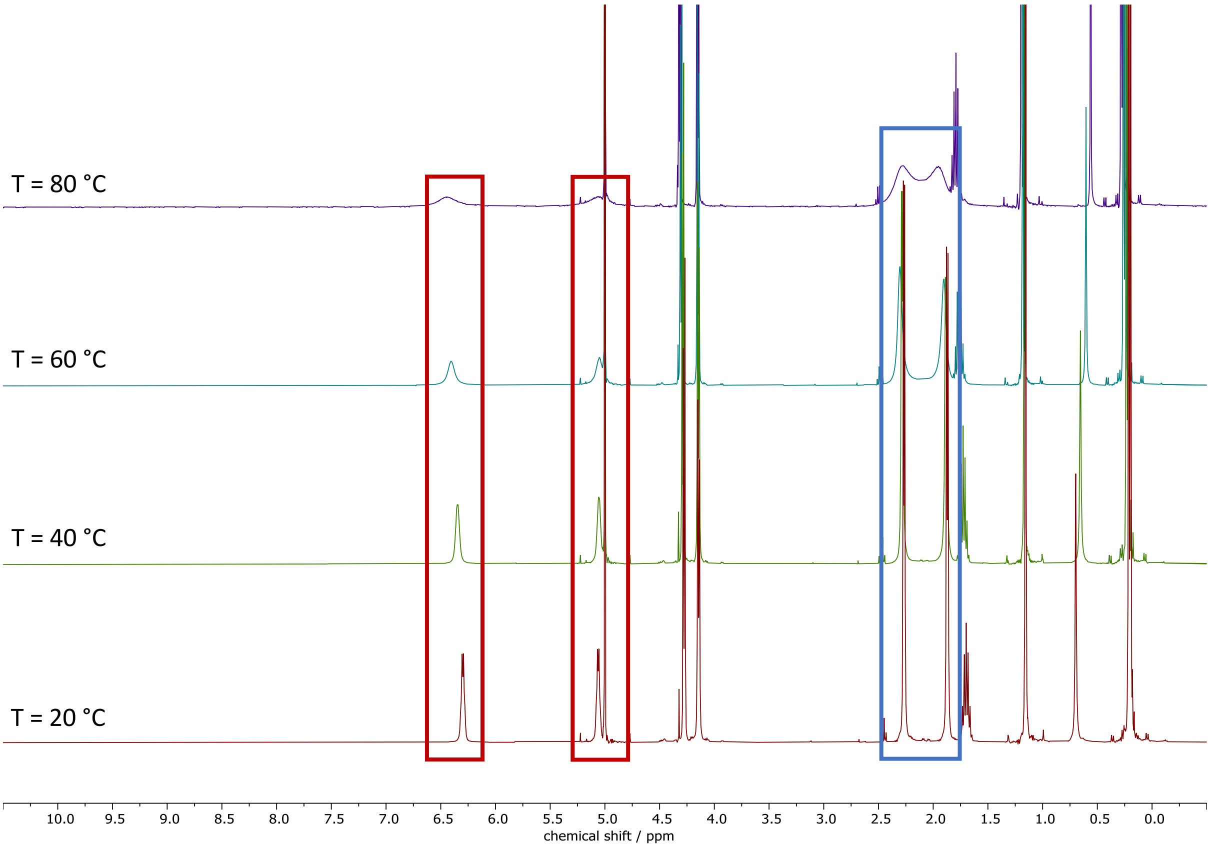

Signal	FWHM / Hz (20 °C)	FWHM / Hz (80 °C)	$FWHM_{80°C}$ / $FWHM_{20°C}$
Ar-$(CH_3)_2$	9	8	1
Ar-CH_3	2	2	1
Ar-CH	16	1	1
**N-CH_3**	**8**	**205**	**26**
**N-CH_3**	**7**	**217**	**29**
Ar-H	9	8	1
Ar-H	8	8	1
**N-H**	**13**	**131**	**10**
**N-H**	**13**	**101**	**8**

Figure S30: Top panel shows ^{1}H NMR spectrum (298 K–353 K, 400 MHz) of **Ru-6** in in $C_2D_2Cl_4$. The N–H and N–CH_3 resonances are highlighted in red and blue, respectively, with considerable line broadening pointing to exchange dynamics. Bottom panel lists the line-shape analysis of the resonances as full width at half maximum at 20 and 80 °C (FWHM20 and FWHM80, respectively), demonstrating the selective line broadening of the N–CH3 and N–H resonances exclusively and indicating that the dynamic behavior is restricted to the ADC ligand.

Solvent dependent NH resonance

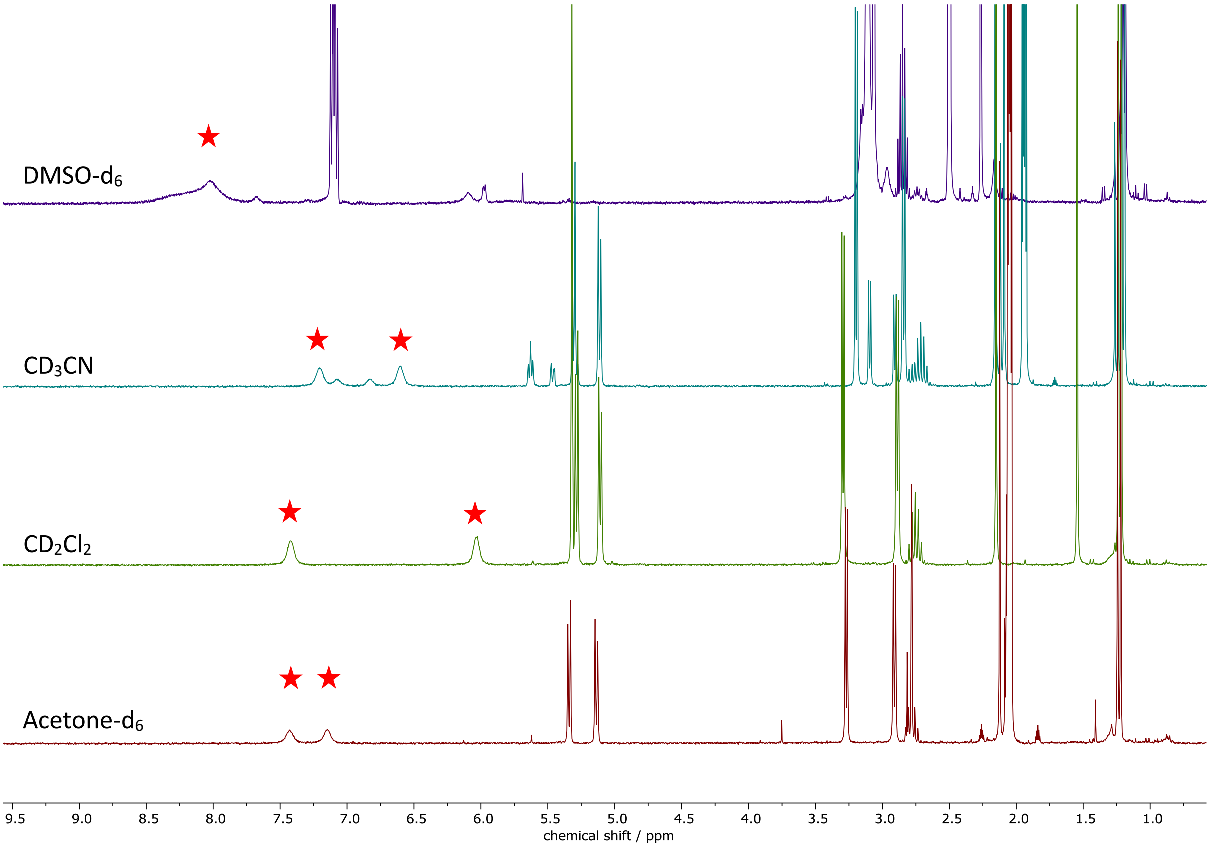

Figure S31: ^{1}H NMR spectrum (298 K, 400 MHz) of **Ru-6** in solvents of different polarity. The N–H resonances are highlighted with a red star.

H/D exchange in Ru-6

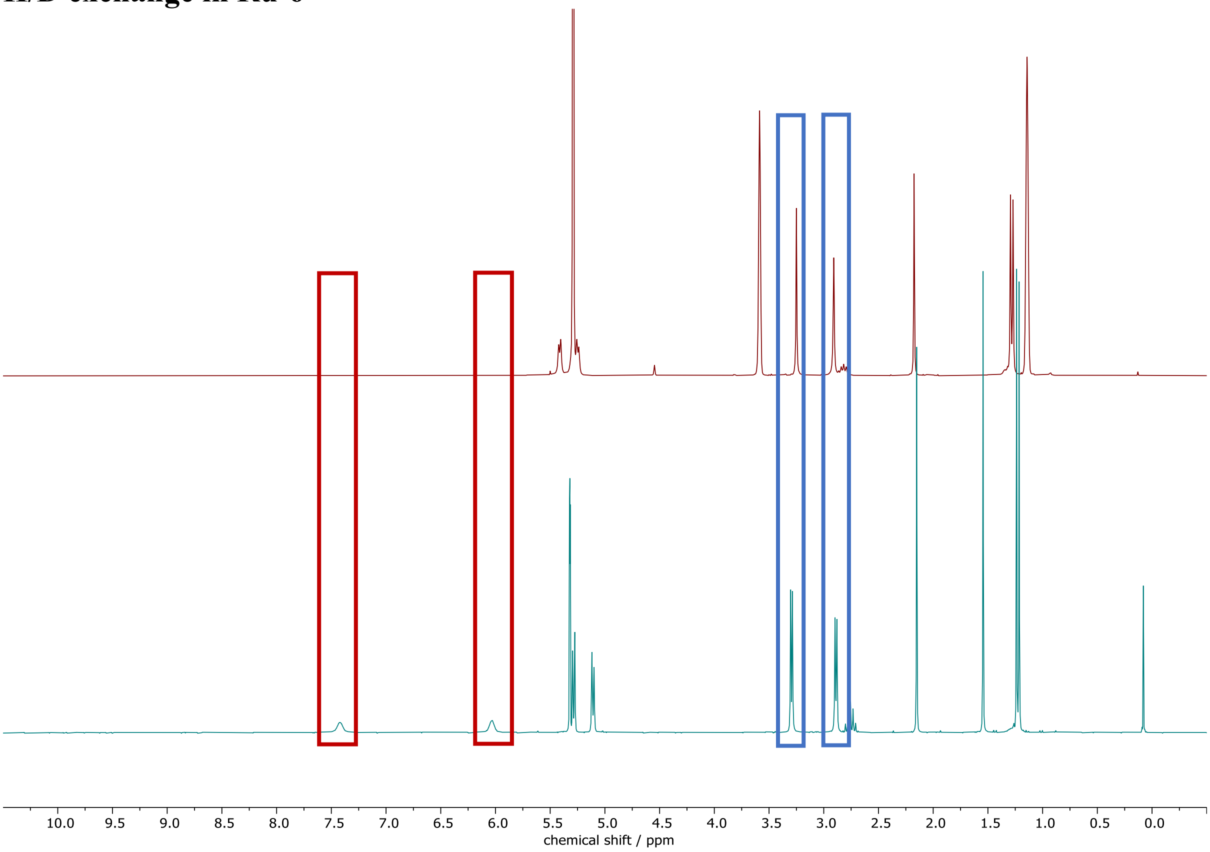

Figure S32: ^{1}H NMR spectrum (298 K, 400 MHz) of **Ru-6** in CD_2Cl_2 (bottom) and in CD_3CD_2OD (top). H/D exchange is highlighted by disappearance of the N-H signal at δ = 7.42 and 6.02 ppm (highlighted in red) and transition of $N\text{-}CH_3$ doublet (δ =3.30 and 2.88 ppm) to a singlet (highlighted in blue).

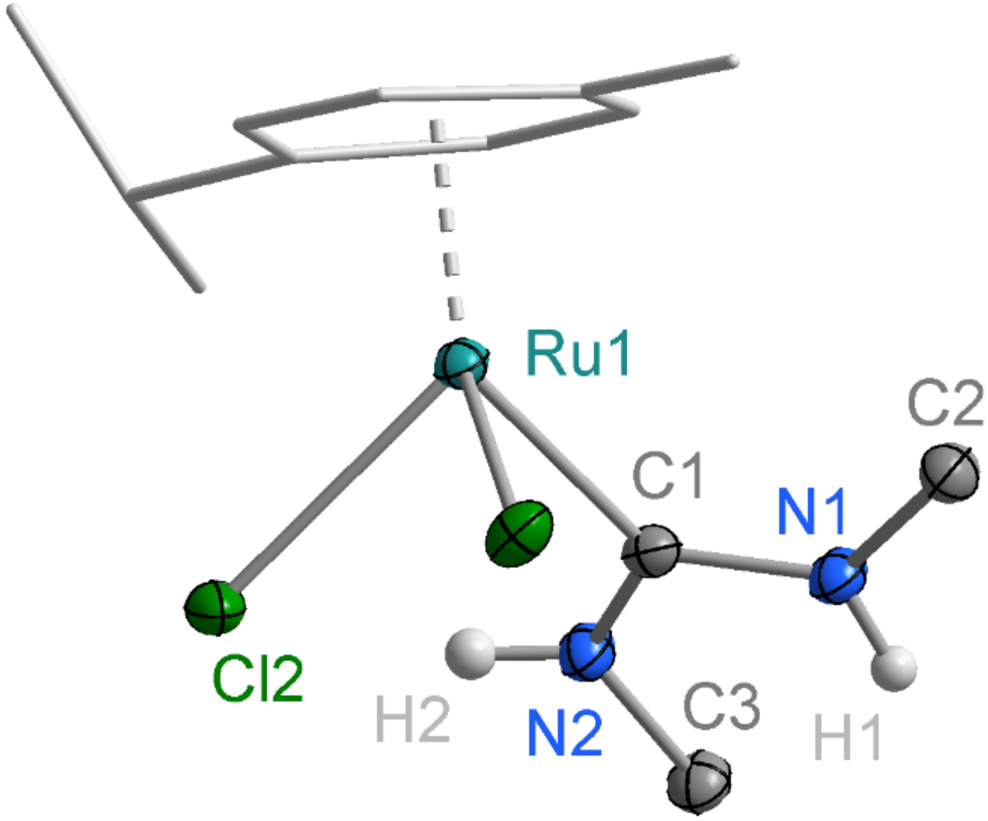

Figure S33: Molecular structure of **Ru-6** with 30% probability displacement ellipsoids. All H atoms (except H1 and H2) have been omitted for clarity.

Complex Interconversion

Ru-2c is converted back to **Ru-2a** by addition of 1.2 eqv of pyrrolidine: **Ru-2c** (20 mg, 0.048 mmol) was added to a J Young NMR tube followed by the addition of d-CD_2Cl_2 (0.5 mL) and pyrrolidine (5 µL, 0.058 mmol). After 15 min a ^{1}H NMR spectrum was recorded as shown in **Figure S34** (top spectrum) indicating the formation of **Ru-2a**.

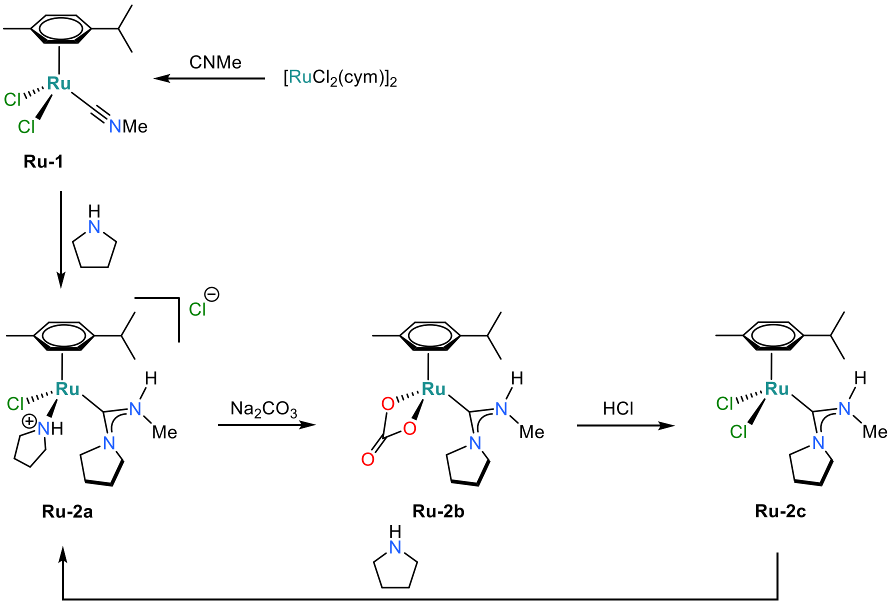

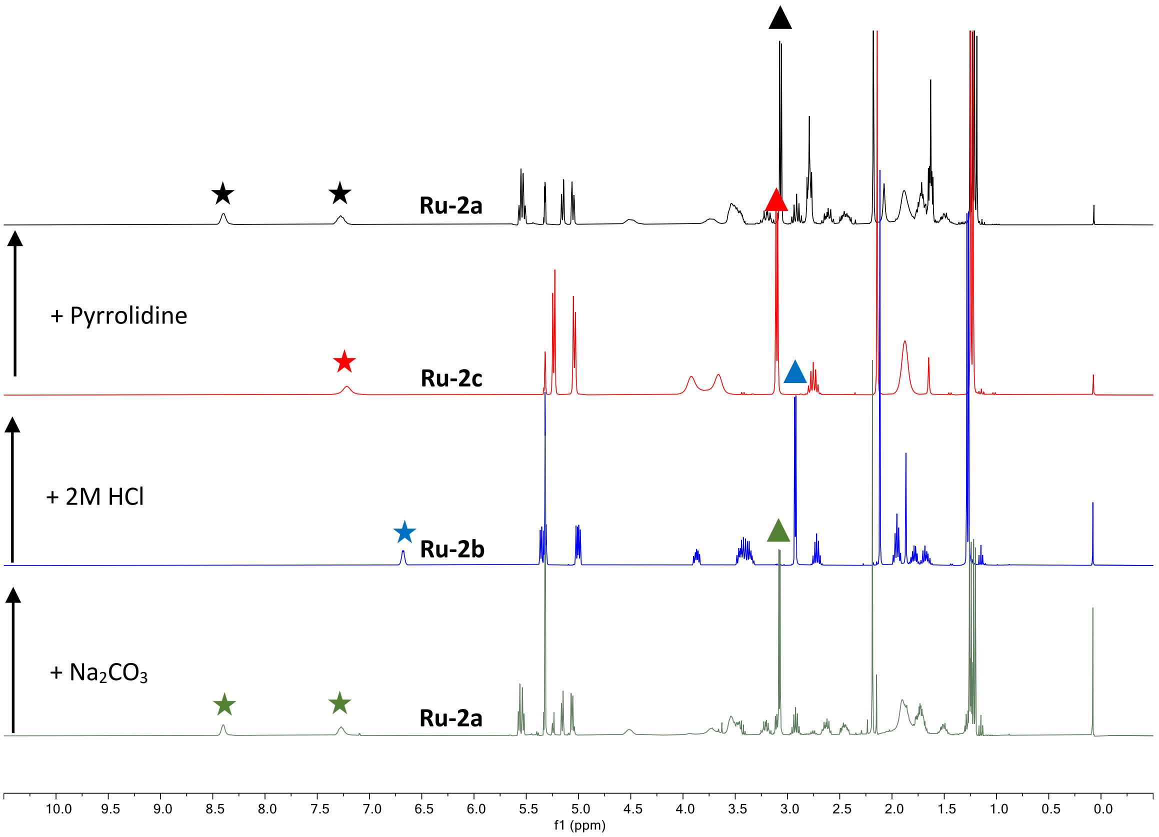

Figure S34: 1H NMR (CD_2Cl_2, 298 K, 400 MHz) spectrum of the interconversion of **Ru-2a** to **Ru-2b** to **Ru-2c** back to **Ru-2a**. Characteristic N-H peaks are highlighted by a star and N-Me doublets with a triangle.

Catalytic Experiments – General Procedure

To a microwave vial equipped with a stirring bar was added a known amount of 1,3,5-trimethoxybenzene (internal standard = TMB, see NMR spectra for detailed information), isopropanol (1.5 mL) and substrate (0.287 mmol). The mixture was degassed with N_2 for 5 minutes. A catalyst solution was prepared by evaporating a CH_2Cl_2 solution of the Ru complex (52 μL, 55 mM stock solution, 2.9 μmol, 1 mol% with respect to substrate) to dryness and re-dissolving it in iPrOH (0.5 mL). This solution was added to the substrate solution, and the vial was placed in an oil bath pre-heated to 85 °C. After indicated times, aliquots (0.15 mL) were taken and filtered over silica. Yield and conversion were determined by ^{1}H NMR spectroscopy with respect to the internal standard or based on starting material consumption and product formation respectively (see next page for details on calculation of conversion and yield).

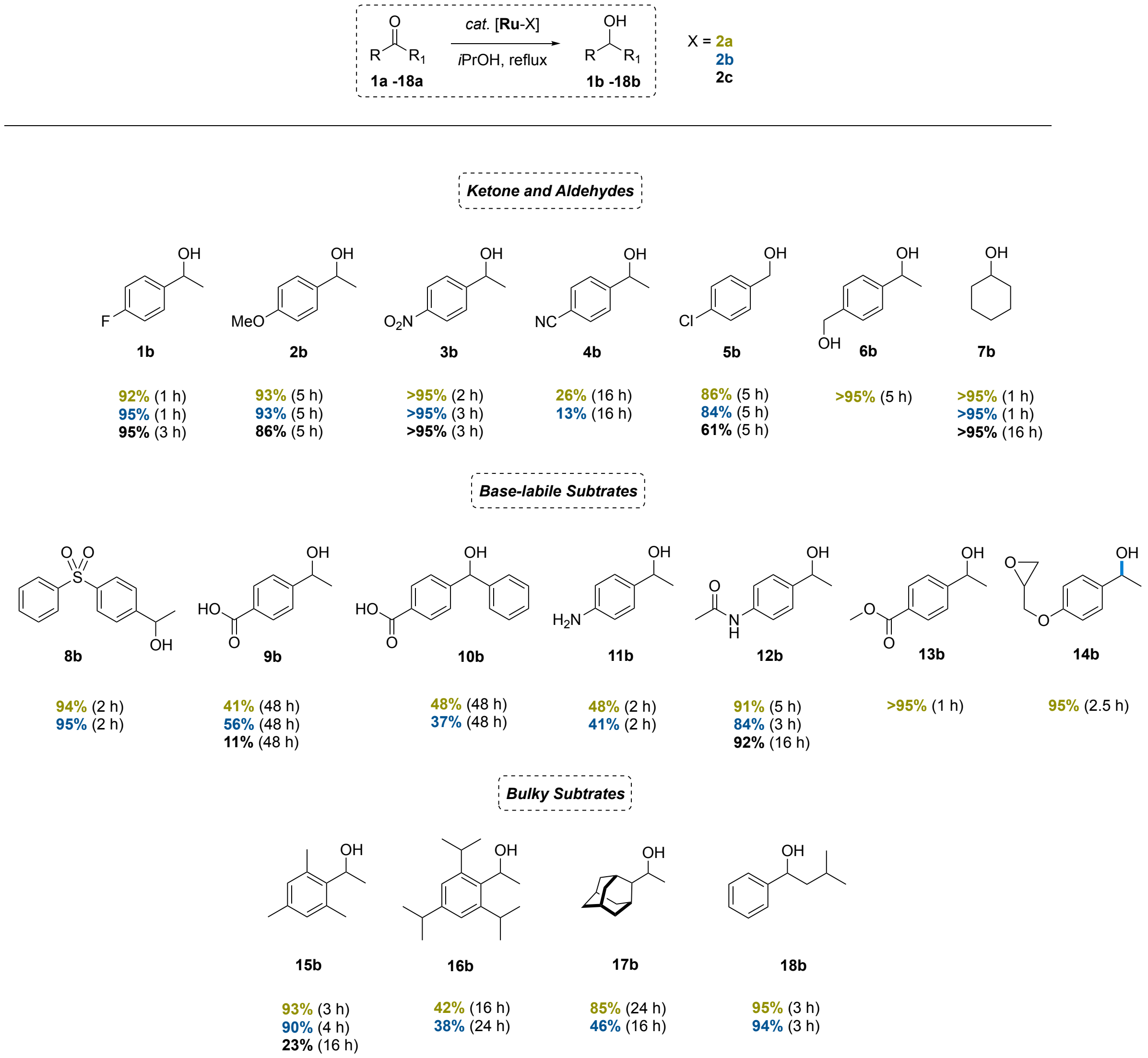

Scheme S1: Substrate scope: Yields of **Ru-2a** catalyzed reactions are shown in olive, **Ru-2b** in blue and **Ru-2c** in black.

Calculation of Conversion and Yield

Yields of the transfer hydrogenation reactions were calculated based on 1,3,5-trimetoxybenzene as internal standard (TMB) as followed: Integration of the starting material (SM) at t = 0, signal (integral set to 1H) resulting in a specific integral value for TMB (in the example in Fig. S35 equal to 0.15H).

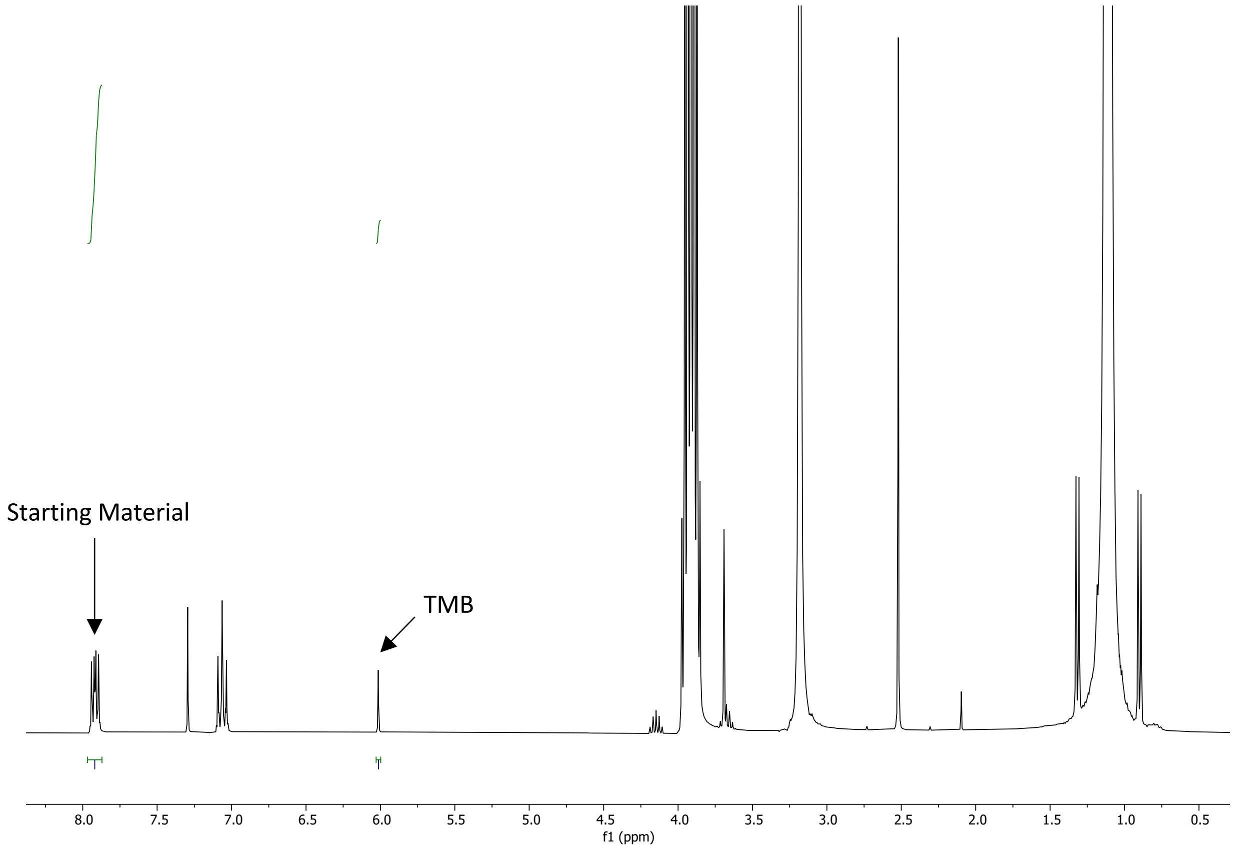

Figure S35: ^{1}H spectrum ($CDCl_3$, 298 K, 400 MHz) at t = 0 for calculating the yield for the reduction of 4-fluoroacetophenone (**1a**) catalyzed by **Ru-2a**.

After a given time, the integral of the TMB was set to the value determined at t = 0 (in this example 0.15H). Integration of the SM and the signals corresponding to the product then show the conversion and yield, respectively. In the example shown in Fig. S36, SM = 0.06 resulting in 94% conversion; product integral = 0.94, resulting in 94% yield).

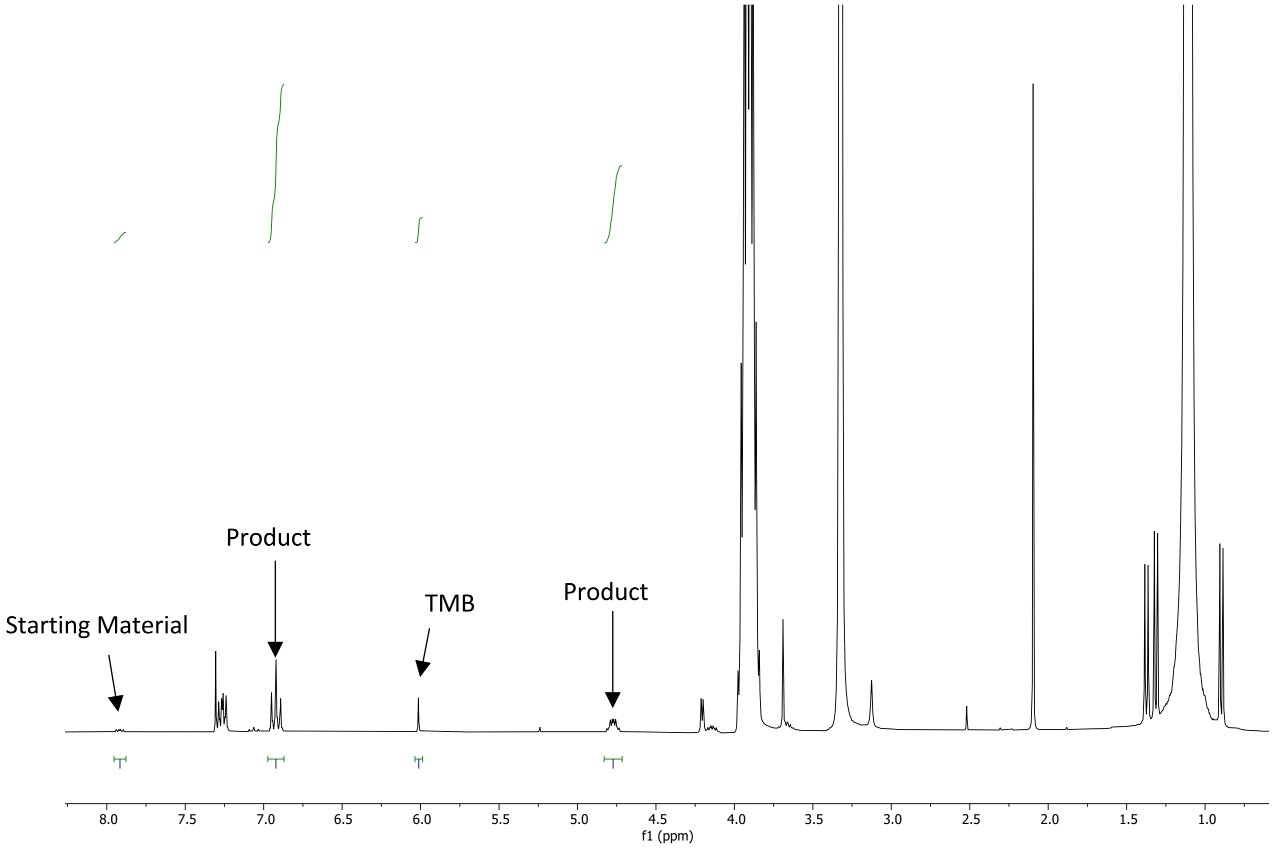

Figure S36: ^{1}H spectrum ($CDCl_3$, 298 K, 400 MHz) at t = 1h for calculating the yield for the reduction of 4'-Fluoroacetophenone catalyzed by **Ru-2a**.

Considering the full selectivity of the reaction, the conversion is equal to yield and can be determined by integrating a signal for the SM at t = 0. At a given time interval, integrating the same signal along with the integration of a resonance representing the product and using the ratio Int(P) / [Int(P) + Int(SM)] gives the conversion and yield.

Reduction of 4-Fluoroacetophenone (1a) – Catalyzed by Ru-2a

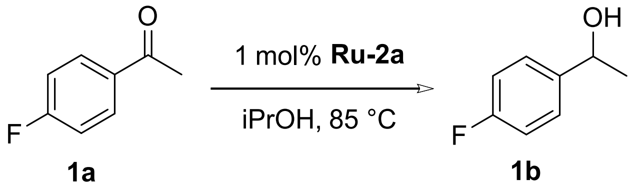

Time / h	Yield / %
0.25	67
0.5	82
1	92
3	>95

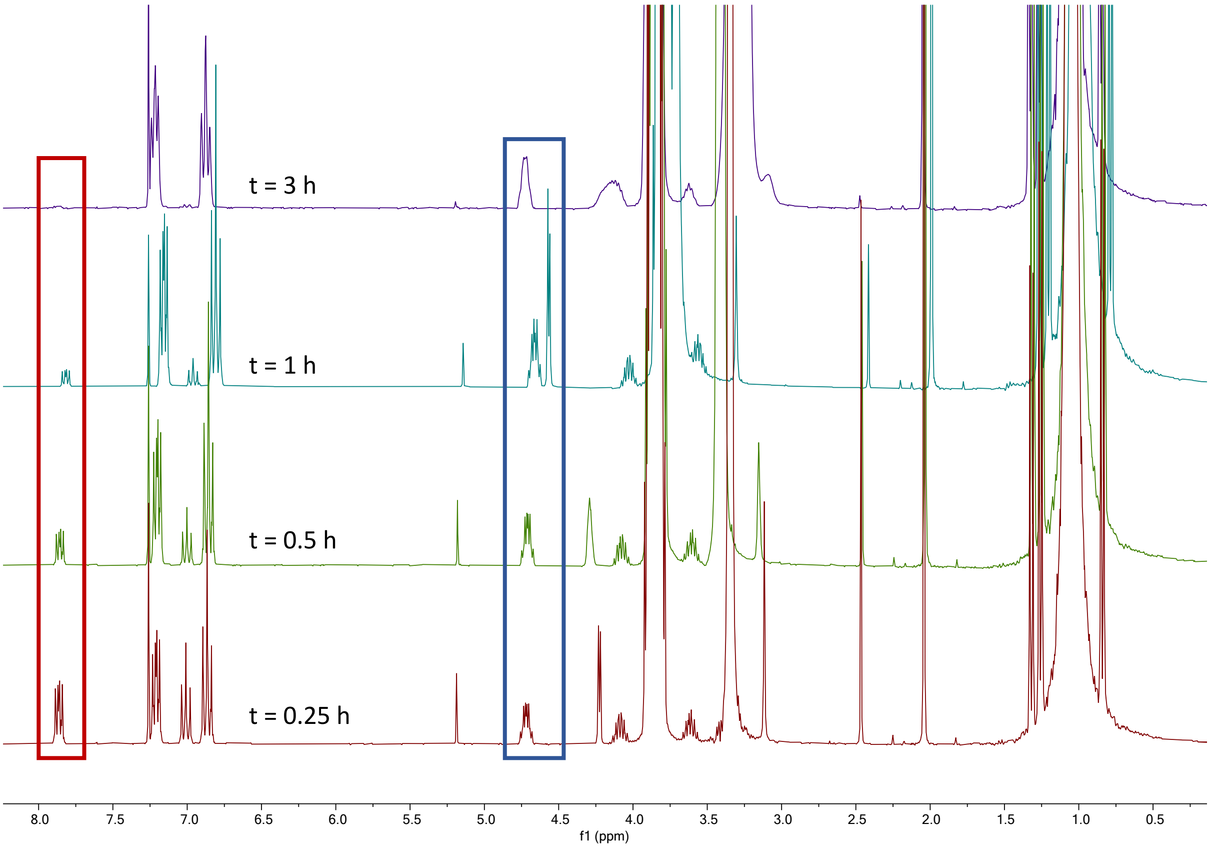

Figure S37: ^{1}H spectrum ($CDCl_3$, 298 K, 400 MHz) from the reduction of (**1a**) mediated by **Ru-2a**. Starting material consumption is highlighted in red and formation of characteristic signal for product formation is highlighted in blue.

Reduction of 4-Fluoroacetophenone (1a) – Catalyzed by Ru-2b

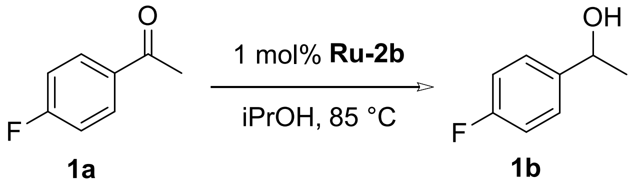

Time / h	Yield / %
0.25	77
0.5	87
1	95
3	>95

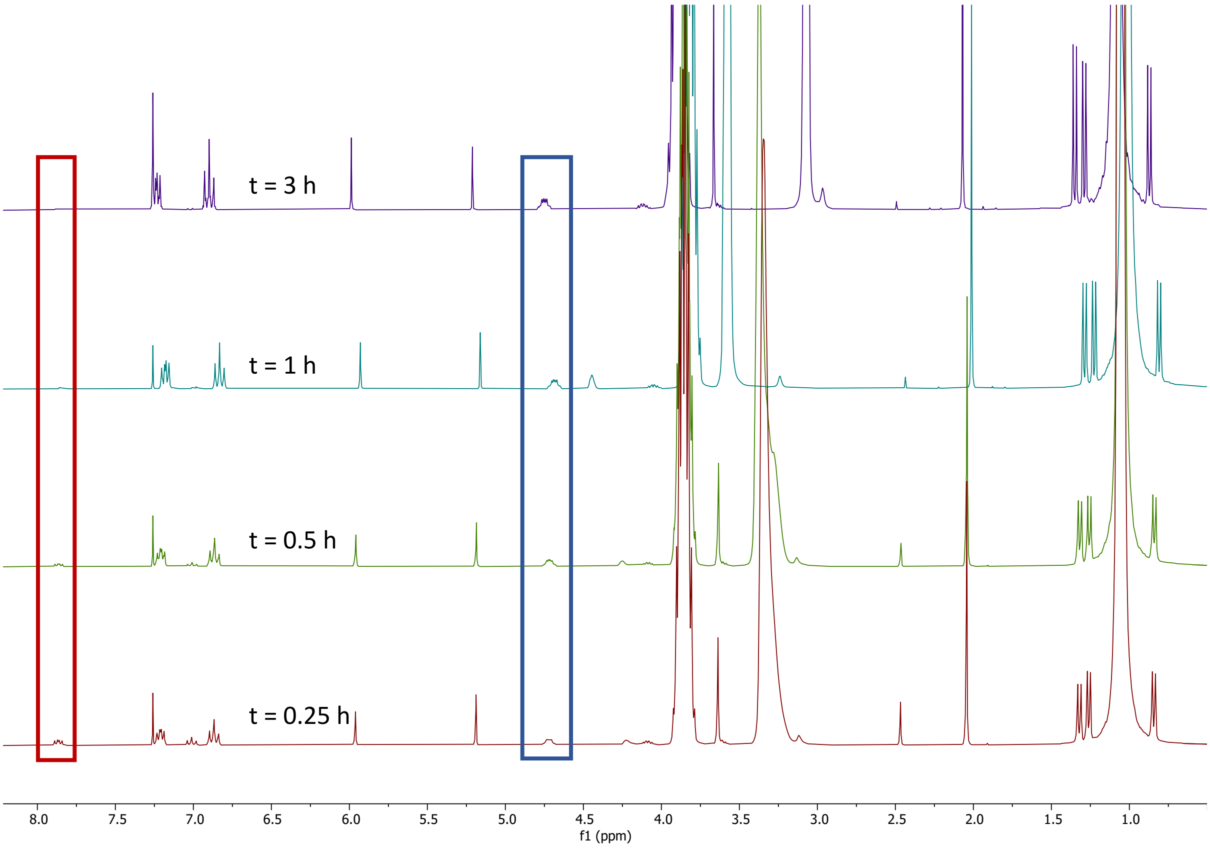

Figure S38: ^{1}H spectrum ($CDCl_3$, 298 K, 400 MHz) of reduction from the (**1a**) mediated by **Ru-2b**. Starting material consumption is highlighted in red and formation of characteristic signal for product formation is highlighted in blue.

Reduction of 4-Fluoroacetophenone (1a) – Catalyzed by Ru-2c

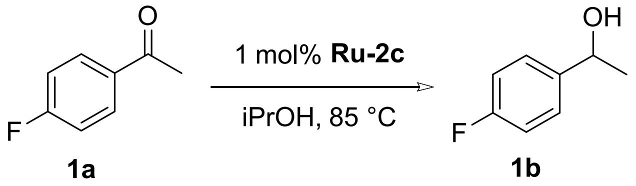

Time / h	Yield / %
0.25	40
0.5	58
1	74
3	>95

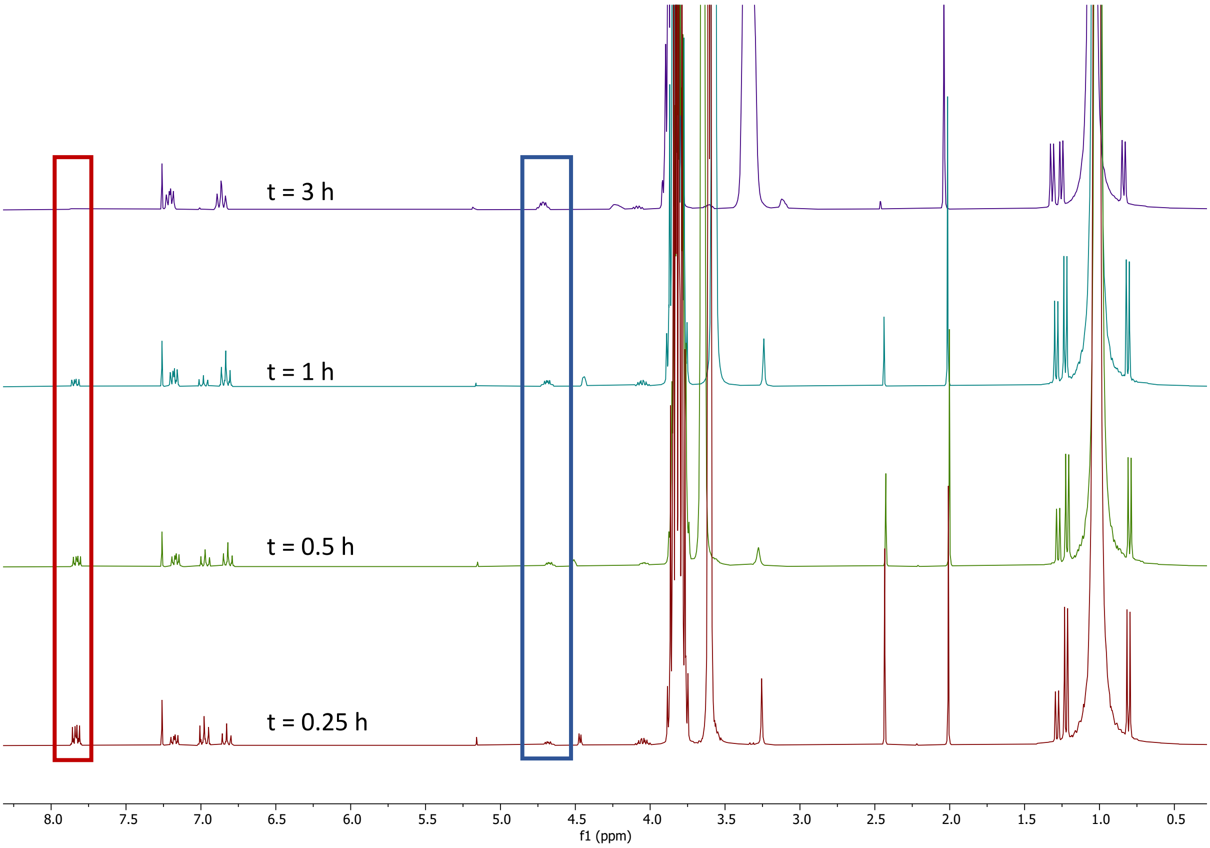

Figure S39: ^{1}H spectrum ($CDCl_3$, 298 K, 400 MHz) of reduction from the (**1a**) mediated by **Ru-2c** Starting material consumption is highlighted in red and formation of characteristic signal for product formation is highlighted in blue.

Reduction of 4-Methoxyacetophenone (2a) – Catalyzed by Ru-2a

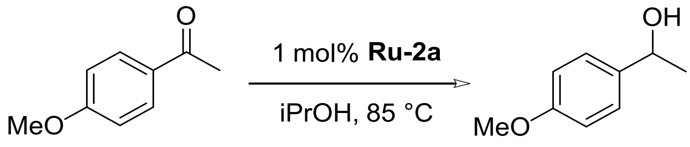

2a **2b**

Time / h	Yield / %
0.5	58
1	80
5	93
16	>95

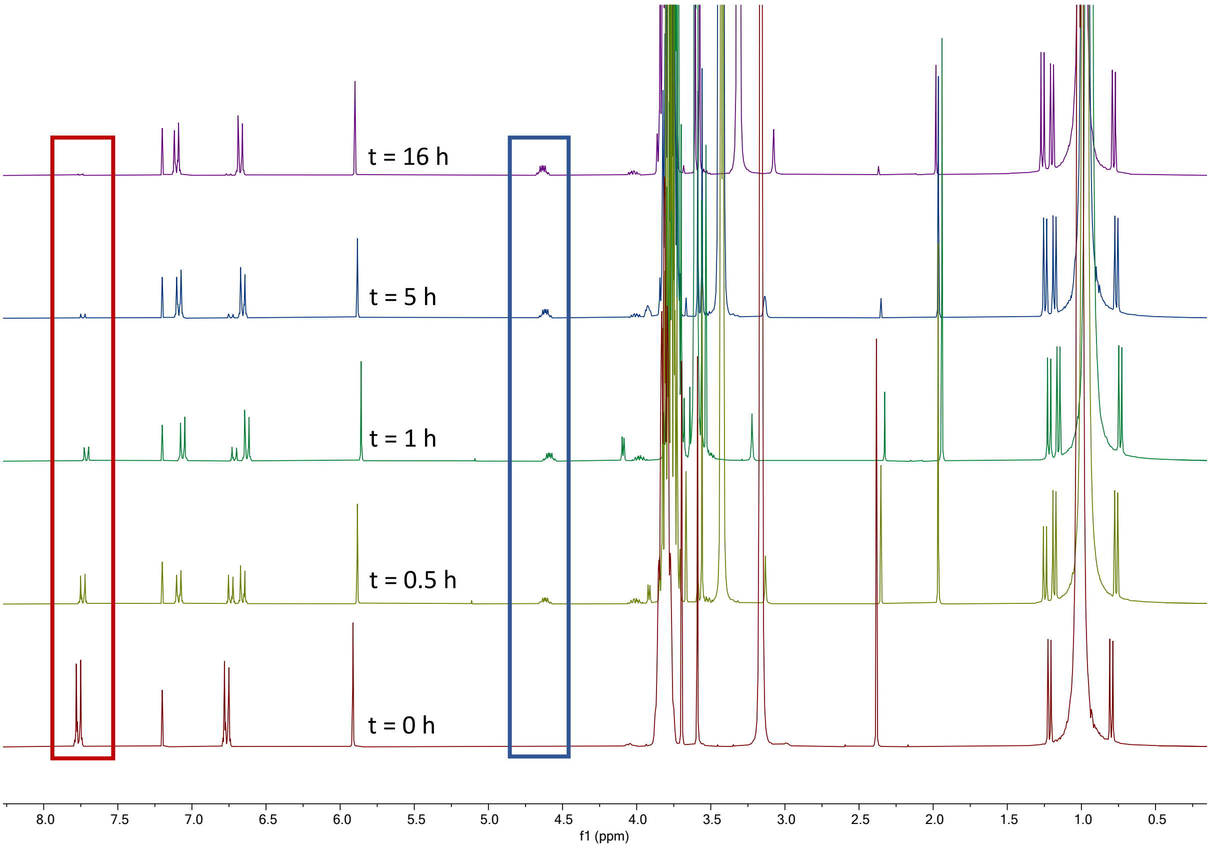

Figure S40: ^{1}H spectrum ($CDCl_3$, 298 K, 400 MHz) from the reduction of (**2a**) mediated by **Ru-2a** (1,3,5-trimethoxybenzene: 16 mg). Starting material consumption is highlighted in red and formation of characteristic signal for product formation is highlighted in blue.

Reduction of 4-Methoxyacetophenone (2a) – Catalyzed by Ru-2b

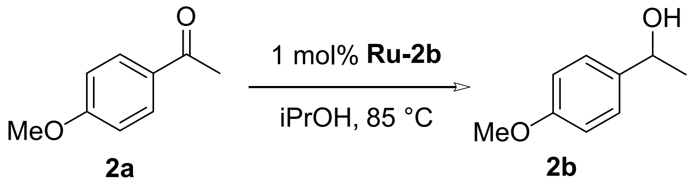

Time / h	Yield / %
1	90
3	92
5	93

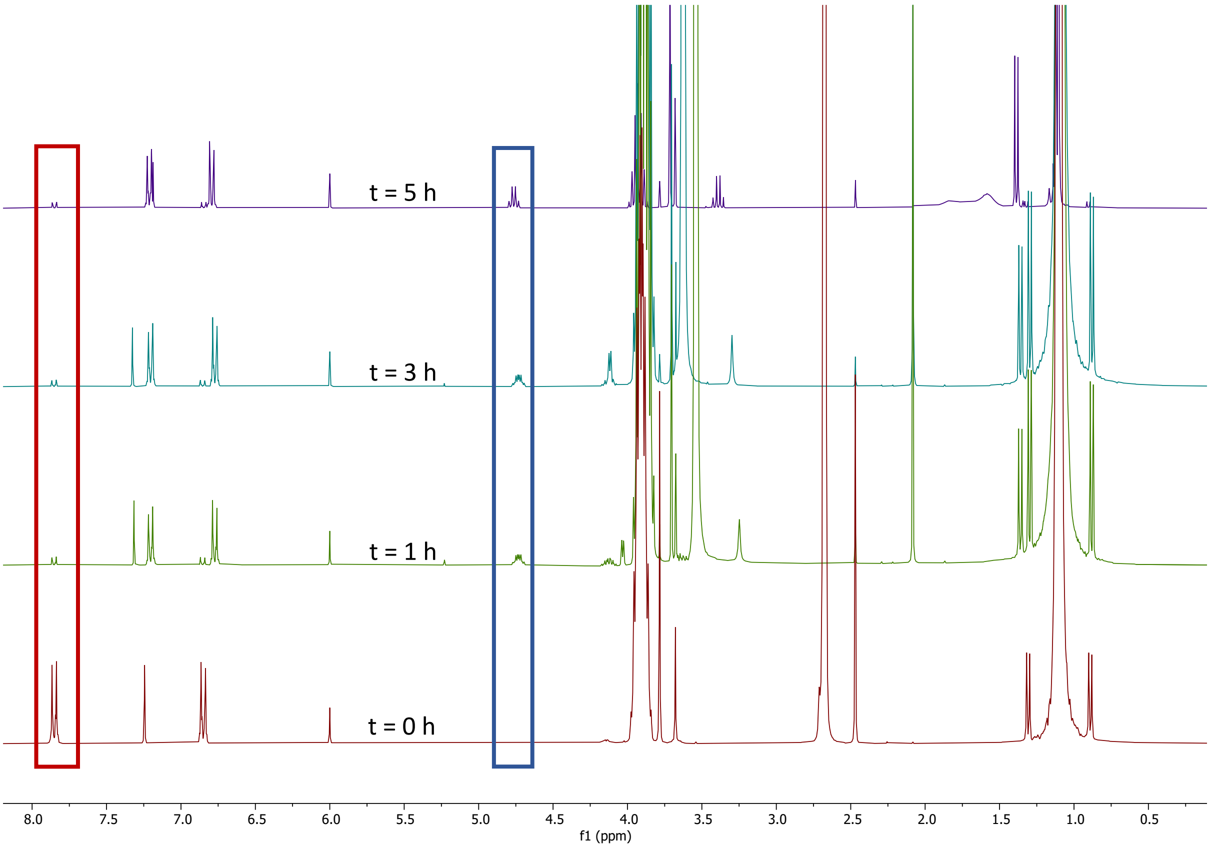

Figure S41: ^{1}H spectrum ($CDCl_3$, 298 K, 400 MHz) from the reduction of (**2a**) mediated by **Ru-2b** (1,3,5-trimethoxybenzene: 9 mg). Starting material consumption is highlighted in red and formation of characteristic signal for product formation is highlighted in blue.

Reduction of 4-Methoxyacetophenone (2a) – Catalyzed by Ru-2c

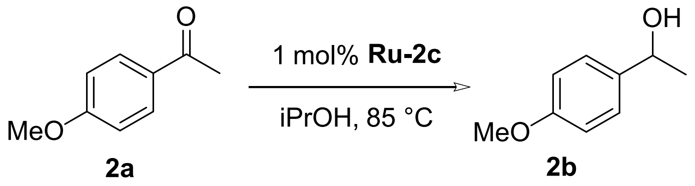

Time / h	Yield / %
1	43
3	78
5	86

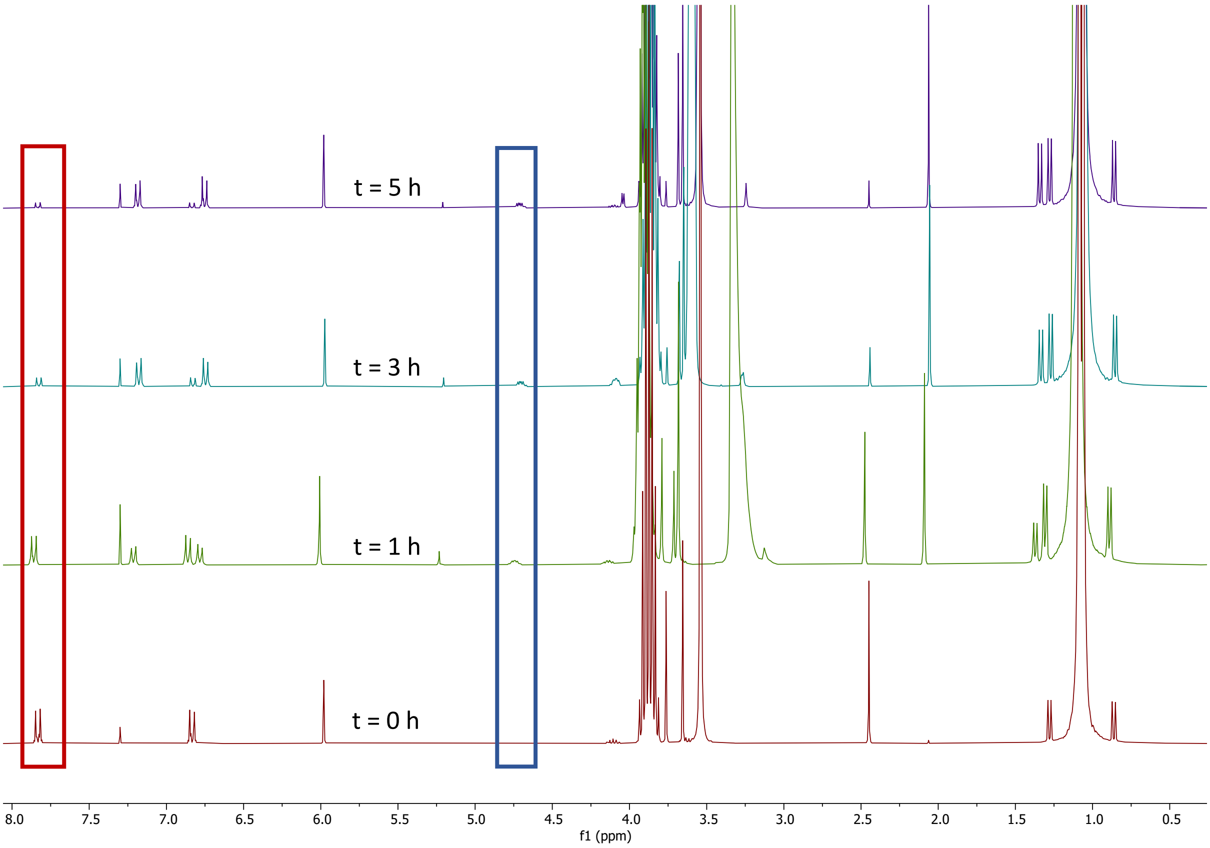

Figure S42: ^{1}H spectrum ($CDCl_3$, 298 K, 400 MHz) from the reduction of (**2a**) mediated by **Ru-2c** (1,3,5-trimethoxybenzene: 20 mg). Starting material consumption is highlighted red and formation of characteristic signal for product formation is highlighted in blue.

Reduction of 4-Nitroacetophenone (3a) – Catalyzed by Ru-2a

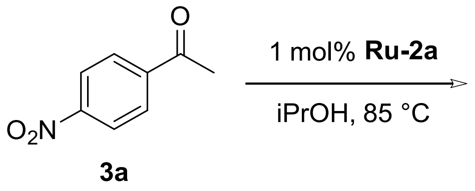

Time / h	Yield / %
0.5	56
1	80
2	>95

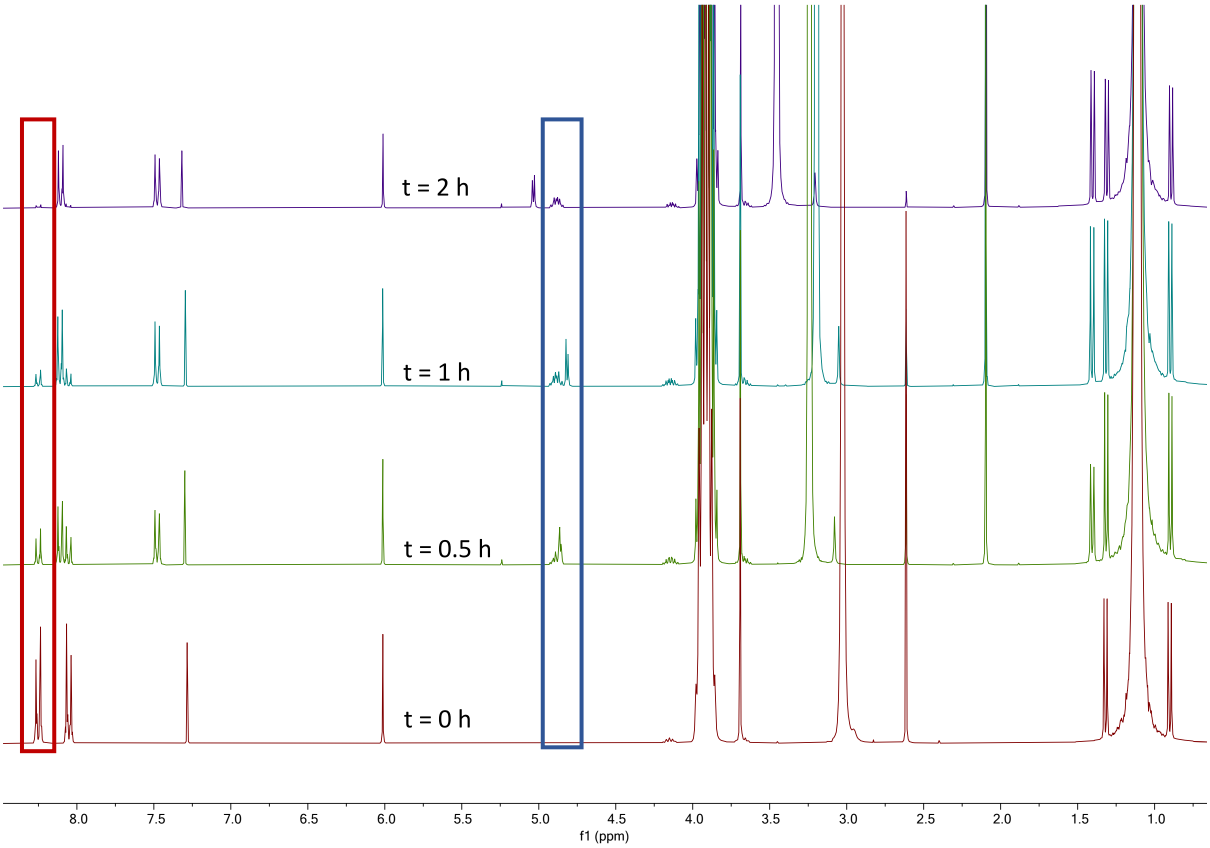

Figure S43: ^{1}H spectrum ($CDCl_3$, 298 K, 400 MHz) from the reduction of (**3a**) mediated by **Ru-2a** (1,3,5-trimethoxybenzene: 11 mg). Starting material consumption is highlighted in red and formation of characteristic signal for product formation is highlighted in blue.

Reduction of 4-Nitroacetophenone (3a) – Catalyzed by Ru-2b

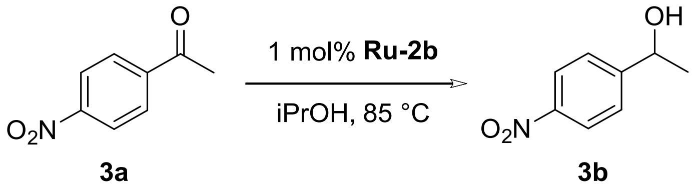

Time / h	Yield / %
1	89
1.5	91
2.5	>95

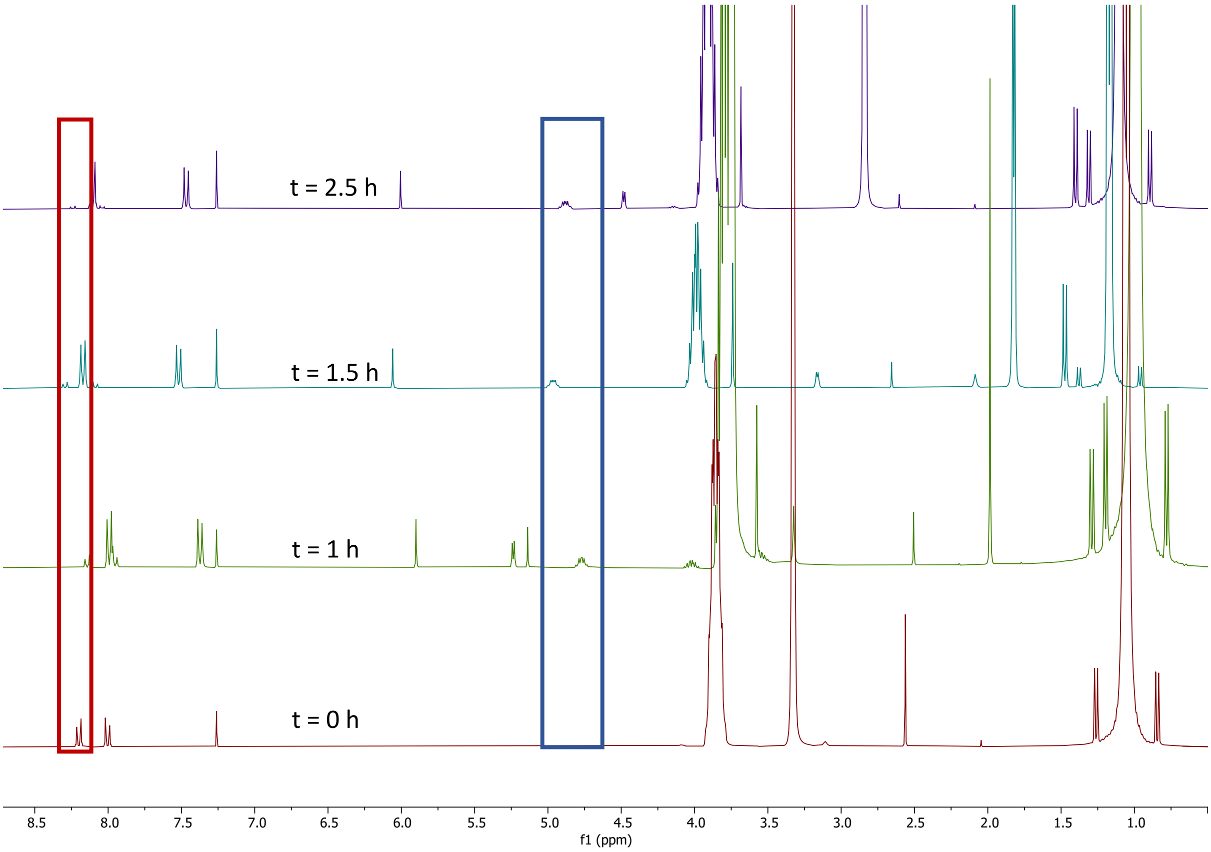

Figure S44: ^{1}H spectrum ($CDCl_3$, 298 K, 400 MHz) from the reduction of (**3a**) mediated by **Ru-2b** (1,3,5-trimethoxybenzene: 12 mg). Starting material consumption is highlighted red and formation of characteristic signal for product formation is highlighted in blue.

HR-MS Calcd. for $C_8H_8O_3N$ [M–H]$^-$ 166.0499. Found: 166.0501.

Reduction of 4-Nitroacetophenone (3a) – Catalyzed by Ru-2c

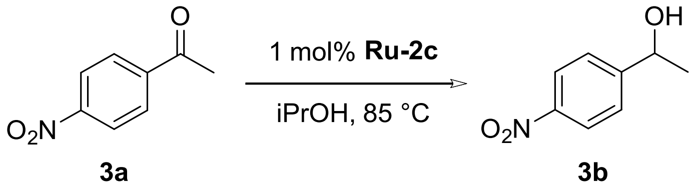

Time / h	Yield / %
1	88
3	>95

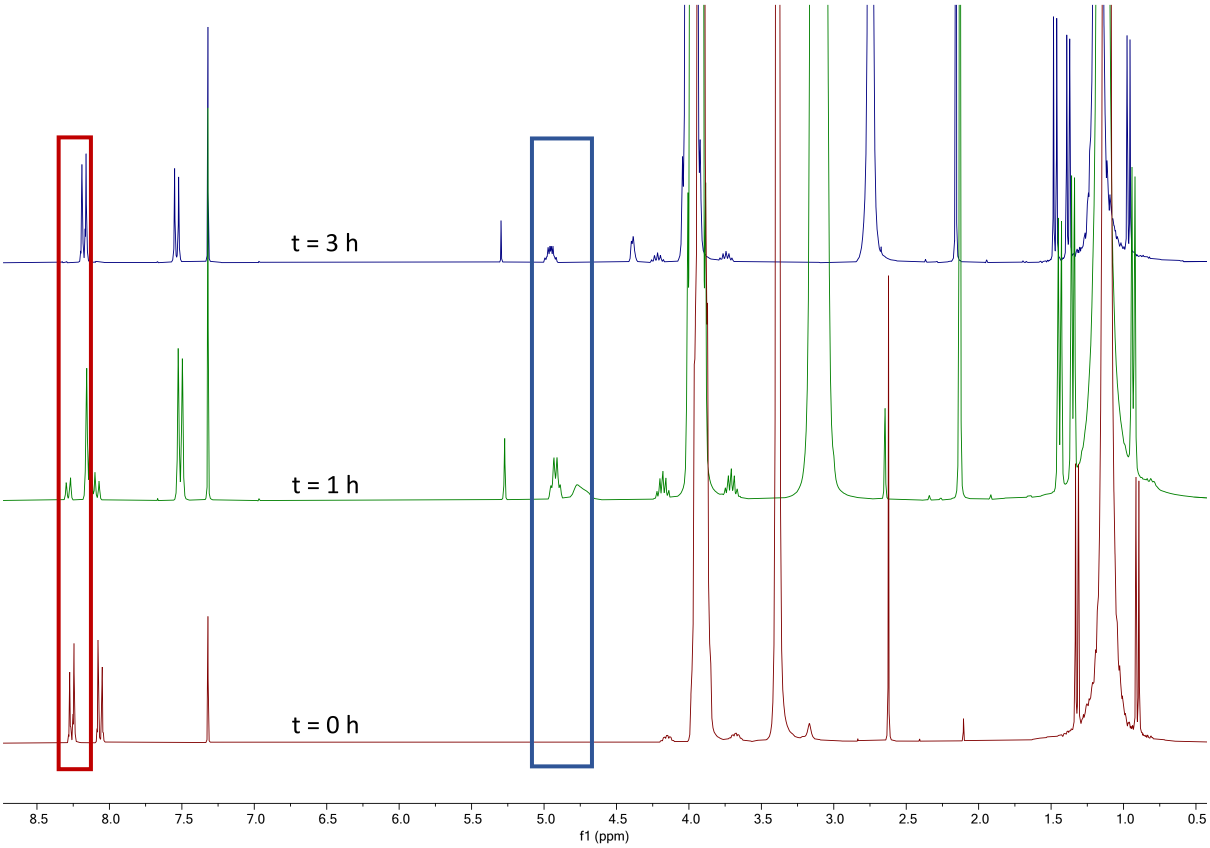

Figure S45: ^{1}H spectrum ($CDCl_3$, 298 K, 400 MHz) from the reduction of (**3a**) mediated by **Ru-2c**. Starting material consumption is highlighted in red and formation of characteristic signal for product formation is highlighted in blue.

Reduction of 4-Acetylbenzonitrile (4a) – Catalyzed by Ru-2a

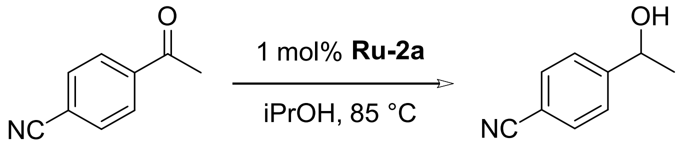

4a **4b**

Time / h	Yield / %
1	2
5	14
16	26

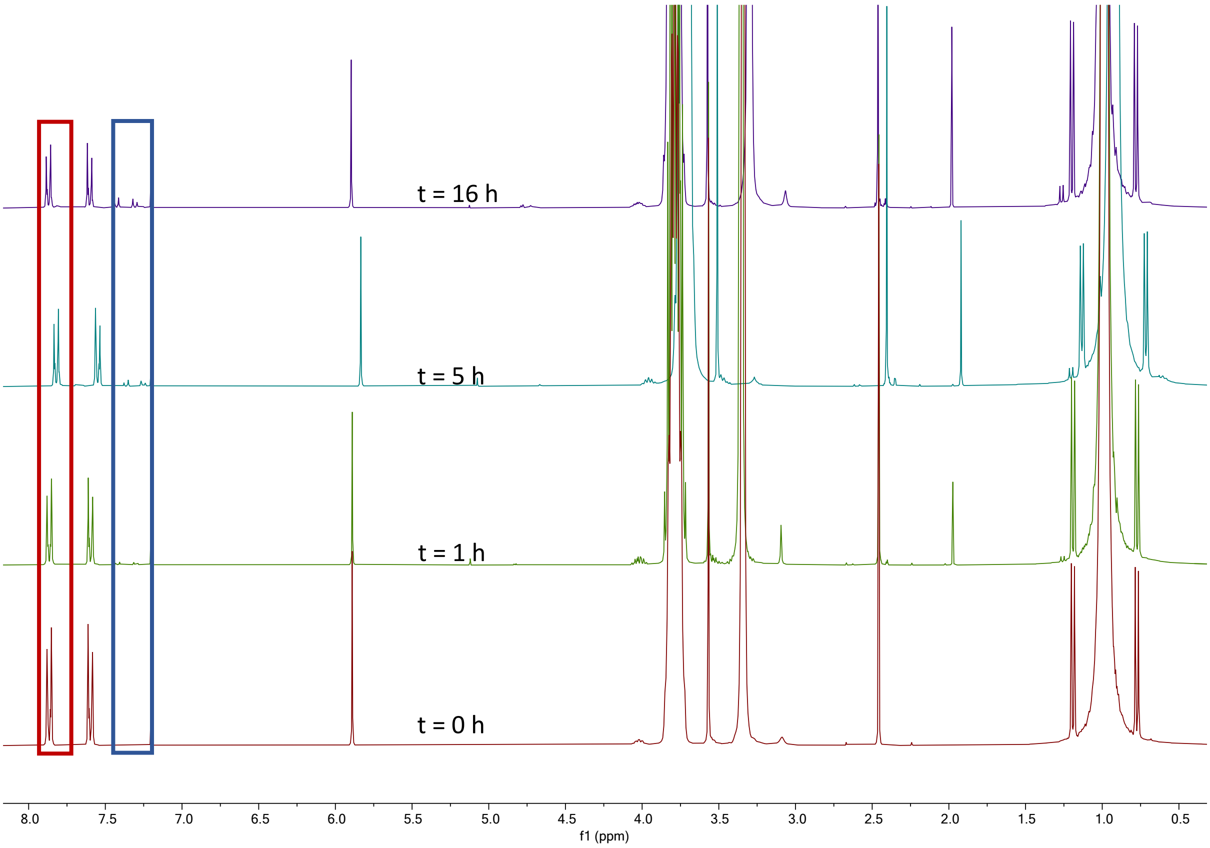

Figure S46: ^{1}H spectrum ($CDCl_3$, 298 K, 400 MHz) from the reduction of (**4a**) mediated by **Ru-2a** (1,3,5-trimethoxybenzene: 18 mg). Starting material consumption is highlighted in red and formation of characteristic signal for product formation is highlighted in blue.

Reduction of 4-Acetylbenzonitrile (4a) – Catalyzed by Ru-2b

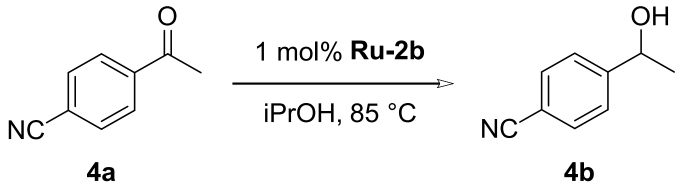

Time / h	Yield / %
1	7
2	9
16	13

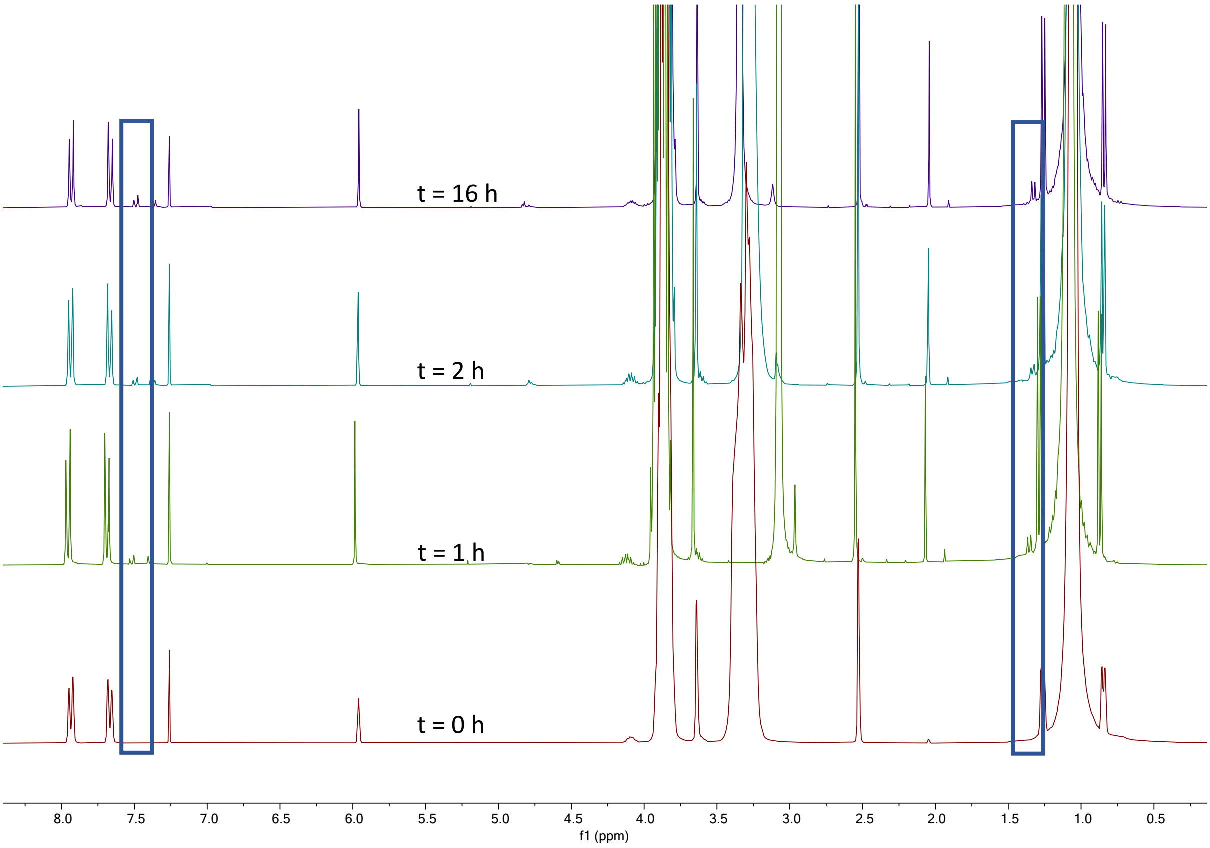

Figure S47: ^{1}H spectrum ($CDCl_3$, 298 K, 400 MHz) from the reduction of (**4a**) mediated by **Ru-2b** (1,3,5-trimethoxybenzene: 10 mg). Formation of characteristic signal for product formation is highlighted in blue.

Reduction of 4-chlorobenzaldehyde (5a) – Catalyzed by Ru-2a

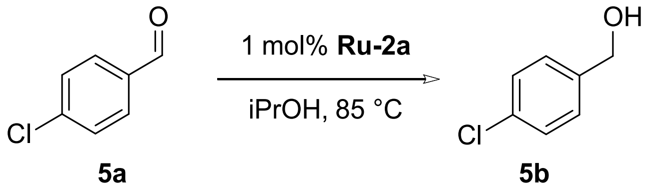

Time / h	Yield / %
1	29
3	69
5	86
16	>95

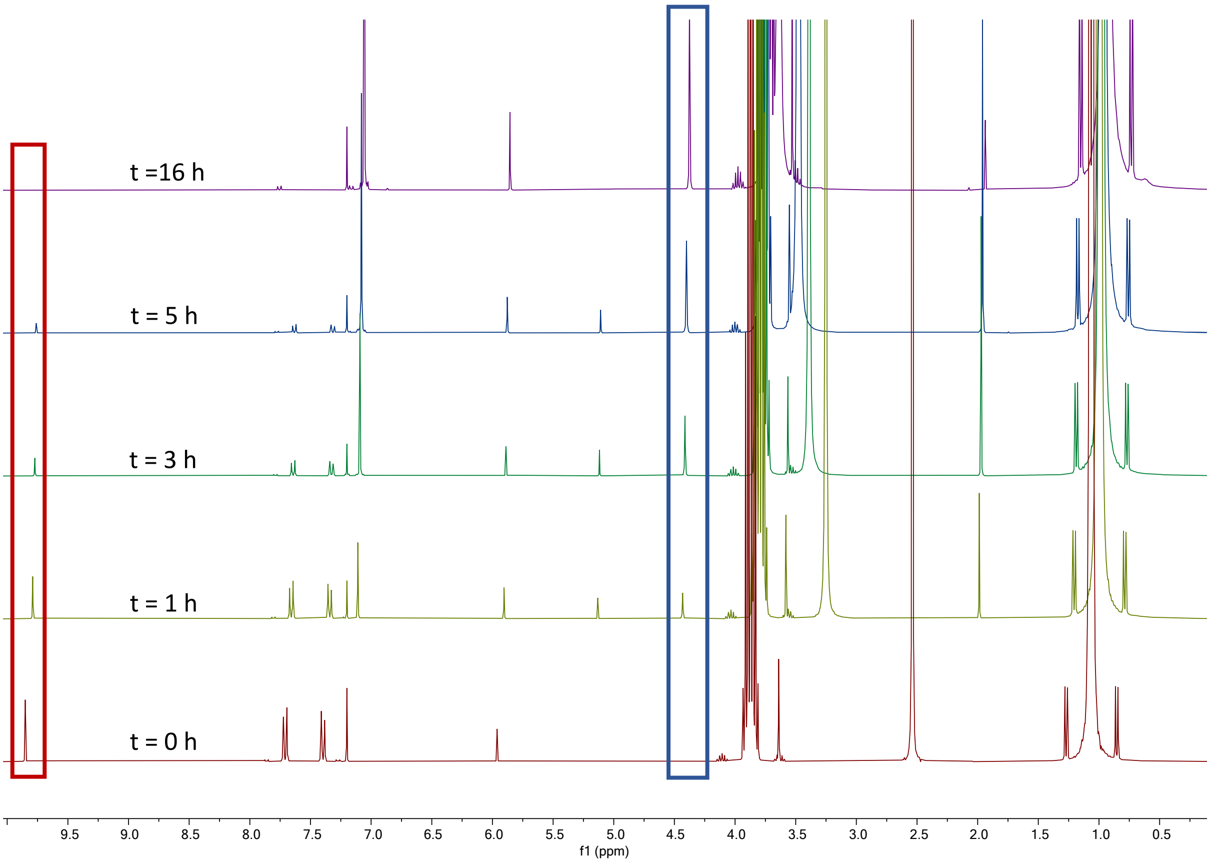

Figure S48: ^{1}H spectrum ($CDCl_3$, 298 K, 400 MHz) from the reduction of (**5a**) mediated by **Ru-2a** (1,3,5-trimethoxybenzene: 10 mg). Starting material consumption is highlighted in red and formation of characteristic signal for product formation is highlighted in blue.

Reduction of 4-chlorobenzaldehyde (5a) – Catalyzed by Ru-2b

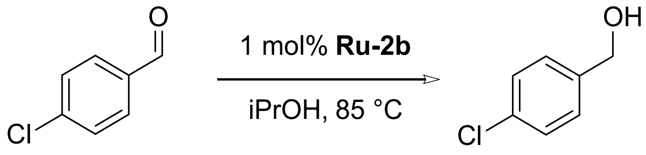

5a **5b**

Time / h	Yield / %
1	48
3	68
5	84

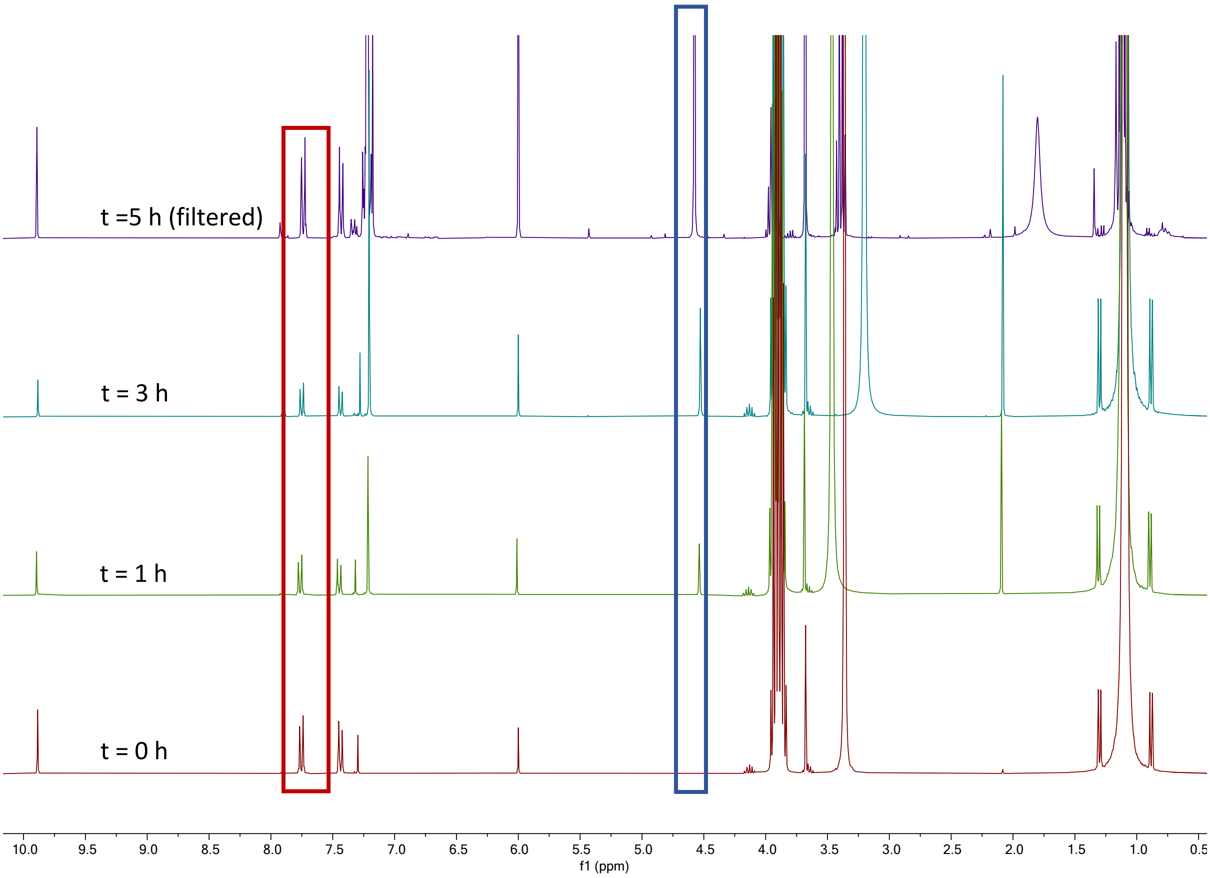

Figure S49: ^{1}H spectrum ($CDCl_3$, 298 K, 400 MHz) from the reduction of (**5a**) mediated by **Ru-2b** (1,3,5-trimethoxybenzene: 10 mg). Starting material consumption is highlighted red and formation of characteristic signal for product formation is highlighted in blue.

Reduction of 4-chlorobenzaldehyde (5a) – Catalyzed by Ru-2c

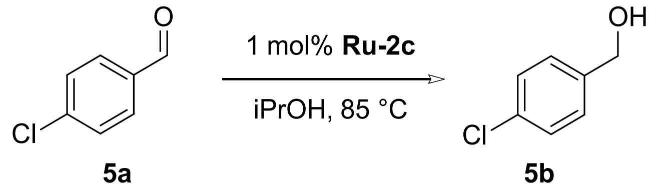

Time / h	Yield / %
1	31
5	61
16	90

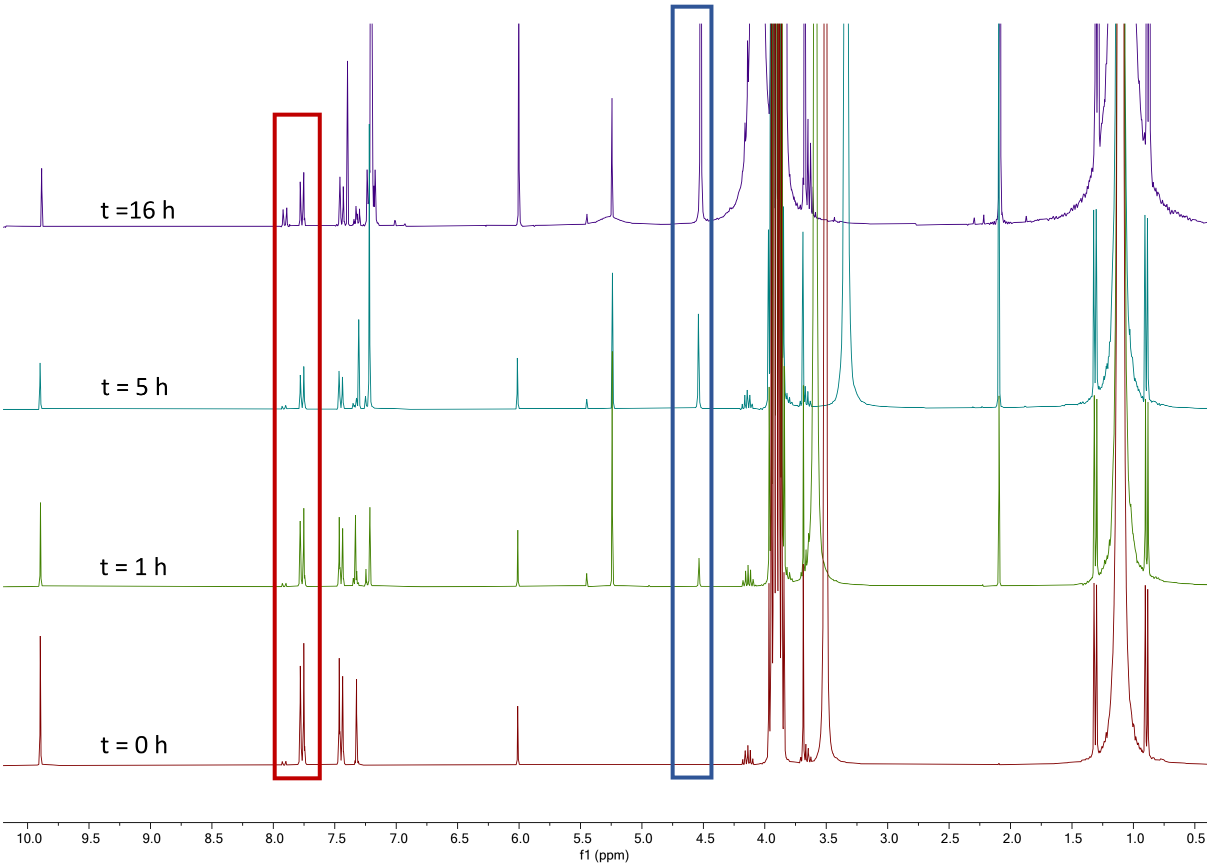

Figure S50: ^{1}H spectrum ($CDCl_3$, 298 K, 400 MHz) from the reduction of (**5a**) mediated by **Ru-2c** (1,3,5-trimethoxybenzene: 5 mg). Starting material consumption is highlighted red and formation of characteristic signal for product formation is highlighted in blue.

Reduction of 4-Acetylbenzaldehyde (6a) – Catalyzed by Ru-2a

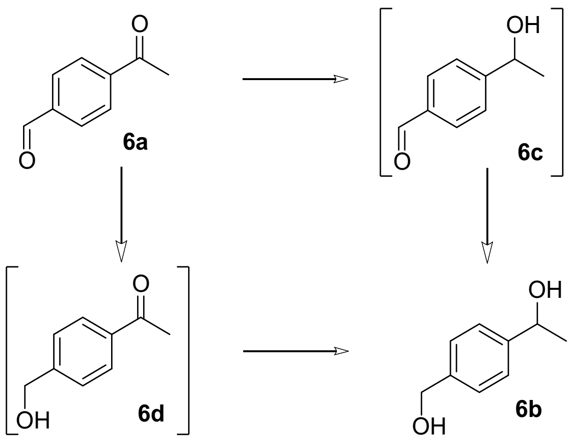

Time / h	6a / %	6c / %	6d / %	6b / %
1	29	6	16	49
2	6	3	7	84
3	2	1	3	94
5	<1	1	<5	>95

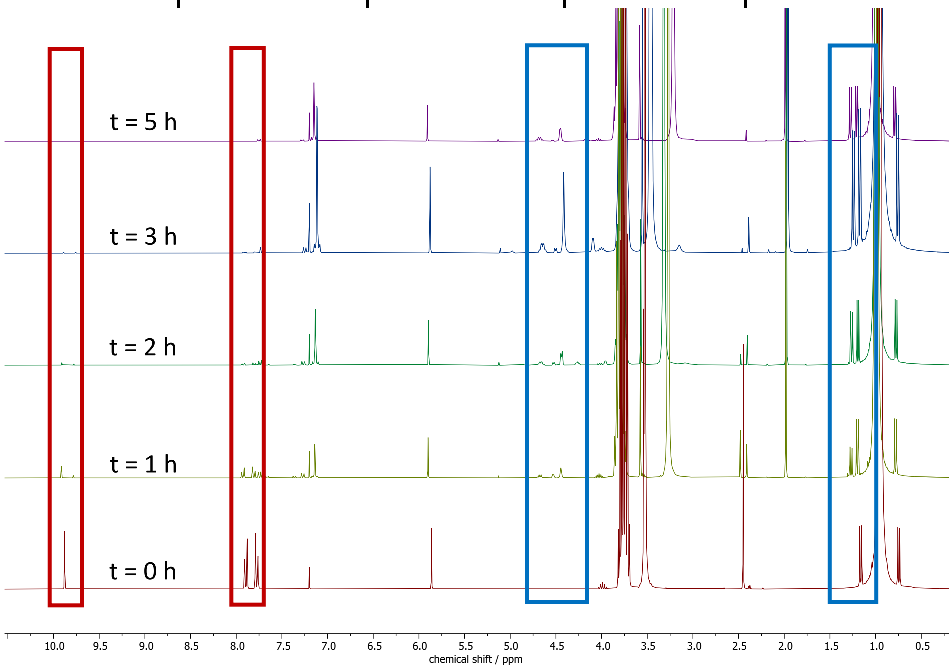

Figure S51: ^{1}H spectrum ($CDCl_3$, 298 K, 400 MHz) from the reduction of (**6a**) mediated by **Ru-2a** (1 mol%) (1,3,5-trimethoxybenzene: 14 mg). Starting material consumption is highlighted in red and formation of characteristic signal for product formation is highlighted in blue. **6d** was calculated based on **6a**, **6b** and **6c**.

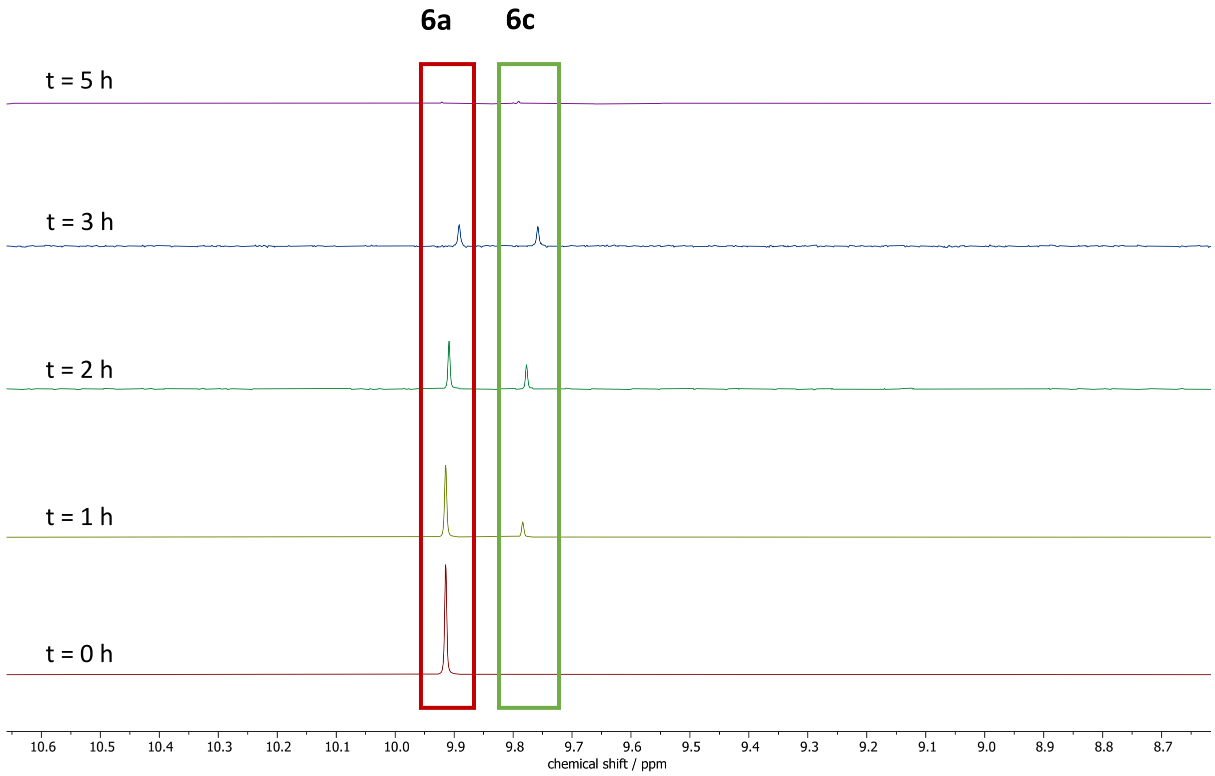

Figure S52: ^{1}H spectrum ($CDCl_3$, 298 K, 400 MHz) from the reduction of (**6a**) showing the aldehyde resonance from the starting material (**6a**) in red and the aldehyde signal for (**6c**) in green.

Reduction of Cyclohexanone (7a) – Catalyzed by Ru-2a

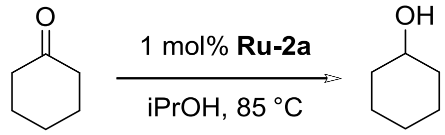

7a **7b**

Time / h	Yield / %
0.25	45
0.5	79
0.75	89
1	>95

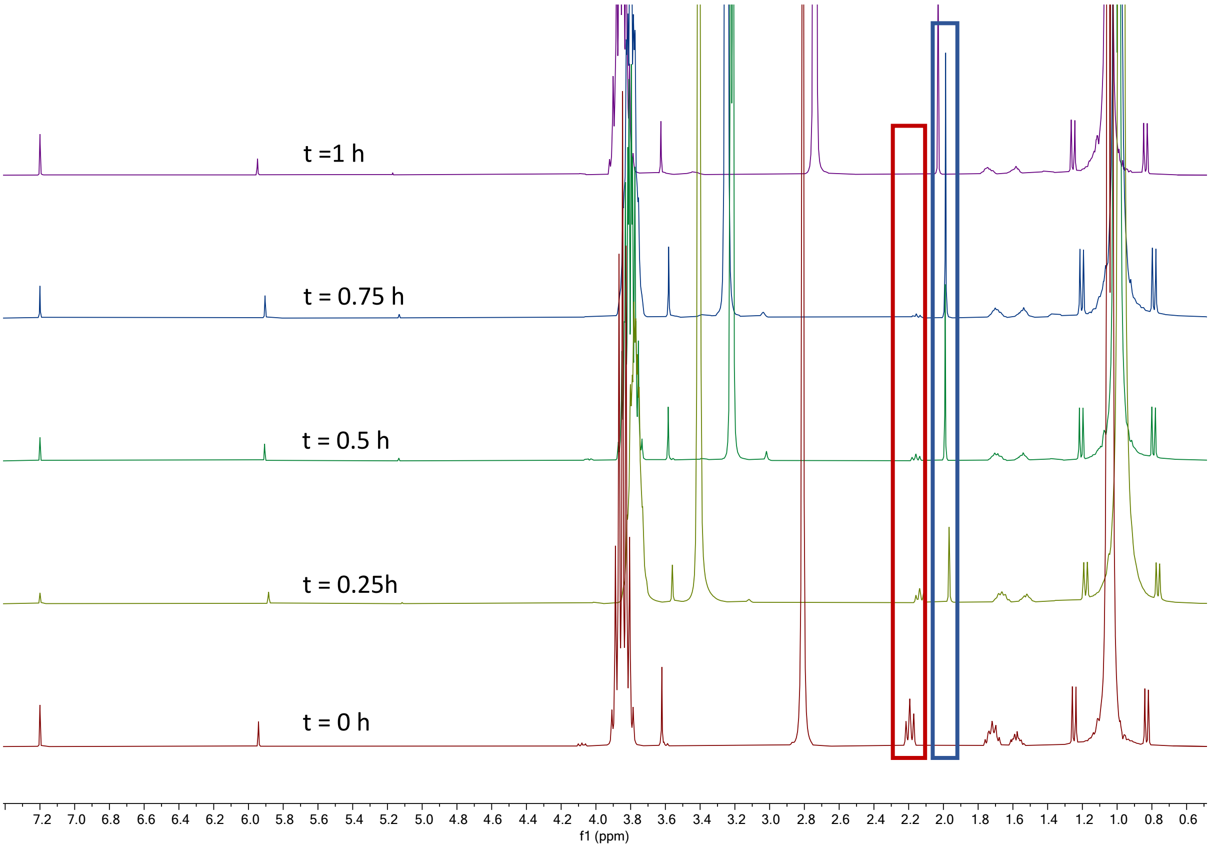

Figure S53: ^{1}H spectrum ($CDCl_3$, 298 K, 400 MHz) from the reduction of (**7a**) mediated by **Ru-2a** (1,3,5-trimethoxybenzene: 8 mg). Starting material consumption is highlighted in red and formation of characteristic signal for product formation is highlighted in blue.

Reduction of Cyclohexanone (7a) – Catalyzed by Ru-2b

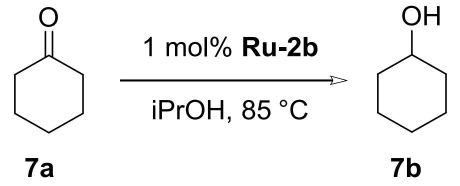

Time / h	Yield / %
0.25	70
0.5	83
0.75	93
1	>95

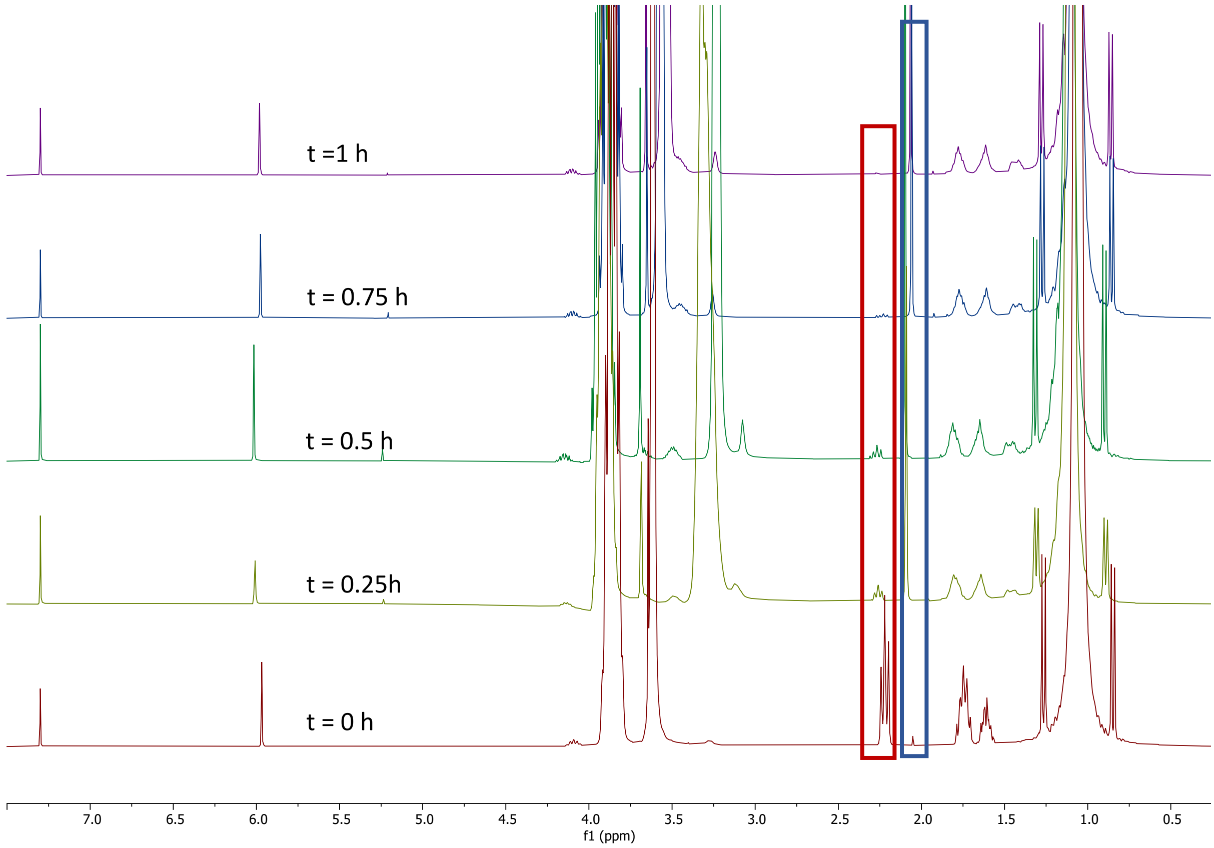

Figure S54: ^{1}H spectrum ($CDCl_3$, 298 K, 400 MHz) from the reduction of (**7a**) mediated by **Ru-2b** (1,3,5-trimethoxybenzene: 7 mg). Starting material consumption is highlighted red and formation of characteristic signal for product formation is highlighted in blue.

Reduction of Cyclohexanone (7a) – Catalyzed by Ru-2c

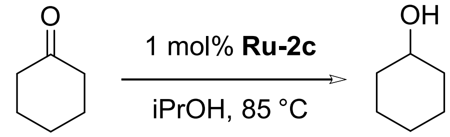

7a **7b**

Time / h	Yield / %
1	48
2	67
5	85
16	>95

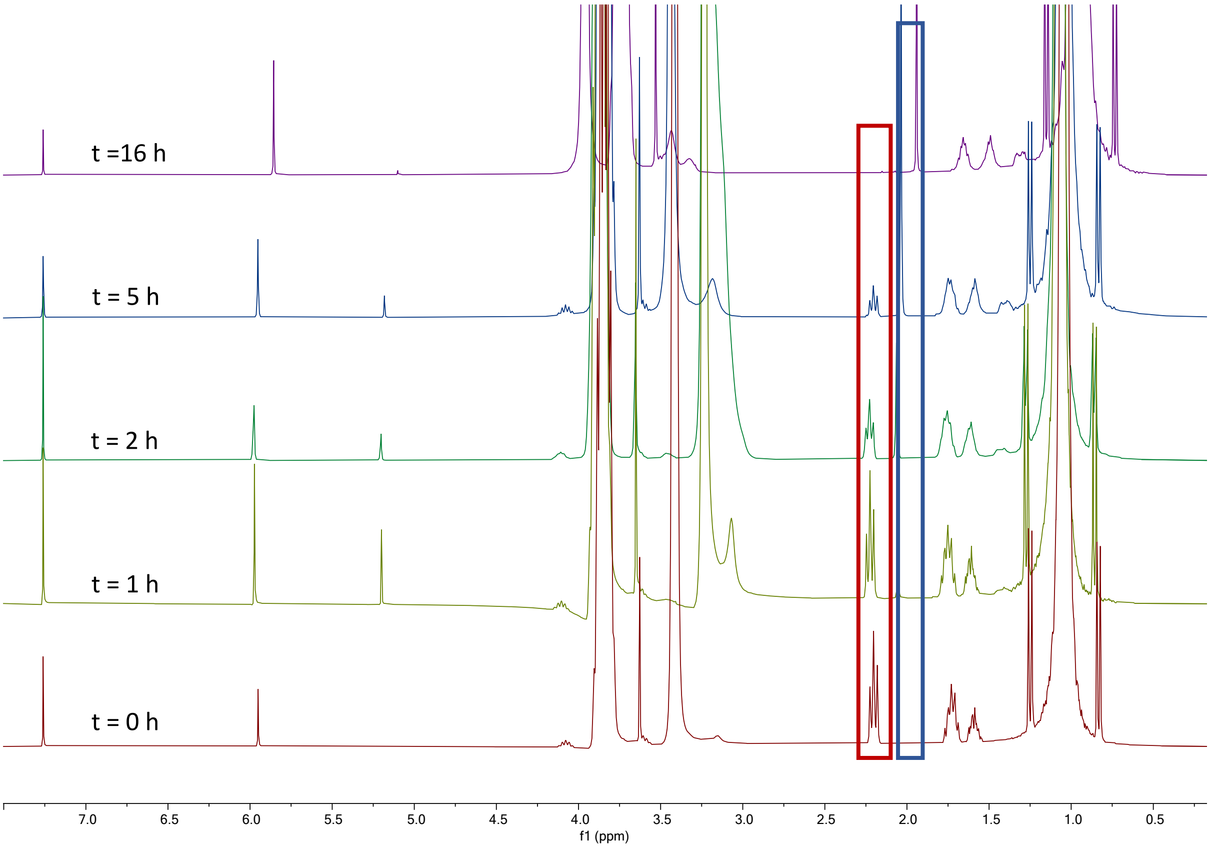

Figure S55: ^{1}H spectrum ($CDCl_3$, 298 K, 400 MHz) from the reduction of (**7a**) mediated by **Ru-2c** (1,3,5-trimethoxybenzene: 8 mg). Starting material consumption is highlighted in red and formation of characteristic signal for product formation is highlighted in blue.

Reduction of 4-Acetyldiphenyl sulfone (8a) – Catalyzed by Ru-2a

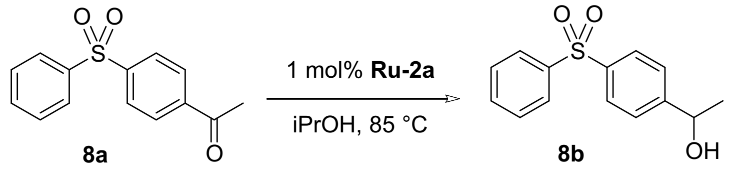

Time / h	Yield / %
1	87
2	94
3	>95

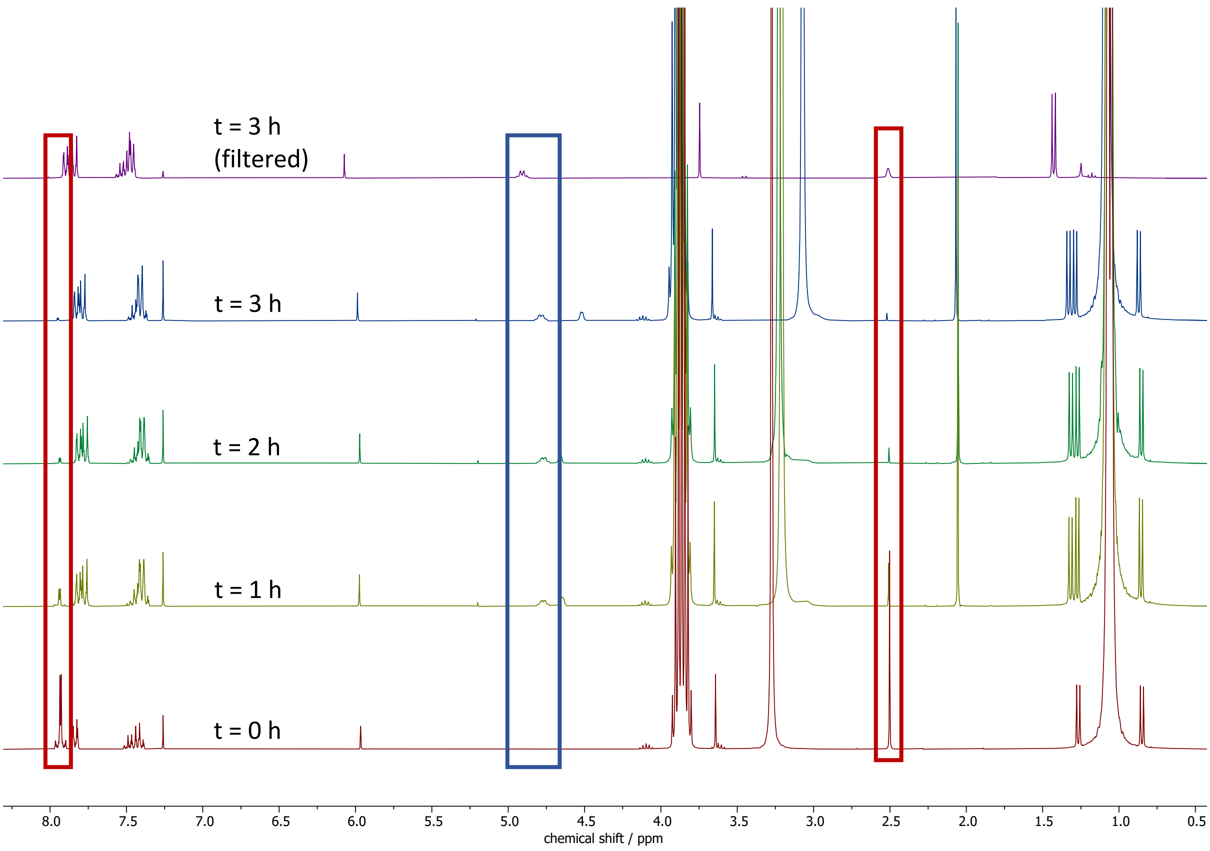

Figure S56: ^{1}H spectrum ($CDCl_3$, 298 K, 400 MHz) from the reduction of (**8a**) mediated by **Ru-2a** (1,3,5-trimethoxybenzene: 6 mg). Starting material consumption is highlighted in red and formation of characteristic signal for product formation is highlighted in blue.

Reduction of 4-Acetyldiphenyl sulfone (8a) – Catalyzed by Ru-2b

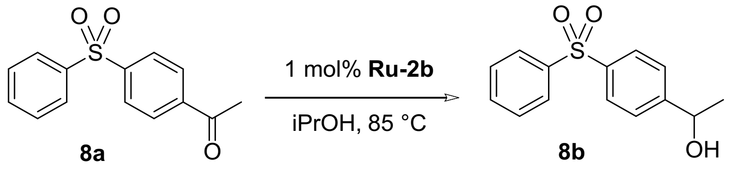

Time / h	Yield / %
1	92
2	>95
3	>95

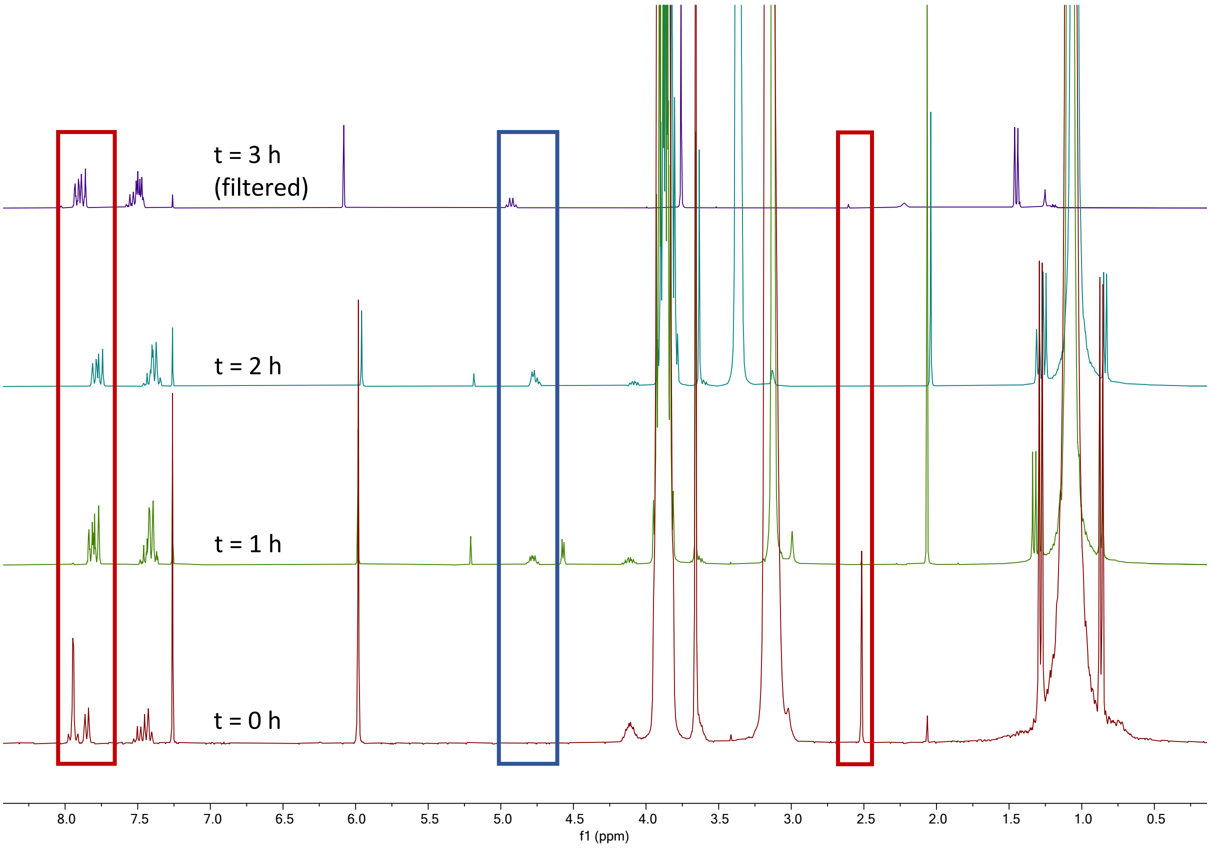

Figure S57: ^{1}H spectrum ($CDCl_3$, 298 K, 400 MHz) from the reduction of (**8a**) mediated by **Ru-2b** (1,3,5-trimethoxybenzene: 15 mg). Starting material consumption is highlighted in red and formation of characteristic signal for product formation is highlighted in blue.

HR-MS Calcd. for $C_{14}H_{15}O_3S$ $[M+H]^+$ 263.0734. Found: 263.0732.

Reduction of 4-Acetylbenzoic acid (9a) – Catalyzed by Ru-2a

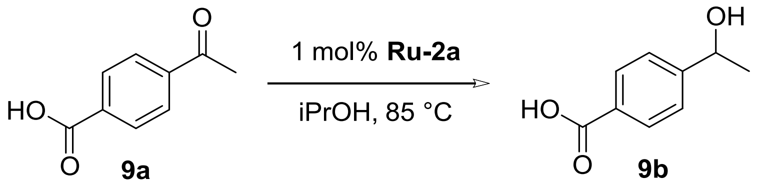

Time / h	Yield / %
1	3
24	29
48	41

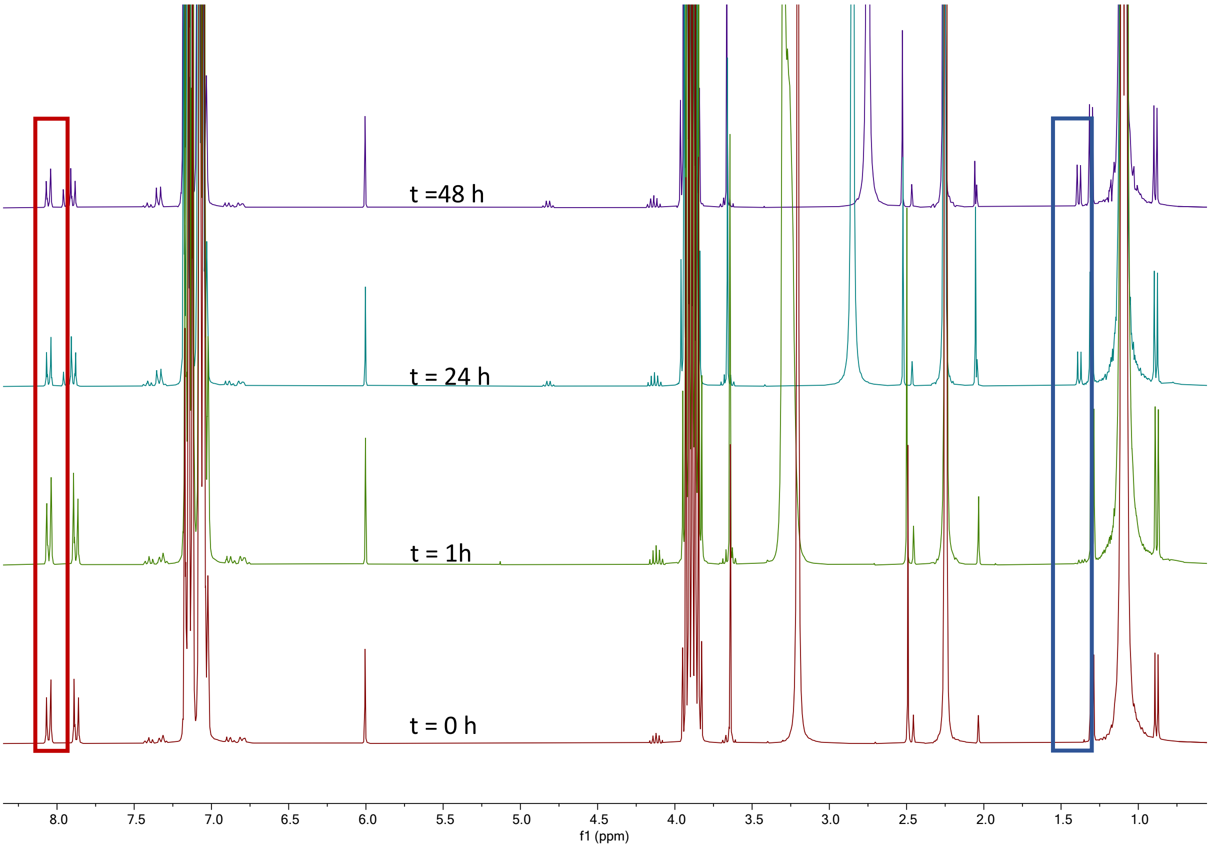

Figure S58: ^{1}H spectrum ($CDCl_3$, 298 K, 400 MHz) from the reduction of (**9a**) mediated by **Ru-2a** (1,3,5-trimethoxybenzene: 17 mg). Starting material consumption is highlighted in red and formation of characteristic signal for product formation is highlighted in blue.

Reduction of 4-Acetylbenzoic acid (9a) – Catalyzed by Ru-2b

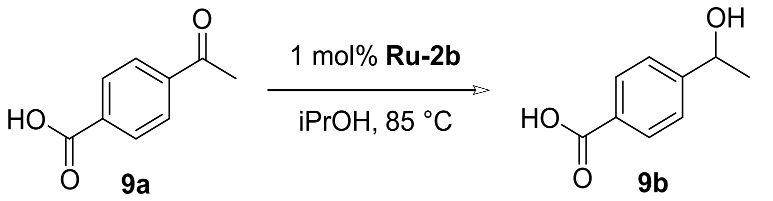

Time / h	Yield / %
1	6
24	42
48	56

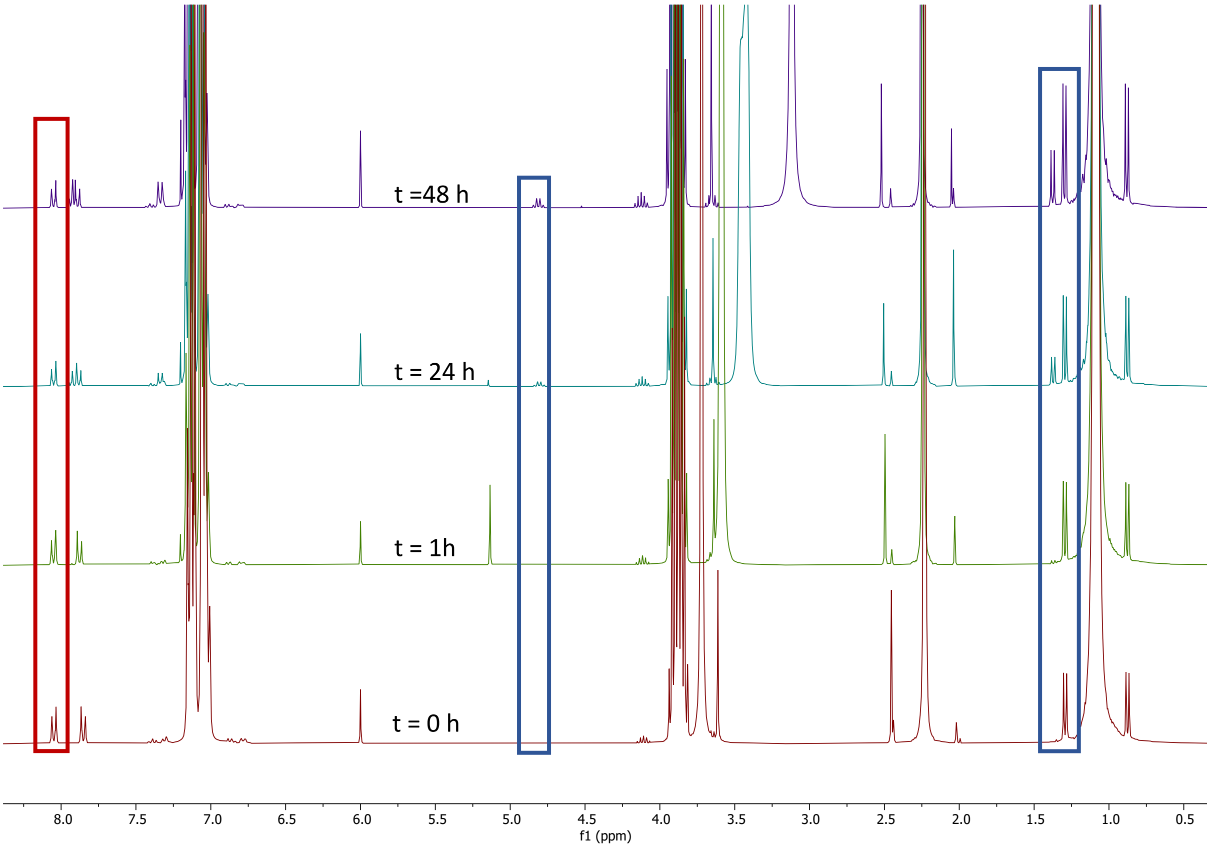

Figure S59: ^{1}H spectrum ($CDCl_3$, 298 K, 400 MHz) from the reduction of (**9a**) mediated by **Ru-2b** (1,3,5-trimethoxybenzene: 18 mg). For improved solubility of (**9a**), toluene (1 mL) was added to the reaction mixture. Starting material consumption is highlighted in red and formation of characteristic signal for product formation is highlighted in blue.

Reduction of 4-Acetylbenzoic acid (9a) – Catalyzed by Ru-2c

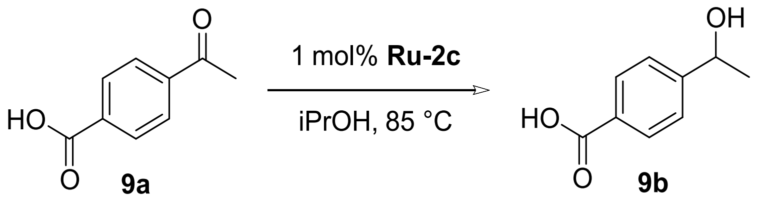

Time / h	Yield / %
1	0
24	10
48	11

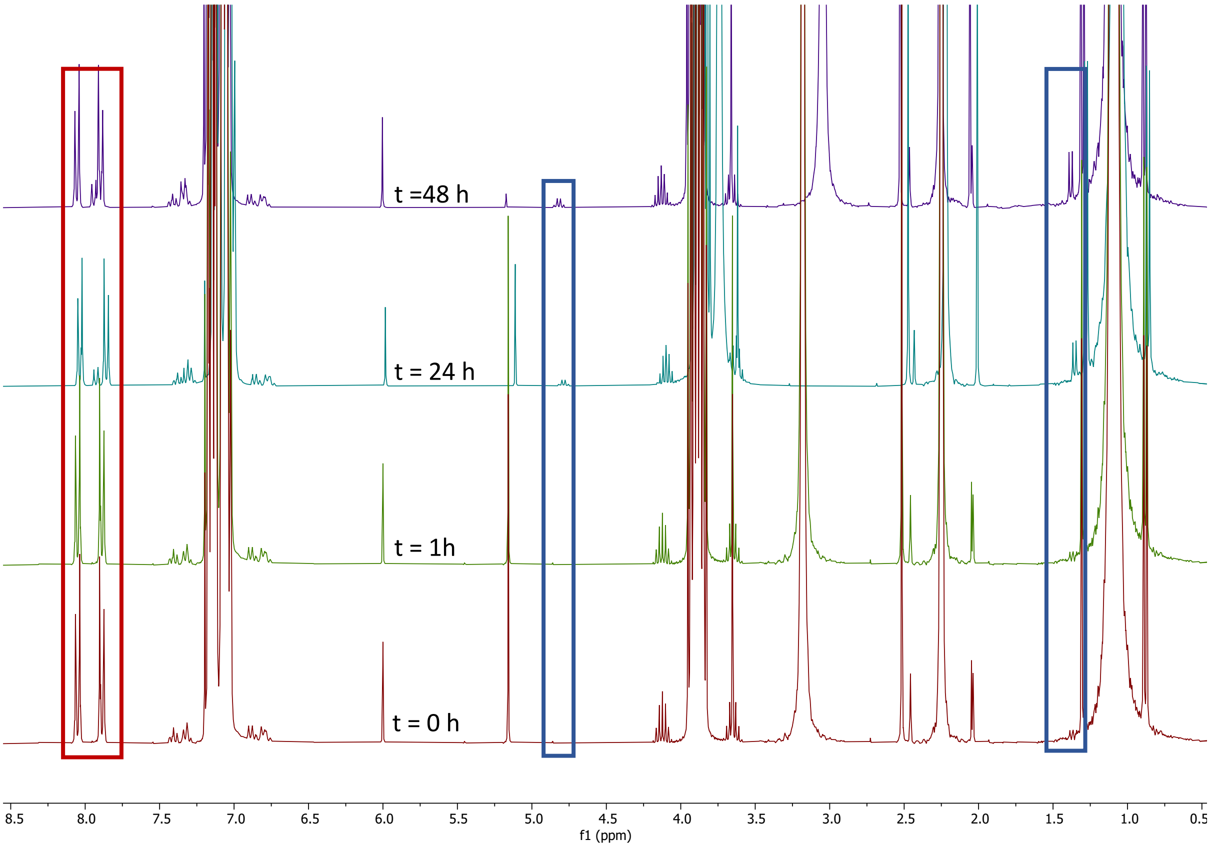

Figure S60: ^{1}H spectrum ($CDCl_3$, 298 K, 400 MHz) from the reduction of (**9a**) mediated by **Ru-2c** (1,3,5-trimethoxybenzene: 6 mg). For improved solubility of (**9a**), toluene (1 mL) was added to the reaction mixture. Starting material consumption is highlighted red and formation of characteristic signal for product formation is highlighted in blue.

Reduction of 4-Benzoylbenzoic acid (10a) – Catalyzed by Ru-2a

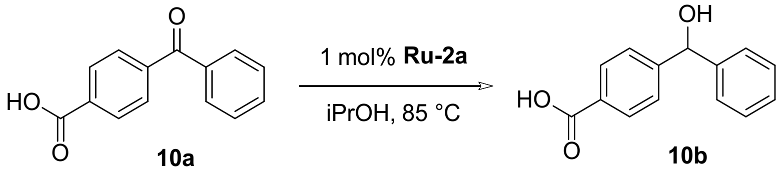

Time / h	Yield / %
4	19
16	38
48	48

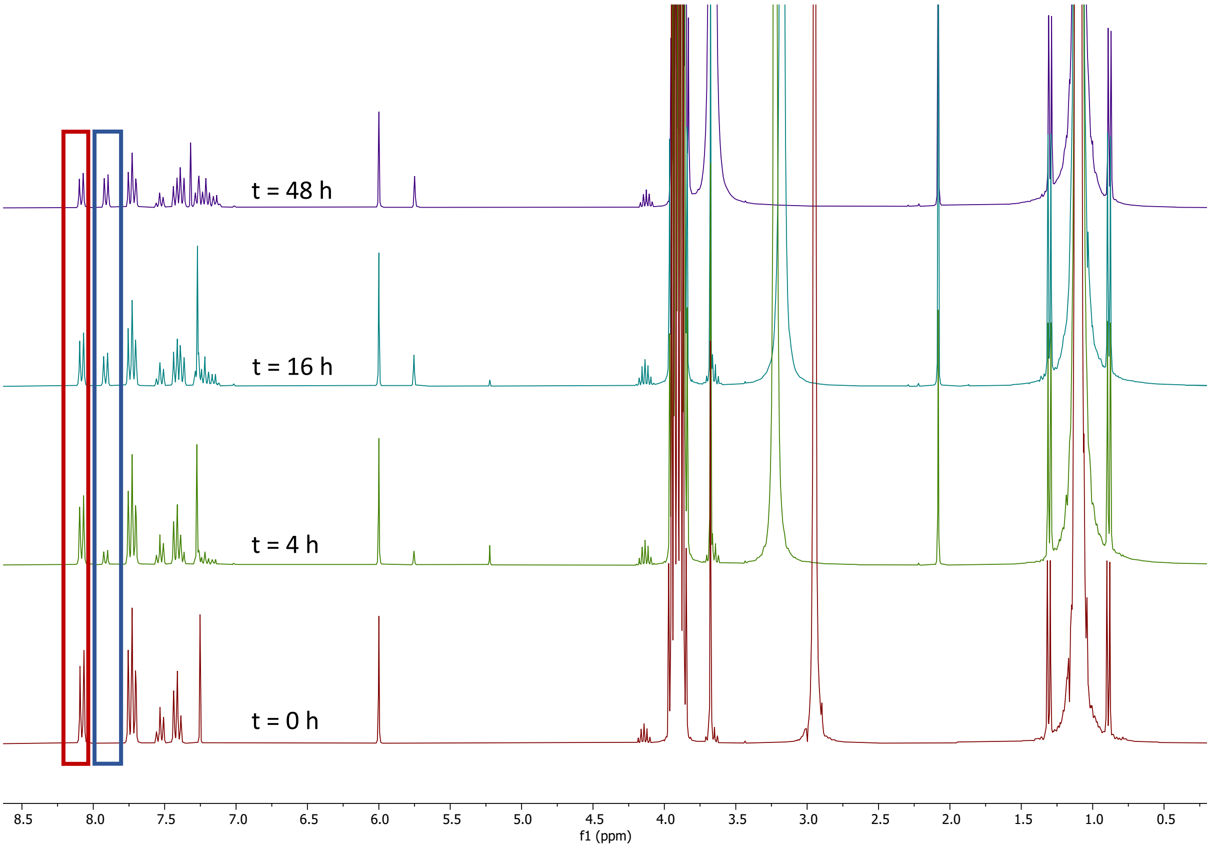

Figure S61: ^{1}H spectrum ($CDCl_3$, 298 K, 400 MHz) from the reduction of (**10a**) mediated by **Ru-2a** (1,3,5-trimethoxybenzene: 11 mg). Starting material consumption is highlighted red and formation of characteristic signal for product formation is highlighted in blue.

Reduction of 4-Benzoylbenzoic acid (10a) – Catalyzed by Ru-2b

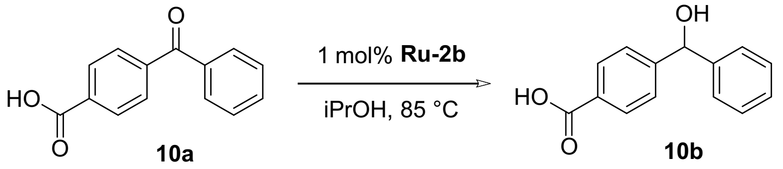

Time / h	Yield / %
1	4
24	35
48	37

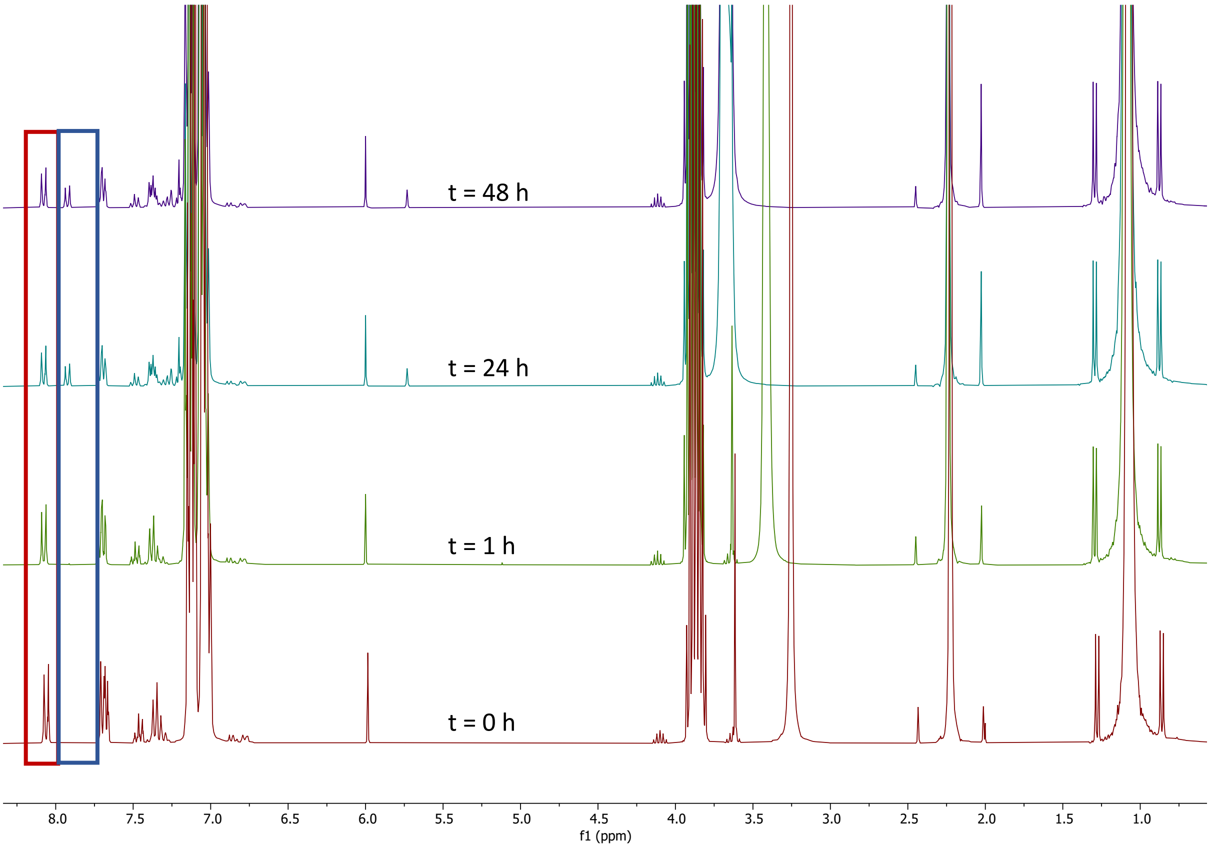

Figure S62: ^{1}H spectrum ($CDCl_3$, 298 K, 400 MHz) from the reduction of (**10a**) mediated by **Ru-2b** (1,3,5-trimethoxybenzene: 12 mg). For improved solubility of (**10a**), toluene (1 mL) was added to the reaction mixture. Starting material consumption is highlighted red and formation of characteristic signal for product formation is highlighted in blue.

Reduction of 4-Aminoacetophenone (11a) – Catalyzed by Ru-2a

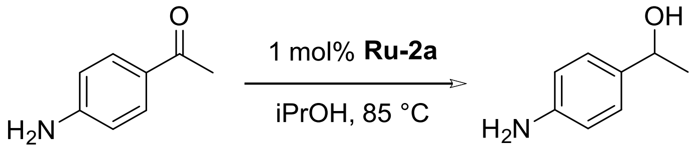

11a **11b**

Time / h	Yield / %
0.5	22
1	40
2	48

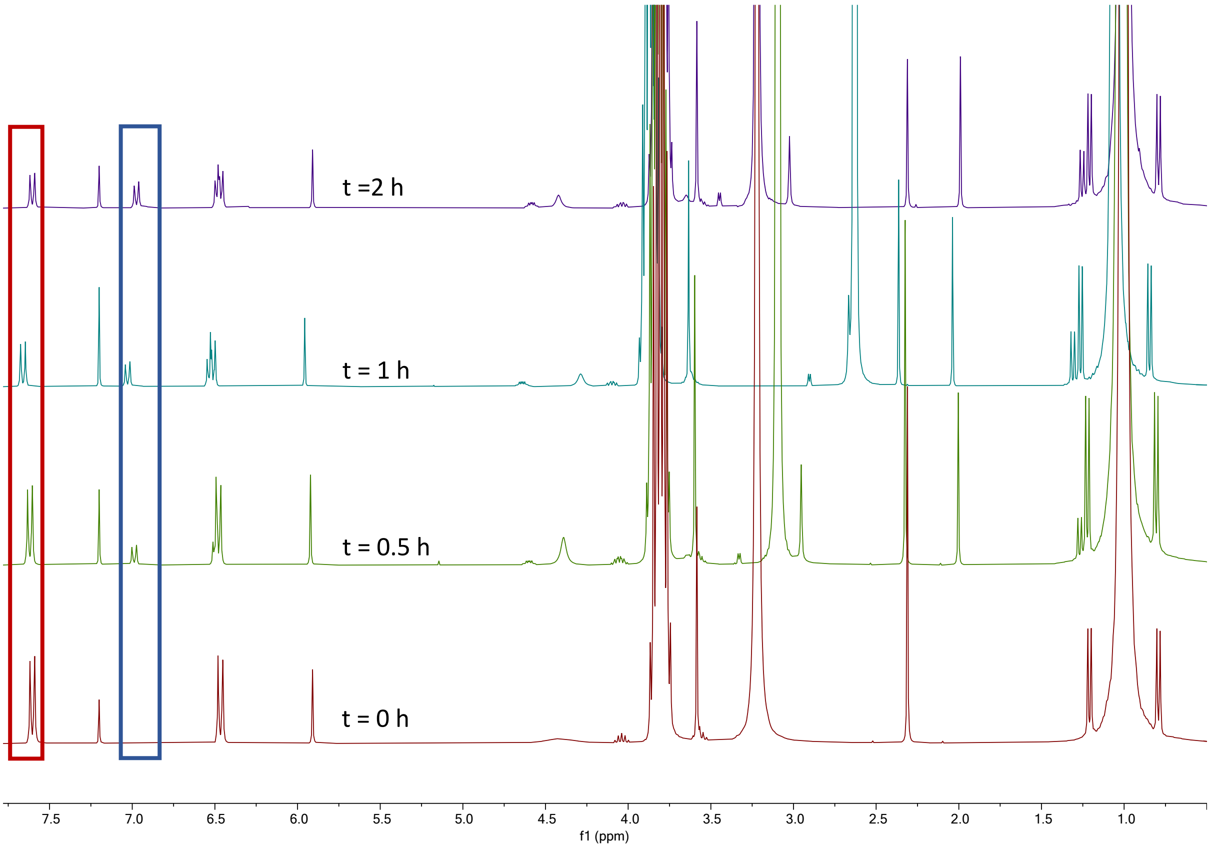

Figure S63: ^{1}H spectrum ($CDCl_3$, 298 K, 400 MHz) from the reduction of (**11a**) mediated by **Ru-2a** (1,3,5-trimethoxybenzene: 10 mg). Starting material consumption is highlighted in red and formation of characteristic signal for product formation is highlighted in blue.

Reduction of 4-Aminoacetophenone (11a) – Catalyzed by Ru-2b

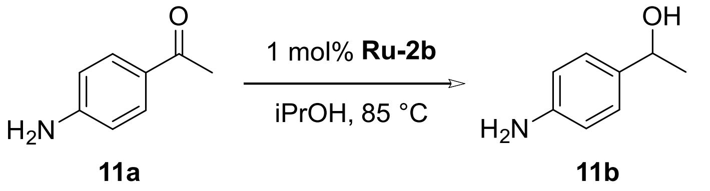

Time / h	Yield / %
0.5	16
1	24
2	41

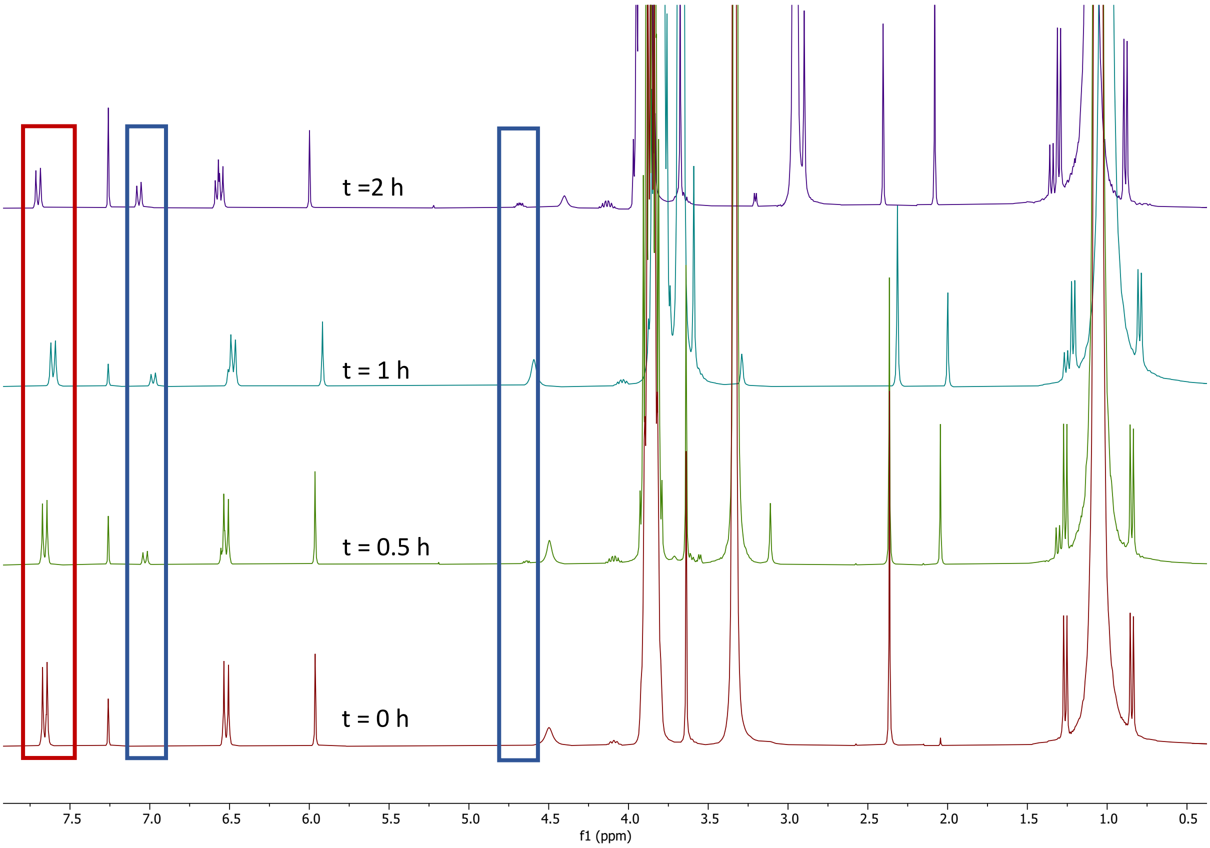

Figure S64: ^{1}H spectrum ($CDCl_3$, 298 K, 400 MHz) from the reduction of (**11a**) mediated by **Ru-2b** (1,3,5-trimethoxybenzene: 12 mg). Starting material consumption is highlighted in red and formation of characteristic signal for product formation is highlighted in blue.

Reduction of 4-Acetamidoacetophenone (12a) – Catalyzed by Ru-2a

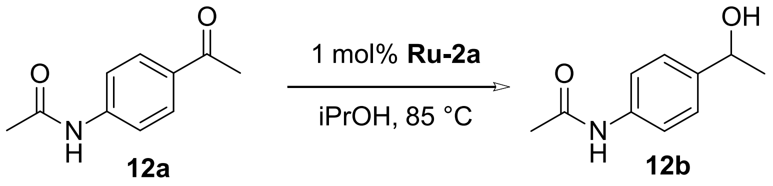

Time / h	Conv. / %
1	44
2	74
3	83
5	91
16	93

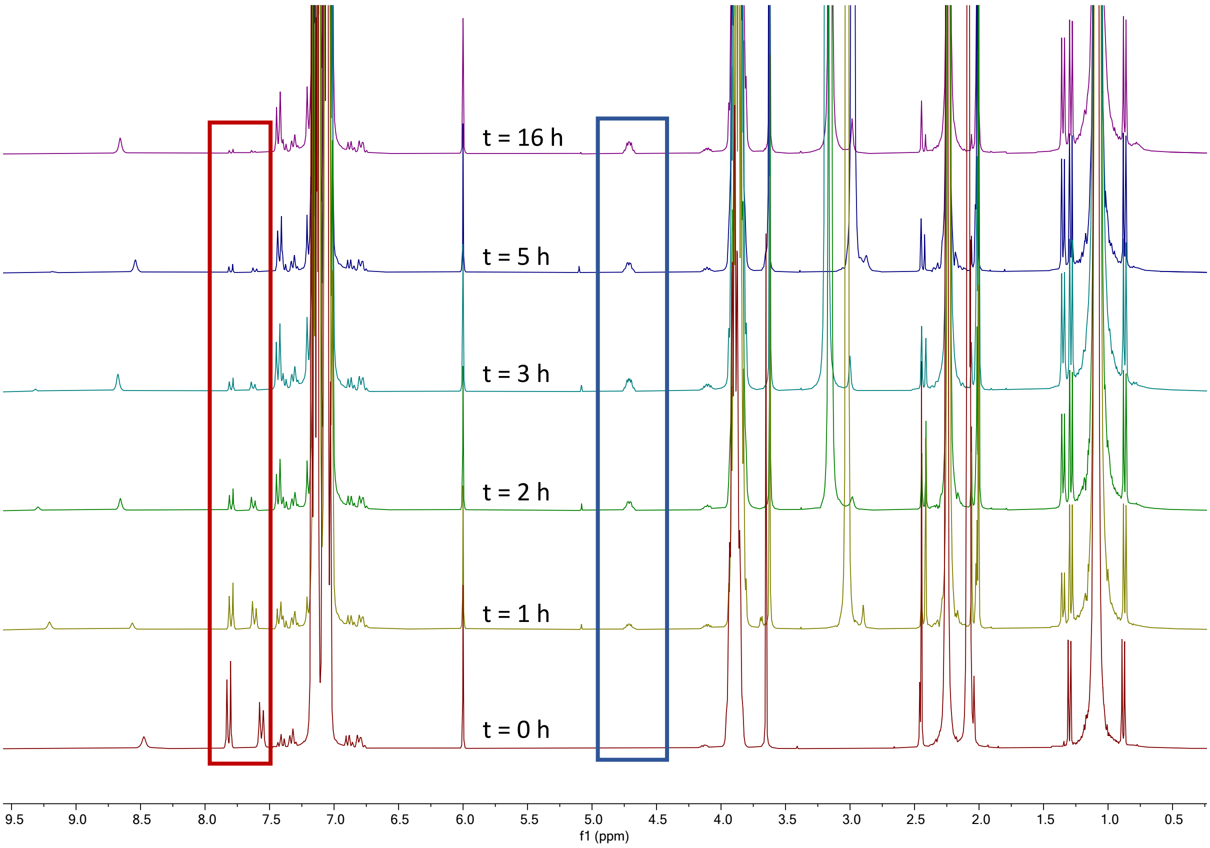

Figure S65: ^{1}H spectrum ($CDCl_3$, 298 K, 400 MHz) from the reduction of (**12a**) mediated by **Ru-2a** (1,3,5-trimethoxybenzene: 20 mg). For improved solubility of (**12a**), toluene (1.5 mL) was added to the reaction mixture. Starting material consumption is highlighted in red and formation of characteristic signal for product formation is highlighted in blue.

Reduction of 4-Acetamidoacetophenone (12a) – Catalyzed by Ru-2b

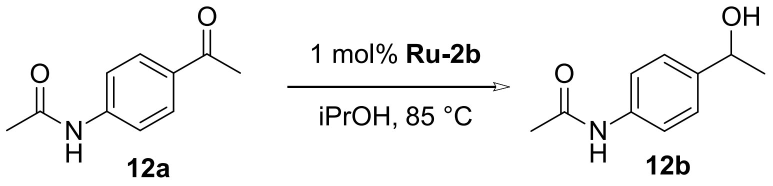

Time / h	Conv. / %
1	82
2	84

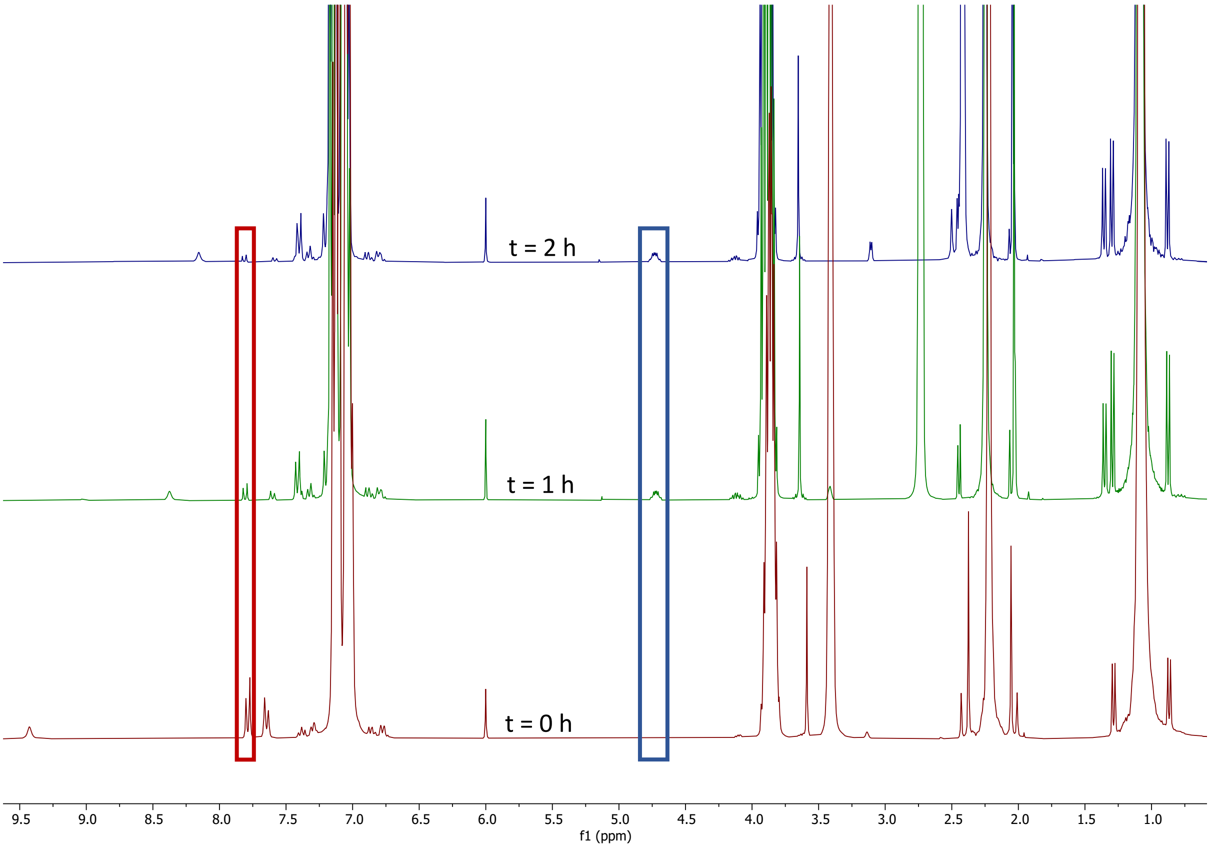

Figure S66: ^{1}H spectrum ($CDCl_3$, 298 K, 400 MHz) from the reduction of (**12a**) mediated by **Ru-2b** (1,3,5-trimethoxybenzene: 13 mg). For improved solubility of (**12a**), toluene (1.5 mL) was added to the reaction mixture. Starting material consumption is highlighted in red and formation of characteristic signal for product formation is highlighted in blue.

HR-MS Calcd. for $C_{10}H_{14}O_2N$ $[M+H]^+$ 180.1019. d: 180.1018.

Reduction of 4-Acetamidoacetophenone (12a) – Catalyzed by Ru-2c

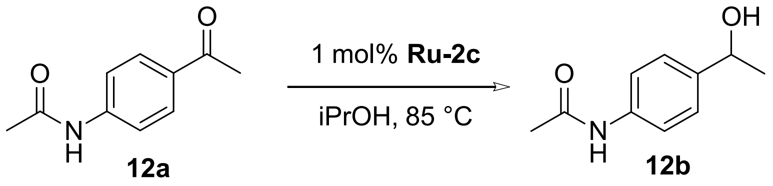

Time / h	Conv. / %
1	16
2	35
3	50
5	63
16	92

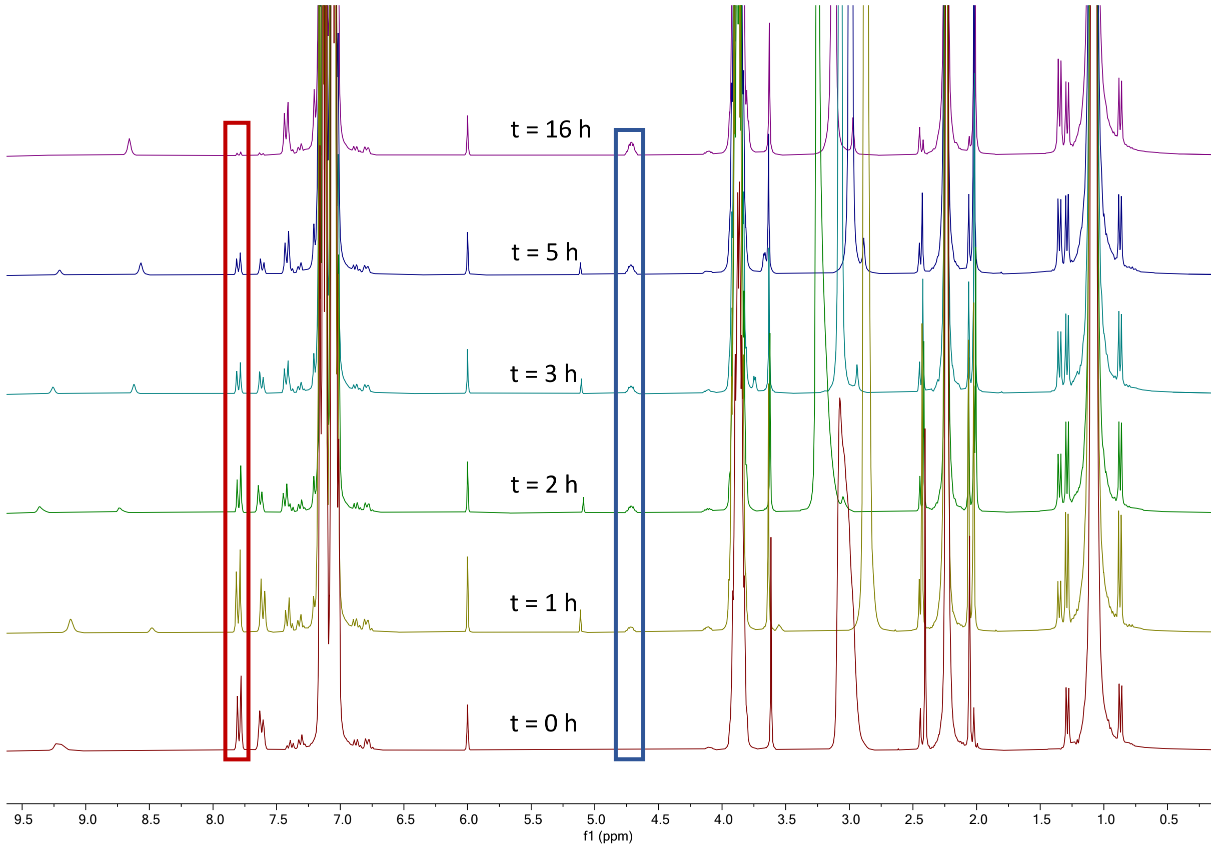

Figure S67: ^{1}H spectrum ($CDCl_3$, 298 K, 400 MHz) from the reduction of (**12a**) mediated by **Ru-2c** (1,3,5-trimethoxybenzene: 15 mg). For improved solubility of (**12a**), toluene (1.5 mL) was added to the reaction mixture. Starting material consumption is highlighted red and formation of characteristic signal for product formation is highlighted in blue.

Reduction of 4-Acetylphenyl acetate (13a) – Catalyzed by Ru-2a

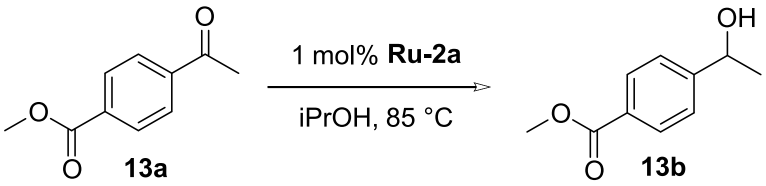

Time / h	Yield / %
1	88
2	>95%

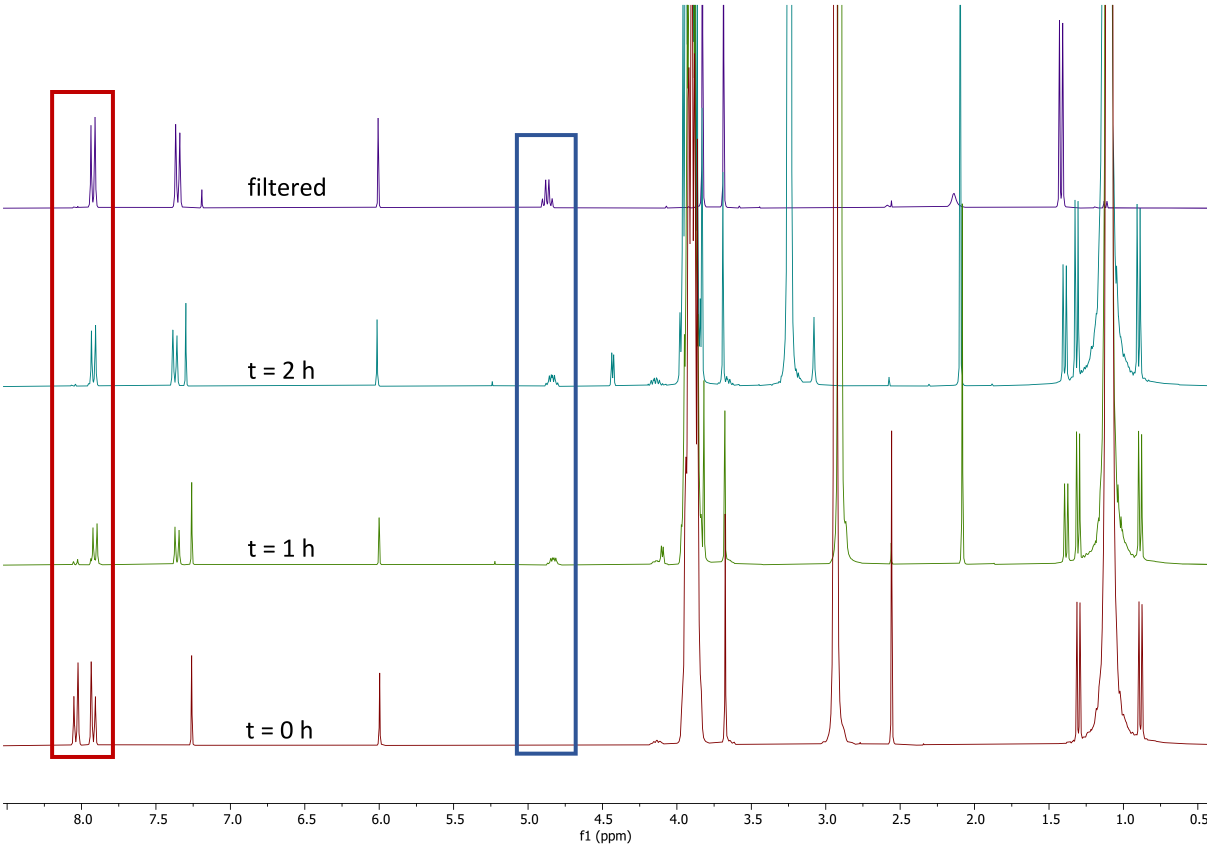

Figure S68: ^{1}H spectrum ($CDCl_3$, 298 K, 400 MHz) from the reduction of (**13a**) mediated by **Ru-2a** (1,3,5-trimethoxybenzene: 13 mg). Starting material consumption is highlighted in red and formation of characteristic signal for product formation is highlighted in blue.

HR-MS Calcd. for $C_{10}H_{12}O_3Na$ $[M+Na]^+$ 203.0679. Found: 203.0676.

Reduction of 1-(4-(oxiran-2-ylmethoxy)phenyl)ethan-1-one (14a) – Catalyzed by Ru-2a

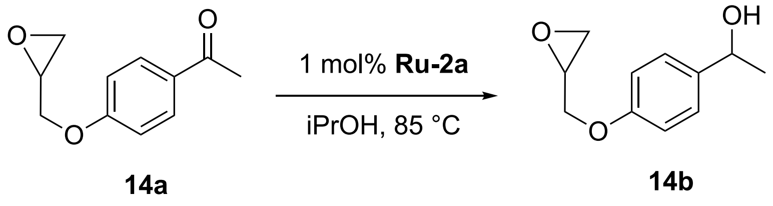

Time / h	Yield / %
2.5	95

Spectroscopic yield from **14a** (55.15 mg, 0.287 mmol), **Ru-2a** (1.4 mg, 2.87 µmol), hexamethylbenzene (8 mg). Isolated by gradient column chromatography (Al_2O_3, cyclohexane:EtOAc 4:1 to 100% EtOAc).

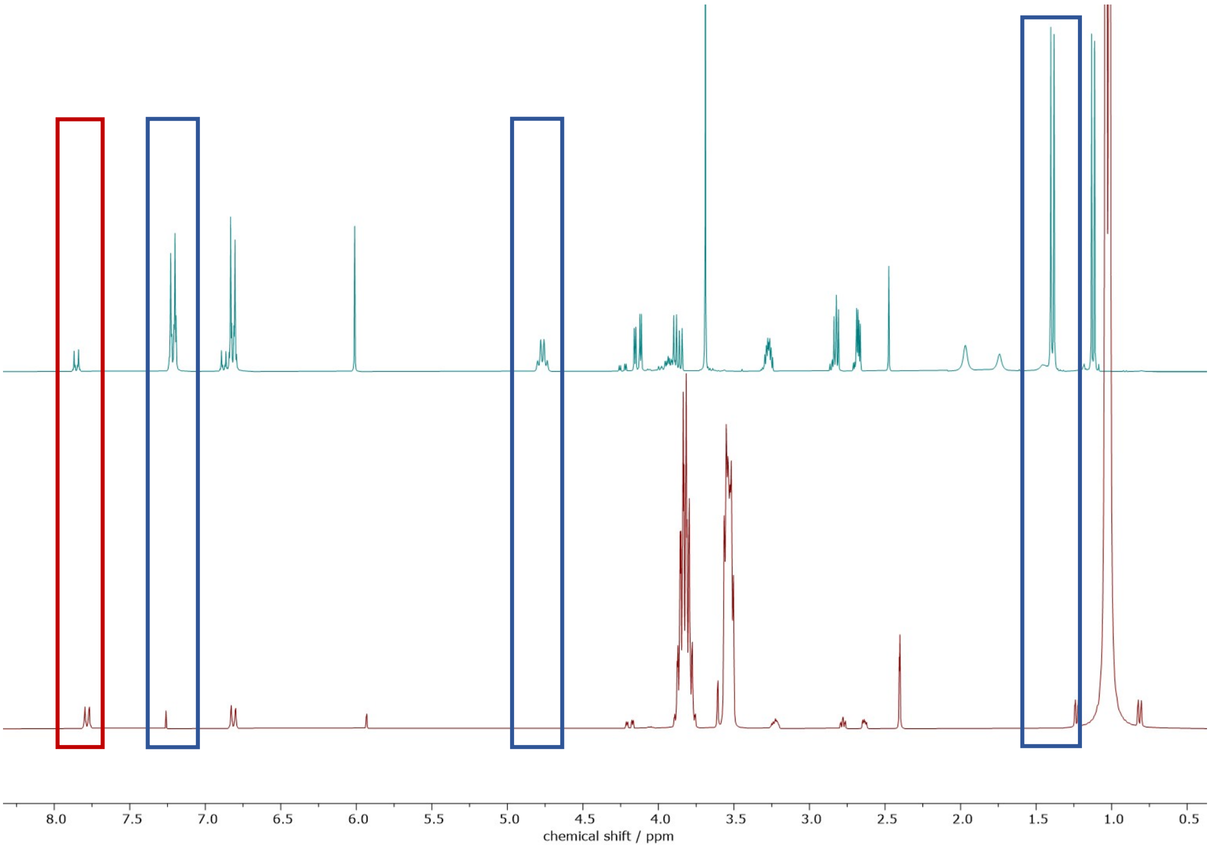

Figure S69: ^{1}H spectrum ($CDCl_3$, 298 K, 400 MHz) from the reduction of (**14a**) mediated by **Ru-2a** (1,3,5-trimethoxybenzene: 8 mg). Starting material consumption is highlighted in red and formation of characteristic signal for product formation is highlighted in blue.

GC-MS Calcd. for $C_{11}H_{14}O_3$ $[M]^+$ 194.0937. Found 194.0941.

Reduction of Acetomesitylene (15a) – Catalyzed by Ru-2a

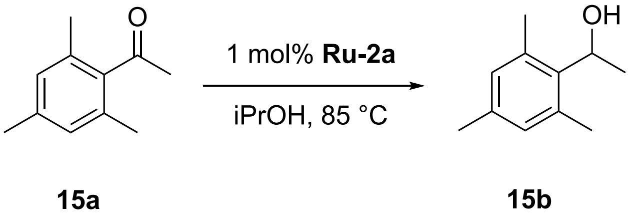

Time / h	Yield / %
1	58
2	80
3	91
4	93
16	>95

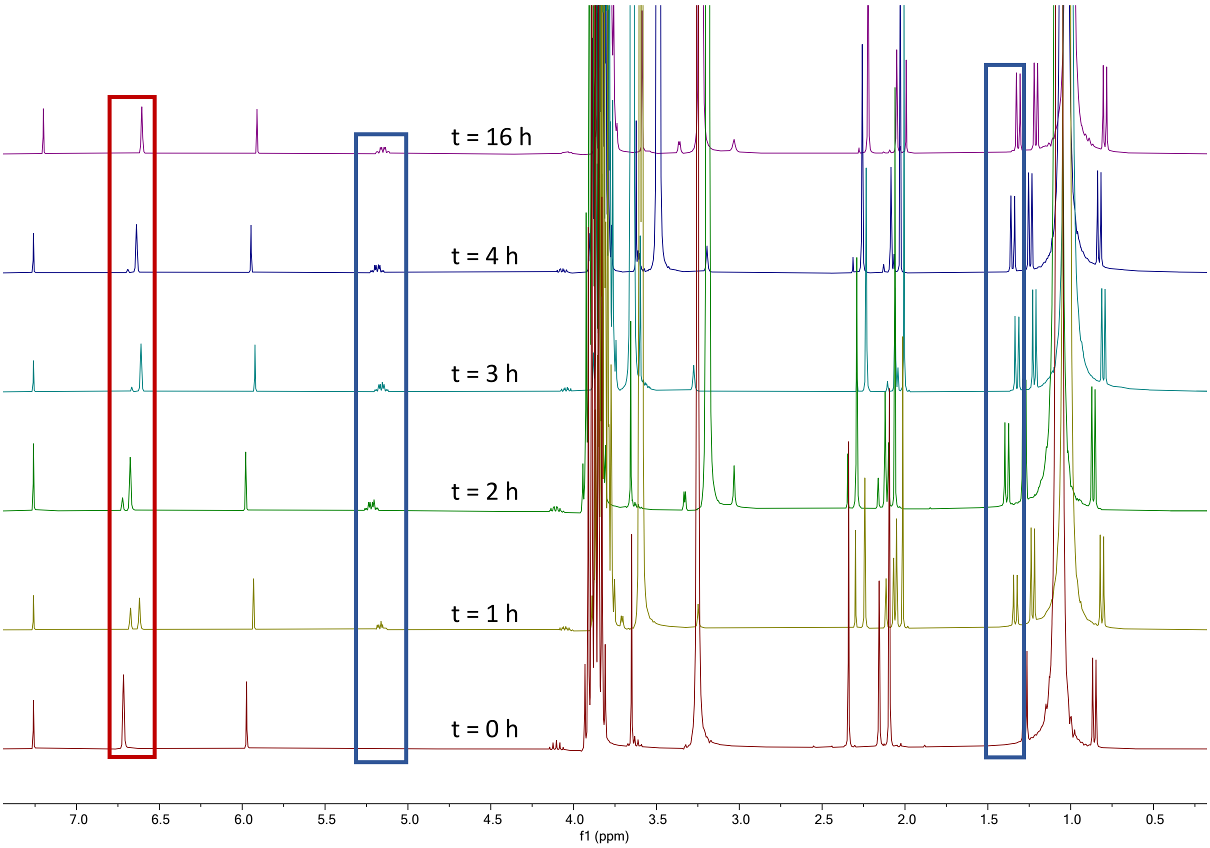

Figure S70: ^{1}H spectrum ($CDCl_3$, 298 K, 400 MHz) from the reduction of (**15a**) mediated by **Ru-2a** (1,3,5-trimethoxybenzene: 8 mg). Starting material consumption is highlighted in red and formation of characteristic signal for product formation is highlighted in blue.

Reduction of Acetomesitylene (15a) – Catalyzed by Ru-2b

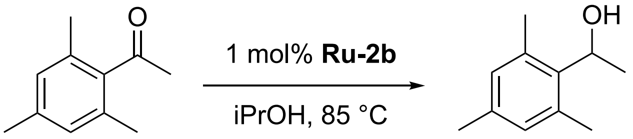

15a **15b**

Time / h	Yield / %
1	48
2	74
3	84
4	90
16	>95

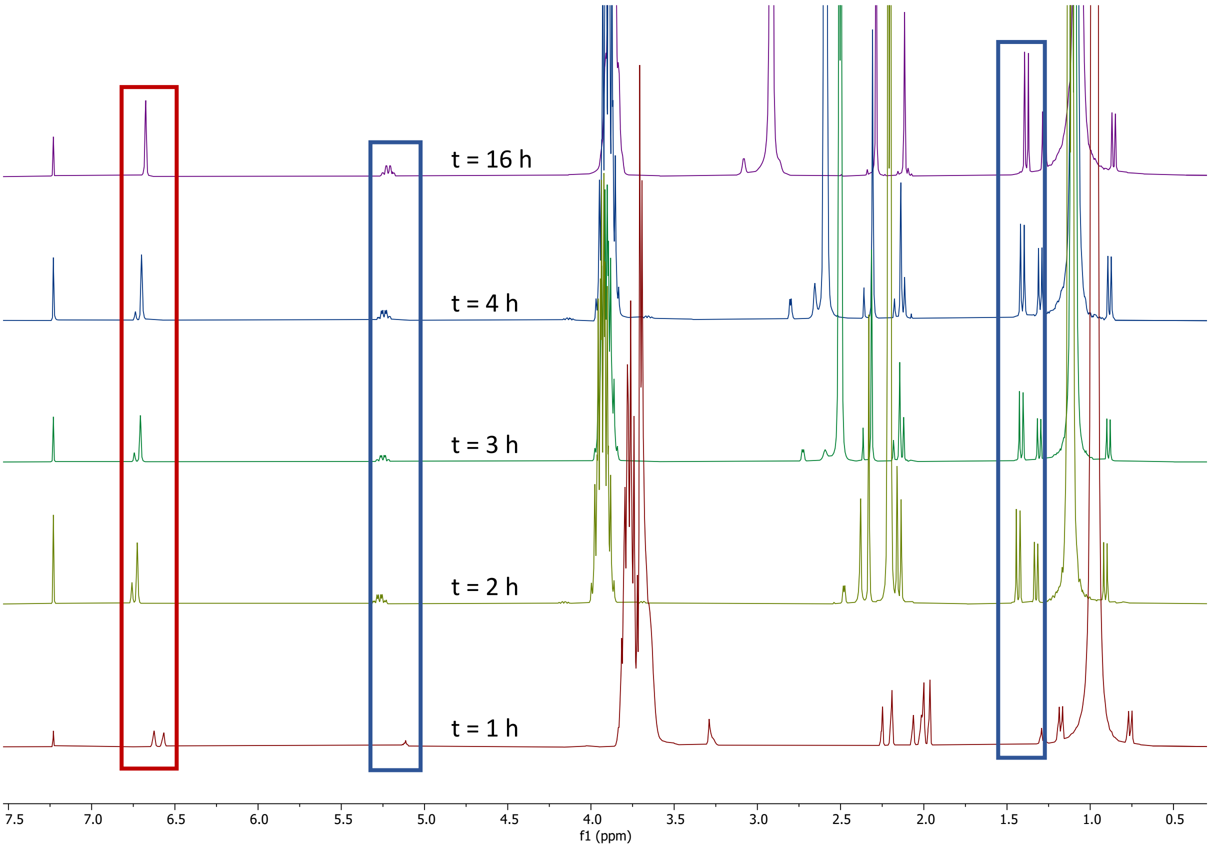

Figure S71: ^{1}H spectrum ($CDCl_3$, 298 K, 400 MHz) from the reduction of (**15a**) mediated by **Ru-2b**. Starting material consumption is highlighted in red and formation of characteristic signal for product formation is highlighted in blue.

Reduction of Acetomesitylene (15a) – Catalyzed by Ru-2c

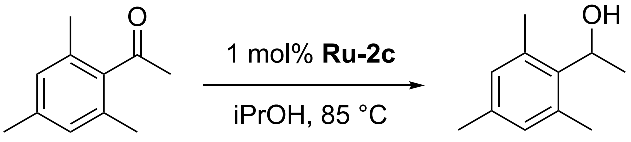

15a **15b**

Time / h	Yield / %
1	14
2	19
3	20
16	23

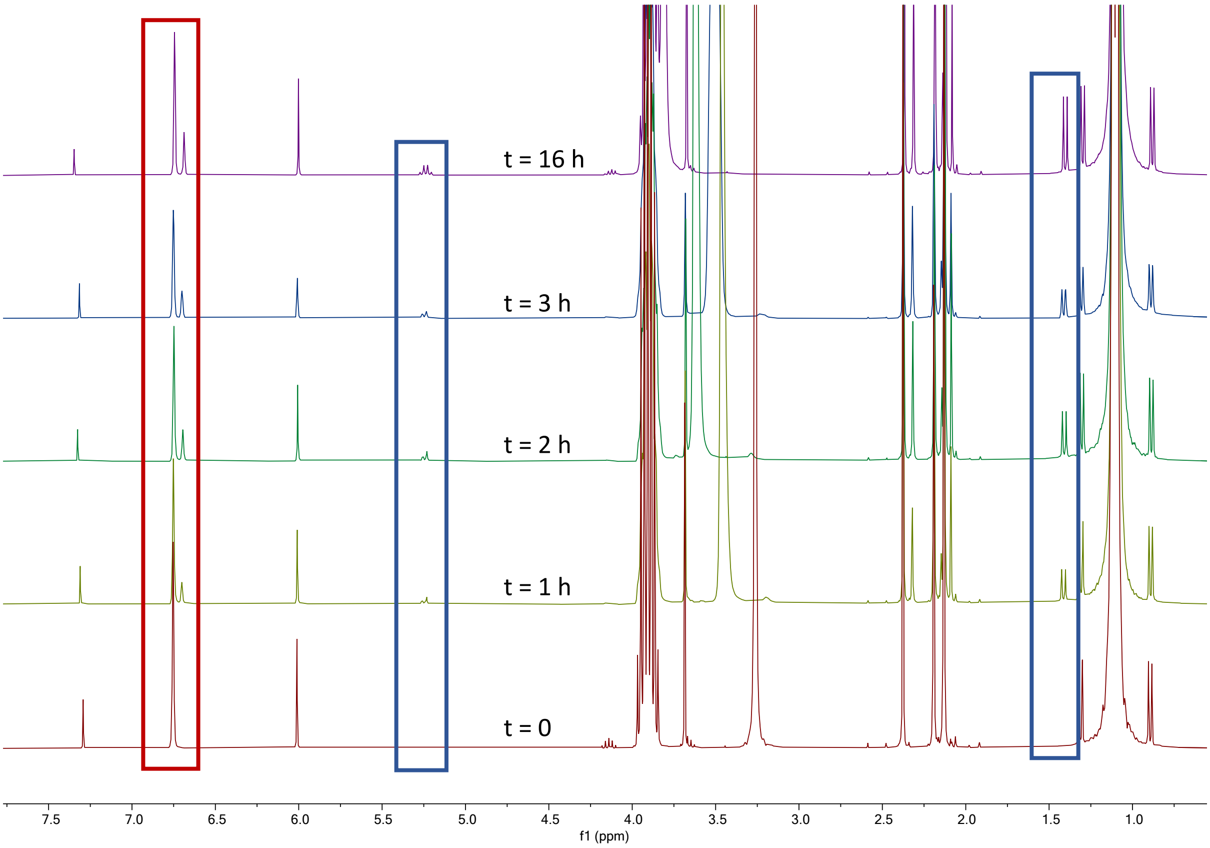

Figure S72: ^{1}H spectrum ($CDCl_3$, 298 K, 400 MHz) from the reduction of (**15a**) mediated by **Ru-2c** (1,3,5-trimethoxybenzene: 17 mg). Starting material consumption is highlighted in red and formation of characteristic signal for product formation is highlighted in blue.

Reduction of Acetomesitylene (15a) – Catalyzed by Ru-4

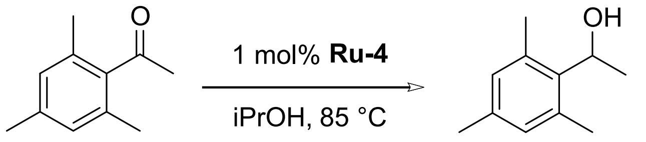

15a **15b**

Time / h	Yield / %
4	21

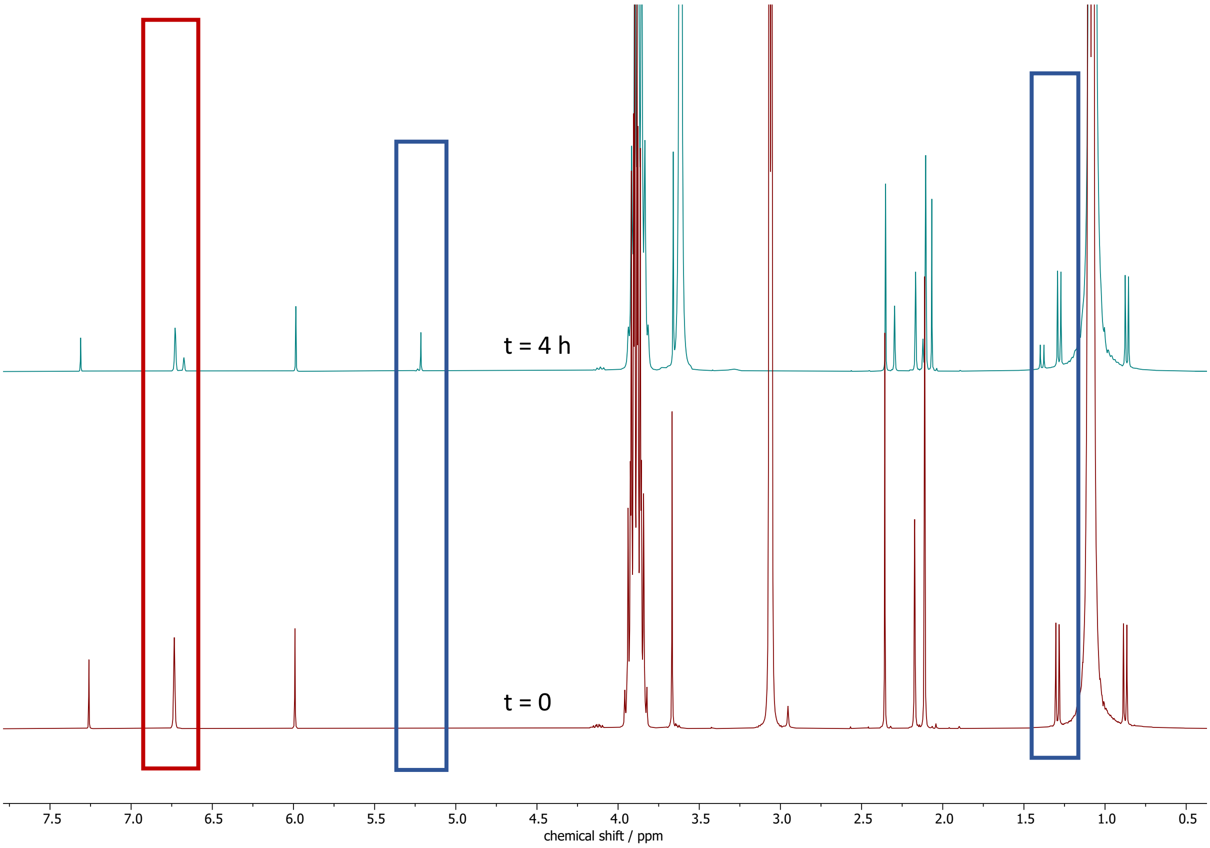

Figure S73: ^{1}H spectrum ($CDCl_3$, 298 K, 400 MHz) from the reduction of (**15a**) mediated by **Ru-4** (1,3,5-trimethoxybenzene: 11 mg). Starting material consumption is highlighted in red and formation of characteristic signal for product formation is highlighted in blue.

Reduction of 2,4,6-tri-isopropylacetophenone (16a) – Catalyzed by Ru-2a

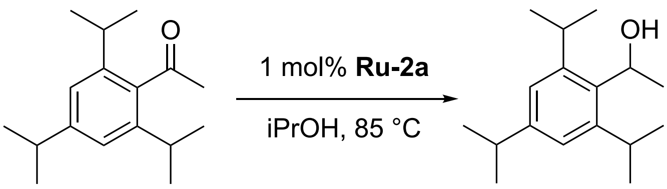

16a **16b**

Time / h	Yield / %
1	14
3	21
5	22
16	42

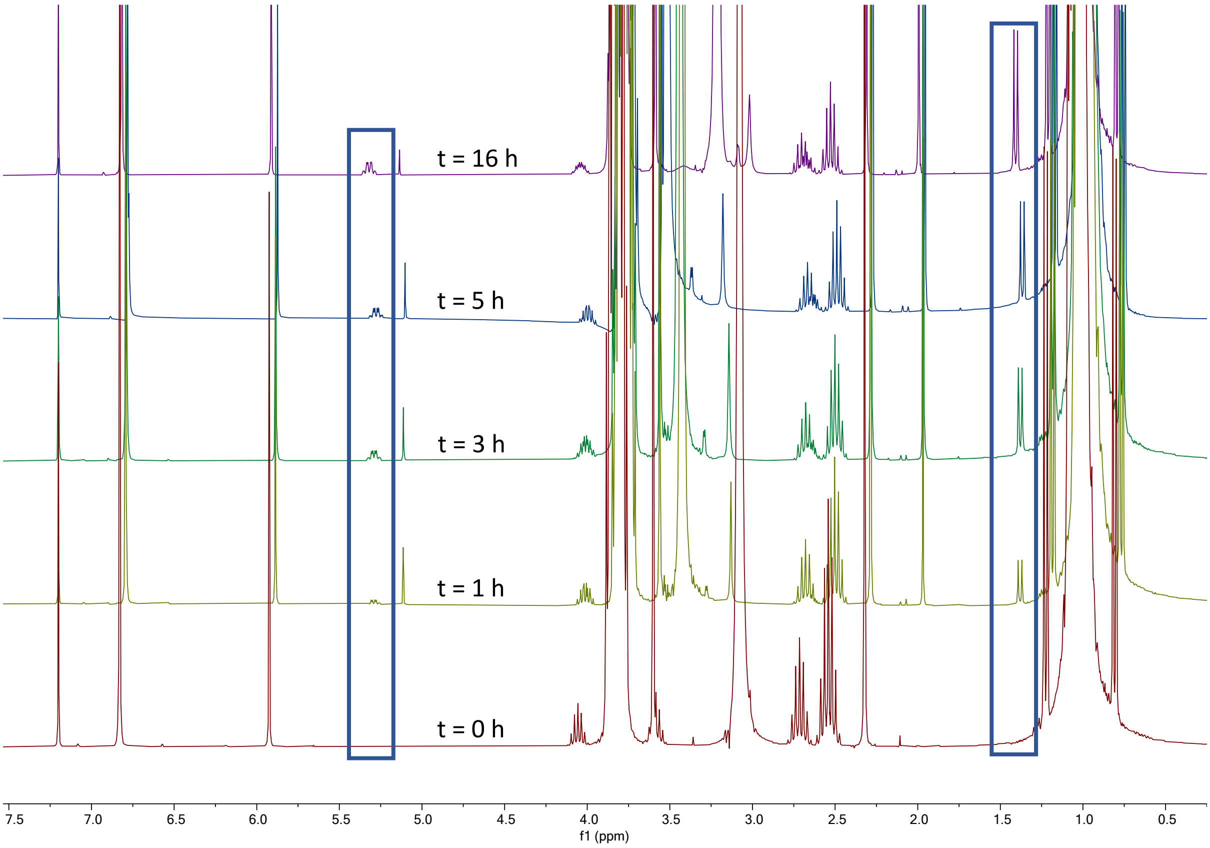

Figure S74: ^{1}H spectrum ($CDCl_3$, 298 K, 400 MHz) from the reduction of (**16a**) mediated by **Ru-2a** (1,3,5-trimethoxybenzene: 13 mg). Formation of characteristic signal for product formation is highlighted in blue.

Reduction of 2,4,6-tri-isopropylacetophenone (16a) – Catalyzed by Ru-2b

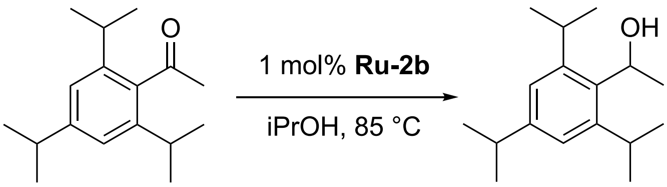

16a → **16b**

Time / h	Yield / %
1	14
3	21
16	38

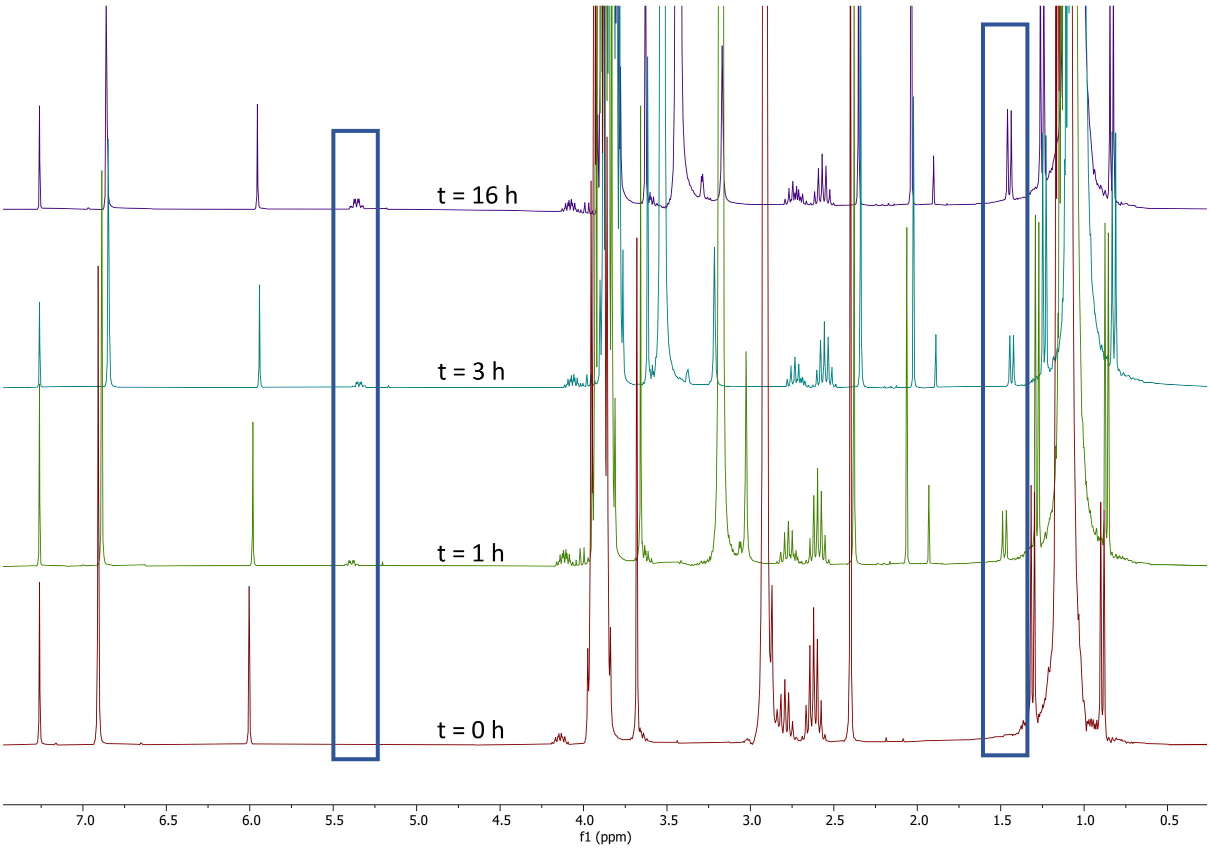

Figure S75: ^{1}H spectrum ($CDCl_3$, 298 K, 400 MHz) from the reduction of (**16a**) mediated by **Ru-2b** (1,3,5-trimethoxybenzene: 8 mg). Formation of characteristic signal for product formation is highlighted in blue.

Reduction of 2,4,6-tri-isopropylacetophenone (16a) – Catalyzed by Ru-4

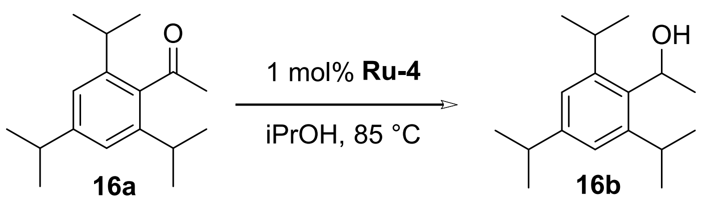

Time / h	Yield / %
16	17

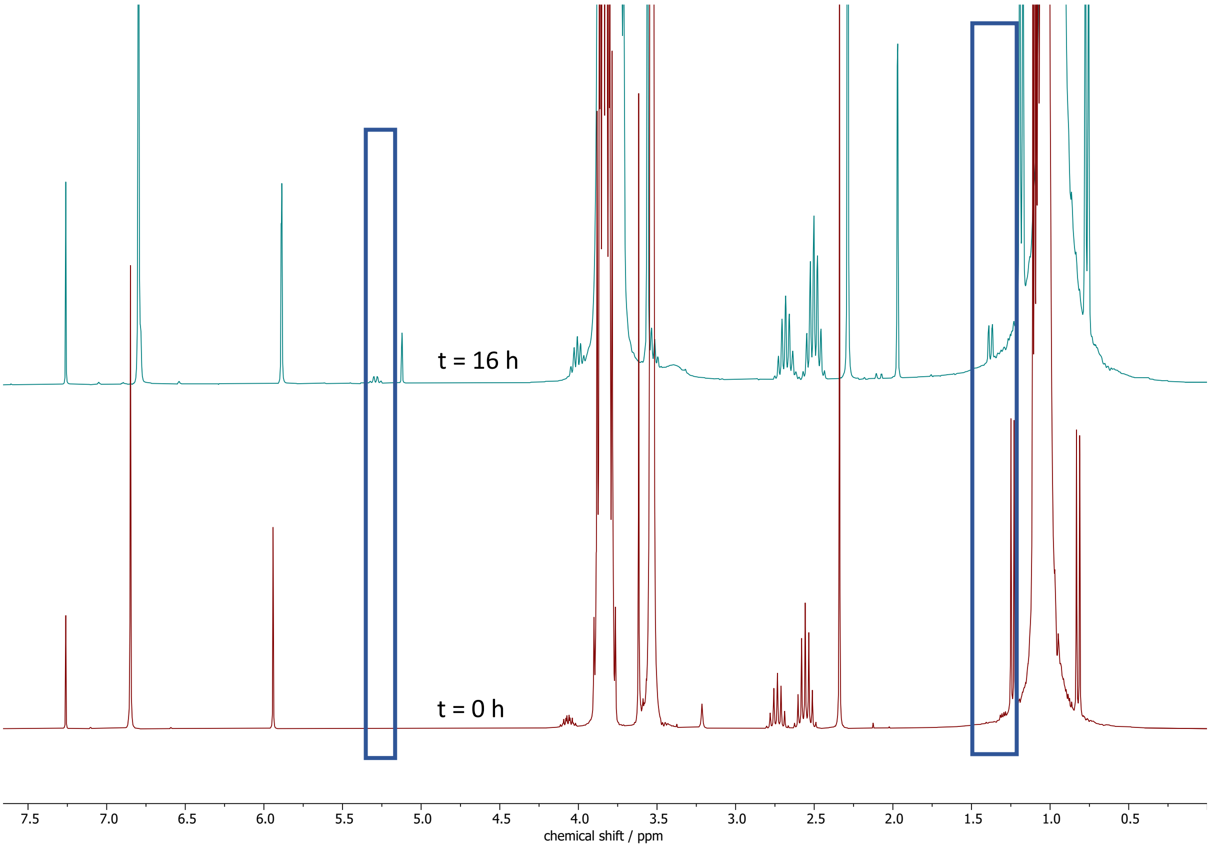

Figure S76: ^{1}H spectrum ($CDCl_3$, 298 K, 400 MHz) from the reduction of (**16a**) mediated by **Ru-4** (1,3,5-trimethoxybenzene: 10 mg). Formation of characteristic signal for product formation is highlighted in blue.

Reduction of 1-(adamantan-2-yl)ethan-1-one (17a) – Catalyzed by Ru-2a

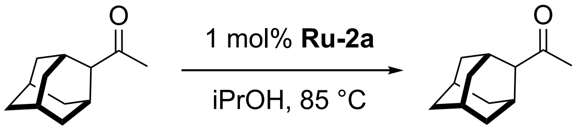

17a **17b**

Time / h	Yield / %
1	15
3	35
5	47
16	78
24	85

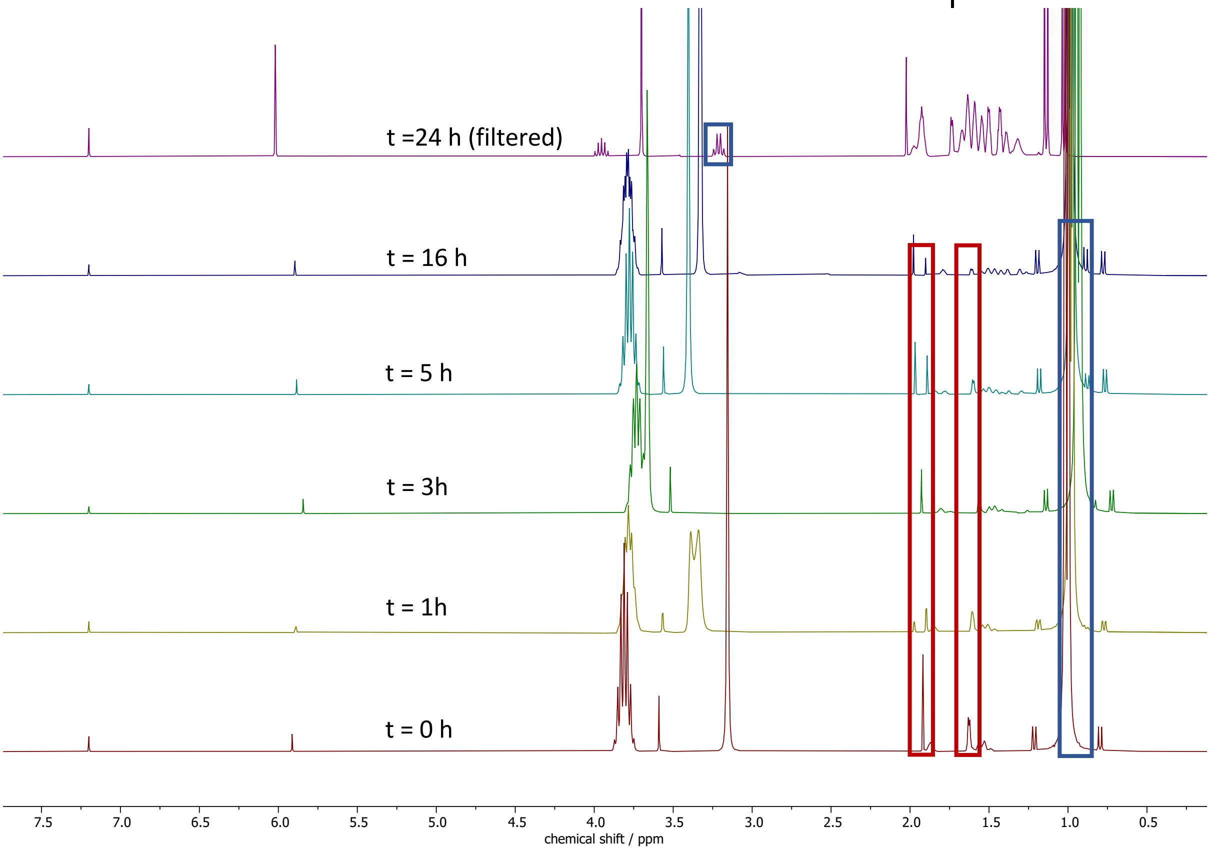

Figure S77: ^{1}H spectrum ($CDCl_3$, 298 K, 400 MHz) from the reduction of (**17a**) mediated by **Ru-2a** (1,3,5-trimethoxybenzene: 11 mg). Starting material consumption is highlighted in red and formation of characteristic signal for product formation is highlighted in blue.

Reduction of 1-(adamantan-2-yl)ethan-1-one (17a) – Catalyzed by Ru-2b

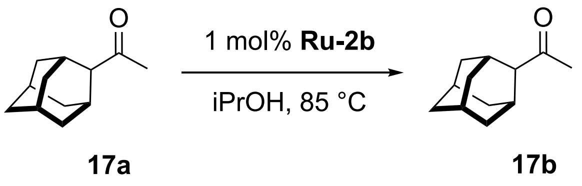

Time / h	Yield / %
1	19
2	31
16	46

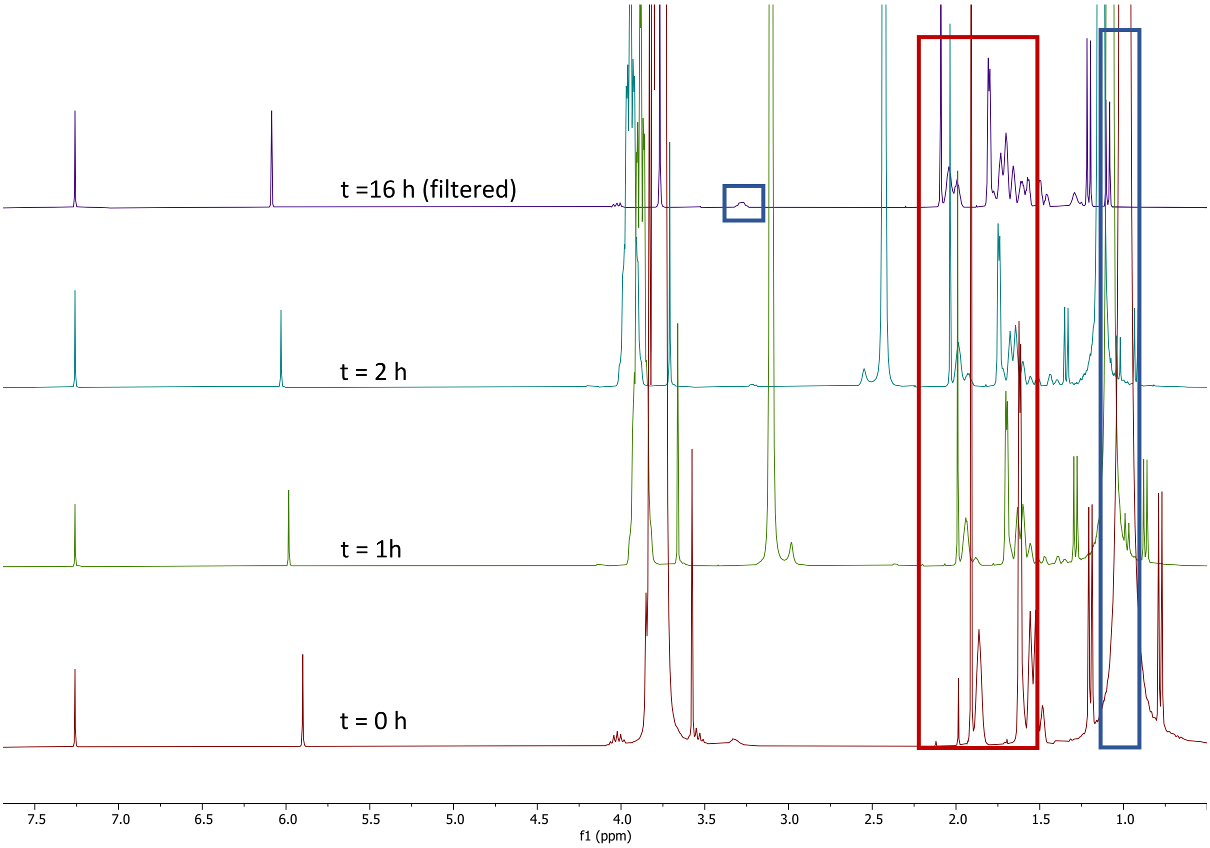

Figure S78: ^{1}H spectrum ($CDCl_3$, 298 K, 400 MHz) from the reduction of (**17a**) mediated by **Ru-2b** (1,3,5-trimethoxybenzene: 10 mg Starting material consumption is highlighted in red and formation of characteristic signal for product formation is highlighted in blue.

Reduction of 3-methyl-1-phenylbutan-1-one (18a) – Catalyzed by Ru-2a

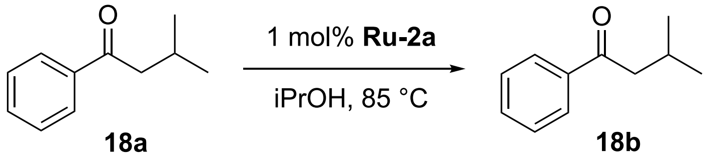

Time / h	Yield / %
1	60
2	86
3	95
5	>95

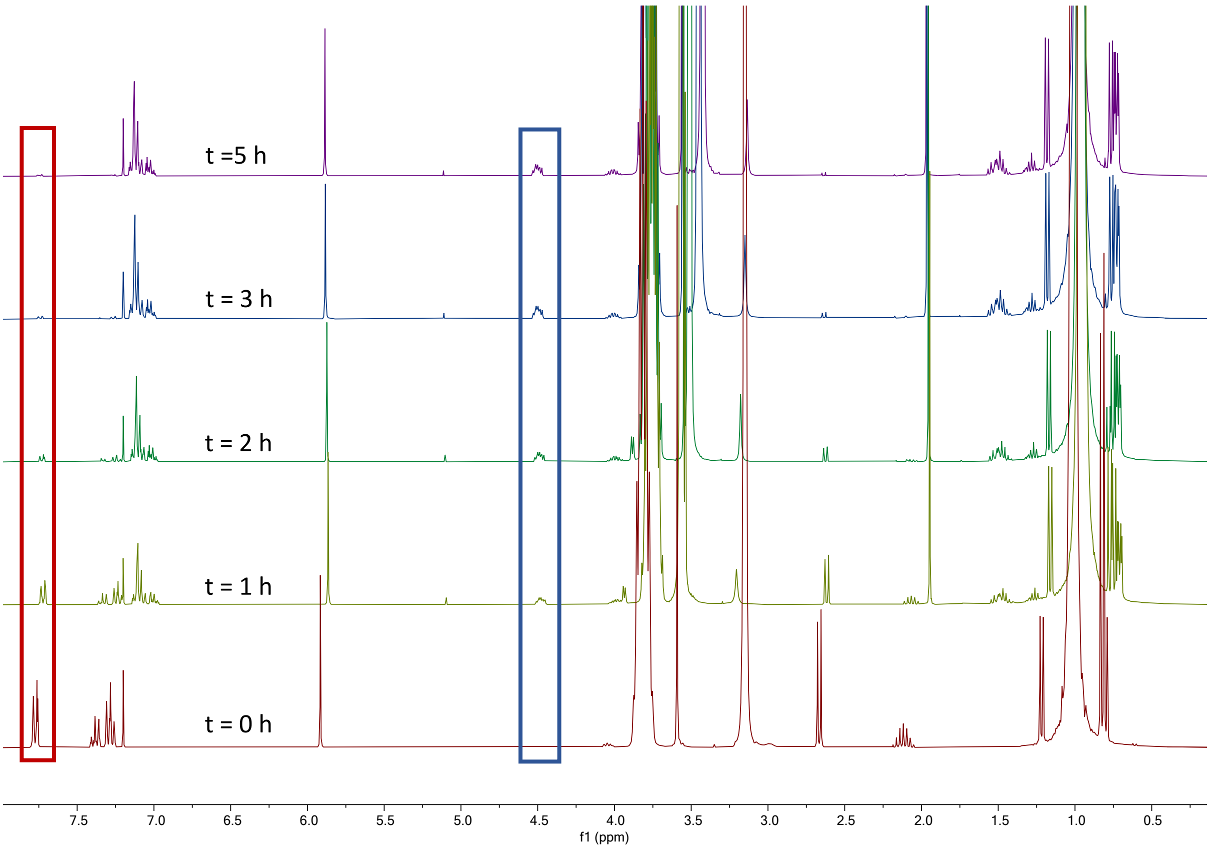

Figure S79: ^{1}H spectrum ($CDCl_3$, 298 K, 400 MHz) from the reduction of (**18a**) mediated by **Ru-2a** (1,3,5-trimethoxybenzene: 17 mg). Starting material consumption is highlighted in red and formation of characteristic signal for product formation is highlighted in blue.

Reduction of 3-methyl-1-phenylbutan-1-one (18a) – Catalyzed by Ru-2b

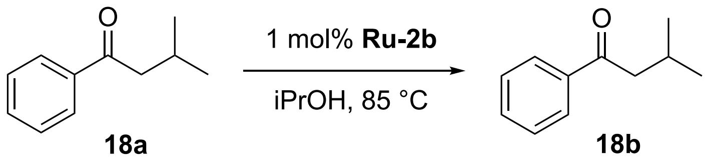

Time / h	Yield / %
1	90
3	94
5	>95

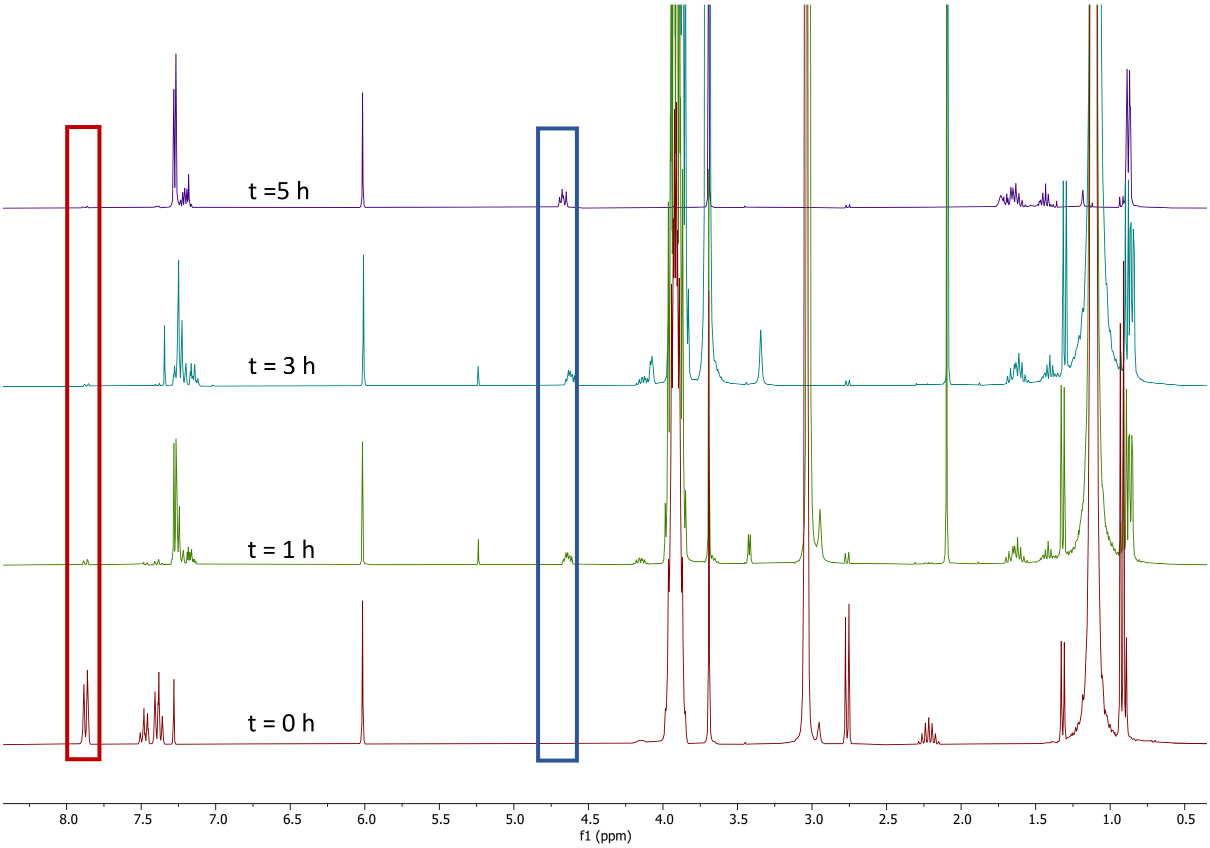

Figure S80: ^{1}H spectrum ($CDCl_3$, 298 K, 400 MHz) from the reduction of (**18a**) mediated by **Ru-2b** (1,3,5-trimethoxybenzene: 16 mg). Starting material consumption is highlighted red and formation of characteristic signal for product formation is highlighted in blue.

Reduction of 3-methyl-1-phenylbutan-1-one (18a) – Catalyzed by Ru-4

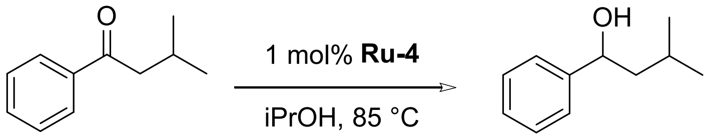

18a **18b**

Time / h	Yield / %
3	20

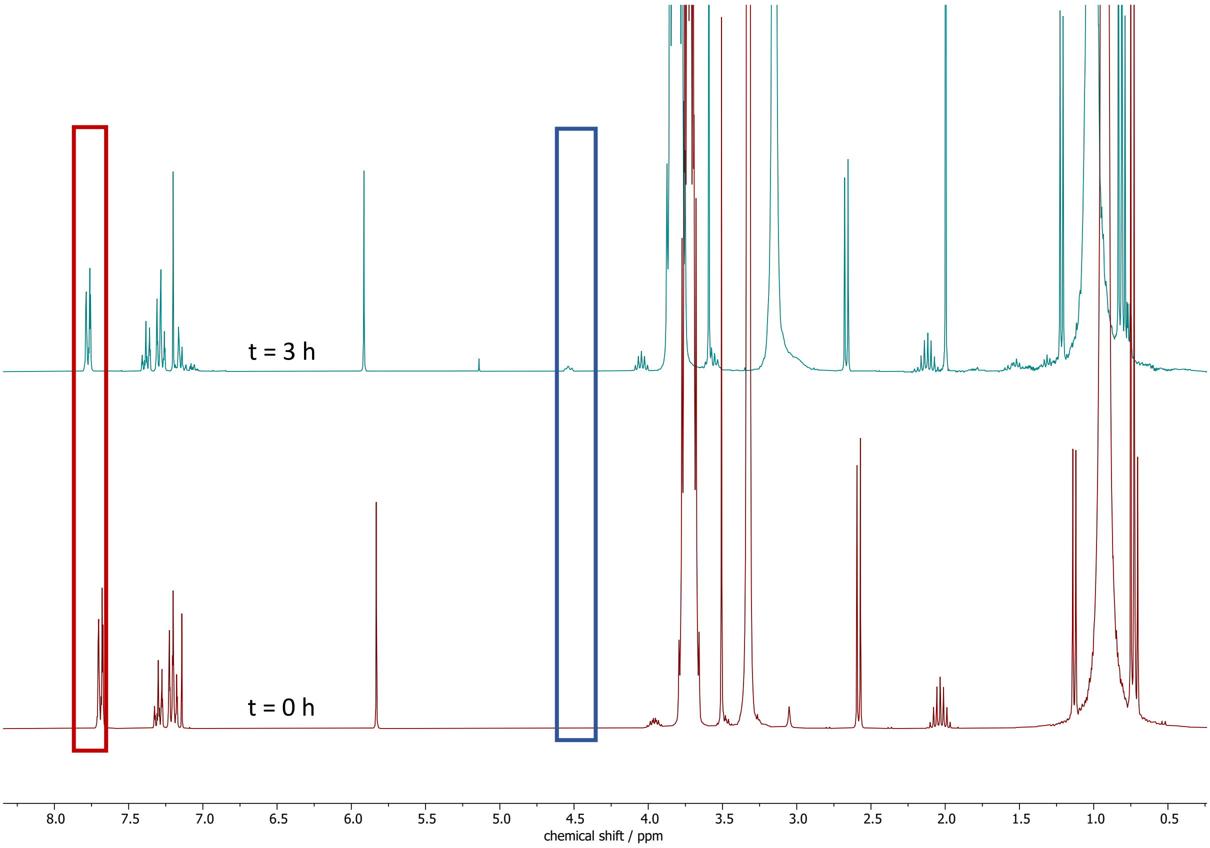

Figure S81: ^{1}H spectrum ($CDCl_3$, 298 K, 400 MHz) from the reduction of (**18a**) mediated by **Ru-4b** (1,3,5-trimethoxybenzene: 12 mg). Starting material consumption is highlighted red and formation of characteristic signal for product formation is highlighted in blue.

Reduction of citronellal (19a) – Catalyzed by Ru-2a

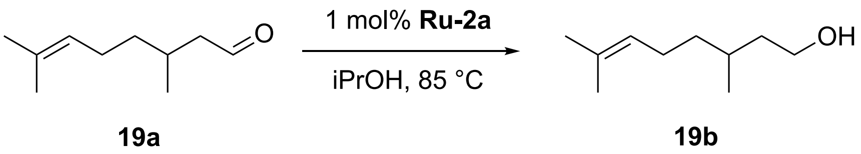

Time / h	Yield / %
16	45

Spectroscopic conversion 62% based on internal standard from **19a** (44.2 mg, 0.287 mmol) and **Ru-2a** (1.4 mg, 2.9 µmol) and hexamethylbenzene (8 mg, internal standard). The crude product was filtered over silica using Et_2O as eluent and subsequently evaporated to dryness, yielding **19b** (20.17 mg, 45%).

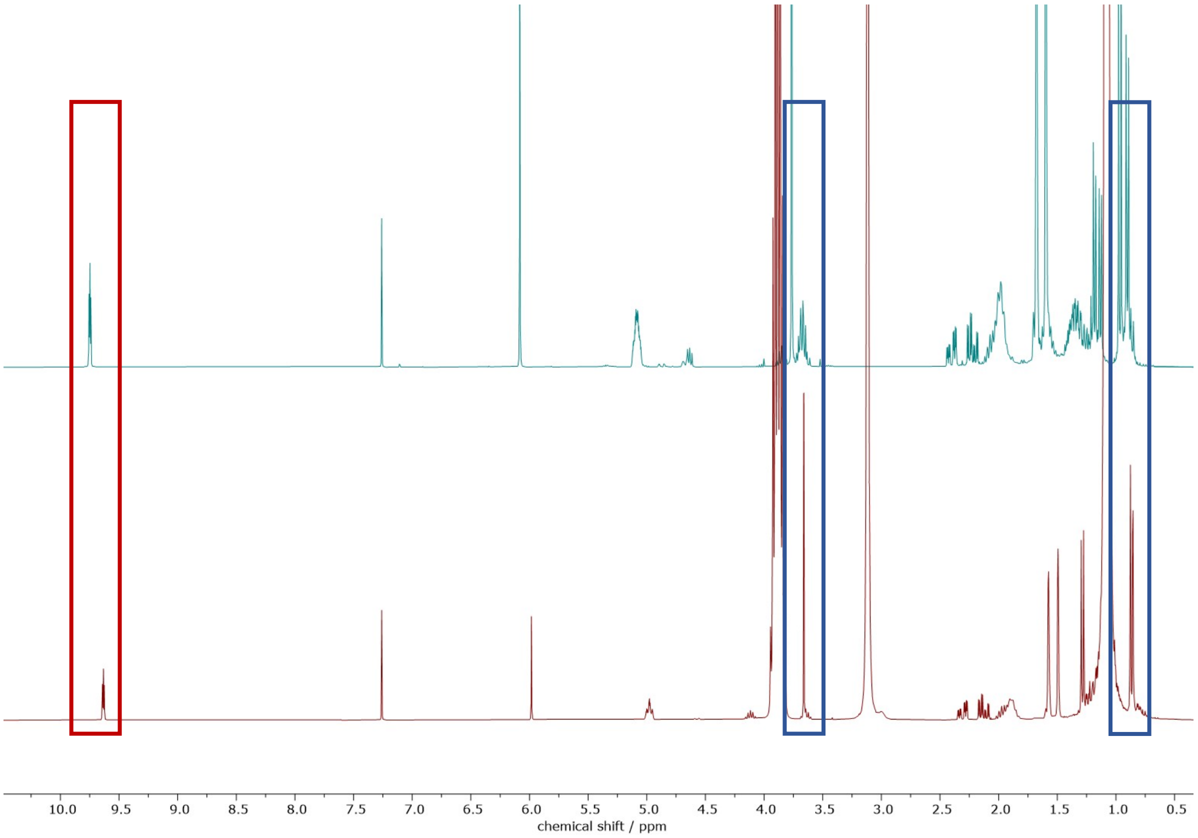

Figure S82: ^{1}H spectrum ($CDCl_3$, 298 K, 400 MHz) from the reduction of (**19a**) mediated by **Ru-2a** (1,3,5-trimethoxybenzene: 7 mg). Product formation (**19b**) is highlighted by the appearance of the multiplet (δ = 3.5 ppm) which is characteristic for H-α.

GC-MS Calc. for $C_{10}H_{19}O$ $[M-H]^+$, Calc. for 155.1430. Found 155.1433.

Reduction of 17α-Methylandrostan-17β-ol-3-one (20a) – Catalyzed by Ru-2a

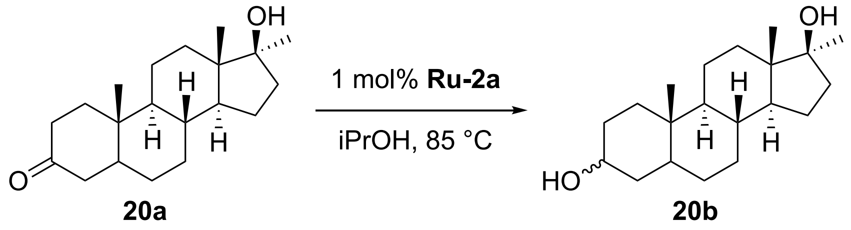

Time / h	Yield / %
5	80

Yield is isolated yield. The crude product was filtered over silica using Et_2O as eluent and was not further purified yielding 140 mg (80%) of **20b**. Reaction was performed with 0.574 mmol (174 mg) of **20a** and 5.74 µmol (2.81 mg) of **Ru-2a**

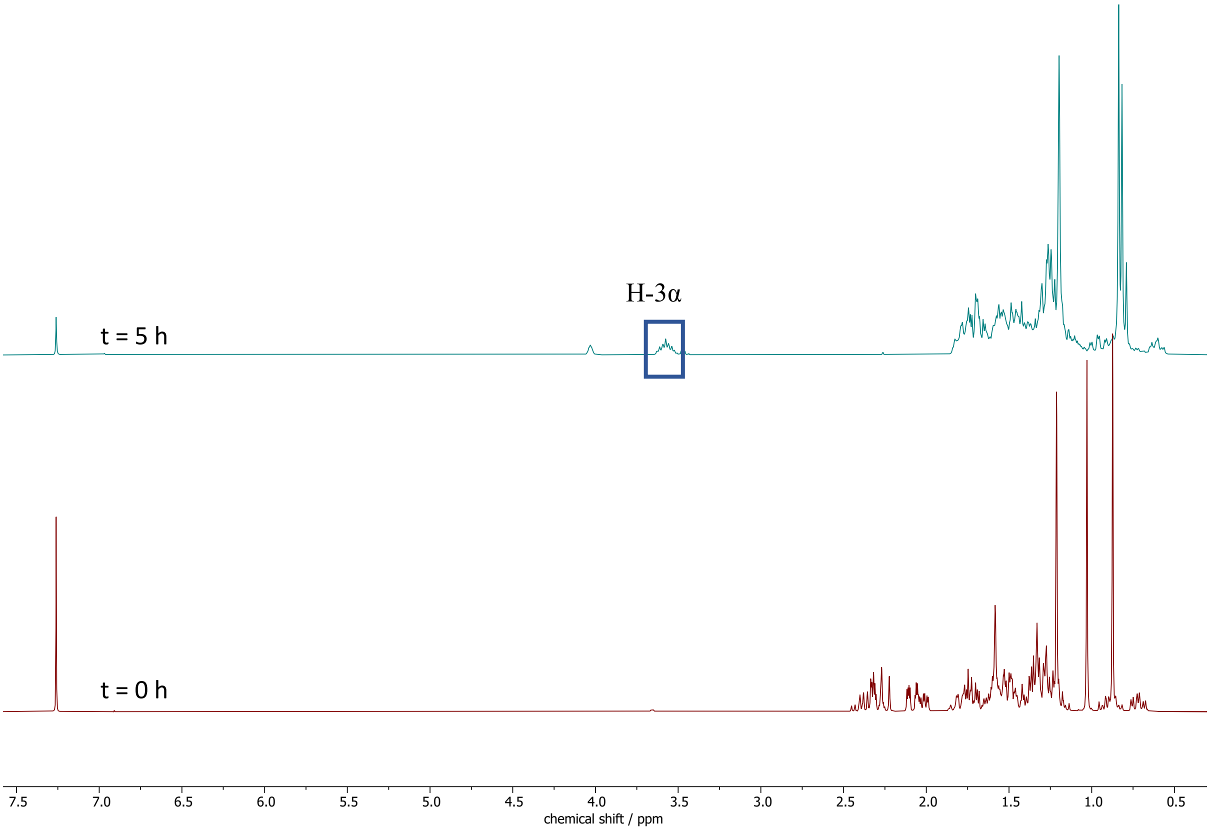

Figure S83: ^{1}H spectrum ($CDCl_3$, 298 K, 400 MHz) from the reduction of (**20a**) mediated by **Ru-2a** (1,3,5-trimethoxybenzene: 7 mg). Product formation (**20b**) is highlighted by the appearance of the multiplet (δ = 3.58 ppm) which is characteristic for H-3α and indicates that the C-3 carbonyl group was reduced.[S2,S3]

HR-MS Calcd. for $C_{20}H_{34}O_2$ $[M]^+$ 306.2553. Found: 306.2558.

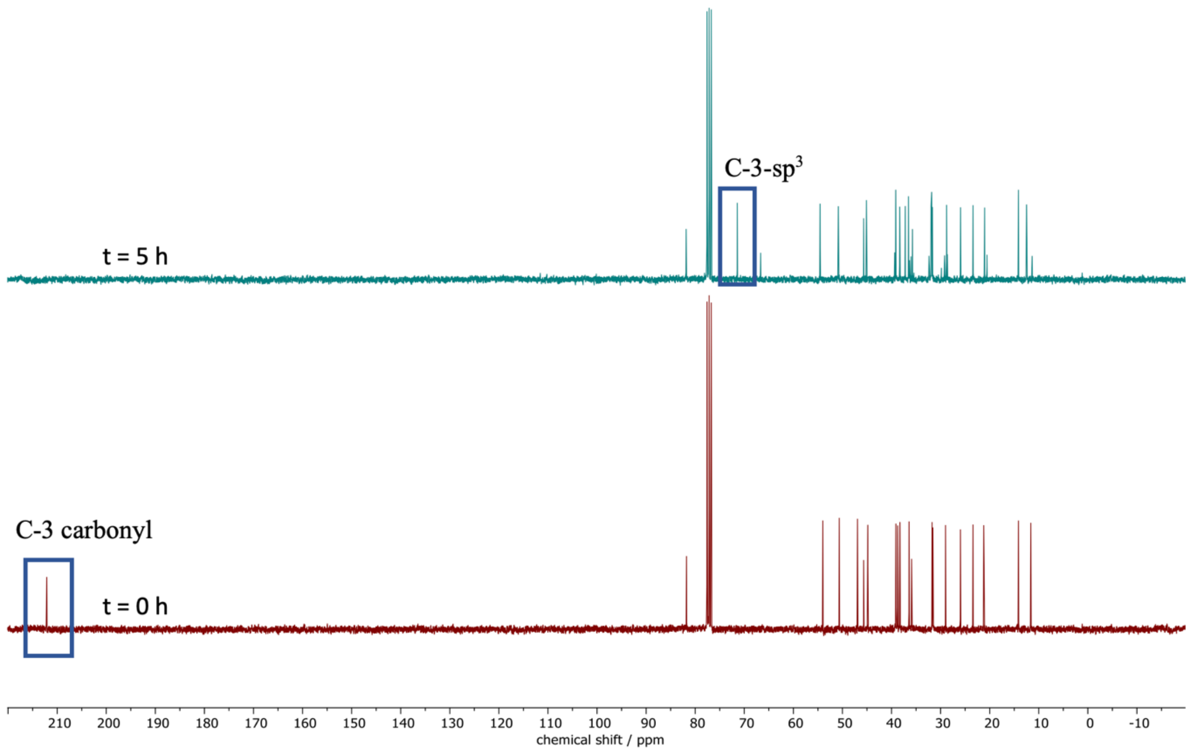

Figure S84: $^{13}C\{^{1}H\}$ spectrum ($CDCl_3$, 298 K, 75 MHz) from the reduction of (**20a**) mediated by **Ru-2a** (1,3,5-trimethoxybenzene: 7 mg). Product formation (**20b**) is indicated by the appearance of a new signal at δ = 71.41 ppm representing the newly formed sp^3-C-3 along with the disappearance of the carbonyl resonance at 212 ppm.

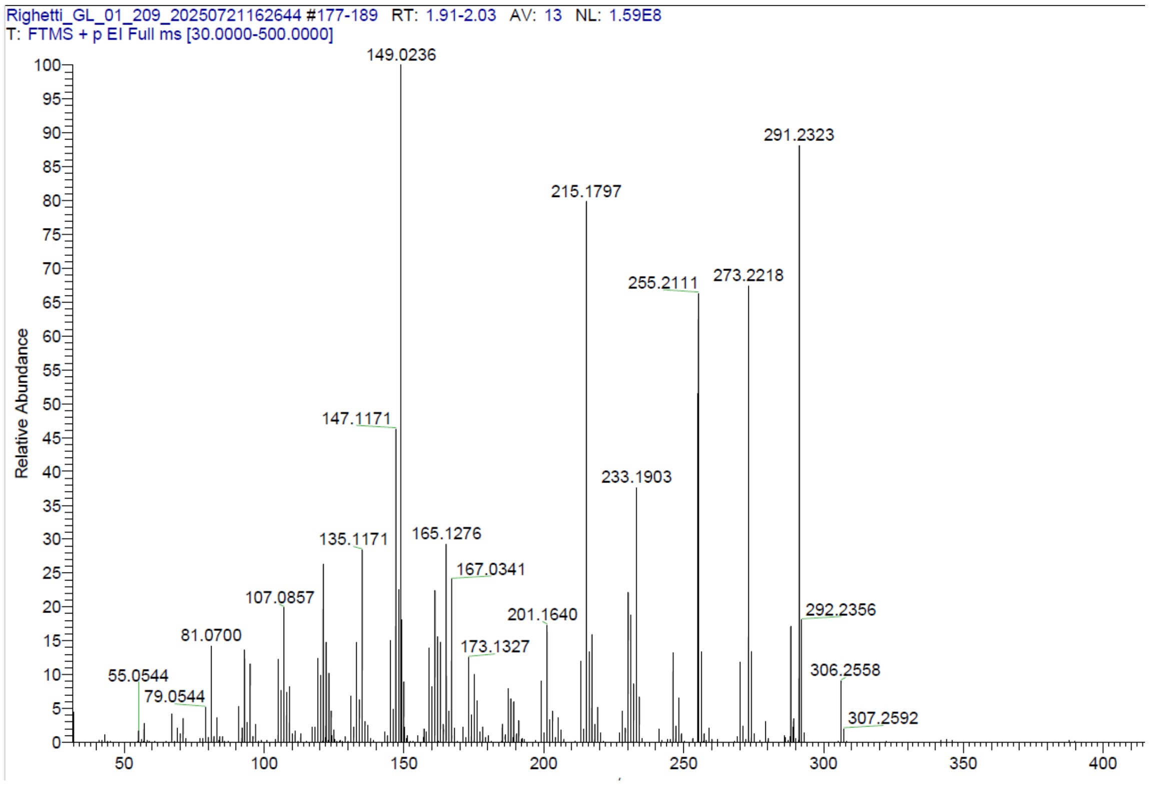

Figure S85: FTMS + p EI Full ms spectrum of isolated **20b**.

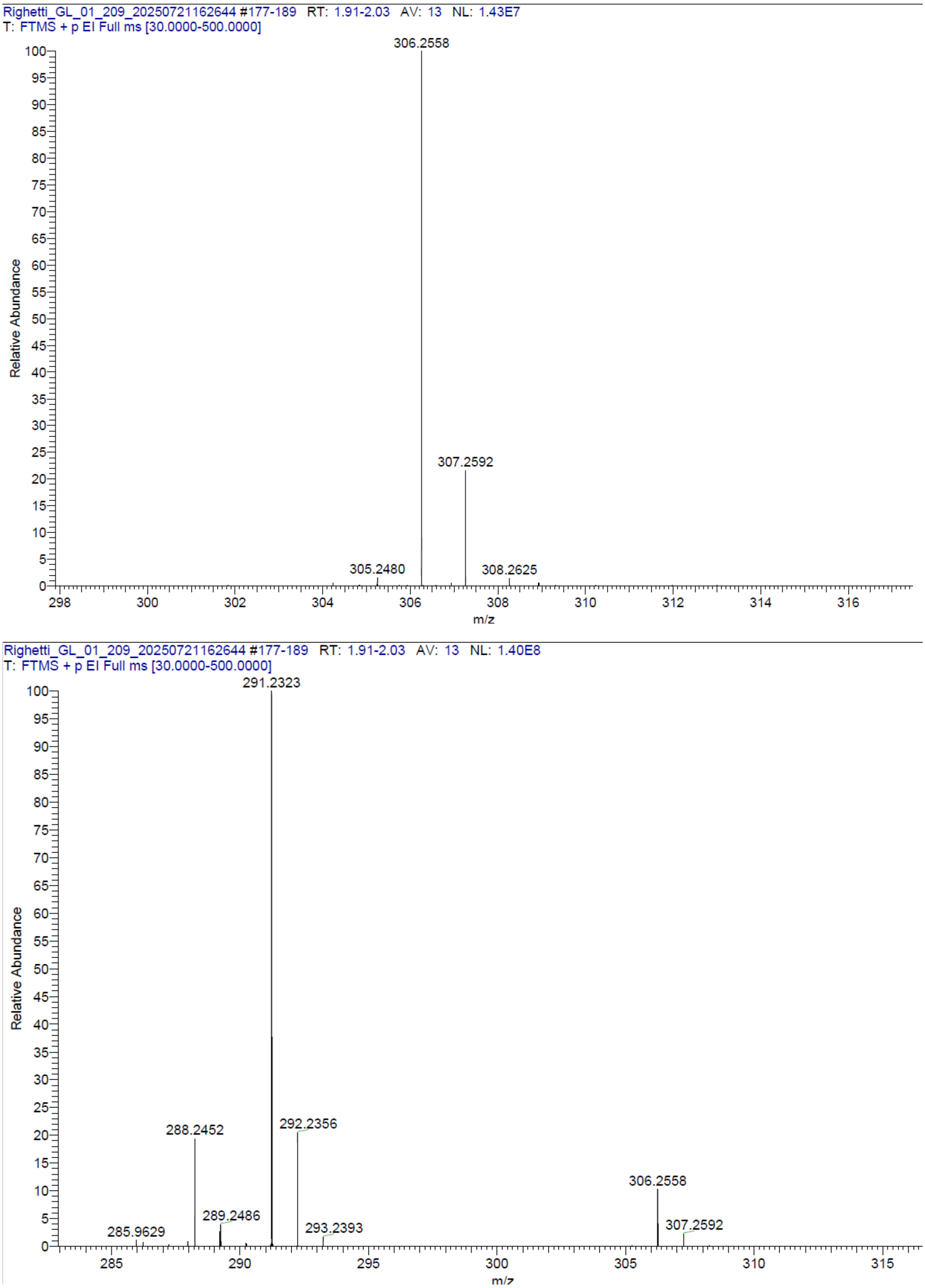

Figure S86: FTMS + p EI full ms spectrum (selected peaks) of isolated **20b**.

Time Conversion Profile – Different Catalysts

All the following reactions have been performed according to the general procedure of the catalytic experiments (see p. S43).

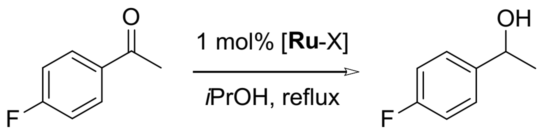

1a 1b

Table S1: Time/conversion data of the reduction of 4-fluoroacetophenone (**1a**) catalyzed by 1mol% **Ru-2a**, **Ru-2b**, **Ru-2c**, **Ru-2c** + Ag_2CO_3, **Ru-3** or **Ru-5;** Reaction conditions: 1 mol% **Ru-X**, 2 mL degassed iPrOH, reflux), 1.5 mol% Ag_2CO_3 (4.31 µmol, 1.19 mg) where indicated.

Time / min	Ru-2a	Ru-2b	Ru-2c	Ru-2c + Ag_2CO_3[a]	Ru-3	Ru-5
0	0	0	0	0	0	0
5	36	36	15	50	-	-
10	55	56	34	64	-	-
15	67	77	40	72	-	-
30	82	87	58	83	-	-
45	88	93	68	87	-	-
60	92	95	74	90	21	10
120	98	97	90	94	26	21
180	99	98	96	96	28	29

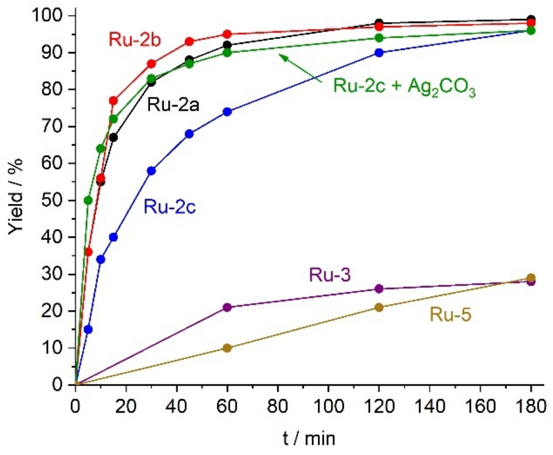

Figure S87: Time conversion profile from the reduction of 4-Fluoroacetophenone (**1a**) catalyzed by **Ru-2a** (black), **Ru-2b** (red), **Ru-2c** (blue), **Ru-2c** + Ag_2CO_3 (green), **Ru-3** (purple) and **Ru-5** (brown).

Reduction of 4-fluoroacetophenone (1a) – Catalyzed by Ru-2a

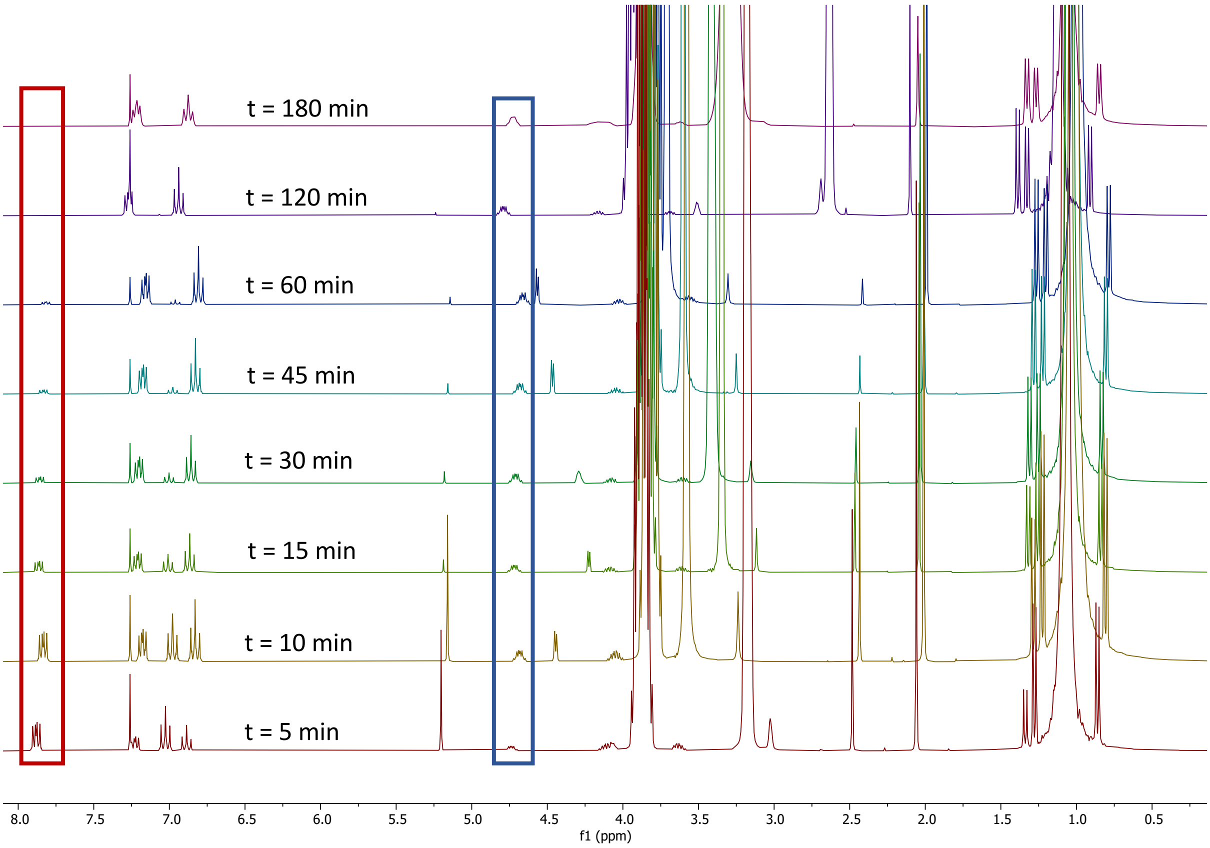

Figure S88: ^{1}H spectrum ($CDCl_3$, 298 K, 400 MHz) from the reduction of (**1a**) mediated by **Ru-2a**. Starting material consumption is highlighted in red and formation of characteristic signal for product formation is highlighted in blue.

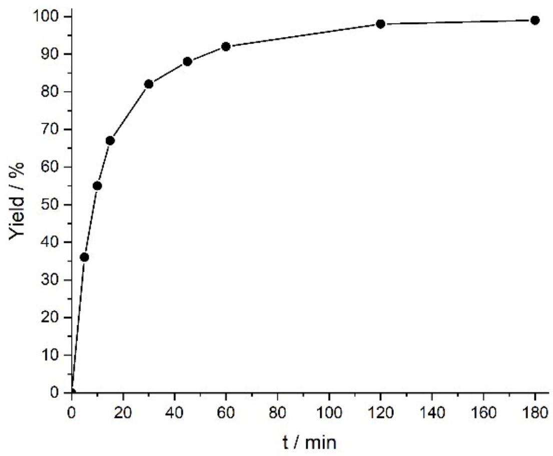

Figure S89: Time conversion profile from the reduction of 4-fluoroacetophenone (**1a**) catalyzed by **Ru-2a**.

Reduction of 4-fluoroacetophenone (1a) – Catalyzed by Ru-2b

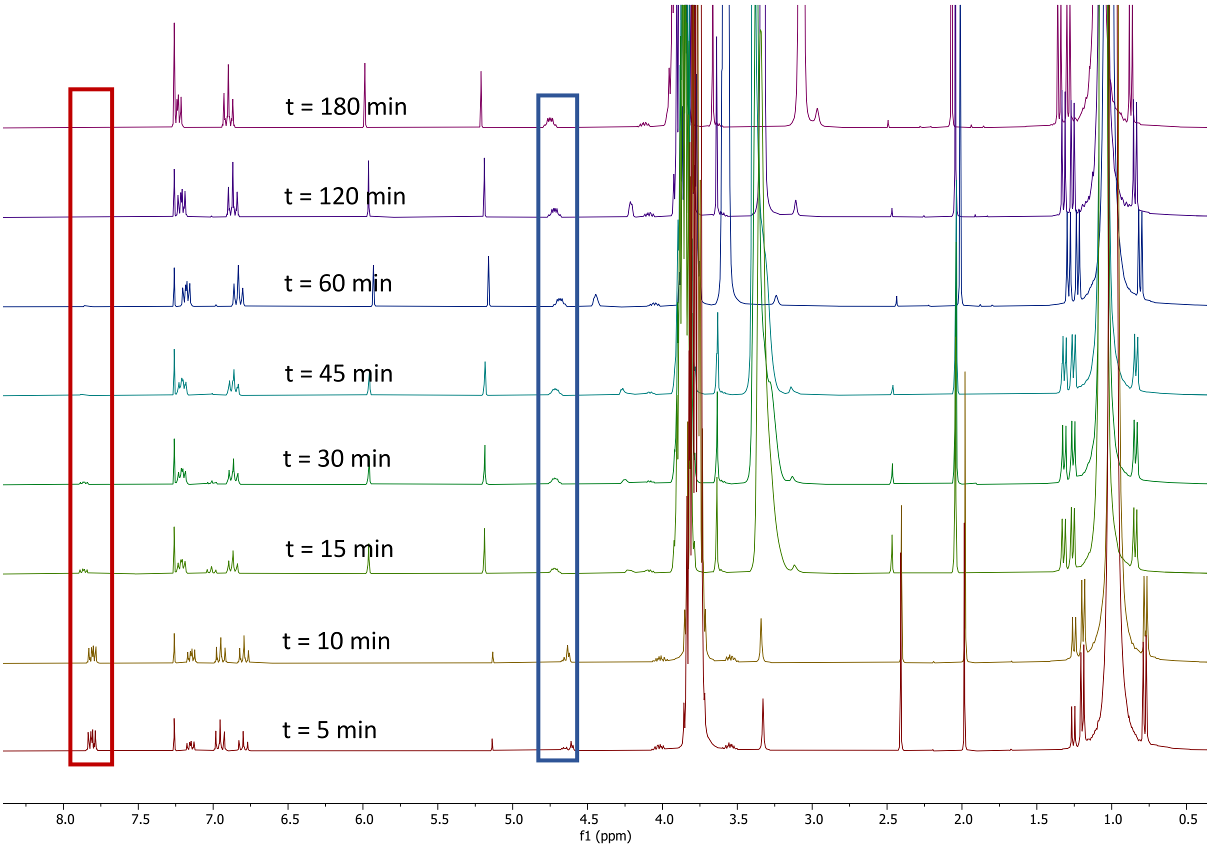

Figure S90: ^{1}H spectrum ($CDCl_3$, 298 K, 400 MHz) from the reduction of (**1a**) mediated by **Ru-2b**. Starting material consumption is highlighted in red and formation of characteristic signal for product formation is highlighted in blue.

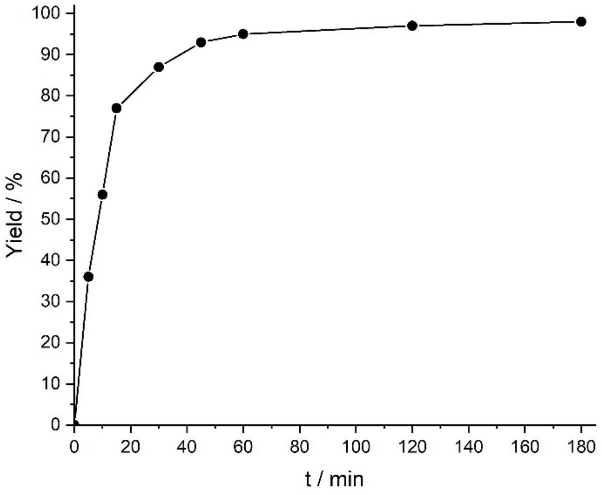

Figure S91: Time conversion profile from the reduction of 4-fluoroacetophenone (**1a**) catalyzed by **Ru-2b**.

Reduction of 4-fluoroacetophenone (1a) – Catalyzed by Ru-2c

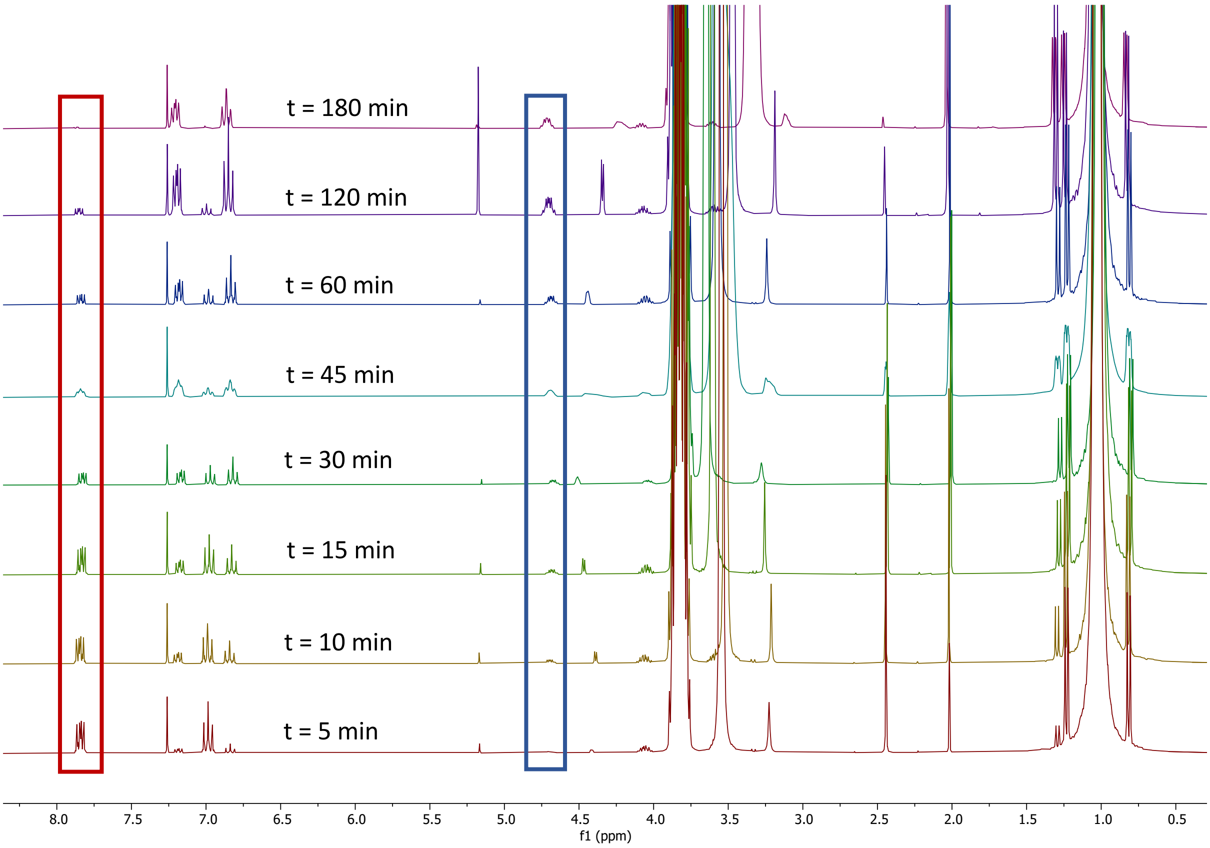

Figure S92: ^{1}H spectrum ($CDCl_3$, 298 K, 400 MHz) from the reduction of (**1a**) mediated by **Ru-2c**. Starting material consumption is highlighted in red and formation of characteristic signal for product formation is highlighted in blue.

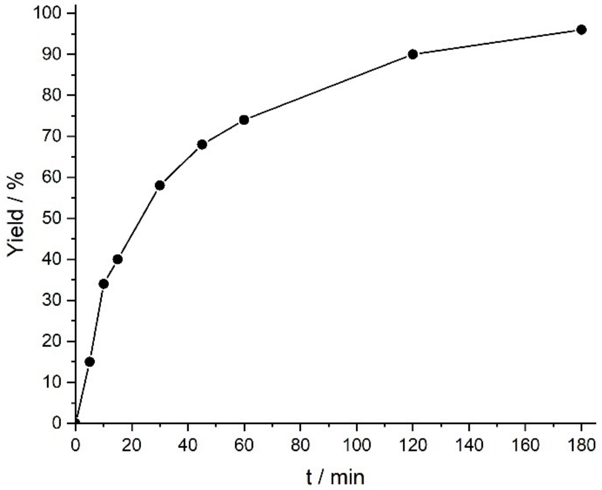

Figure S 93: Time conversion profile from the reduction of 4-fluoroacetophenone (**1a**) catalyzed by **Ru-2c**.

Reduction of 4-fluoroacetophenone (1a) – Catalyzed by Ru-2c + Ag_2CO_3

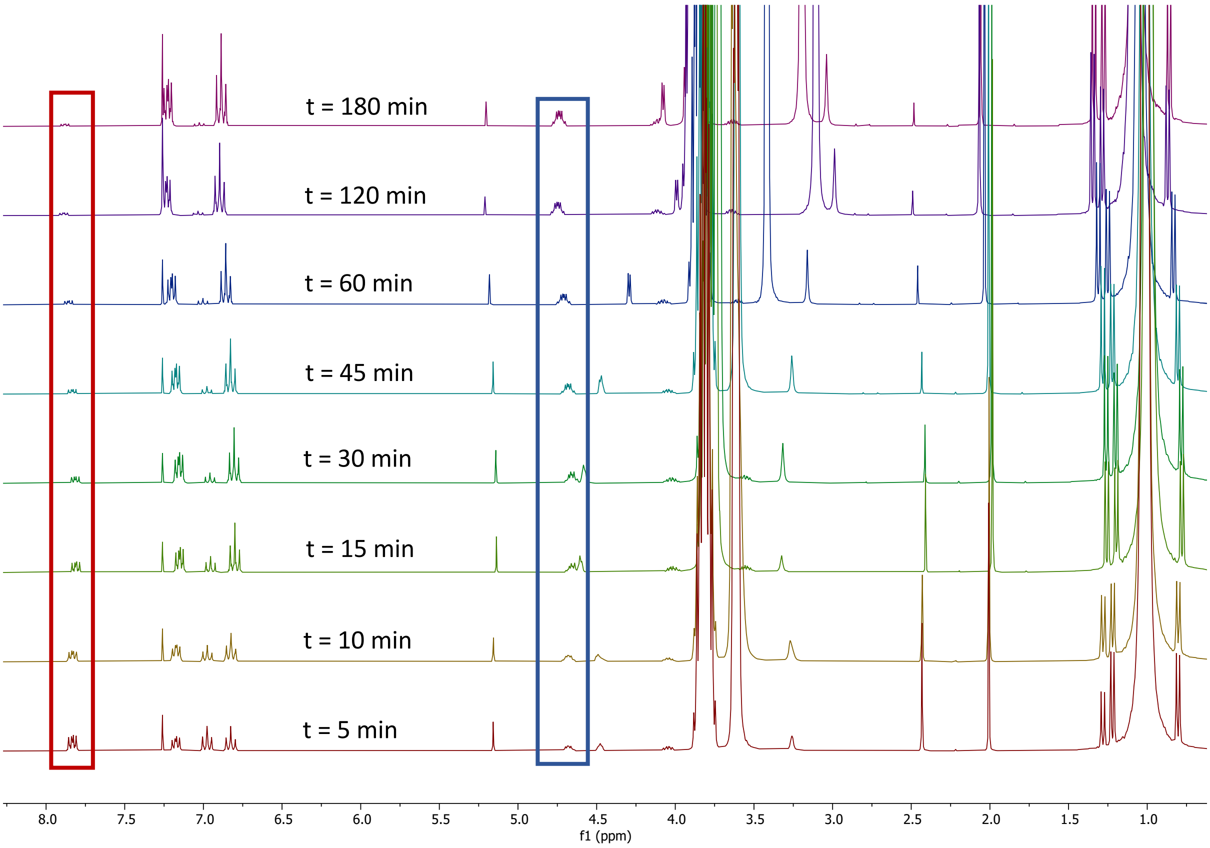

Figure S94: ^{1}H spectrum ($CDCl_3$, 298 K, 400 MHz) from the reduction of (**1a**) mediated by **Ru-2c** + Ag_2CO_3. Starting material consumption is highlighted in red and formation of characteristic signal for product formation is highlighted in blue.

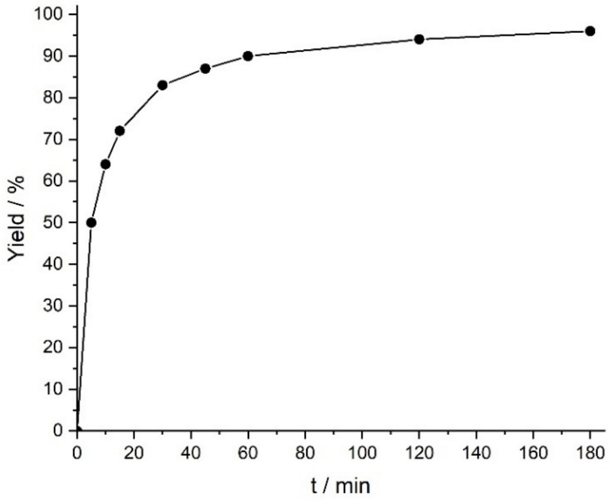

Figure S95: Time conversion profile from the reduction of 4-fluoroacetophenone (**1a**) catalyzed by **Ru-2c** + Ag_2CO_3.

Reduction of 4-fluoroacetophenone (1a) – Catalyzed by Ru-3

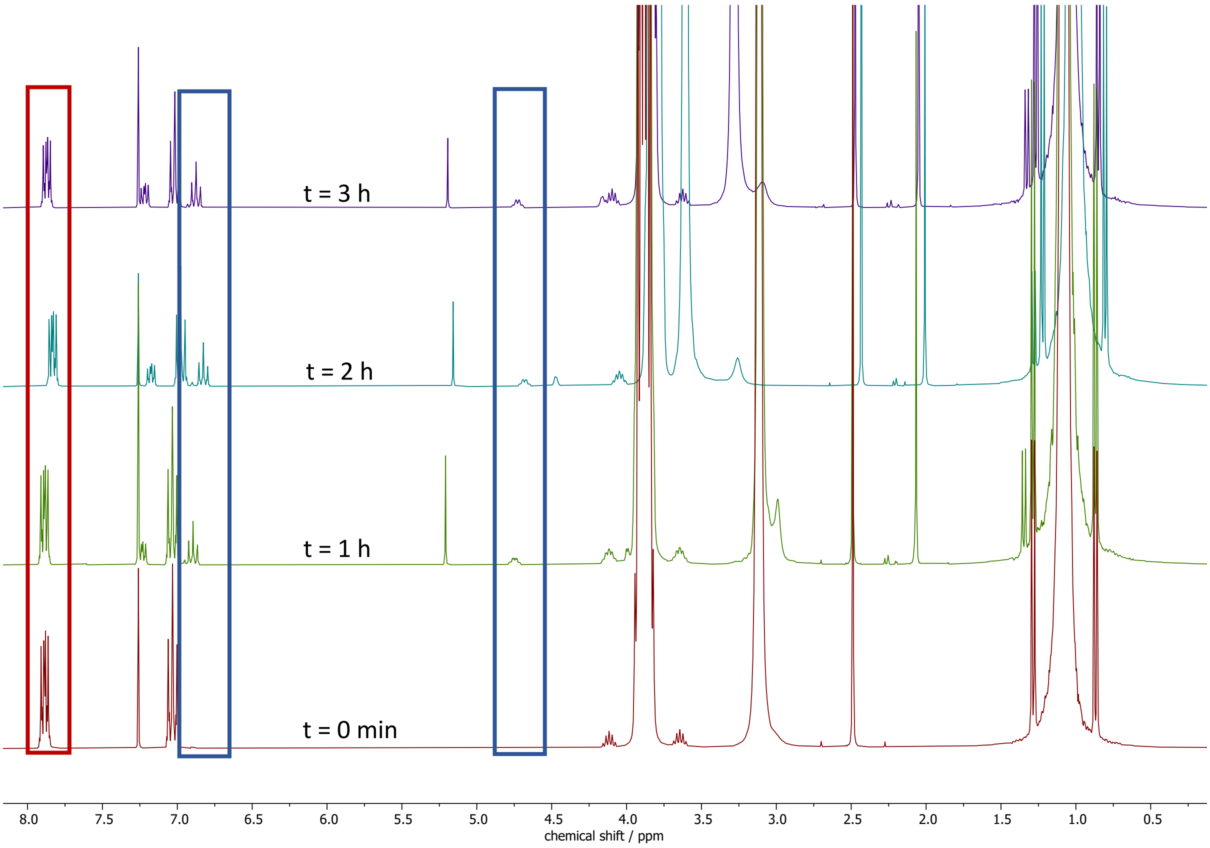

Figure S96: ^{1}H spectrum ($CDCl_3$, 298 K, 400 MHz) from the reduction of (**1a**) mediated by **Ru-3**. Starting material consumption is highlighted in red and formation of characteristic signal for product formation is highlighted in blue.

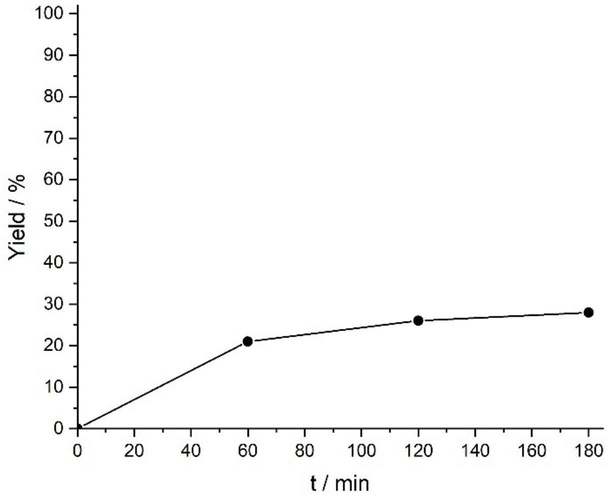

Figure S97: Time conversion profile from the reduction of 4-fluoroacetophenone (**1a**) catalyzed by **Ru-3**.

Reduction of 4-fluoroacetophenone (1a) – Catalyzed by Ru-5

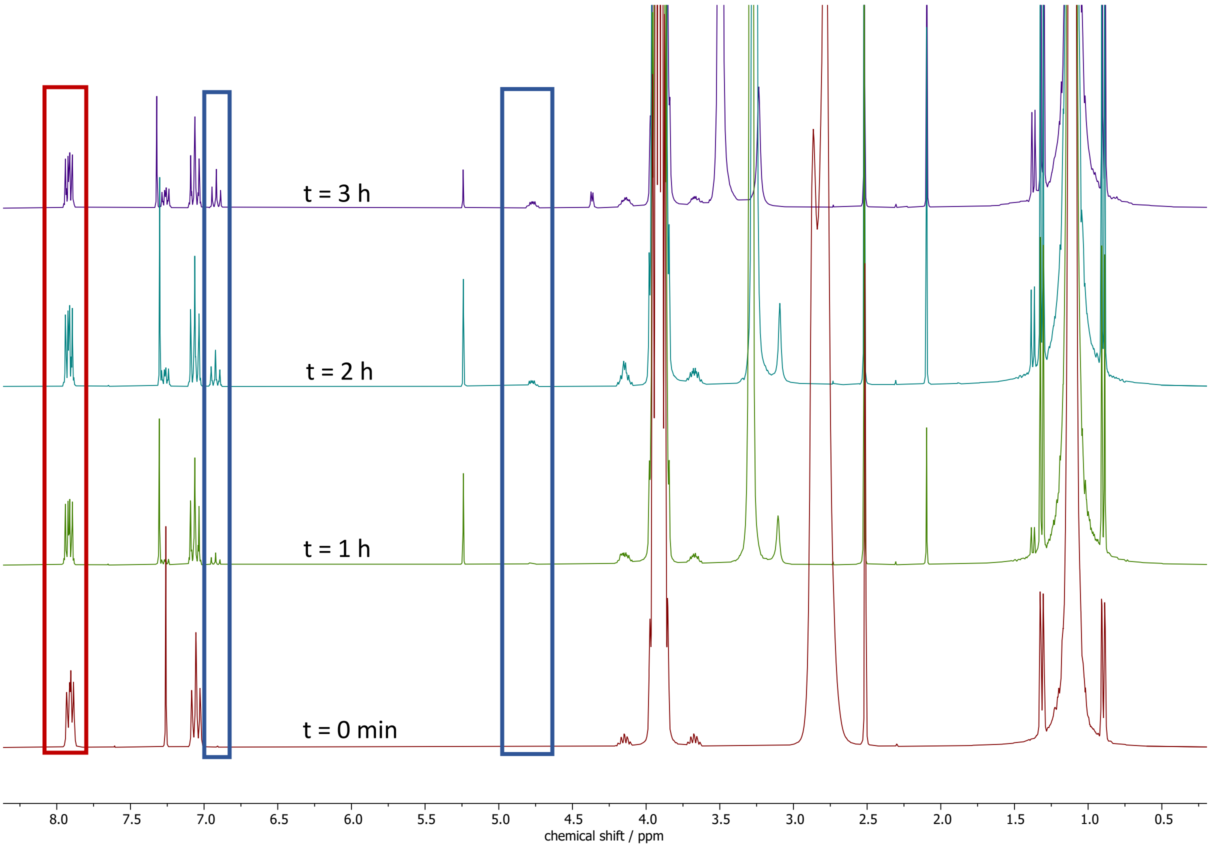

Figure S98: ^{1}H spectrum ($CDCl_3$, 298 K, 400 MHz) from the reduction of (**1a**) mediated by **Ru-5**. Starting material consumption is highlighted in red and formation of characteristic signal for product formation is highlighted in blue.

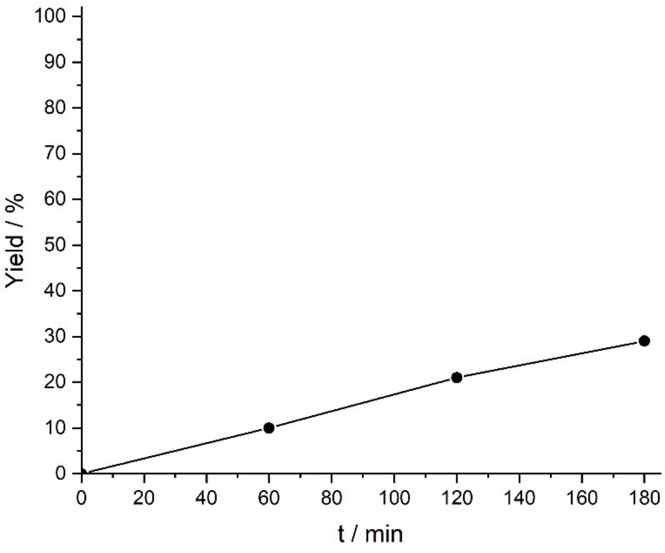

Figure S99: Time conversion profile from the reduction of 4-fluoroacetophenone (**1a**) catalyzed by **Ru-5**.

Temperature-dependent Experiments

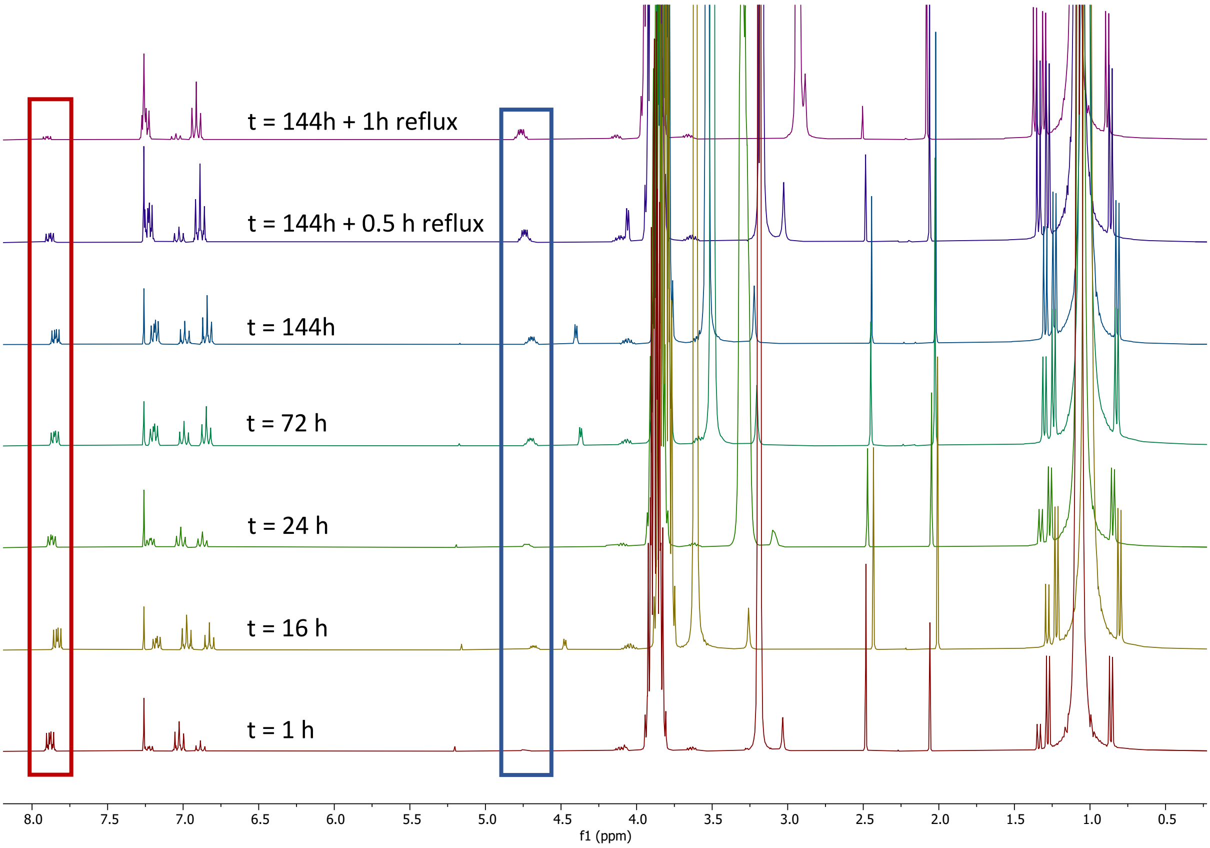

Figure S100: ^{1}H spectrum ($CDCl_3$, 298 K, 400 MHz) from the reduction of (**1a**) mediated by **Ru-2a** at 40 °C (for 144 h) and at reflux (subsequent 1 h). Starting material consumption is highlighted in red and formation of characteristic signal for product formation is highlighted in blue.

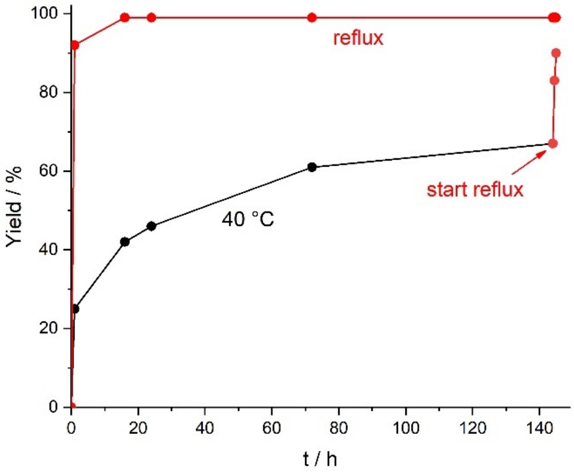

Figure S101: Time conversion profile from the reduction of 4-fluoroacetophenone (**1a**) catalyzed by **Ru-2a** at different temperatures (black: 40 °C and red: at reflux (85 °C).

Variation of Catalyst Loading

0.1 mol% Ru-2a

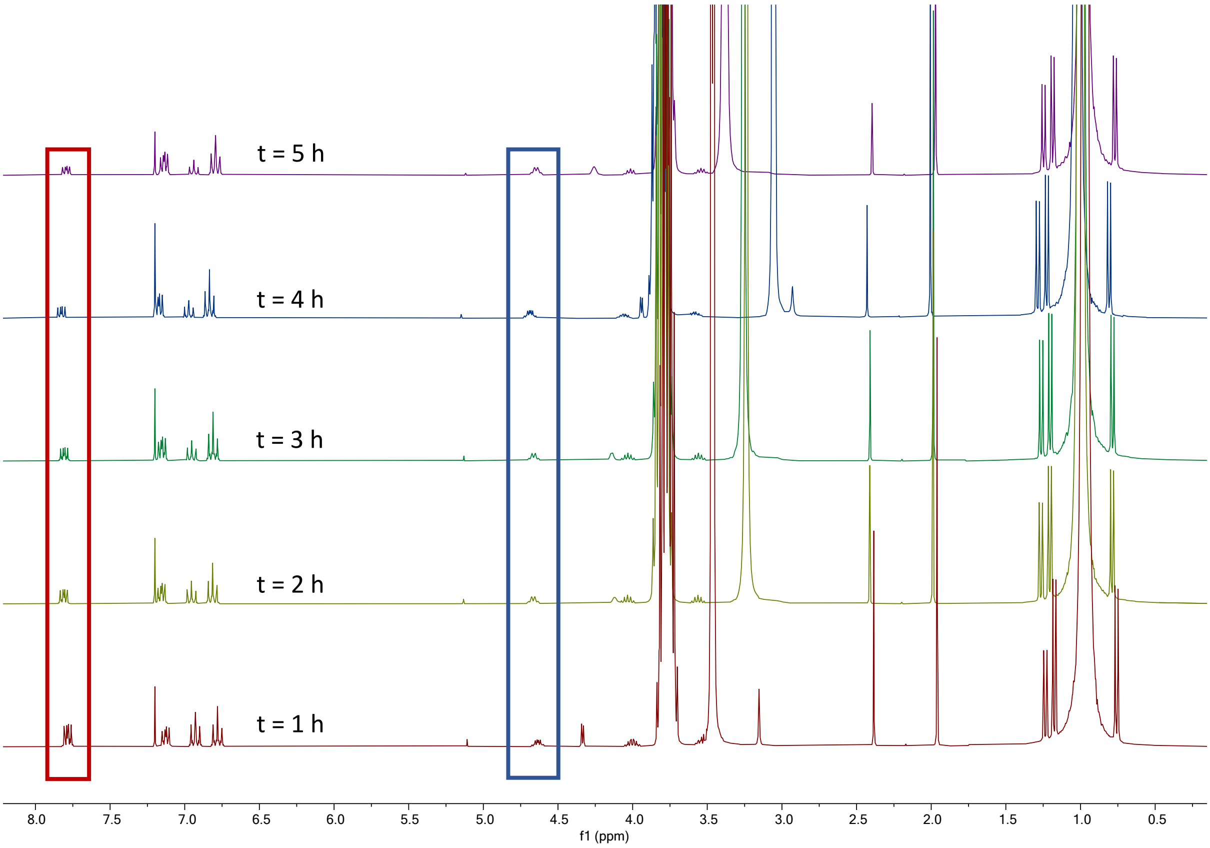

Figure S102: ^{1}H spectrum (CDCl₃, 298 K, 400 MHz) from the reduction of (**1a**) mediated by **Ru-2a** using 0.1 mol% catalyst loading. Starting material consumption is highlighted in red and formation of characteristic signal for product formation is highlighted in blue.

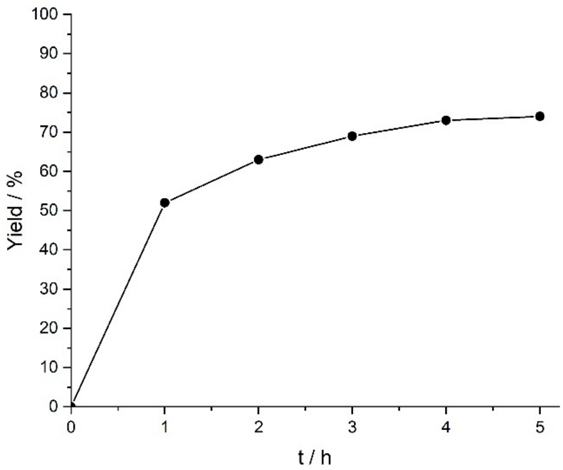

Figure S103: Time conversion profile from the reduction of 4-fluoroacetophenone (**1a**) catalyzed by 0.1 mol% (0.287 µmol) **Ru-2a**.

0.05 mol% Ru-2a

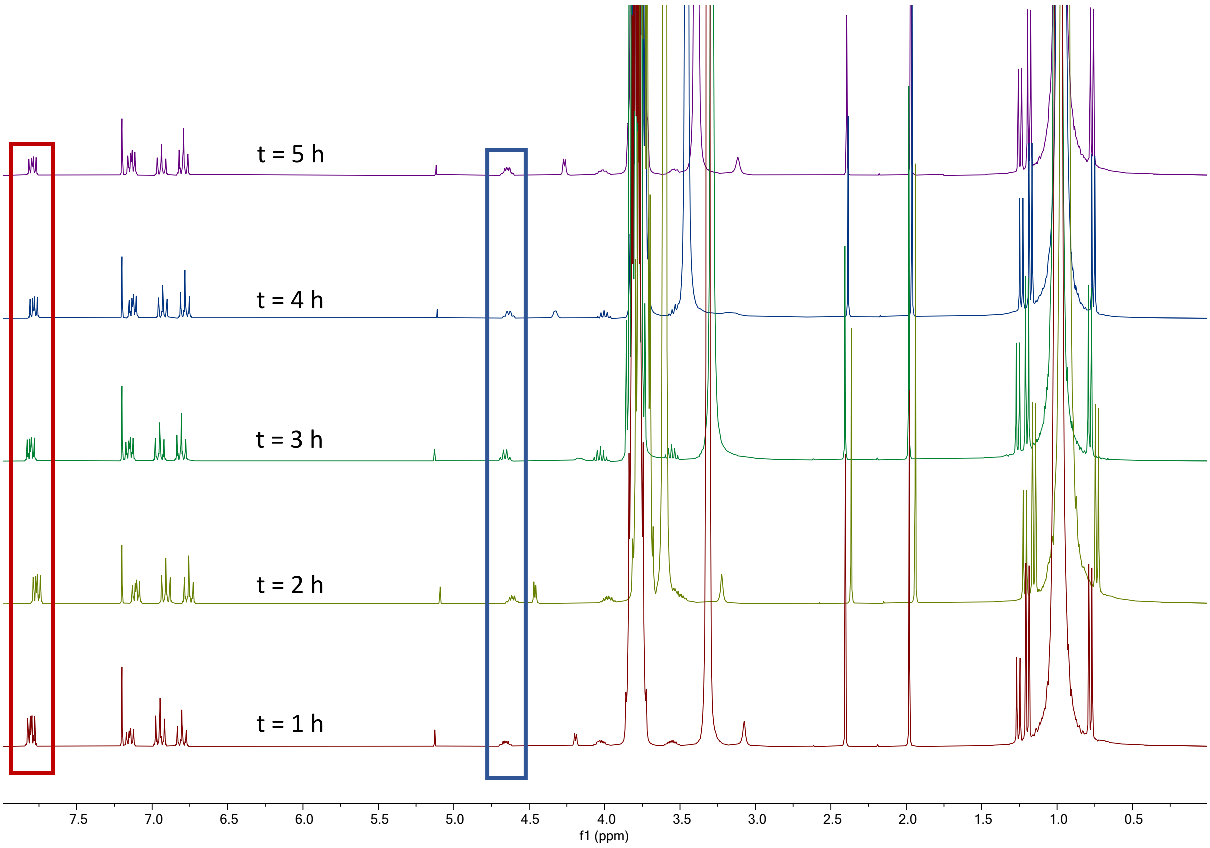

Figure S104: ^{1}H spectrum ($CDCl_3$, 298 K, 400 MHz) from the reduction of (**1a**) mediated by **Ru-2a** using 0.05 mol% catalyst loading. Starting material consumption is highlighted red and formation of characteristic signal for product formation is highlighted in blue.

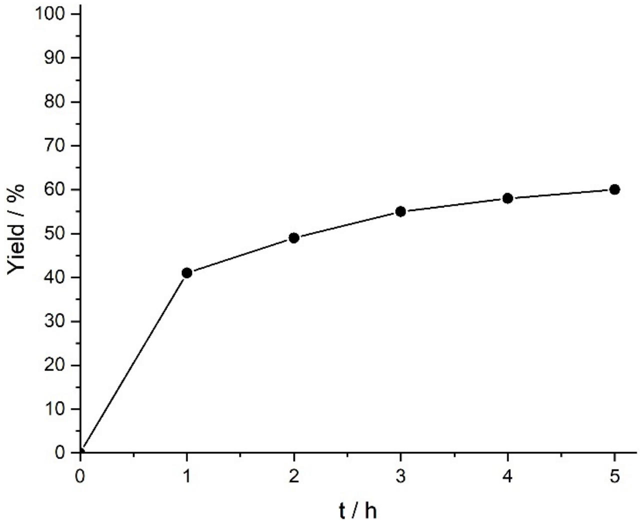

Figure S105: Time conversion profile from the reduction of 4-fluoroacetophenone (**1a**) catalyzed by 0.05 mol% (0.1435 µmol) **Ru-2a**.

Calculation of Relative Buried Volume (%V_{bur})

%V_{bur} of Ru-2a

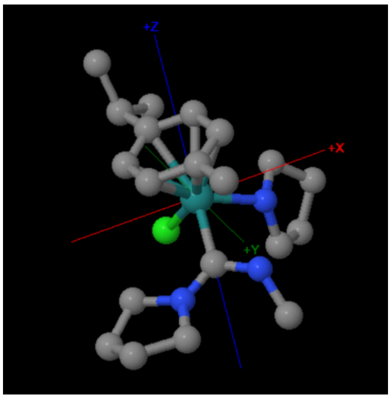

%V Free	%V Buried	% V Tot/V Ex
76.8	**23.2**	99.9

Quadrant	V f	V b	V t	%V f	%V b
SW	33.0	11.9	44.9	**73.5**	26.5
NW	33.4	11.5	44.9	**74.4**	25.6
NE	35.7	9.2	44.9	**79.6**	20.4
SE	35.7	9.1	44.9	**79.6**	20.4

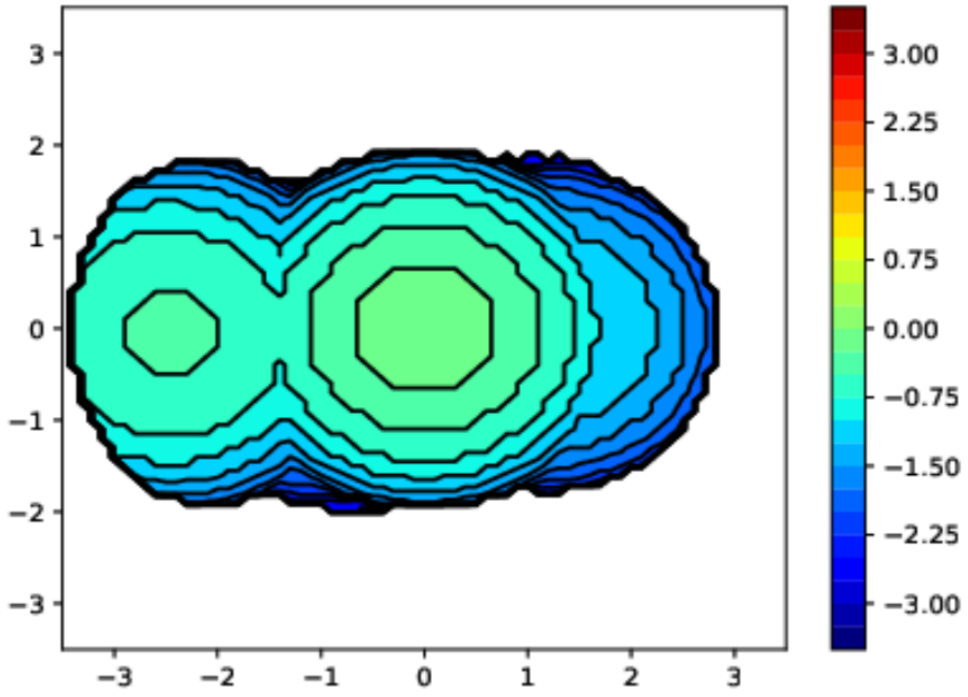

Figure S106: XRD structure with the specific orientation used to determine %V_{bur} (top left). Data obtained from calculating %V_{bur} (top right). Steric map of **Ru-2a** (bottom).

%V_{bur} of Ru-2b

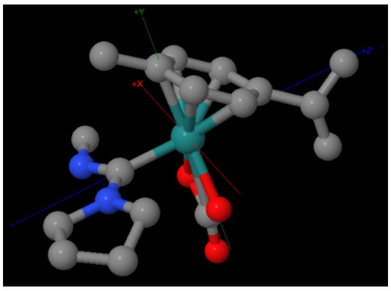

%V Free	%V Buried	% V Tot/V Ex
73.2	**26.8**	99.9

Quadrant	V f	V b	V t	%V f	%V b
SW	33.0	11.9	44.9	**73.5**	26.5
NW	32.6	12.2	44.9	**72.8**	27.2
NE	33.0	11.8	44.9	**73.6**	26.4
SE	32.8	12.1	44.9	**73.1**	26.9

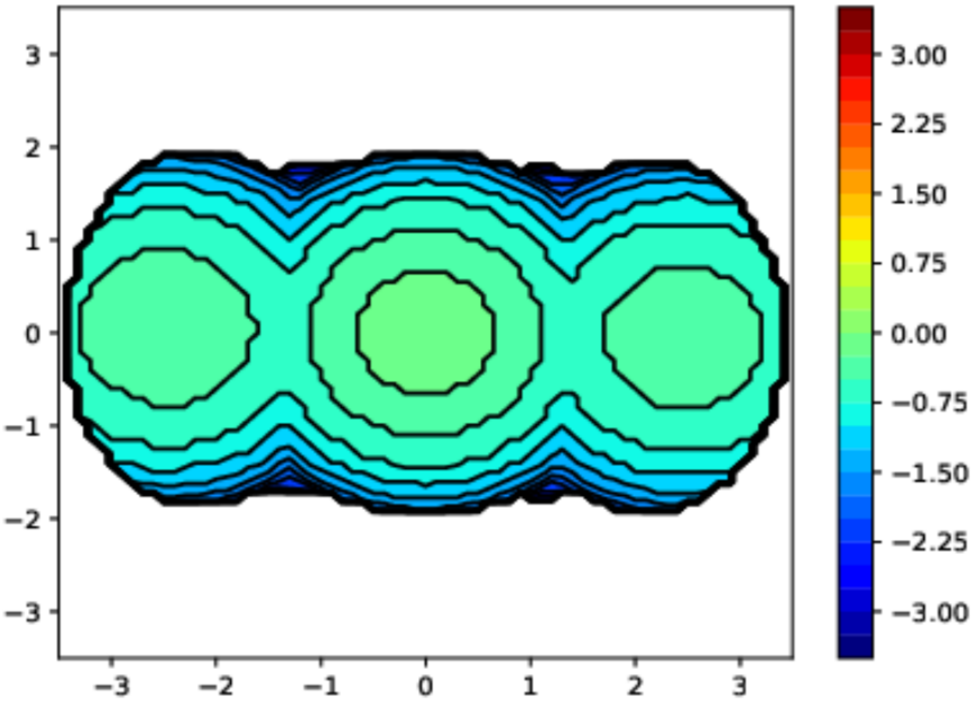

Figure S107: XRD structure with the specific orientation used to determine %V_{bur} (top left). Data obtained from calculating %V_{bur} (top right). Steric map of **Ru-2b** (bottom).

%V_{bur} of Ru-2c

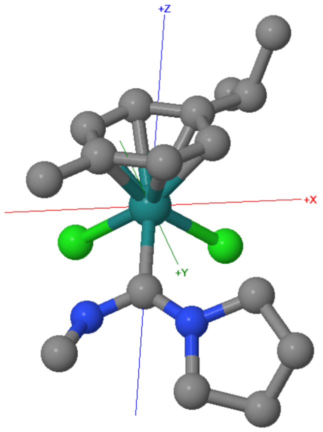

%V Free	%V Buried	% V Tot/V Ex
76.7	**23.3**	99.9

Quadrant	V f	V b	V t	%V f	**%V b**
SW	35.4	9.5	44.9	**78.8**	21.2
NW	35.9	8.9	44.9	**80.1**	19.9
NE	33.4	11.4	44.9	**74.5**	25.5
SE	32.8	12.0	44.9	**73.1**	26.9

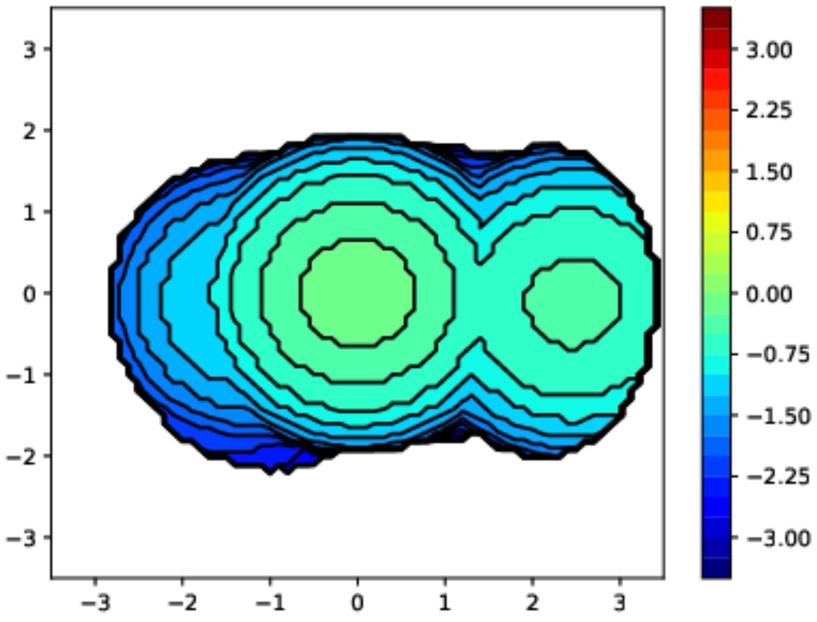

Figure S108: XRD structure with the specific orientation used to determine %V_{bur} (top left). Data obtained from calculating %V_{bur} (top right). Steric map of **Ru-2c** (bottom).

%V_{bur} of Ru-5

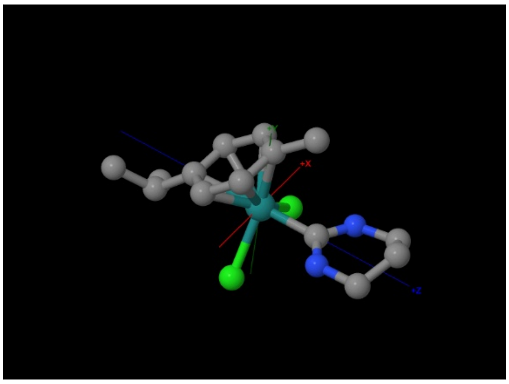

%V Free	%V Buried	% V Tot/V Ex
80.0	**20.0**	99.9

Quadrant	V f	V b	V t	%V f	%V b
SW	36.1	8.8	44.9	**80.4**	19.6
NW	35.7	9.1	44.9	**79.6**	20.4
NE	35.8	9.0	44.9	**79.9**	20.1
SE	35.8	9.0	44.9	**79.9**	20.1

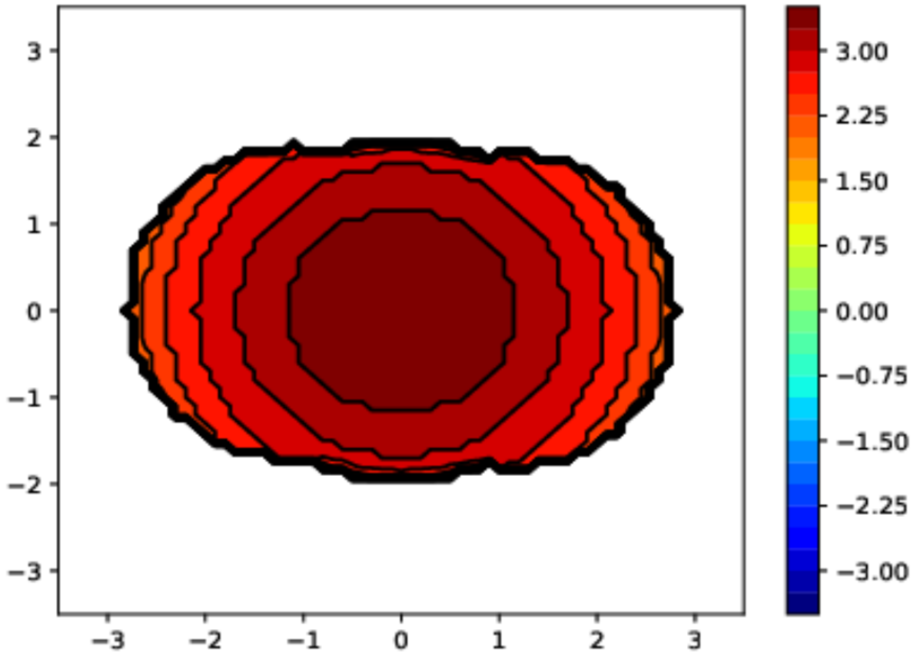

Figure S109: XRD structure including specific orientation used to determine %V_{bur} (top left). Data obtained from calculating %V_{bur} (top right). Steric map of **Ru-5** (bottom).

Thermal Elemental Analysis

CHN of Ru-1

Sample name	Anal. Date	Inj. Time	(mg)	Nitrogen	Carbon	Hydrogen
**GL_Ru-CNMe_1**	18.08.2025	10:56	2.004	3.93	41.74	4.93
**GL_Ru-CNMe_2**	18.08.2025	11:05	3.646	4.03	41.72	4.95
**GL_Ru-CNMe_3**	18.08.2025	11:13	2.402	3.76	41.34	4.88
3 Sample(s) in Group No : 3						
Component Name	Average	Std. Dev.	% Rel. S. D.		theor.	dev.
**Nitrogen**	**3.91**	**0.137**	**3.494**		**4.03**	**-0.12**
**Carbon**	**41.6**	**0.225**	**0.542**		**41.51**	**0.09**
**Hydrogen**	**4.92**	**0.036**	**0.733**		**4.93**	**-0.01**

Figure S110: Elemental analysis of **Ru-1**.

CHN of Ru-2a

Thermal Elemental Analysis

Name: **Gianluca Righetti**

Summarize Results
Date : 20.03.2025 09:00:19
Method Name : CHN_fluorinated_Compounds
Method Filename : CHN_F 2025_03_19.mth

Sample name	Anal. Date	Inj. Time	(mg)	% Nitrogen	% Carbon	% Hydrogen
**GL_01_073_1**	19.03.2025	12:15	1.794	8.45	49.12	7.25
**GL_01_073_2**	19.03.2025	12:23	2.092	8.54	49.19	7.27
**GL_01_073_3**	19.03.2025	12:31	1.792	8.51	49.33	7.28
3 Sample(s) in Group No : 9						
Component Name	Average	Std. Dev.	% Rel. S. D.		theor.	dev.
**Nitrogen**	**8.50**	**0.046**	**0.539**		**8.58**	**-0.08**
**Carbon**	**49.21**	**0.107**	**0.217**		**49.08**	**0.13**
**Hydrogen**	**7.27**	**0.015**	**0.210**		**7.21**	**0.06**

Figure S111: Elemental analysis of **Ru-2a**.

CHN of Ru-2b

Thermal Elemental Analysis

Name: **Gian Luca Righetti**

Summarize Results
Date : 30.01.2025 14:30:32
Method Name : CHN_fluorinated_Compounds
Method Filename : CHN_F 2025_01_30.mth

Sample name	Anal. Date	Inj. Time	(mg)	% Nitrogen	% Carbon	% Hydrogen
**GL_V18_C_Carb_1**	30.01.2025	12:07	1.660	6.38	48.79	6.31
**GL_V18_C_Carb_2**	30.01.2025	12:16	1.910	6.44	48.65	6.29
**GL_V18_C_Carb_3**	30.01.2025	12:24	2.714	6.57	48.86	6.37
3 Sample(s) in Group No : 2						
Component Name	Average	Std. Dev.	% Rel. S. D.		theor.	dev.
**Nitrogen**	**6.46**	**0.097**	**1.503**		**6.88**	**-0.42**
**Carbon**	**48.77**	**0.107**	**0.219**		**50.11**	**-1.34**
**Hydrogen**	**6.32**	**0.042**	**0.658**		**6.43**	**-0.11**

Figure S112: Elemental analysis of **Ru-2b**.

CHN of Ru-2c

Thermal Elemental Analysis

Name: **Gian Luca Righetti**

Summarize Results
Date : 13.02.2025 13:59:55
Method Name : CHN_fluorinated_Compounds
Method Filename : CHN_F 2025_02_12.mth

Sample name	Anal. Date	Inj. Time	(mg)	% Nitrogen	% Carbon	% Hydrogen
**GL-026-Cl2_1**	12.02.2025	16:13	1.904	6.30	45.59	5.95
**GL-026-Cl2_2**	12.02.2025	16:21	2.730	6.51	45.24	6.13
**GL-026-Cl2_3**	12.02.2025	16:30	1.824	6.35	46.10	6.02
3 Sample(s) in Group No : 12						
Component Name	Average	Std. Dev.	% Rel. S. D.		theor.	dev.
**Nitrogen**	**6.39**	**0.110**	**1.718**		**6.70**	**-0.31**
**Carbon**	**45.64**	**0.432**	**0.948**		**45.93**	**-0.29**
**Hydrogen**	**6.03**	**0.091**	**1.504**		**6.26**	**-0.23**

Figure S113: Elemental analysis of **Ru-2c**.

CHN of Ru-5

Sample name	Anal. Date	Inj. Time	(mg)	Nitrogen	Carbon	Hydrogen
**GL_Ru-CDC_1**	18.08.2025	11:29	1.770	7.07	43.42	5.70
**GL_Ru-CDC_2**	18.08.2025	11:38	2.272	7.13	43.27	5.71
**GL_Ru-CDC_3**	18.08.2025	11:46	1.780	7.04	43.10	5.64
3 Sample(s) in Group No : 4						
Component Name	Average	Std. Dev.	% Rel. S. D.		theor.	dev.
**Nitrogen**	**7.08**	**0.046**	**0.647**		**7.18**	**-0.10**
**Carbon**	**43.26**	**0.160**	**0.370**		**43.08**	**0.18**
**Hydrogen**	**5.68**	**0.038**	**0.666**		**5.68**	**0.00**

Figure S114: Elemental analysis of **Ru-5**.

CHN of Ru-6

Thermal Elemental Analysis

Name: **Gianluca Righetti**

Summarize Results
Date : 15.07.2025 16:41:33
Method Name : CHN_fluorinated_Compounds
Method Filename : CHN_F 2025_07_15.mth

Sample name	Anal. Date	Inj. Time	(mg)	% Nitrogen	% Carbon	% Hydrogen
**GL_01_205a**	15.07.2025	12:24	3.024	7.49	41.37	5.91
**GL_01_205b**	15.07.2025	12:32	2.712	7.45	41.40	5.92
**GL_01_205c**	15.07.2025	12:40	1.692	7.31	41.26	5.80
3 Sample(s) in Group No : 6						
Component Name	Average	Std. Dev.	% Rel. S. D.		theor.	dev.
**Nitrogen**	**7.42**	**0.095**	**1.274**		**7.41**	**0.01**
**Carbon**	**41.34**	**0.074**	**0.178**		**41.27**	**0.07**
**Hydrogen**	**5.88**	**0.067**	**1.133**		**5.86**	**0.02**

Figure S115: Elemental analysis of **Ru-6**.

Crystallographic Details

General Procedure for Crystal Structure Determination

Crystals of the compounds **Ru-1**, **Ru-2a**, **Ru-2b**, **Ru-2c**, **Ru-5**, and **Ru-6** were mounted in parabar oil under ambient conditions and transferred to a stream of nitrogen (173 K) except for **Ru-2b** (measured at 300.26(10) K). Data were collected on RIGAKU diffractometers (Synergy S or XtaLAB Synergy R) equipped with HyPix-Arc 100 area detectors and mirror optics using Mo Kα (λ = 0.71073 Å) or Cu Kα (λ = 1.54184 Å) radiation. The data collection strategies involved ω scans with rotation widths of 0.5° and exposure times ranging from 0.1 to 0.8 s, with crystal-to-detector distances between 31.0 and 43.0 mm.

Unit cell constants and orientation matrices were obtained from least-squares refinement of the setting angles of reflections in the respective θ ranges. Data reduction was carried out using the CrysAlisPro[S5] software. Lorentz and polarization corrections were applied, and absorption corrections were performed by Gaussian integration over multifaceted crystal models combined with empirical corrections using spherical harmonics implemented in SCALE3 ABSPACK. For **Ru-2b** and **Ru-6** additional analytical corrections following the method of Clark and Reid[S6] were applied.

The structures were solved by intrinsic phasing with SHELXT[S7], which revealed the positions of all non-hydrogen atoms. Refinements were performed on F^2 using full-matrix least-squares methods within SHELXL-2014/7[S8] as implemented in OLEX2.4.[S9] All non-hydrogen atoms were refined anisotropically. Hydrogen atoms were generally placed in calculated positions and refined using a riding model, with isotropic displacement parameters set to 1.2Ueq of the parent atom (1.5Ueq for methyl groups). In selected cases, hydrogen atoms were located from Fourier difference maps and refined freely: H2 in **Ru-2c**, the H1 in **Ru-2b**; H1 and H3 in **Ru-2a**, and H1 and H2 in **Ru-6**.

The crystal of **Ru-2b** underwent cracking at low temperatures due to a phase transition to P1, thus data were collected at room temperature. For **Ru-2a**, selected regions of the structure were modeled as disordered, and occupancies were refined using a free variable constrained to 100%. Selected crystallographic parameters are collected in Tables S3 and S4. CCDC 2487355 (**Ru-1**), 2487353 (**Ru-2a**), 2487352 (**Ru-2b**), 2487351 (**Ru-2c**), 2487354 (**Ru-5**) and 2487350 (**Ru-6**) contain the supplementary crystallographic data for this paper. These data can be obtained free of charge via www.ccdc.cam.ac.uk/data_request/cif, or by emailing data_request@ccdc.cam.ac.uk, or by contacting The Cambridge Crystallographic Data Centre, 12 Union Road, Cambridge CB2 1EZ, UK; fax: +44 1223 336033.

Table S2: Selected crystallographic parameters for **Ru-2a-c** and reference complexes **Ru-3-5.**

Complex	**Ru-2a**	**Ru-2b**	**Ru-2c**	**Ru-3**[S10]	**Ru-4**[S1]	**Ru-5**
Ru1–C1 /Å	2.0724(11)	2.080(2)	2.0735(17)	2.142(4)	2.0084(19)	2.052(2)
C1–N1 /Å	1.3451(15)	1.337(3)	1.347(2)	1.375(5)	1.286(3)	1.330(4)
C1–N2 /Å	1.3374(14)	1.332(3)	1.328(2)	1.370(5)	—	1.333(3)
Ru1···H1 /Å	2.861	3.796	2.846	—	—	2.967
N1–C1–N2 / °	117.03(10)	115.3(2)	117.44(15)	102.0(3)	—	117.4(2)
Σ∠N1 / ° [c)]	355	360	357	360	360	360
Σ∠N2 / ° [c)]	360	360	357	360	—	359
%V_{bur} [d)]	23.2	26.8	23.3	28.0	21.0	20

a) sum of angles with vertex N1 and N2, respectively.

Table S3: Crystallographic details for **Ru-1**, **Ru-2a**, **Ru-2b**, **Ru-2b**, **Ru-5** and **Ru-6.**

Complex	**Ru-1**	**Ru-2a**	**Ru-2b**
CCDC No	2487355	2487353	2487352
empirical formula	$C_{12}H_{17}Cl_2NRu$	$C_{20}H_{35}Cl_2N_3Ru$	$C_{17}H_{26}N_2O_3Ru$
Formula weight	347.23	489.48	407.47
Temperature (K)	173.00(10)	173.00(10)	300.26(10)
Crystal system	monoclinic	monoclinic	monoclinic
Space group	$P2_1/c$	$P2_1/n$	$P2_1/c$
a (Å)	19.55034(8)	10.69797(14)	8.13073(13)
b (Å)	11.01135(5)	9.17128(11)	11.29004(18)
c (Å)	12.79041(5)	22.3384(3)	19.3239(3)
α (°)	90	90	90
β (°)	99.3053(4)	92.6898(11)	101.8491(14)
γ (°)	90	90	90
Volume ($Å^3$)	2717.23(2)	2189.30(5)	1736.07(5)
Z	8	4	4
ρ_{calc} (g/cm^3)	1.698	1.485	1.559
μ (mm^{-1})	12.731	0.97	0.919
F(000)	1392.0	1016.0	840.0
Crystal size (mm^3)	0.249×0.164×0.064	0.268×0.193×0.086	0.142×0.124×0.065
Radiation	Cu Kα (1.54184)	Mo Kα (0.71073)	Mo Kα (0.71073)
2Θ range (°)	4.58–148.974	4.148–61.014	4.202–61.014
Index ranges	–24≤h≤24, –13≤k≤13, –15≤l≤15	–15≤h≤14, –13≤k≤13, –31≤l≤31	–11≤h≤11, –16≤k≤16, –27≤l≤27
Reflections collected	56430	65779	52124
Independent reflections	5543 [R_{int} = 0.0267, $R\sigma$ = 0.0103]	6679 [R_{int} = 0.0243, $R\sigma$ = 0.0124]	5296 [R_{int} = 0.0274, $R\sigma$ = 0.0138]
Data/restraints/parameters	5543/0/298	6679/26/268	5296/73/244
Goodness-of-fit on F^2	1.186	1.05	1.058
Final R indexes [I≥2σ(I)]	0.0208 / 0.0565	0.0183 / 0.0470	0.0324 / 0.0922
Final R indexes [all data]	0.0209 / 0.0566	0.0202 / 0.0477	0.0395 / 0.0957
Largest diff. peak/hole (e $Å^{-3}$)	0.78/–0.41	0.39/–0.40	0.78/–0.72

Table S4: Crystallographic details for **Ru-1**, **Ru-2a**, **Ru-2b**, **Ru-2b**, **Ru-5** and **Ru-6.**

Complex	**Ru-2c**	**Ru-5**	**Ru-6**
CCDC No	2487351	2487354	2487350
Empirical formula	$C_{16}H_{26}Cl_2N_2Ru$	$C_{15}H_{24}N_2Cl_4Ru$	$C_{13}H_{22}Cl_2N_2Ru$
Formula weight	418.36	475.23	378.29
Temperature (K)	173.00(10)	173.00(10)	173.00(10)
Crystal system	orthorhombic	triclinic	triclinic
Space group	Pbca	P-1	P-1
a (Å)	11.43884(6)	8.8497(2)	8.0151(3)
b (Å)	15.36310(9)	10.67790(10)	10.3158(4)
c (Å)	19.58592(10)	11.6417(2)	10.4456(3)
α (°)	90	69.3130(10)	69.794(3)
β (°)	90	75.8530(10)	68.844(3)
γ (°)	90	76.1410(10)	78.767(3)
Volume ($Å^3$)	3441.95(3)	983.62(3)	753.44(5)
Z	8	2	2
ρ_{calc} (g/cm^3)	1.615	1.605	1.667
μ (mm^{-1})	10.176	1.338	11.549
F(000)	1712.0	480.0	384.0
Crystal size (mm^3)	0.396×0.125×0.053	0.23×0.16×0.10	0.169×0.124×0.085
Radiation	Cu Kα (1.54184)	Mo Kα (0.71073)	Cu Kα (1.54184)
2Θ range (°)	9.03–148.98	3.798–59.994	9.164–149.004
Index ranges	$-14 \le h \le 14$, $-19 \le k \le 19$, $-24 \le l \le 15$	$-12 \le h \le 12$, $-15 \le k \le 15$, $-16 \le l \le 16$	$-10 \le h \le 9$, $-12 \le k \le 12$, $-13 \le l \le 13$
Reflections collected	34841	60077	30311
Independent reflections	3516 [R_{int} = 0.0243, R_σ = 0.0119]	5729 [R_{int} = 0.0370, R_σ = 0.0164]	3054 [R_{int} = 0.0452, R_σ = 0.0169]
Data/restraints/parameters	3516/0/199	5729/0/206	3054/0/176
Goodness-of-fit on F^2	1.101	1.072	1.081
Final R indexes [$I \ge 2\sigma(I)$]	0.0187 / 0.0461	0.0319 / 0.0840	0.0269 / 0.0721
Final R indexes [all data]	0.0189 / 0.0462	0.0340 / 0.0856	0.0272 / 0.0724
Largest diff. peak/hole (e $Å^{-3}$)	0.44/–0.37	1.81/–1.73	1.00/–0.88

References

(S1) G, Rigoni, M, Casciotti, T. R, Belderrain, P. J, Pérez and M. Albrecht, *Organometallics* 2024, **43**, 2843–2849.

(S2) D. N, Kirk, H. C, Toms, C, Douglas, K. A, White, K. E, Smith, S, Latif and R. W. P. A. Hubbard, *J. Chem. Soc., Perkin. Trans. 2* 1990, 1567–1594.

(S3) T, Janeczko, A, Świzdor, J, Dmochowska-Gładysz, A, Białońska, Z, Ciunik and E. Kostrzewa-Susłow, *J. Mol. Catal. B.* 2012, **82**, 24–31.

(S4) L, Falivene, Z, Cao, A, Petta, L, Serra, A, Poater, R, Oliva, V, Scarano and L. Cavallo, *Nat. Chem.* 2019, **11**, 872–879.

(S5) Oxford Diffraction **2018**. CrysAlisPro (Version 1.171.40.37a). Oxford Diffraction Ltd., Yarnton, Oxfordshire, UK.

(S6) R. C, Clark and J. S. Reid, *Acta Cryst.* 1995, **A51**, 887–897

(S7) G. M. Sheldrick, *Acta Cryst.* 2015, **A71**, 3–8.

(S8) G. M. Sheldrick, *Acta Cryst.* 2015, **C71**, 3–8.

(S9) O. V. Dolomanov, L.J. Bourhis, R. J. Gildea, J. A. K, Howard and H. Puschmann, *J. Appl. Cryst.* 2009, **42**, 339–341.

(S10) C, Lo, R, Cariou, C, Fischmeister and P. H. Dixneuf, *Adv. Synth. Catal.* 2007, **349**, 546–550.
